# Palladium-catalyzed enantioselective Heck alkenylation of trisubstituted allylic alkenols: a redox-relay strategy to construct vicinal stereocenters†
†Electronic supplementary information (ESI) available. See DOI: 10.1039/c6sc04585e



**DOI:** 10.1039/c6sc04585e

**Published:** 2016-12-09

**Authors:** Chun Zhang, Brandon Tutkowski, Ryan J. DeLuca, Leo A. Joyce, Olaf Wiest, Matthew S. Sigman

**Affiliations:** a Department of Chemistry , University of Utah , 315 South 1400 East , Salt Lake City , Utah 84112 , USA; b Department of Chemistry and Biochemistry , University of Notre Dame , Notre Dame , Indiana 46556-5670 , USA; c Process Research & Development , Merck Research Laboratories , Rahway , New Jersey 07065 , USA

## Abstract

An enantioselective, redox-relay Heck alkenylation of trisubstituted allylic alkenol substrates has been developed. This process enables the construction of vicinal stereocenters in high diastereo- and enantioselectivity and allows the formation of enolizable α-carbonyl methyl-substituted stereocenters with no observed epimerization under the reported reaction conditions.

## Abstract 2

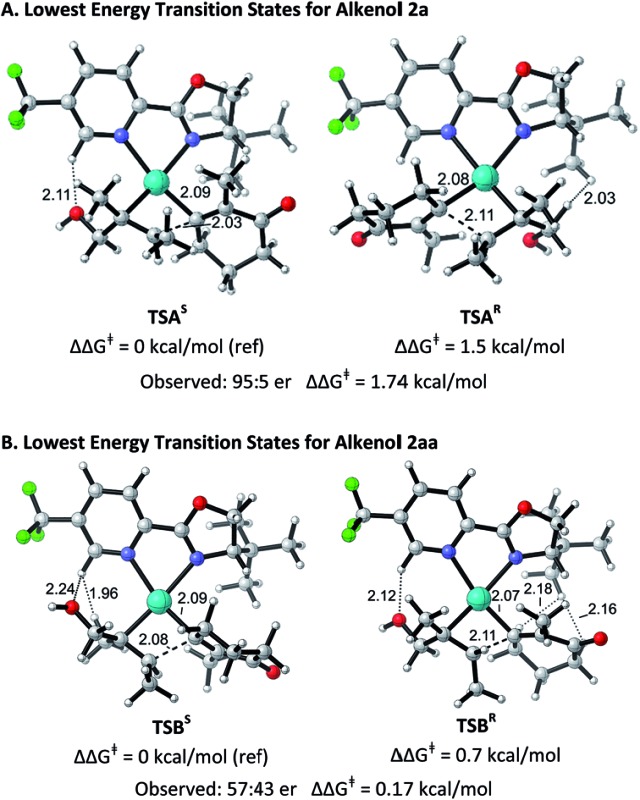
An enantioselective, redox-relay Heck alkenylation of trisubstituted allylic alkenol substrates has been developed.

## Introduction

**Scheme 1:**
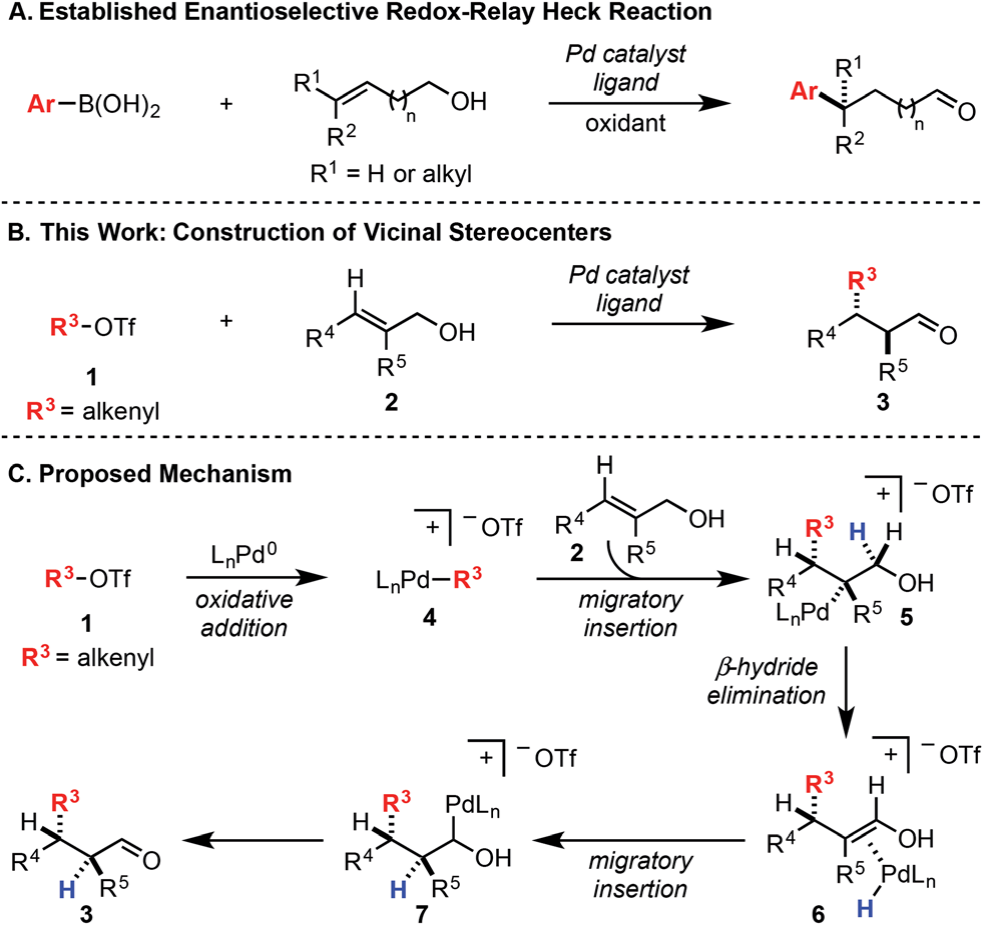
(A) Previous work with di- and trisubstituted alkenes to form tertiary and quaternary stereocenters. (B) Proposed synthesis of vicinal stereocenters using a redox-relay Heck strategy. (C) Mechanistic rationale for the construction of vicinal stereocenters.

In a Heck reaction using multi-substituted alkenes, the initial *syn*-carbopalladation sets two vicinal stereocenters by virtue of the migratory insertion process.^1^ Unfortunately, the resultant Pd–alkyl undergoes facile β-hydride elimination, which generally eliminates the stereochemistry imparted by migratory insertion. Recently, we have reported a modern variant of the Heck reaction, wherein the directionality and stereochemical fidelity of β-hydride elimination can be controlled and, thus, the initial stereochemical consequence of migratory insertion is not lost. Termed redox-relay Heck reactions, the unsaturation of the alkene is conserved as it is transferred to a different position on the alkyl chain, most commonly by oxidation of an alcohol to a carbonyl.^2^ These reactions have been rendered enantioselective and are effective on both disubstituted alkenes to form tertiary stereocenters and trisubstituted alkenes to form quaternary stereocenters (Scheme 1A).^2^ However, the potential power of the *syn* migratory insertion has not been realized as only a single stereocenter has been set.^2–5^ Therefore, we set out to investigate if vicinal centers can be forged through the use of trisubstituted alkenols of type **2** in enantioselective redox-relay Heck reactions (Scheme 1B). In this case, such stereocenters^6^ could be strategically constructed if we took advantage of the propensity for the alkenyl electrophile (**1**) to add at the alkene carbon distal to the alcohol functionality, producing a new Csp^2^–Csp^3^ bond and set an adjacent stereocenter in a single migratory insertion event.^7^ In order to render this transformation enantioselective, the chiral Pd–ligand complex must differentiate the two similar prochiral faces of sterically encumbered trisubstituted alkenol **2**, which can be challenging on the basis of past reports.^8^

Mechanistically, we propose the reaction initiates with oxidative addition of alkenyl triflate **1** with Pd(0) to produce cationic Pd–alkenyl intermediate **4** (Scheme 1C).^2*c*^ Alkenol **2** can undergo migratory insertion into the Pd–alkenyl bond to furnish Pd–alkyl intermediate **5**. The cationic Pd–alkyl species **5** undergoes β-hydride elimination to deliver Pd–enol intermediate **6**, which subsequently leads to reinsertion to yield intermediate **7**. This type of Pd–alkyl intermediate is unique since the alcohol and Pd-catalyst are bonded to the same carbon atom. As a result, alcohol oxidation occurs (either through β-hydride elimination or an E_2_-type elimination^9^), reminiscent of the final step in the Wacker process.^10^ This yields final product **3** and Pd(0), closing the catalytic cycle.

## Results and discussion

Preliminary investigations to develop this process were conducted with trisubstituted alkenol **2a** using reaction conditions previously shown to effectively promote such processes. Unfortunately, commonly employed amide solvents, such as dimethylacetamide (DMA) and dimethylformamide (DMF), produced the desired redox-relay Heck product (**3a**) in low yield and enantioselectivity (entries 1 and 2, Table 1). A variety of solvents were then evaluated and only ester-derived solvents resulted in significant improvements. For example, ethyl acetate (EtOAc) enhanced the yield of product **3a** to 45% and enantioselectivity to 94 : 6 (entry 3). Isopropyl acetate (*i*-PrOAc) and ethyl isobutyrate also resulted in similar yield and selectivity (entries 4 and 5). However, changing the solvent to ethyl pivalate delivered product **3a** in 62% yield and 95 : 5 er. It should be noted that the enantioselectivity can be correlated to the natural bond orbital (NBO) charge of the oxygen or nitrogen atom of the ester or amide functional group of the solvent (see ESI for details†).^11^ Next, an assortment of bases was screened, as an equivalent of triflic acid is formed with each catalytic turnover. The addition of 1.5 equivalents of NEt_3_ slowed the desired reaction and resulted in trace product formation (entry 7). In contrast, adding 1.5 equivalents of Li_2_CO_3_ improved the yield of product **3a** to 74% with no erosion in selectivity (entry 8). Exchanging the cation of the carbonate base from lithium to sodium or potassium resulted in slightly lower yields (entries 9 and 10).

**Table 1:** Reaction optimization^*a*^

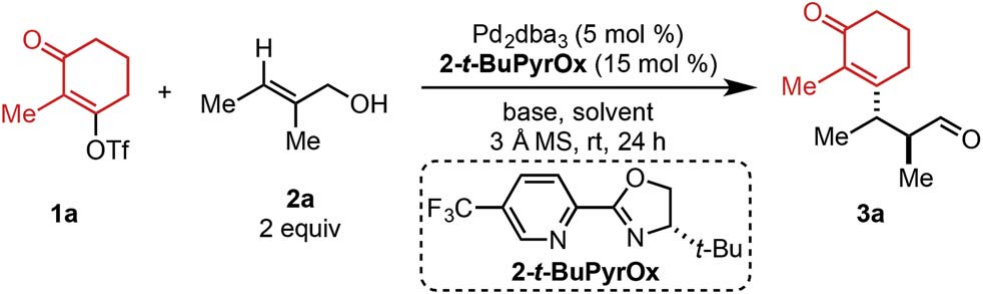					
entry	solvent	base	% yield	dr	er
1	DMA	—	14	>20 : 1	71 : 29
2	DMF	—	15	>20 : 1	75 : 25
3	EtOAc	—	45	>20 : 1	94 : 6
4	*i*-PrOAc	—	39	>20 : 1	94 : 6
5	Ethyl isobutyrate	—	49	>20 : 1	95 : 5
6	Ethyl pivalate	—	62	>20 : 1	95 : 5
7	Ethyl pivalate	NEt_3_	trace	—	—
8	Ethyl pivalate	Li_2_CO_3_	74	>20 : 1	95 : 5
9	Ethyl pivalate	Na_2_CO_3_	70	>20 : 1	95 : 5
10	Ethyl pivalate	K_2_CO_3_	61	>20 : 1	95 : 5

^*a*^Each entry represents the isolated yield on 0.25 mmol scale. er values were determined by SFC or HPLC. Entries 7–10 used 1.5 equiv of base.

Using ethyl pivalate as solvent and Li_2_CO_3_ as base, the substrate scope of trisubstituted allylic alkenols was investigated (Table 2). Simple alkyl groups, such as methyl (**3a**) and ethyl (**3b**), at the R^1^ position on the alkene resulted in 74% and 84% yields, respectively. Introduction of a phenethyl moiety (**3c**) at the R^1^ position gave the desired product in 41% yield and 95 : 5 er. The inclusion of methyl (**3d**) and ethyl (**3e**) at the R^3^ position provided the corresponding ketone products in 60% and 54% yields, with increased enantioselectivity (97 : 3 and 98 : 2 er, respectively). An *n*-octyl alkyl group was examined giving 51% yield of **3f** with 98 : 2 er. Evaluation of phenethyl (**3g**), benzyl (**3h**), and phenyl (**3i**) at R^3^ indicated that, as the phenyl group was positioned closer to the resulting carbonyl moiety, slightly lower yields and enantioselectivities were observed. In addition, the reaction could tolerate an isopropyl (*i*-Pr) group at the R^3^ position furnishing product **3j** in 45% yield and 98 : 2 er. A trimethylsilyl (TMS) group could also be positioned α to the resultant carbonyl in high enantioselectivity, albeit in 19% yield (**3k**). The absolute configuration of product **3d** was determined to be (2*S*,3*S*) using electronic circular dichroism (see ESI for details†).^12^ The other products were assigned by analogy to product **3d**.

**Table 2:** Evaluation of alkenol substrates^*a*^

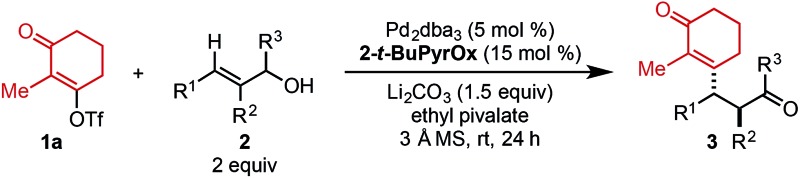
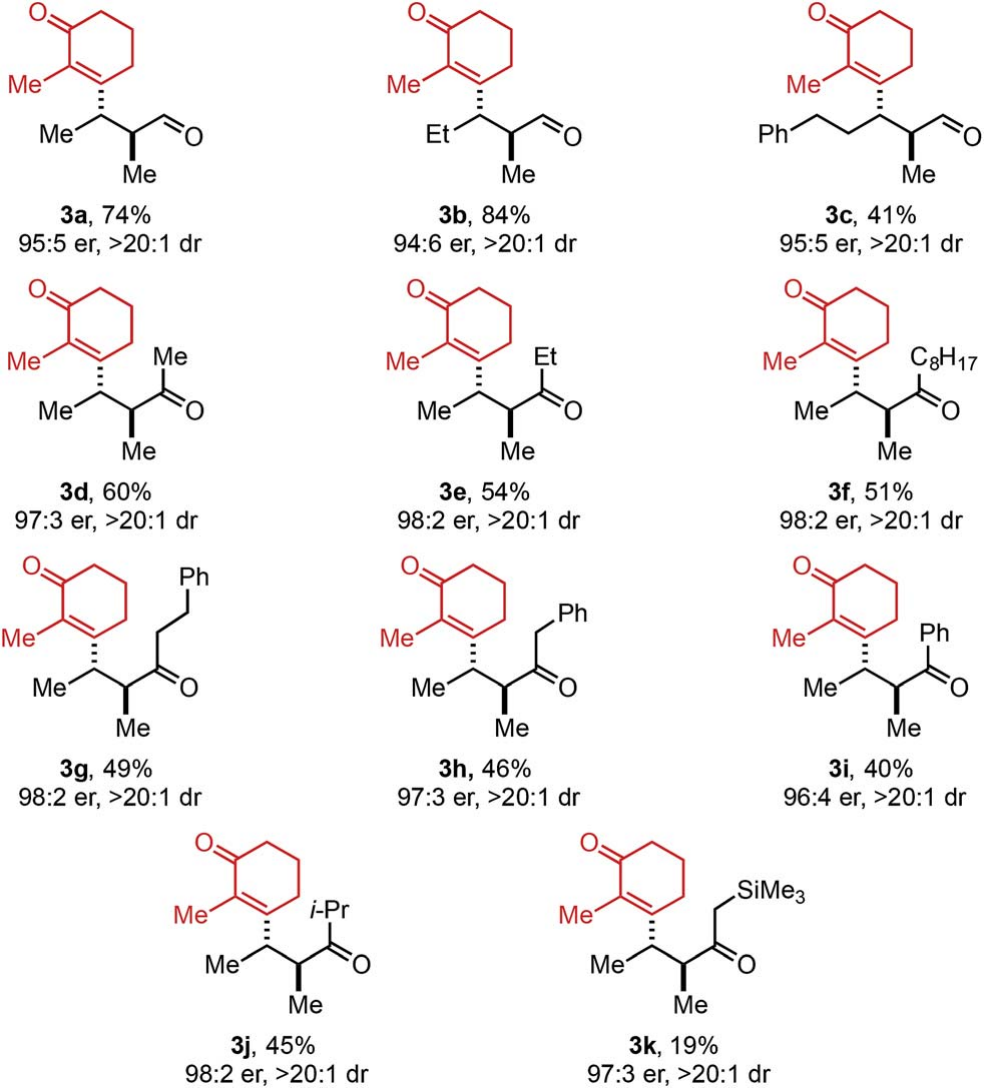

^*a*^Each entry represents the isolated yield on 0.25 mmol scale. er values were determined by SFC or HPLC.

**Scheme 2:**
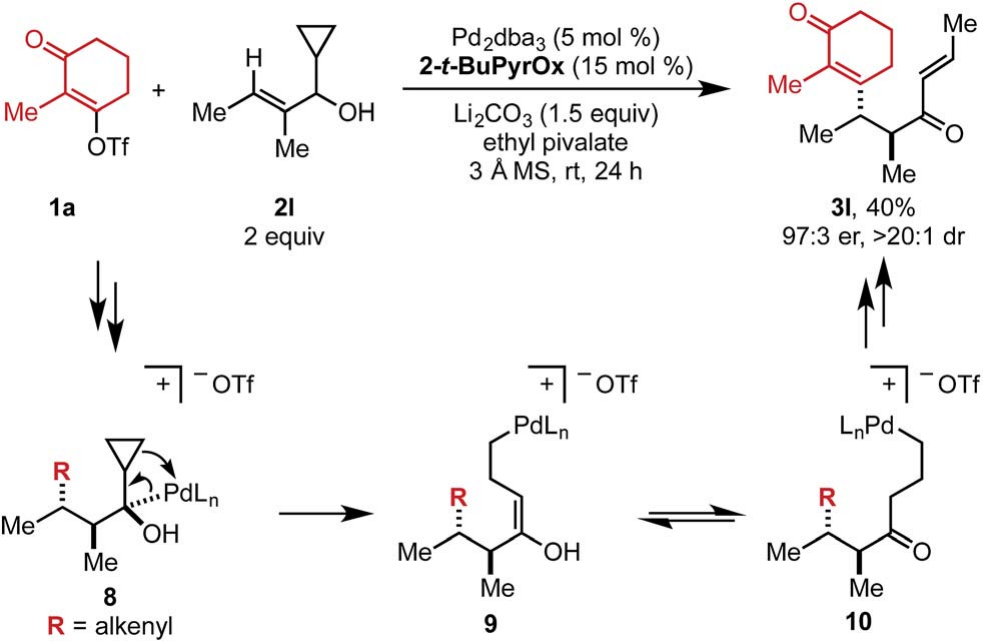
Putative mechanism for the Heck/cyclopropyl ring opening cascade.

When cyclopropyl-containing substrate **2l** was subjected to the reaction conditions, the ring opening product (**3l**) was isolated in 40% yield and 97 : 3 er (Scheme 2). This α,β-unsaturated product could arise through Pd-mediated ring opening of β-cyclopropyl Pd–alkyl intermediate **8** to yield enol **9** that could tautomerize to ketone **10**. Primary Pd–alkyl intermediate **10** can then undergo β-hydride elimination to produce a terminal alkene that isomerizes to the internal position to produce α,β-unsaturated product **3l**.^13^ Ultimately, this confirms that the Pd-center migrates to the carbon attached to the alcohol.

Next, the scope of alkenyl triflates was explored including a variety of tri-and tetrasubstituted alkenyl triflates (**1**, Table 3). Starting with 2-substituted cyclohexenone triflates, an 85% yield was isolated for the ethyl-substituted product (**3m**). As the apparent steric impact of the aliphatic group was increased to Bn (**3n**) and *i*-Pr (**3o**), lower product yields were observed. In the case of a 2-bromo-substituted triflate, only 22% yield was isolated (**3p**). Enol triflates containing a methyl-substituted cyclopentenone (**1q**) and 5-membered lactone (**1r**) delivered the corresponding products in excellent yield and good selectivity. Reaction with β-keto ester derived (*Z*)-enol triflate yielded the (*Z*)-alkene product in 72% yield (**3s**). In contrast, reaction with the (*E*)-enol triflate gave a near equal mixture of (*E*)- and (*Z*)-tetrasubstituted alkene products in 56% yield (**3t**). Interestingly, the (*E*)-alkene product isomer has a 97 : 3 er, while the (*Z*)-alkene isomer product has a 91 : 9 er, the same er as observed when (*Z*)-enol triflate **1s** was used. This result can be explained through isomerization of (*E*)-enol triflate **1t** producing a mixture of (*E*)- and (*Z*)-enol triflates. Reaction with Pd(0) would produce distinct Pd–alkenyl species and ultimately deliver alkene isomeric products with different enantioselectivities (97 : 3 and 91 : 9 er). In addition, a TMS-containing enol triflate furnished product **3u** in 86% yield and 90 : 10 er. An enol triflate containing a phthalimide provided product **3v** in 58% yield and 91 : 9 er. Furthermore, (+)-nootkatone derivative **3w** was produced in 31% yield and 8 : 1 dr. Estrone derivative **3x** was synthesized in 79% yield and >20 : 1 dr. Lastly, the enol triflate derived from cholesterol delivered product **3y** in 26% yield and 8 : 1 dr. During our investigation of chiral triflate reagents (**1w–1y**), we found that the (*R*)-*t*-BuPyrOx ligand gave superior diastereoselectivities (see ESI for additional details†).

**Table 3:** Evaluation of alkenyl triflate scope^*a*^

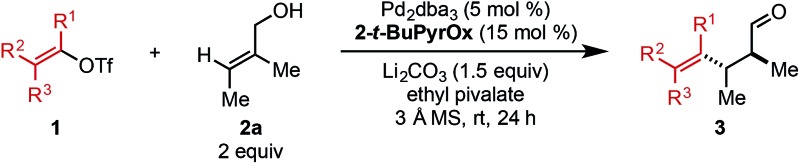
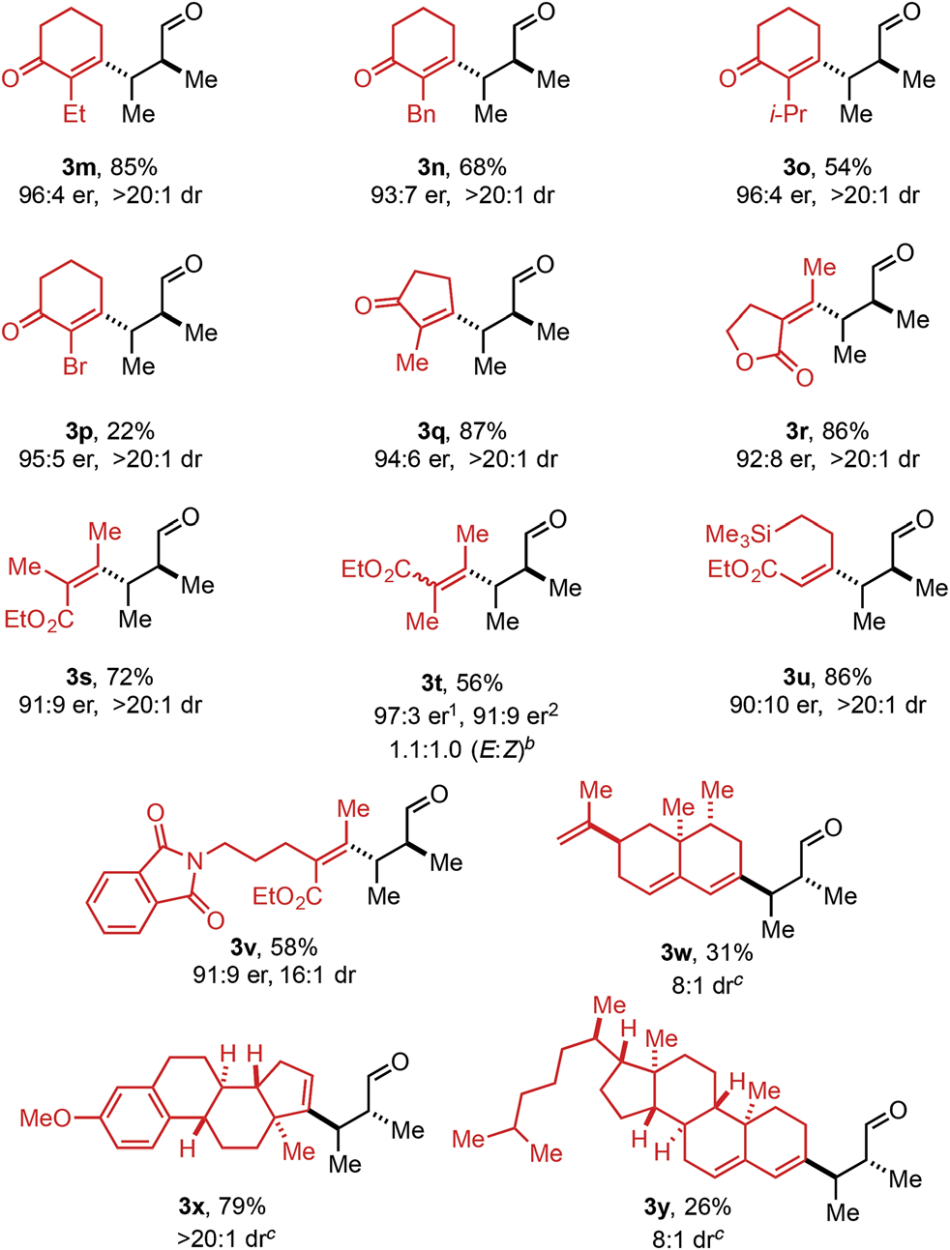

^*a*^Each entry represents the isolated yield on 0.25 mmol scale. er values were determined by SFC or HPLC.

^*b*^A mixture of seperable (*E*)- and (*Z*)-alkene isomers were observed.

^*c*^(*R*)-2-*t*-BuPyrOx was used.

In an effort to expand this redox-relay strategy beyond allylic alkenols, homoallylic alkenol **2z** was subjected to the optimized reaction conditions and gave product **3z** in 31% yield and 89 : 11 er. This result, albeit promising, suggests the current system is optimized for allylic substrates.
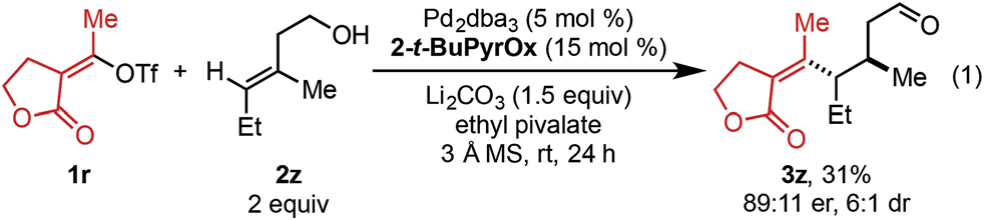


As this alkene class is distinct from others previously evaluated, we were interested in exploring how related substructures performed under these reaction conditions. Accordingly, several alkenes with unique substitution patterns were sdudied (Table 4). The model substrate, (*E*)-alkenol **2a**, yielded the desired redox-relay product in 95 : 5 er. In contrast, (*Z*)-alkenol **2aa** delivered the product in a significantly reduced enantioselectivity (57 : 43 er), underscoring the importance of the substitution pattern on the trisubstituted alkene for face selection. For comparison, disubstituted alkene **2ab** provided the corresponding product in 89 : 11 er, while 1,1-disubstituted alkene **2ac** gave product in 79 : 21 er.

**Table 4:** Evaluation of akenol substitution^*a*^

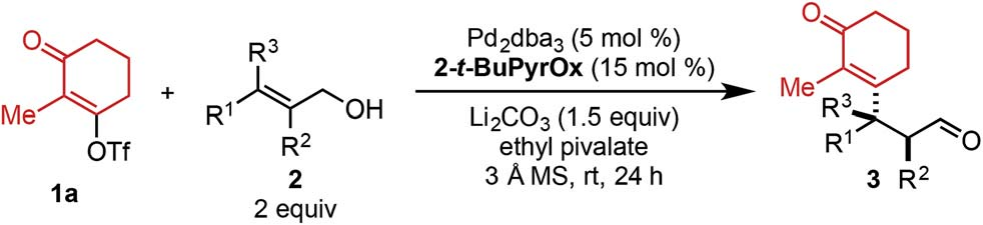
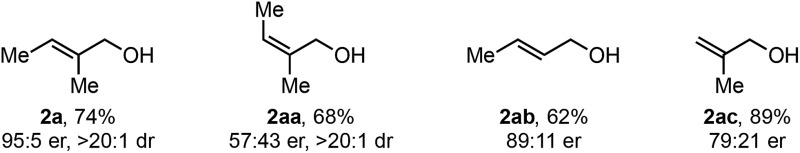

^*a*^The isolated yields of products **3a–3ac** are shown. Each entry represents the isolated yield on 0.25 mmol scale. er values were determined by SFC or HPLC.

**Fig. 1:**
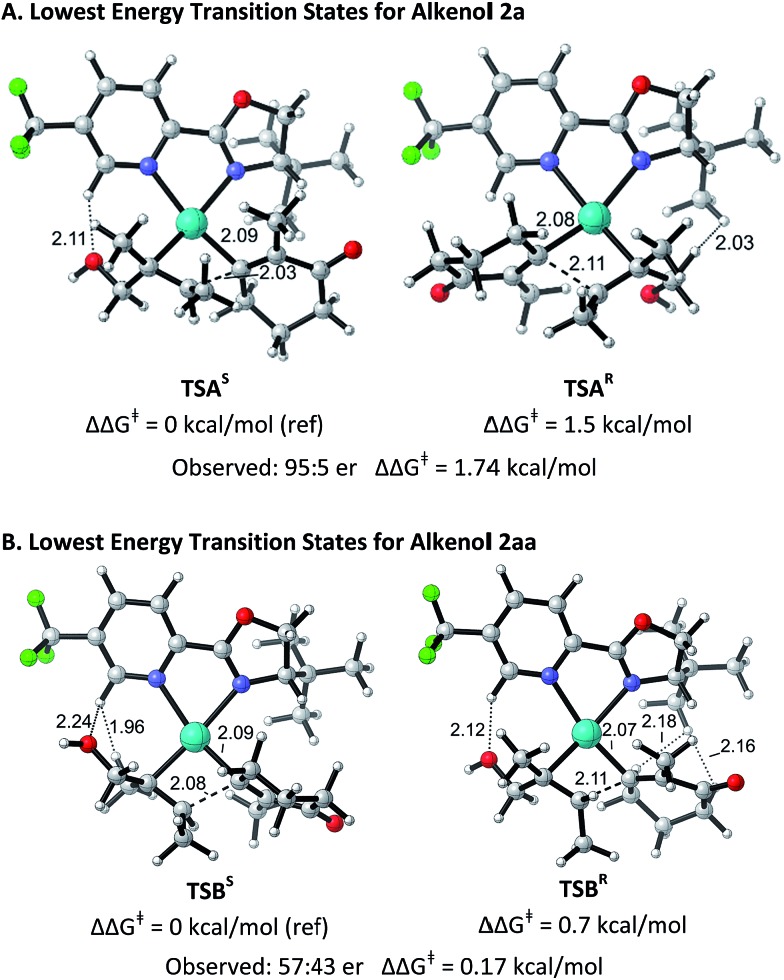
Optimized geometries of the isomers of the transition states for substrates **2a** and **2aa**. Bond distances (in Å) and activation free energies are shown. Data and coordinates for these structures and all structures calculated can be found in the ESI.†

The structural origin of the difference in enantioselectivity between the (*E*)- and the (*Z*)-alkenes was elucidated computationally in analogy to our previous work.^9^ The different conformations and configurations at the metal center in the stereodetermining alkene migratory insertion step for (*E*)-alkenol **2a** and (*Z*)-alkenol **2aa** with electrophile **1a** mediated by a Pd–PyrOx catalyst were calculated using the M06/6-31+G*/LANL2DZ level of theory in G09^14^ (for details see ESI†). The lowest energy transition structures leading to the (*S*)- and (*R*)-products starting from **2a** are shown in Fig. 1A. **TSA^R^** is 1.5 kcal mol^–1^ higher in free energy than **TSA^S^**, in good agreement with the experimentally determined selectivity (95 : 5 er, 1.74 kcal mol^–1^). This free energy difference is due to a through-space steric repulsion between a hydrogen on the carbon α to the hydroxyl group on the (*E*)-alkenol substrate and the *t*-Bu group on the ligand with an H–H distance of 2.03 Å in **TSA^R^**. This unfavorable interaction is not present in **TSA^S^**. Surprisingly, **TSA^S^** has the alkenol positioned proximally to the pyridine ring and the alkenyl group next to the *t*-Bu oxazoline portion of the ligand. As a result, a potentially stabilizing interaction between a hydrogen on the electron deficient pyridine ring and the hydroxyl group of the alkenol substrate with an H–O distance of 2.11 Å and a C–H–O angle of 144.9° is observed.

Fig. 1B shows the lowest energy transition structures **TSB^S^** and **TSB^R^** leading to the (*S*)- and (*R*)-products for (*Z*)-alkenol **2aa**. **TSB^R^** is only 0.7 kcal mol^–1^ higher in free energy than **TSB^S^**. This difference matches, within the expected error of the calculations, the experimentally determined selectivity (57 : 43 er). The free energy difference is smaller for **2aa** due to a combination of several unfavorable interactions present in both transition structures. In **TSB^R^**, there are two close contacts between the *t*-Bu group on the ligand and the alkenyl substrate (H–H = 2.16 Å and H–H = 2.18 Å). In **TSB^S^**, there is a close contact between a hydrogen on an alkenol methyl group and a hydrogen on the pyridine ring of the ligand (H–H = 1.96 Å). The same potentially stabilizing interaction between a hydrogen on the electron deficient pyridine ring of the ligand and the hydroxyl group of the alkenol substrate seen in **TSA^S^** was also observed in both structures. The similarity of the interactions in **TSB^R^** and **TSB^S^** leads to the low selectivity observed experimentally for this substrate.

## Conclusions

In summary, we have developed an enantioselective redox-relay Heck reaction of trisubstituted allylic alkenols to deliver vicinal stereocenters in generally high enantio- and diastereoselectivity. This reaction is conducted under mild reaction conditions that allows the construction of enolizable α-carbonyl methyl-substituted stereocenters with no or little observed epimerization. Systematic evaluation of the substitution pattern on the alkenol substrate has revealed the importance of the double bond geometry to achieve high enantioselectivity. Computational studies were used to explore this finding and showcase a significantly different network of interactions responsible for high enantioselectivity. This information will be utilized in the development of new variants of this reaction with an eye towards expansion to homoallylic alcohols and related substrate classes.
