# Supplementary material for: Palladium-catalyzed enantioselective Heck alkenylation of trisubstituted allylic alkenols: a redox-relay strategy to construct vicinal stereocenters
Source: Chem Sci. 2016 Dec 9;8(3):2277–82. doi: 10.1039/c6sc04585e (PMC5395252; doi:10.1039/c6sc04585e)
Supplement: Supplementary file 1 [file SC-008-C6SC04585E-s001.pdf]

Electronic Supplementary Material (ESI) for Chemical Science.
This journal is © The Royal Society of Chemistry 2016

Palladium-Catalyzed Enantioselective Heck Alkenylation of Trisubstituted Alkenols: A Redox-Relay Strategy to Construct Vicinal Stereocenters

Chun Zhang,[a] Brandon Tutkowski,[b] Ryan J. DeLuca,[a] Leo A. Joyce,[c] Olaf Wiest[b] and Matthew S. Sigman*[a]

[a.] Department of Chemistry, University of Utah, 315 South 1400 East, Salt Lake City, Utah, 84112, USA.

[b.] Department of Chemistry & Biochemistry, University of Notre Dame, Notre Dame, Indiana, 46556, USA.

[c.] Process Research & Development, Merck Research Laboratories, Rahway, New Jersey, 07065, USA.

E-mail: sigman@chem.utah.edu

Supporting Information

Table of Contents

Computational and Correlative Studies S2

Synthesis of Substrates and Ligands S62

Control Experiments S69

X-ray Analysis S70

ECD Analysis S71

Analytical Data for Products S82

Enantiomeric Ratios of Products S96

NMR Spectra of Products S127

Computational and Correlative Studies

A. Effects of Solvents on Enantio-selectivity

i. DFT Calculations

For computationally derived IR vibration values, solvents were geometrically optimized and their frequencies and intensities were calculated using Gaussian 09 software.[1] The functional used for DFT calculation is M06-2x. A triple zeta potential basis-set (jun-cc-pvtz) was chosen based on the evaluation of the M06-2x functional for organic molecules, as triple zeta quality basis sets mostly result to quantitative correlations.[2] Subsequently, Sterimol values of the groups of the solvent were calculated for the geometry optimized structures using Molecular Modeling Pro®.[3] NBO charges were obtained using NBO Version 3.1 in Gaussian 09.[4]

Table S1. Solvent Screen.[a,b]

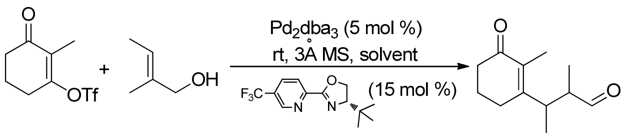

entry	solvent	Experiment 1 yield	Experiment 1 er	Experiment 1 dr	Experiment 2 yield	Experiment 2 er	Experiment 2 dr
1	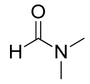	12%	23.5:76.5	>20:1	15%	24.6:75.4	>20:1
2	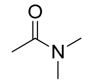	11%	29.1:70.9	>20:1	14%	29.0:71.0	>20:1
3	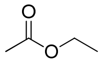	29%	5.9:94.1	>20:1	45%	5.9:94.1	>20:1
4	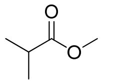	50%	7.7:92.3	>20:1	41%	7.9:92.1	>20:1
5	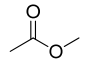	40%	6.5:93.5	>20:1	49%	6.8:93.2	>20:1
6	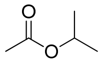	27%	5.7:94.3	>20:1	39%	5.7:94.3	>20:1
7	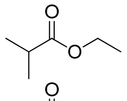	41%	5.5:94.5	>20:1	49%	5.4:94.6	>20:1
8	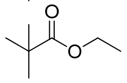	52%	5.5:94.5	>20:1	62%	5.3:94.7	>20:1
9	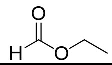	40%	7.1:92.9	>20:1	29%	7.3:92.7	>20:1

[a] **1a** (0.25 mmol), **2a** (0.5 mmol), $Pd_2(dba)_3$ (0.0125 mmol), ligand (0.0375 mmol) ,3Å MS (50 mg), solvent (3 mL), Under an atmosphere of N_2 condition for 24 h. [b] Isolated yield, dr value based on NMR, er value based on SFC or HPLC.

ii. Model Development

First, parameters that describe the steric and electronic perturbations induced by the substituents of the solvent were identified. Using MATLAB® R2014a software[5], a multiple linear regression model was developed toutilize the normalized steric and electronic parameters in predicting the difference in transition state energies ($\Delta\Delta G^{\ddagger}$).These predicted $\Delta\Delta G^{\ddagger}$values were then compared to the measured $\Delta\Delta G^{\ddagger}$ values obtained from an average of two experimental results. A good correlation between the two indicates that the predicted $\Delta\Delta G^{\ddagger}$ approximates the measured $\Delta\Delta G^{\ddagger}$ adequately. The resulting model was then evaluated for accuracy and precision using Leave-One-Out (LOO) validation.[6]

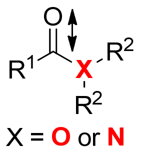

Figure S1.Carbonyl stretch of solvent.

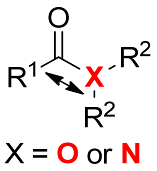

Figure S2.C-X stretch of solvent.

Table S2. Summary of Solvent Screen

Solvent	Trial 1 er[a]	Trial 1 Ratio[b]	**Trial 1 *ΔΔG‡* (kcal mol^{-1})[c]**	Trial 2 er[a]	Trial 2 Ratio[b]	**Trial 2 *ΔΔG‡* (kcal mol^{-1})[c]**	**Average *ΔΔG‡* (kcal mol^{-1})[d]**
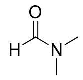	23.5:76.5	3.26	0.6992	24.6:75.4	3.07	0.6636	0.6814
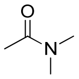	29.1:70.9	2.44	0.5276	29:71	2.45	0.5304	0.5290
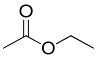	5.9:94.1	15.95	1.6407	5.9:94.1	15.95	1.6407	1.6407
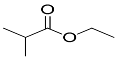	5.4:94.6	17.52	1.6963	5.4:94.6	17.52	1.6963	1.6963
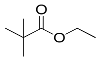	5.4:94.6	17.52	1.6963	5.3:94.7	17.87	1.7080	1.7021
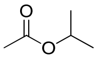	5.7:94.3	16.54	1.6624	5.7:94.3	16.54	1.6624	1.6624
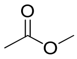	6.5:93.5	14.38	1.5795	6.8:93.2	13.71	1.5509	1.5652
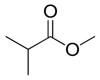	7.7:92.3	11.99	1.4715	7.9:92.1	11.66	1.4550	1.4632
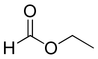	7.1:92.9	13.08	1.5234	7.3:92.7	12.70	1.5056	1.5145

[a]Enantiomeric ratios of two trial runs. [b] Enantiomeric ratios ratios of two trial runs. [c]Difference in ΔΔG‡ calculated from enantiomeric ratios. [d]ΔΔG‡ calculated from average enantiomeric ratios.

Table S3. Crude values of electronic and steric parameters of Solvents

Solvent	NBO of O (Carbonyl)	NBO of X (X = O/N)	NBO of C (Carbonyl)	B1 of -R^1 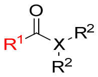	B5 of -R^1 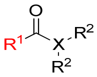	B1 of -XR^2 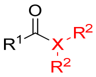	B5 of -XR^2 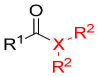	IR frequencies $V_{C=O}$
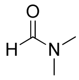	-0.61613	-0.43116	0.53659	1.17000	1.17000	2.05700	3.247279	1805.77
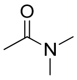	-0.62728	-0.43481	0.67598	1.7000	2.20714	1.860973	3.251941	1773.54
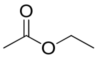	-0.59059	-0.51754	0.79089	1.7000	2.19657	1.52000	3.211042	1853.06
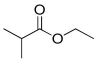	-0.60202	-0.51479	0.80347	2.07980	3.34643	1.52000	4.307413	1840.95
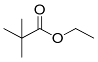	-0.6053	-0.5137	0.81079	4.29077	3.34524	1.52000	4.306221	1834.64
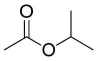	-0.60516	-0.51229	0.79599	1.7000	2.19642	1.52000	4.329018	1843.81
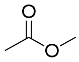	-0.59166	-0.5014	0.787	1.7000	2.19666	1.52000	3.220223	1856.69
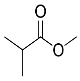	-0.59972	-0.50239	0.79853	2.07287	3.34633	1.52000	3.219782	1846.63
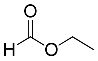	-0.54480	-0.49768	0.65446	1.17000	1.17000	1.52000	3.211948	1891.88

Table S4. Normalized values of solvent electronic and steric parameters

Solvent	NBO of O (Carbonyl)	NBO of X (X = O/N)	NBO of C (Carbonyl)	B1 of -R[1] 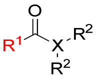	B5 of -R[1] 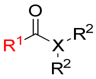	B1 of -XR[2] 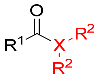	B5 of -XR[2] 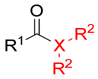	IR frequencies $V_{C=O}$
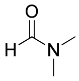	-0.78480	1.78147	-2.11937	-0.83916	-1.38291	2.20074	-0.62917	-0.98850
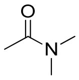	-1.26941	1.67415	-0.662022	-0.27166	-0.17026	1.21904	-0.62060	-1.96036
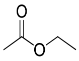	0.32525	-0.75823	0.53938	-0.27166	-0.18262	-0.48854	-0.69580	0.43746
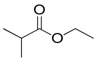	-0.17153	-0.67738	0.67091	0.13501	1.16183	-0.48854	1.32027	0.07230
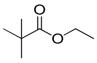	-0.31409	-0.64533	0.74744	2.50238	1.16044	-0.48854	1.31808	-0.11797
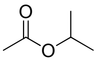	-0.30801	-0.60387	0.59270	-0.27166	-0.18279	-0.48854	1.36000	0.15854
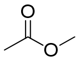	0.27874	-0.28369	0.49871	-0.27166	-0.18251	-0.48854	-0.67892	0.54692
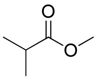	-0.071570	-0.31280	0.61926	0.12759	1.16172	-0.48854	-0.67973	0.24357
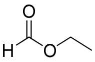	2.31542	-0.17432	-0.88702	-0.83916	-1.38291	-0.48854	-0.69414	1.60803

Equation S1. Crude (a) and normalized (b)linear regression model relating the NBO of X atom of solvents to the $\Delta\Delta G^{\ddagger}$.

(a) Crude Regression Model: $\Delta\Delta G^{\ddagger} = -13.089 NBO_x - 5.0528$

(b) Normalized Regression Model: $\Delta\Delta G^{\ddagger} = 1.3839 - 0.4452 NBO_x$

Table S5. Measured and Predicted differences in transition state energies (ΔΔG‡) derived using equation S1a.

Solvent	Measured *ΔΔG‡* (kcal mol^{-1})	Predicted *ΔΔG‡* (kcal mol^{-1})
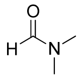	0.6814	0.5907
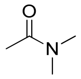	0.5290	0.6384
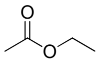	1.6407	1.7213
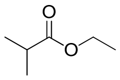	1.6963	1.6853
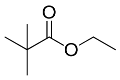	1.7021	1.6710
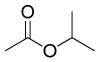	1.6624	1.6526
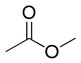	1.5652	1.5100
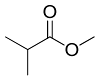	1.4632	1.5230
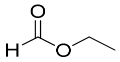	1.5145	1.4613

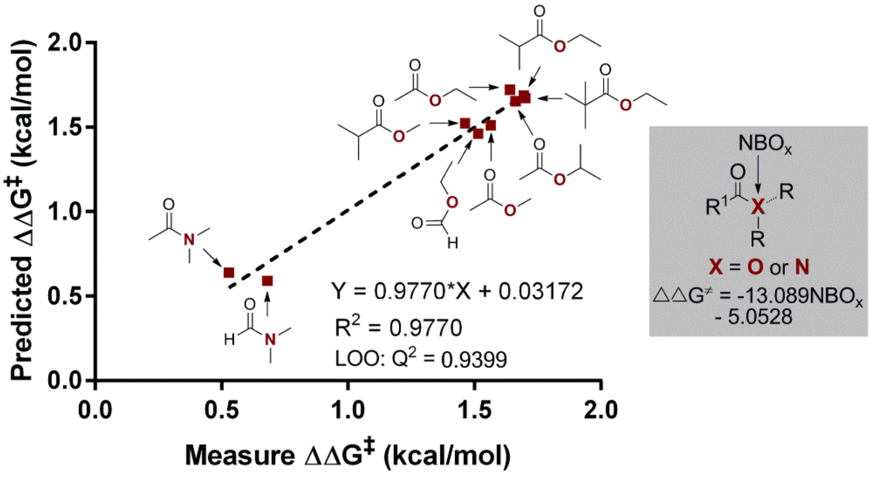

Figure S3. Comparison of predicted difference in ΔΔG‡ derived using equation S1a that related the NBO charge of the X atom of various solvents, with the measured ΔΔG‡.

Table S6. Measured and Leave-One-Out (LOO) predicted ΔΔG‡ as validation to the linear regression models obtained.

Solvent	Measured $\Delta\Delta G^{\ddagger}$ (kcal mol^{-1})	Predicted $\Delta\Delta G^{\ddagger}$ (kcal mol^{-1})
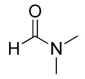	0.6814	0.4973
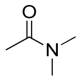	0.5290	0.7324
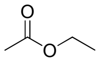	1.6407	1.7395
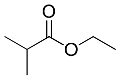	1.6963	1.6832
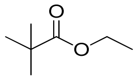	1.7021	1.6651
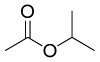	1.6624	1.6509
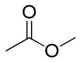	1.5652	1.5026
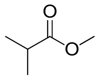	1.4632	1.5315
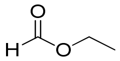	1.5145	1.4546

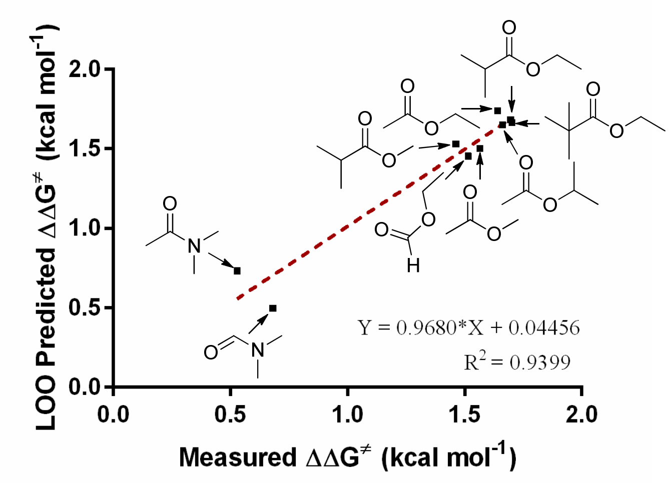

Figure S4. Leave-One-Out (LOO) validation of linear regression model based on equation S1 relating the predicted $\Delta\Delta G^{\ddagger}$ in response to the NBO_x of the solvents.

B. Effects of Ligand on Enantioselectivity

i. DFT Calculations

For computationally derived NBO charges were obtained using NBO Version 3.1 in Gaussian 09.[4] For Hammett value based on the literature.[7]

Table S7. Evaluation of Ligand Electronics[a,b]

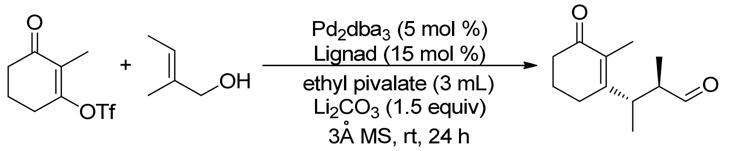

ligand		Experiment 1 yield%	Experiment 1 er	Experiment 1 dr	Experiment 2 yield%	Experiment 2 er	Experiment 2 dr
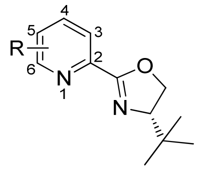	1) R = 5-CF_3	74%	5.4:94.6	> 20:1	74%	5.3:94.7	> 20:1
	2) R = 4-Cl	4%	7.4:92.6	> 20:1	6%	7.3:92.7	> 20:1
	3) R = 5-CN	13%	5.1:94.9	> 20:1	12%	5.3:94.7	> 20:1
	4) R = 5-NO_2	47%	5.3:94.7	> 20:1	49%	4.9:95.1	> 20:1
	5) R = CO_2Me	25%	7.3:92.6	> 20:1	31%	8.4:91.6	> 20:1

[a] **1a** (0.25 mmol), **2a** (0.5 mmol), $Pd_2(dba)_3$ (0.0125 mmol), ligand (0.0375 mmol), Li_2CO_3 (0.375 mmol), 3Å MS (50 mg), solvent (3 mL), Under an atmosphere of N_2 condition for 24 h. [b] Isolated yield, dr value based on NMR, er value based on SFC or HPLC.

Table S8. Summary of Ligand Screen

ligand	Trial 1 er[a]	Trial 1 Ratio[b]	Trial 1 $\Delta\Delta G^{\ddagger}$ (kcal mol^{-1})[c]	Trial 2 er[a]	Trial 2 Ratio[b]	Trial 2 $\Delta\Delta G^{\ddagger}$ (kcal mol^{-1})[c]	Average $\Delta\Delta G^{\ddagger}$ (kcal mol^{-1})[d]
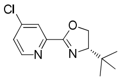	7.4:92.6	12.51	1.4969	7.3:92.7	12.70	1.5056	1.5013
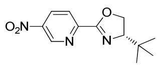	5.3:94.7	17.87	1.7080	4.9:95.1	19.41	1.7569	1.7325
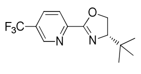	5.4:94.6	17.52	1.6963	5.3:94.7	17.87	1.7080	1.7021
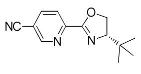	5.1:94.9	18.61	1.7320	5.3:94.7	17.87	1.7080	1.7200
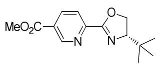	7.4:92.6	12.51	1.4969	8.4:91.6	10.90	1.4154	1.4562

[a]Enantiomeric ratios of two trial runs. [b]Enantiomeric ratios ratios of two trial runs. [c]Difference in $\Delta\Delta G^{\ddagger}$ calculated from enantiomeric ratios. [d]$\Delta\Delta G^{\ddagger}$ calculated from average enantiomeric ratios.

Table S9. Ligand NBO Values

ligand	NBO of N_1	NBO of N_2	NBO of O	Hammett Value of substituents
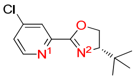	-0.37230	-0.43311	-0.49675	0.23
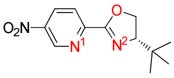	-0.36000	-0.42178	-0.49565	0.71
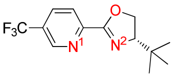	-0.36115	-0.42900	-0.49650	0.43
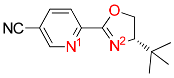	-0.36046	-0.42480	-0.49606	0.56
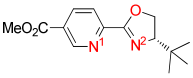	-0.37222	-0.43111	-0.49625	0.37

Equation S2. Crude linear regression model relating the NBO of N^1 atom of ligand to the $\Delta\Delta G^{\ddagger}$.

Crude Regression Model: $\Delta\Delta G^{\ddagger} = 20.44x + 9.09$

Table S10. Measured and Predicted differences in transition state energies (ΔΔG‡) derived using equation S2.

ligand	Measured $\Delta\Delta G^{\ddagger}$ (kcal mol^{-1})	Predicted $\Delta\Delta G^{\ddagger}$ (kcal mol^{-1})
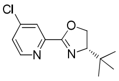	1.5013	1.4780
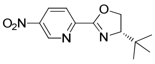	1.7325	1.7294
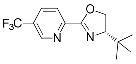	1.7021	1.7059
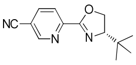	1.7200	1.7200
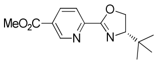	1.4562	1.4796

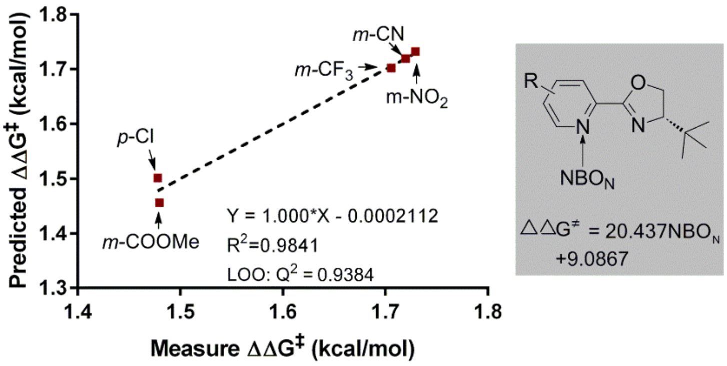

Figure S5. Comparison of predicted difference in $\Delta\Delta G^{\ddagger}$ derived using equation S2 that related the NBO charge of the X atom of various solvents, with the measured $\Delta\Delta G^{\ddagger}$.

Table S11. Measured and Leave-One-Out (LOO) predicted ΔΔG‡ as validation to the linear regression models obtained.

ligand	**Measured *ΔΔG‡* (kcal mol⁻¹)**	**Predicted *ΔΔG‡* (kcal mol⁻¹)**
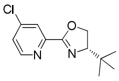	1.5013	1.4542
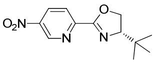	1.7325	1.7273
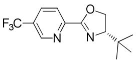	1.7021	1.7073
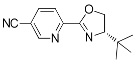	1.7200	1.7197
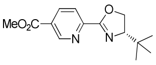	1.4562	1.5023

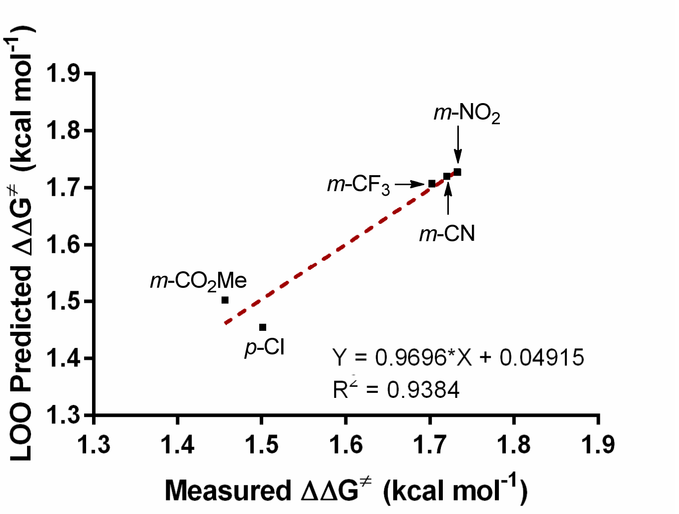

Figure S6. Leave-One-Out (LOO) validation of linear regression model based on equation S1 relating the predicted *ΔΔG‡* in response to the NBO_{N1} of the Ligands.

iv. Cartesian Coordinates of Geometry Optimized Structures

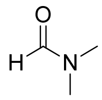

C	-2.58345	0.07050	-0.03310
H	-3.56261	0.13527	-0.45965
O	-2.04612	1.09264	0.46700
N	-1.86592	-1.21248	-0.03128
C	-2.18343	-1.94973	1.20021
H	-3.23708	-2.13028	1.24621
H	-1.66115	-2.88360	1.20153
H	-1.88317	-1.37194	2.04927
C	-2.27844	-2.00626	-1.19775
H	-2.04733	-1.46963	-2.09413
H	-1.75615	-2.94014	-1.19642
H	-3.33209	-2.18681	-1.15175

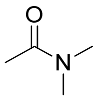

C	-1.24867	0.06128	0.08830
H	-1.02838	-0.98453	0.13988
H	-0.56565	0.60077	0.71067
H	-2.24965	0.23199	0.42564
C	-1.10809	0.54432	-1.36721
O	-1.81236	1.50042	-1.78366
N	-0.15120	-0.11148	-2.27009
C	1.15084	0.56477	-2.17913
H	1.84736	0.08742	-2.83633
H	1.04021	1.59101	-2.46108
H	1.51271	0.50812	-1.17377
C	-0.64834	-0.03365	-3.65129
H	0.04818	-0.51100	-4.30848
H	-1.59608	-0.52589	-3.71750
H	-0.75897	0.99259	-3.93324

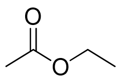

C	-3.56248	-0.25194	0.75974
H	-3.57997	-1.32013	0.81954
H	-3.83043	0.05432	-0.22985
H	-4.26049	0.15600	1.46064
C	-2.14702	0.25752	1.08920
O	-1.72492	1.32293	0.56929
O	-1.31234	-0.48010	1.98592
C	-0.43577	0.41920	2.66988

H	-1.01415	1.12911	3.22342
H	0.17394	0.93412	1.95713
C	0.46312	-0.37516	3.63559
H	1.11901	0.29775	4.14735
H	-0.14659	-0.89008	4.34833
H	1.04150	-1.08506	3.08204

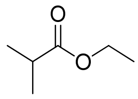

C	-3.59667	-0.08326	0.66543
C	-2.19106	0.43604	1.02064
O	-1.59470	1.22203	0.23952
O	-1.56353	0.02508	2.23810
C	-0.69467	1.06254	2.70034
H	-0.22512	0.75504	3.61131
H	-4.11034	0.65012	0.07960
C	-1.51238	2.34318	2.95115
H	-2.26111	2.14920	3.69053
H	-0.86226	3.11946	3.29702
H	-1.98193	2.65068	2.04018
H	0.05406	1.25653	1.96096
C	-4.38690	-0.35186	1.95965
H	-5.36353	-0.71267	1.71285
H	-3.87323	-1.08524	2.54548
H	-4.47299	0.55571	2.51984
C	-3.47277	-1.38947	-0.14082
H	-4.44940	-1.75028	-0.38762
H	-2.92371	-1.20284	-1.04005
H	-2.95910	-2.12285	0.44501

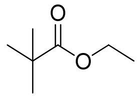

C	-3.59772	-0.08318	0.66650
C	-2.19133	0.43502	1.02024
O	-1.59468	1.21960	0.23794
O	-1.56342	0.02463	2.23770
C	-0.69318	1.06165	2.69831
H	-0.22334	0.75458	3.60928
C	-1.50940	2.34339	2.94839
H	-2.25783	2.15091	3.68846
H	-0.85824	3.11934	3.29305
H	-1.97924	2.65046	2.03743
H	0.05526	1.25412	1.95825
C	-4.38736	-0.34967	1.96151

H	-5.36453	-0.70972	1.71573
H	-3.87406	-1.08299	2.54774
H	-4.47214	0.55854	2.52085
C	-3.47570	-1.39033	-0.13853
H	-4.45287	-1.75038	-0.38431
H	-2.92706	-1.20518	-1.03831
H	-2.96240	-2.12366	0.44770
C	-4.33649	0.97225	-0.17723
H	-3.78785	1.15741	-1.07701
H	-5.31366	0.61220	-0.42301
H	-4.42127	1.88047	0.38211

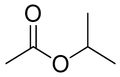

C	-3.59705	-0.08501	0.66668
H	-4.14454	-0.27281	1.56662
C	-2.19196	0.43695	1.02007
O	-1.59816	1.22418	0.23825
O	-1.56201	0.02704	2.23663
C	-0.69452	1.06611	2.69784
H	-0.22316	0.75939	3.60813
H	-4.11291	0.64746	0.08164
H	-3.50996	-0.99233	0.10626
C	0.38112	1.34752	1.63228
H	-0.09024	1.65423	0.72198
H	1.03021	2.12500	1.97738
H	0.95075	0.45884	1.45713
C	-1.51436	2.34514	2.94992
H	-2.26172	2.14962	3.69027
H	-0.86527	3.12263	3.29502
H	-1.98572	2.65185	2.03962

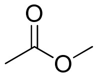

C	-3.58655	-0.16739	0.74140
H	-4.12724	-0.31476	1.65290
C	-2.18676	0.39039	1.05932
O	-1.60705	1.14310	0.23414
O	-1.54571	0.05297	2.29225
C	-0.68975	1.12585	2.69368
H	-1.27014	2.01510	2.82513
H	0.05146	1.29187	1.94005
H	-0.21008	0.87338	3.61621
H	-4.11475	0.52669	0.12161
H	-3.49023	-1.10167	0.22880

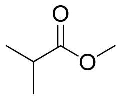

C	-3.58655	-0.16739	0.74140
H	-4.12724	-0.31476	1.65290
C	-2.18676	0.39039	1.05932
O	-1.60705	1.14310	0.23414
O	-1.54571	0.05297	2.29225
C	-0.68975	1.12585	2.69368
H	-1.27014	2.01510	2.82513
H	0.05146	1.29187	1.94005
H	-0.21008	0.87338	3.61621
C	-3.44793	-1.51205	0.00364
H	-2.91973	-2.20614	0.62344
H	-4.42051	-1.89960	-0.21725
H	-2.90723	-1.36468	-0.90786
C	-4.34676	0.83157	-0.15064
H	-5.31934	0.44403	-0.37153
H	-4.44307	1.76585	0.36196
H	-3.80607	0.97894	-1.06214

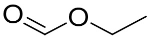

C	-1.98473	-0.07634	0.00000
H	-3.05473	-0.07634	0.00000
O	-1.35769	1.01471	0.00000
O	-1.27219	-1.31617	0.00000
C	-2.20305	-2.40170	0.00003
H	-2.81811	-2.34411	0.87369
H	-2.81814	-2.34412	-0.87362
C	-1.43502	-3.73652	0.00003
H	-0.81993	-3.79410	0.87367
H	-2.13154	-4.54878	0.00005
H	-0.81996	-3.79412	-0.87363

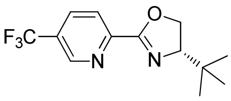

C	-5.03081	0.59050	0.00000
C	-4.2024	0.93515	-1.08363
C	-3.50909	2.14590	-1.03409
C	-3.66527	2.96445	0.08588
C	-4.51684	2.53298	1.11942
N	-5.19312	1.36801	1.08816
H	-2.85321	2.44942	-1.86239
H	-4.10771	0.26235	-1.94544
H	-4.66794	3.15071	2.02266

C	-5.17035	-2.83840	1.09668
C	-6.21223	-2.15534	1.97096
H	-5.55525	-3.82365	0.73095
H	-4.24003	-3.04569	1.68233
H	-5.78711	-1.93210	2.98181
O	-4.8697	-1.91901	-0.07822
N	-6.63483	-0.86764	1.27827
C	-7.42656	-3.07817	2.18401
C	-5.81929	-0.73234	0.00017
C	-2.92656	4.31113	0.19708
F	-2.08959	4.27919	1.25583
F	-3.82507	5.30485	0.36356
F	-2.21747	4.52989	-0.93068
C	-6.97422	-4.36500	2.89888
H	-7.80275	-4.79050	3.42555
H	-6.61288	-5.06636	2.17607
H	-6.19273	-4.13226	3.59170
C	-8.4767	-2.35379	3.04661
H	-7.98792	-1.84444	3.85069
H	-9.00546	-1.64487	2.44432
H	-9.16638	-3.06878	3.44415
C	-8.04309	-3.43853	0.81959
H	-8.38599	-2.54743	0.33662
H	-7.30422	-3.91362	0.20864
H	-8.86743	-4.10492	0.96552

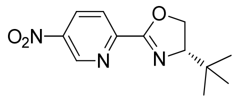

C	-1.79558	-0.05670	-0.14229
C	-2.66158	-1.07810	0.23466
C	-3.99886	-0.76851	0.42919
C	-4.41453	0.53750	0.24136
C	-3.46628	1.48381	-0.13446
N	-2.1862	1.20325	-0.32332
C	1.40409	-1.71355	-0.42481
C	1.80019	-0.25858	-0.76529
H	1.9319	-2.12825	0.43232
H	1.51352	-2.38997	-1.27238
H	2.22194	-0.19969	-1.7726
O	0.01134	-1.63003	-0.09309
N	0.52361	0.46127	-0.75525
C	2.81749	0.39513	0.19431
C	-0.35855	-0.34778	-0.36113
C	4.12907	-0.38887	0.13453

H	4.88341	0.09412	0.75845
H	4.01389	-1.41349	0.49412
H	4.51427	-0.42743	-0.88740
C	3.06814	1.82983	-0.27139
H	3.46666	1.84337	-1.28867
H	2.14314	2.40508	-0.26122
H	3.79456	2.31685	0.38248
C	2.27847	0.41386	1.62435
H	1.35855	0.99721	1.68139
H	2.07154	-0.59146	1.99853
H	3.01196	0.86866	2.29283
H	-2.28539	-2.08166	0.36820
H	-4.70207	-1.53722	0.72246
H	-3.75563	2.51772	-0.28913
N	-5.81825	0.93041	0.43136
O	-6.24713	0.50502	1.64984
O	-6.58282	0.36522	-0.54105

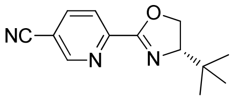

C	-2.37396	0.58699	-1.52637
C	-1.38872	0.19165	-0.61057
C	-1.26947	0.87309	0.60619
C	-2.14760	1.93412	0.87784
C	-3.11081	2.28763	-0.08090
N	-3.19302	1.61421	-1.24314
H	-0.51988	0.58417	1.31677
H	-2.47544	0.06781	-2.45811
H	-2.08680	2.47128	1.80482
C	-5.67098	4.96264	-0.07892
C	-4.59052	5.46051	0.85870
H	-5.98649	5.71223	-0.77403
H	-4.95252	6.15931	1.58410
H	-3.80865	5.92868	0.30060
C	-0.44809	-0.98692	-0.94045
N	0.28001	-1.85244	-1.13805
C	-4.08619	3.45527	0.18520
N	-4.98715	3.84010	-0.69432
O	-4.09972	4.24440	1.41675
C	-6.91615	4.47623	0.68646
C	-6.48543	3.42449	1.72748
H	-6.02124	2.59628	1.22368
H	-5.79087	3.86812	2.41226
H	-7.33878	3.08010	2.26759

C	-7.58673	5.66473	1.39434
H	-7.88686	6.38221	0.66267
H	-8.44581	5.32250	1.93401
H	-6.89606	6.11363	2.07530
C	-7.92042	3.85220	-0.30291
H	-8.20496	4.58637	-1.03826
H	-7.46319	3.01500	-0.79400
H	-8.79284	3.52251	0.23192

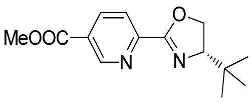

C	-2.45988	0.45741	-1.46327
C	-1.51239	0.05925	-0.51036
C	-1.40110	0.77240	0.68407
C	-2.24105	1.86670	0.89534
C	-3.17074	2.22070	-0.09192
N	-3.25530	1.51535	-1.23235
H	-0.68438	0.48522	1.42484
H	-2.55044	-0.08496	-2.38117
H	-2.17589	2.43021	1.80260
C	-5.65415	4.95816	-0.13303
C	-4.55018	5.45844	0.76966
H	-5.98174	5.70282	-0.82806
H	-4.88775	6.17606	1.48797
H	-3.77197	5.90500	0.18667
C	-4.10193	3.42665	0.13200
N	-4.99814	3.81938	-0.75064
O	-4.07244	4.24663	1.34264
C	-6.87895	4.48797	0.67344
C	-6.44295	3.41890	1.69255
H	-6.01563	2.58575	1.17470
H	-5.71633	3.83594	2.35814
H	-7.29395	3.09221	2.25288
C	-7.49396	5.68708	1.41874
H	-7.79690	6.42988	0.71066
H	-8.34496	5.36039	1.97908
H	-6.76735	6.10412	2.08433
C	-7.92473	3.88774	-0.28452
H	-8.22766	4.63054	-0.99260
H	-7.49741	3.05460	-0.80236
H	-8.77572	3.56106	0.27582
C	-0.60258	-1.15391	-0.77885
O	0.24617	-1.50635	0.08079
O	-0.72224	-1.87992	-2.00502

C	0.54769	-2.43608	-2.35556
H	0.87386	-3.09717	-1.58001
H	0.45815	-2.97931	-3.27304
H	1.26127	-1.64791	-2.47591

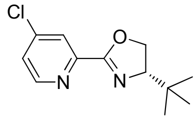

C	-1.79558	-0.05670	-0.14229
C	-2.66158	-1.07810	0.23466
C	-3.99886	-0.76851	0.42919
C	-4.41453	0.53750	0.24136
C	-3.46628	1.48381	-0.13446
N	-2.1862	1.20325	-0.32332
C	1.40409	-1.71355	-0.42481
C	1.80019	-0.25858	-0.76529
H	1.9319	-2.12825	0.43232
H	1.51352	-2.38997	-1.27238
H	2.22194	-0.19969	-1.77260
O	0.01134	-1.63003	-0.09309
N	0.52361	0.46127	-0.75525
C	2.81749	0.39513	0.19431
C	-0.35855	-0.34778	-0.36113
C	4.12907	-0.38887	0.13453
H	4.88341	0.09412	0.75845
H	4.01389	-1.41349	0.49412
H	4.51427	-0.42743	-0.88740
C	3.06814	1.82983	-0.27139
H	3.46666	1.84337	-1.28867
H	2.14314	2.40508	-0.26122
H	3.79456	2.31685	0.38248
C	2.27847	0.41386	1.62435
H	1.35855	0.99721	1.68139
H	2.07154	-0.59146	1.99853
H	3.01196	0.86866	2.29283
H	-2.28539	-2.08166	0.36820
H	-4.70207	-1.53722	0.72246
H	-3.75563	2.51772	-0.28913
N	-5.81825	0.93041	0.43136
O	-6.24713	0.50502	1.64984
O	-6.58282	0.36522	-0.54105

C. Calculations on the Stereodetermining Alkene Insertion Transition State

i. Computational Methods

Unconstrained geometry optimizations using the M06[8] functional implemented in Gaussian09[1] were used. This functional includes corrections for dispersion interactions and was found to be reliable in previous work performed on similar Heck reaction transition states[9]. The Lanl2DZ and 6-31+G(d) basis sets were used for Pd and all other atoms, respectively. Frequency calculations at the same level of theory as the optimized geometries confirmed the stationary points as transition states (one imaginary frequency) and were used to calculate the reported Gibbs free energies. Several conformations and configurations of the transition states have been investigated, but only the results for the lowest energy conformer are presented in the manuscript. The results for all of the transition states are presented in Table S12. The final free energies from the optimizations are reported in kcal/mol in the manuscript.

ii. Electronic energies, thermal corrections to energies, and free energies for transition states

Table S12. Electronic energies, thermal corrections to energies, and free energies *in vacuo* at 298 K (Hartree), calculated at the M06/6-31+G*, Lanl2DZ level of theory

Descriptive Name^	TS Name^^	E(M06)	E(correction)	$\Delta G^{\ddagger,298K}$	Imaginary Frequency
ts1_2a_r_up		-1733.4060468	0.496028	-1732.910019	-223.77
ts1_2a_s_up		-1733.4050761	0.494178	-1732.910898	-217.26
ts1_2a_r_down_a		-1733.4045786	0.490829	-1732.913750	-227.63
ts1_2a_r_down_b	TSA^R	-1733.4064095	0.491829	-1732.914580	-238.58
ts1_2a_r_down_c		-1733.4026333	0.492282	-1732.910352	-219.55
ts1_2a_s_down		-1733.4047194	0.493213	-1732.911506	-207.52
ts2_2a_r_up		-1733.4002667	0.490840	-1732.909426	-242.52
ts2_2a_s_up_a		-1733.4058688	0.493658	-1732.912211	-244.53
ts2_2a_s_up_b		-1733.4089118	0.494953	-1732.913959	-237.73
ts2_2a_s_up_c	TSA^S	-1733.4114749	0.494459	-1732.917016	-240.47
ts2_2a_s_up_d		-1733.4033392	0.492169	-1732.911170	-237.67
ts2_2a_r_down		-1733.4031478	0.492167	-1732.910981	-230.76
ts2_2a_s_down		-1733.4055537	0.495084	-1732.910470	-232.21
ts1_2aa_r_up		-1733.4028667	0.495686	-1732.907181	-231.29
ts1_2aa_s_up		-1733.4060126	0.495156	-1732.910856	-223.73
ts1_2aa_r_down		-1733.4025699	0.49525	-1732.907320	-213.04
ts1_2aa_s_down		-1733.4039985	0.4935040	-1732.910495	-224.12
ts2_2aa_r_up_a		-1733.4030154	0.494618	-1732.908397	-249.10
ts2_2aa_r_up_b		-1733.4056640	0.493790	-1732.911874	-246.89
ts2_2aa_r_up_c	TSB^R	-1733.4087506	0.495337	-1732.913414	-245.68
ts2_2aa_s_up		-1733.4022827	0.494207	-1732.908076	-247.09
ts2_2aa_r_down		-1733.4012806	0.493750	-1732.907531	-245.28
ts2_2aa_s_down_a		-1733.4044004	0.491699	-1732.912701	-249.00
ts2_2aa_s_down_b	TSB^S	-1733.4079265	0.493467	-1732.914459	-242.29

^ The descriptive name is split into sections which describe different aspects of the transition state geometry. The sections are broken down as follows utilizing this example: ts1_2a_r_up_a

ts1 indicates a structure with the alkenyl moiety *trans* to the chiral oxazoline portion of the ligand. The alternative, ts2, places the alkenol moiety *trans* to the chiral oxazoline.

2a indicates the use of (*E*)-alkenol substrate **2a**. The alternative, 2aa, indicates use of (Z)-alkenol substrate **2aa**.

r indicates a transition state leading to the *R* enantiomer product. The alternative, s, indicates a transition state leading to the *S* enantiomer product.

up indicates the alkenyl moiety is oriented with the double bond upward relative to a structure with the oxazoline portion of the ligand on the right and behind the reacting center. The alternative, down, indicates the alkenyl moiety is oriented with the double bond downward relative to a structure with the oxazoline portion of the ligand on the right and behind the reacting center

a indicates that more than one conformation of the same geometry was studied. Possible alternatives are b, c, and d.

^^ The transition state name is the designation given to the transition state as it appears in the manuscript. If a TS is not discussed in the main body of the manuscript, this field is left blank.

iii. Cartesian coordinates for complexes and transition states.

(Cartesian coordinates and imaginary frequencies of the reported geometries optimized at M06/6-31+G*, Lanl2dz)

ts1_2a_r_up

Imaginary Frequency: -223.77
Pd -0.6534727749 0.6635700473 0.0315405448
C 0.1324083351 -2.2626808943 0.4114874728
C 0.8381567275 -3.4351524078 0.6506428844
C 2.1999108786 -3.456530058 0.3633373057
C 2.7900102958 -2.3087730024 -0.1496432436
C 2.0050271311 -1.1768437685 -0.3537298601
N 0.7074974719 -1.146948289 -0.0716888656
H 2.7916076681 -4.3520901287 0.5438654798
H 0.3294050891 -4.3060326821 1.0560132101
H 2.4481695163 -0.2608415878 -0.7393932967
C 4.2494660758 -2.2817349681 -0.5235965865
F 4.4220447631 -2.778271352 -1.7552433215
F 4.7283488202 -1.035698281 -0.5153888315
F 4.9759516585 -3.0184157479 0.3163472058
C -1.3125301692 -2.1510602889 0.642098475
O -1.9329612289 -3.1977648519 1.1715809811
N -2.0083183066 -1.1133101952 0.3343657423
C -3.2943580119 -2.7566083954 1.4095104023
H -3.9573192575 -3.5790523181 1.1305522118
H -3.3874139156 -2.5622326876 2.4844600185
C -3.4265537705 -1.4944570053 0.5454610148
H -3.9324993853 -0.6912810353 1.1015232194

C -4.1857193531 -1.7119087741 -0.7866344065
C -3.5622751691 -2.8323866978 -1.6182121229
H -2.5257822958 -2.6004871355 -1.9019742545
H -3.5702922037 -3.8021769932 -1.1012086698
H -4.1297520176 -2.9627517965 -2.5492181368
C -5.639565092 -2.0545936298 -0.4540592791
H -6.1102919253 -1.2689302237 0.1552359139
H -6.223709425 -2.1464446962 -1.3794010728
H -5.744548556 -3.0075253619 0.0821440806
C -4.1701844946 -0.4206504545 -1.5975846946
H -4.7250107439 -0.5541876182 -2.5360628854
H -4.6578462453 0.3957833519 -1.0431754854
H -3.1464686209 -0.1133076543 -1.8552082088
C 3.6467570104 2.7092707328 -0.9664824656
C 1.9464443267 1.8112345928 0.7674738145
C 0.9646015387 1.9612373041 -0.1558127322
C 1.2540962784 2.3904525136 -1.5693054091
C 2.4303147901 3.3627949349 -1.5984245529
H 1.4924861485 1.4982492515 -2.1772267785
H 2.6467847945 3.6720584907 -2.6288552351
C -2.8485873734 2.1152084226 1.4265783684
H -2.3711852258 1.6720192818 2.3092415143
H -3.672951332 1.4620642386 1.1127198319
C -1.894746296 2.35142426 0.287997638
C -0.6503862441 3.0240121728 0.5236516273
C -2.5562566931 2.7237941613 -1.0215969158
H -3.4326496994 2.0879593398 -1.2055602318
H -1.8604610761 2.5950684372 -1.8701653535
H 4.4894784585 3.4008479458 -0.8437614342
C 3.3665138338 2.0843003824 0.3796030051
O 4.2685072657 1.7576962154 1.130013484
C 1.7745624834 1.2700564896 2.1502615248
H 0.724246828 1.0601462628 2.3863707507
H 2.3377252866 0.3302430337 2.2476452088
H 2.1965956194 1.9456439177 2.9060790543
H 4.0216994095 1.8985721343 -1.6168867375
H 2.1562550933 4.2743685761 -1.0418488944
H 0.3681158997 2.8339129765 -2.0433539708
H -0.3705081815 3.6947683325 -0.2941698377
H -3.2977653012 3.073667068 1.7336856349
C -0.3154390496 3.5873490732 1.8684095165
H -0.4439901445 2.884926818 2.6973151811
H 0.7039351217 3.985916451 1.9018284238
H -1.0140977472 4.4220180099 2.0375246189

O -2.9305530018 4.0819983957 -0.8714241263
H -3.4961864468 4.3570275618 -1.6069566315

ts1_2a_s_up

Imaginary Frequency: -217.26
Pd 0.6938458088 0.6435971117 -0.1064238347
C -0.1648166771 -2.2735284354 -0.5383490676
C -0.862778762 -3.4234732571 -0.8761217716
C -2.2511365844 -3.4169610279 -0.7515253676
C -2.86809664 -2.2617227038 -0.299070195
C -2.0866274272 -1.1467262029 0.0119433211
N -0.7653844566 -1.148211634 -0.0973083798
H -2.8393603731 -4.2960787196 -1.0057057657
H -0.327477246 -4.3022932306 -1.2269567166
H -2.5495053322 -0.218947482 0.3540161726
C -4.3606596202 -2.1468041393 -0.1314910531
F -4.9860932773 -3.2681448985 -0.4834801551
F -4.6673552554 -1.8776307964 1.143366521
F -4.8393160814 -1.1462468304 -0.8769815112
C 1.2990519525 -2.2009428879 -0.5904471572
O 1.972852857 -3.3046054051 -0.8870664617
N 1.966874199 -1.1407457177 -0.2995450645
C 3.3701491227 -2.9057901165 -0.9063028996
H 3.9426128173 -3.7062581016 -0.4313566464
H 3.6667949294 -2.8183991404 -1.9582604872
C 3.3777816138 -1.5659746889 -0.1560546353
H 4.0328318365 -0.8363921875 -0.6558385867
C 3.806880951 -1.6673955535 1.3321482912
C 2.954308732 -2.6757680135 2.102152501
H 1.8929343218 -2.3861954236 2.1215466806
H 3.0294305343 -3.6955065043 1.7007158858
H 3.2925480475 -2.7207856544 3.1458327846
C 5.2765341879 -2.0926106652 1.370224393
H 5.9128725802 -1.395805343 0.80508508
H 5.6373802475 -2.0985112818 2.4072790219
H 5.4430790986 -3.1027637095 0.9714437907
C 3.6867124734 -0.3032842172 2.0067871688
H 3.9767264664 -0.3815224071 3.0631486428
H 4.3524867814 0.4353513745 1.5401239398
H 2.6580553184 0.0862089011 1.9719955487
C -3.0121287284 2.8348638401 2.0092211279
C -1.9115156593 2.1450415607 -0.2131727537
C -0.7415490456 2.0244426216 0.455141402

C -0.5943085031 2.2342262166 1.9403735584
C -1.9212341685 1.9994374811 2.6582094663
H 0.186711285 1.5734061441 2.3512597079
H -2.1857606561 0.930621053 2.6163360751
C 2.2660223031 1.7480232503 -2.2563925608
H 1.3508375472 1.4122085835 -2.7753227819
H 2.9869931986 0.912111566 -2.2562981906
C 1.9538430083 2.1781144296 -0.8387189691
C 0.7728374088 2.9689412364 -0.6887764056
H 0.1547624803 3.021120662 -1.5873512721
C 0.8129574183 4.2574669516 0.0781880229
H -0.183877366 4.687821203 0.2266362363
H 1.3258997352 4.1871521071 1.0429691732
H 1.3944019293 4.957179654 -0.5435708414
O 2.8116307925 2.8976390947 -2.8736485983
H 3.0734371234 2.6995066752 -3.7840560789
C 3.1618174764 2.4748421358 0.0011198565
H 3.9336301166 1.7092941993 -0.1420270335
H 3.5990320837 3.4344937677 -0.3171411297
H 2.9353765488 2.5418651165 1.0726383126
H -2.7631600906 3.9071708405 2.1004723384
C -3.1508642986 2.523802598 0.5405978926
O -4.2207267352 2.5710478149 -0.0379756707
C -2.1159267166 1.9169174591 -1.6773997805
H -1.2457228888 1.4520087441 -2.1560401025
H -2.9885109525 1.2709491749 -1.8427269333
H -2.3547848162 2.8584751574 -2.1935427295
H -3.9930437278 2.6996395568 2.479559158
H -1.8166320152 2.2535436404 3.7207869228
H -0.2600602317 3.2615374534 2.152339594

ts1_2a_r_down_a

Imaginary Frequency: -227.63
Pd -0.6016519826 -0.6885377766 -0.1811138727
C -0.0269077928 2.2166799999 -0.7991210136
C 0.5906846836 3.3945012397 -1.1908809806
C 1.9599246989 3.5300755919 -0.9620469195
C 2.6372616527 2.4908983051 -0.3429855167
C 1.9366607969 1.3355816888 0.0105812227
N 0.6403422395 1.1958784126 -0.2229504103
H 2.486070813 4.4347831102 -1.2586345452
H 0.01184859 4.1863015794 -1.6598380625
H 2.4370726095 0.4956611475 0.4984637587

C 4.1104435247 2.5439179557 -0.0369095808
F 4.6738710869 3.6595417264 -0.4918195055
F 4.320606234 2.4710126626 1.2799321869
F 4.7387711347 1.499739464 -0.5991706814
C -1.4770007728 2.0048352856 -0.8922040486
O -2.2254887671 2.9593097687 -1.4289473516
N -2.0646466649 0.9724183997 -0.396826703
C -3.5808563893 2.4295344865 -1.4082538251
H -4.2489133733 3.2555149308 -1.1530029091
H -3.8044320444 2.0780009095 -2.4227983502
C -3.5091585307 1.300792958 -0.3730003844
H -4.0890633064 0.4231629879 -0.696335442
C -3.9878471768 1.7107090597 1.0474722836
C -3.2704419086 2.9628754233 1.5536711058
H -2.1877459485 2.8010341499 1.6612290425
H -3.4298278709 3.8396912642 0.9120431623
H -3.6525128702 3.2242784493 2.5493250009
C -5.4946270322 1.9689210301 0.978053951
H -6.0360122191 1.0922545687 0.5927187438
H -5.8847889945 2.1790747341 1.982786493
H -5.7558472322 2.831348925 0.3496256197
C -3.7394074623 0.5765994693 2.0396147532
H -4.0526001438 0.888131377 3.0451164994
H -4.3239084707 -0.317498282 1.7823746375
H -2.6764848683 0.3006938809 2.0930297031
C 3.9735544213 -2.2333161573 -0.176720175
C 3.1328507288 -2.6727394481 -1.3630003181
C 1.8429699548 -1.8577904263 -1.4216796463
C 1.0993020017 -1.8844043014 -0.1144619493
C 1.7167247446 -2.037092531 1.0810765649
H 3.6940617477 -2.5502233522 -2.2983819727
H 2.0936260912 -0.8076482322 -1.6602606718
C -2.3058988027 -2.3463341963 -1.917300111
H -1.4965705501 -2.237508881 -2.6496282358
H -3.0116083814 -1.5184062503 -2.0668761622
C -1.8136634072 -2.3776245544 -0.4961111726
C -0.6327051366 -3.106071744 -0.1582250732
C -2.9053709276 -2.4312344376 0.5439184956
H -3.686442684 -1.6867525836 0.3156439277
H -2.5060816074 -2.2161403637 1.5506141011
H 4.3611259367 -1.2126093079 -0.3465369137
C 3.2075587208 -2.1983764506 1.1244358536
O 3.7759606461 -2.2505371846 2.199799132
H 4.8573031535 -2.8647049548 -0.0252306639

H 2.8928830432 -3.7430888731 -1.2785191284
H 1.2050895035 -2.1886090603 -2.251359261
C 1.052257156 -2.0103315063 2.4204648585
H 1.2129118732 -2.95231858 2.9632122037
H 1.504140371 -1.2302978847 3.0477728969
H -0.023099764 -1.8048213364 2.3507152576
O -3.4149738851 -3.7491279617 0.4667414335
H -4.1632434519 -3.8566714717 1.071072862
H -2.8448785542 -3.280590566 -2.1433714287
C -0.0923484449 -4.1673345896 -1.0669765683
H 0.8489550154 -4.587481093 -0.6997122586
H -0.8465452199 -4.9709988656 -1.0630735483
H 0.0310498777 -3.8566865354 -2.1091969722
H -0.5665604116 -3.3710212022 0.8994015825

ts1_2a_r_down_b

Imaginary Frequency: -238.58
Pd -0.5768132898 -0.8199515411 -0.1747561113
C -0.1470177951 2.1468137218 -0.6818495159
C 0.3993255355 3.3907495485 -0.960983217
C 1.735595164 3.614400576 -0.6308046428
C 2.4526798555 2.587063717 -0.0370482881
C 1.8279446602 1.3591020887 0.1888575456
N 0.5611549841 1.1402211405 -0.1331048139
H 2.207424026 4.5751437626 -0.8266452098
H -0.2127033029 4.1657204536 -1.4156828975
H 2.3698673982 0.5271642132 0.644452383
C 3.8912258724 2.7333999095 0.3821261835
F 4.0042198804 2.6621942948 1.7107257939
F 4.6288253855 1.7405240702 -0.1353165756
F 4.4105234662 3.8905141933 -0.0219031397
C -1.5708414623 1.8481317515 -0.8735351728
O -2.3260673087 2.7625213536 -1.4712831166
N -2.1383747099 0.7863444913 -0.4177496184
C -3.6403814073 2.1520065533 -1.5710499626
H -4.3806981975 2.9368879358 -1.3985423593
H -3.7422261462 1.7702408239 -2.5944281456
C -3.5972141325 1.0452504837 -0.512748437
H -4.1128947177 0.1389533761 -0.8631758536
C -4.2055948286 1.4602807437 0.8549412128
C -3.5708939731 2.7443888067 1.3914894138
H -2.5001810942 2.6127766415 1.6057276325
H -3.6853481519 3.5962509397 0.7072339935

H -4.0542114605 3.0246582906 2.3365221658
C -5.7090340834 1.6713655858 0.6592096425
H -6.1910209952 0.7775809967 0.236086949
H -6.1845845344 1.871561512 1.6286459862
H -5.9448057655 2.5241228898 0.0084933313
C -4.0077256051 0.3496116311 1.8838873967
H -4.3927325715 0.6769152826 2.8593521699
H -4.5713324978 -0.5532818624 1.6051987606
H -2.9501842463 0.0823834567 2.0134045931
C 4.0795410056 -1.9393274139 -0.6405341001
C 3.159930854 -2.4301805687 -1.7446799237
C 1.8053942897 -1.7351968811 -1.6351347116
C 1.213175102 -1.8723897489 -0.2592789179
C 1.9638207259 -1.9983587591 0.8593275109
H 3.6032853705 -2.2356637315 -2.730022083
H 1.9351988115 -0.6578803467 -1.8464197342
C -2.2997014966 -2.6462334651 -1.7773900402
H -1.5747440658 -2.4190344725 -2.5677202732
H -3.1280242348 -1.9313278368 -1.8666727017
C -1.6905348534 -2.604422801 -0.4021803662
C -0.4179944102 -3.2060720311 -0.1605107206
C -2.7179119689 -2.67136626 0.6987741666
H -3.2338424554 -3.6473099268 0.5999564655
H -3.479921154 -1.8914042895 0.5335676437
H 4.3486529963 -0.8826544665 -0.8180630513
C 3.459178154 -1.9965016903 0.7361756453
O 4.1467386353 -1.980892354 1.7411146679
H 5.0296793732 -2.4857452057 -0.6014970164
H 3.0263201875 -3.519697499 -1.6691448296
H 1.1130417048 -2.0926772867 -2.4083980709
C 1.449470285 -2.0594303032 2.2619345115
H 1.7826755061 -2.9785002138 2.7637435484
H 1.8798971021 -1.2373126878 2.8508201972
H 0.3580761486 -1.9869422369 2.3179621767
O -2.1090019249 -2.5437377169 1.9575079805
H -2.7401234591 -2.7662066241 2.6555802945
H -2.7113706076 -3.6478751923 -1.9871883623
C 0.1407992983 -4.2142819691 -1.1234222601
H 1.1485222822 -4.5347305862 -0.8417697076
H -0.5152156302 -5.097807924 -1.0705950917
H 0.1454944104 -3.8925613837 -2.1698777325
H -0.2480189332 -3.4536919592 0.8875578096

ts1_2a_r_down_c

Imaginary Frequency: -219.55
Pd 0.6049634994 0.7254882983 -0.1461592083
C -0.0587478261 -2.188431041 -0.6712121939
C -0.6947340033 -3.3898169487 -0.9445241567
C -2.0433300735 -3.5138350275 -0.6113607897
C -2.6844158647 -2.4350986383 -0.022878377
C -1.9731758839 -1.251587096 0.1902696251
N -0.6936107407 -1.1275287677 -0.1328235207
H -2.5790423562 -4.4415565997 -0.8002328975
H -0.1424886302 -4.211382208 -1.3937736036
H -2.4536492313 -0.3790027814 0.6374739071
C -4.1246993477 -2.4820728046 0.4159802823
F -4.7176870865 -3.6168022224 0.0526516854
F -4.2107715644 -2.3685153626 1.7448660283
F -4.8108568603 -1.4624947694 -0.1166251491
C 1.3863939554 -1.9997002117 -0.8483361329
O 2.0764212608 -2.9573763158 -1.4548425652
N 2.0280484821 -0.9903972386 -0.3708774281
C 3.4342042152 -2.4437068915 -1.5360931149
H 4.1124091881 -3.2838016307 -1.371727291
H 3.5707588808 -2.0545188046 -2.5525027627
C 3.4647182174 -1.3535873688 -0.4605735216
H 4.0450688222 -0.4803834092 -0.7924774648
C 4.031507206 -1.8309451611 0.9039983584
C 3.3198495403 -3.0879291727 1.4077663247
H 2.2513408816 -2.9053151867 1.5949706589
H 3.4097082583 -3.9378702456 0.7175151632
H 3.7618039215 -3.4027126213 2.3622724429
C 5.5229918454 -2.11964064 0.7178716234
H 6.0607835542 -1.2365489678 0.342456938
H 5.973565896 -2.3929871075 1.6812485704
H 5.7181736786 -2.9528672406 0.0293003989
C 3.8833110383 -0.7329393296 1.9541521458
H 4.2643212652 -1.0891528596 2.9206550431
H 4.4682396455 0.1579213999 1.6858523752
H 2.8346744914 -0.4377304607 2.097700215
C -3.8140102115 2.6473338166 -0.7166082008
C -3.1216543291 1.7272668055 -1.7078974223
C -1.6084740701 1.9380326893 -1.6660511465
C -1.0788886593 1.9360154654 -0.2557637319
C -1.8433932247 2.1176251822 0.8451056649
H -1.1027263536 1.1612500083 -2.262054702
C 2.4355911261 2.4640085783 -1.7223566846

H 1.669940431 2.3692060411 -2.5027307874
H 3.1740947915 1.6661330139 -1.8746171737
C 1.861151013 2.4112967365 -0.3329809706
C 0.6724930769 3.1372383965 -0.0254040428
C 2.8835289973 2.3918341872 0.7771213869
H 3.6933868576 1.6852141944 0.5355095072
H 2.4283691063 2.0821853833 1.734808748
C -3.3009090347 2.4311091104 0.6843960559
O -4.014531099 2.5253342356 1.6654494542
H -1.3697774024 2.8906105832 -2.163178697
C -1.3681842429 2.0315346103 2.2610042318
H -1.4967382981 2.9894811546 2.7847014561
H -1.9796285058 1.3104860943 2.8205943049
H -0.320487746 1.7129119251 2.3306992192
O 3.3683542465 3.7201768547 0.8461208259
H 4.0878368783 3.7821234053 1.4904809084
H 2.9578850958 3.4226158301 -1.8730821972
C 0.221579264 4.2816129783 -0.8810497598
H -0.7937326957 4.6174160406 -0.6424954967
H 0.9150376517 5.1042689662 -0.6414077146
H 0.3076191802 4.0989582999 -1.9563136649
H 0.5233010731 3.3175444235 1.0417794892
H -3.3633893727 0.6784829218 -1.4734139985
H -3.4888520854 1.9068307467 -2.7265498352
H -3.6197116058 3.7009620528 -0.9847136977
H -4.9023441273 2.5191957009 -0.705893937

ts1_2a_s_down

Imaginary Frequency: -207.52
Pd -0.6611493541 -0.7204167402 -0.1334053542
C 0.1405740418 2.1569679986 -0.7204465601
C 0.8281689419 3.3031685291 -1.0894789043
C 2.2039891011 3.3506749944 -0.8636881468
C 2.8161469609 2.2585761453 -0.2694778964
C 2.0472812584 1.1386908568 0.0553465586
N 0.7448677065 1.0794475919 -0.1786583812
H 2.7831563944 4.2295645659 -1.1387918145
H 0.2957916469 4.1410085121 -1.5329392036
H 2.5041566482 0.2641007934 0.5214737009
C 4.2838645194 2.2234331207 0.0641487936
F 4.9404621689 3.2511634333 -0.4681722934
F 4.4638458185 2.2486976804 1.3872132195
F 4.8379188827 1.0904288667 -0.3914703084

C -1.3218313455 2.0507015997 -0.7878602814
O -2.0104038236 3.0677163295 -1.2893256484
N -1.973998192 1.0556026291 -0.2960783018
C -3.399607405 2.6367681985 -1.2483808719
H -4.0019168185 3.5037906197 -0.9674255625
H -3.667345759 2.3184450336 -2.2633570509
C -3.3887401213 1.4893433933 -0.2321399723
H -4.0391398041 0.663040033 -0.5558692481
C -3.8020306027 1.9066815682 1.2056415373
C -3.0125109064 3.1200651496 1.7000826177
H -1.9345840049 2.9111064967 1.7671841484
H -3.156134361 4.0128316664 1.0766867242
H -3.3464200266 3.383443948 2.712368271
C -5.2964503458 2.2356028447 1.1843634632
H -5.8918826343 1.3796675515 0.8341017007
H -5.6399773637 2.480238862 2.1982292475
H -5.5390641308 3.0995882249 0.5500069804
C -3.5729787563 0.7526575705 2.1791293222
H -3.8598917881 1.0605492809 3.193535826
H -4.1853972071 -0.1216639879 1.921596986
H -2.5173916458 0.4455955158 2.2099708416
C 3.7569256408 -2.7020674644 -0.2596671039
C 3.1099771393 -1.9993523037 -1.4426236128
C 1.6188438252 -2.3112216624 -1.4817189424
C 0.9493108609 -2.061595954 -0.1561008426
C 1.6051822144 -2.0942063579 1.0268769007
H 3.2735023496 -0.9128882449 -1.3739795698
H 1.1160223179 -1.7364969625 -2.275680282
C -2.5824834784 -2.0993771252 -1.791973648
H -1.7637754939 -1.9164816921 -2.5114625273
H -3.258070005 -1.2257189766 -1.8148042112
C -2.0316584422 -2.2965752824 -0.3963036752
C -0.8461325403 -3.0952044045 -0.3081286833
H -0.4969542944 -3.4209326964 -1.29071984
C -0.6858249512 -4.128802843 0.7658283866
H 0.2905302222 -4.6225550851 0.7086646862
H -0.8142906649 -3.7328169228 1.7783957587
H -1.4664960015 -4.8891789657 0.608239203
C -3.0757551856 -2.3924867736 0.6762927737
H -3.8606732056 -1.6431384949 0.5234194412
H -3.5615570023 -3.3793857478 0.6191451706
H -2.6710143906 -2.272705486 1.6883009737
O -3.2742643262 -3.2930212278 -2.097380173
H -3.6949794439 -3.2263579469 -2.9663782443

H 4.8239007987 -2.4706006771 -0.1583487998
C 3.080453266 -2.3614279336 1.0444197968
O 3.6911978136 -2.2958379942 2.0956323446
H 3.6810694175 -3.7960006739 -0.3913720919
H 3.5777039961 -2.3198420581 -2.3825654578
H 1.4978699004 -3.3749245371 -1.7570948905
C 1.0052670724 -1.8110523629 2.3675584148
H 1.2191209612 -2.6220155612 3.076562523
H 1.459991329 -0.9100924778 2.8047358095
H -0.0777283922 -1.641399979 2.310497274

ts2_2a_r_up

Imaginary Frequency: -242.52
Pd 0.1877974513 -0.6338130009 -0.2512590799
C -1.6043469203 1.6133863929 0.6485031059
C -2.6654664039 2.3593604314 1.1369237847
C -3.9629347224 1.9245121105 0.8712907529
C -4.1316885886 0.7724837632 0.1198422468
C -3.0061053977 0.0741299131 -0.321892466
N -1.7670312615 0.4673072077 -0.0529493272
H -4.8233940537 2.4808653135 1.2364312044
H -2.4738400446 3.2664584497 1.7050190956
H -3.1201890613 -0.8339902178 -0.9141174469
C -5.4861827156 0.2176901736 -0.2318503099
F -6.4720734075 0.9432164225 0.2865972738
F -5.649025787 0.1712410393 -1.5582050675
F -5.6038208123 -1.0377210293 0.2260677715
C -0.2096727712 2.0531935683 0.7778471898
O 0.0761955885 3.0962723401 1.5481929268
N 0.7424479758 1.500425521 0.1177523683
C 1.5330590104 3.1738738081 1.5350870943
H 1.8051060498 4.2318579991 1.5181873255
H 1.8843351583 2.7221545281 2.4710550749
C 1.9246364821 2.3667076481 0.2906949632
H 2.8052819045 1.733851296 0.4860012464
C 2.1912242736 3.2210809863 -0.9779998777
C 1.088320757 4.2533753602 -1.215692134
H 0.1064278305 3.7790156432 -1.3598493527
H 1.0011068534 4.9892197036 -0.4050284544
H 1.3076388145 4.8161945314 -2.1327898748
C 3.5337998445 3.92719212 -0.7853549332
H 4.3518793564 3.2037136554 -0.652508095
H 3.7718676903 4.5382685563 -1.6661189688

H 3.537625195 4.6007535529 0.0831630868
C 2.2730536908 2.3218558554 -2.2103256048
H 2.5341371309 2.9215803314 -3.0926897896
H 3.048204888 1.552356123 -2.1007226879
H 1.316357115 1.8227340716 -2.4169184233
C 5.0133883928 -1.4039194602 -0.1703264177
C 4.2897054755 -0.7629390382 -1.3425126074
C 2.8564183704 -1.2848610655 -1.4597327707
C 2.155384626 -1.3373101035 -0.1294725604
C 2.7830049217 -1.1841819263 1.0614333342
H 4.2822041729 0.3280598269 -1.1901890287
H 2.2794776063 -0.6527374778 -2.1542956613
C 1.6206784511 -3.9834578893 -0.6846975062
H 2.6660768734 -4.0291803742 -0.3601023881
H 1.5783255321 -3.8897328272 -1.774076701
C 0.8303723154 -2.9485454937 0.0579217565
H 0.9512040871 -3.0282796062 1.1405050612
C -0.5025212306 -2.6157252891 -0.3428852884
C -1.536089596 -2.8309854459 0.7432295277
H -2.5114489611 -2.4044869617 0.4557165972
H -1.2189178832 -2.3544675337 1.6882259034
H 1.1440721226 -4.9434092315 -0.42703923
H 6.0469023957 -1.0540790975 -0.0690576444
O -1.6227031763 -4.2356613559 0.8846608407
H -2.3621759086 -4.472748125 1.4625767044
C -0.964070482 -2.9289915287 -1.7393808771
H -1.1981923523 -4.0027026688 -1.8222521287
H -1.8827498505 -2.3836356961 -1.9943127914
H -0.2106190154 -2.6846095194 -2.4989445032
C 4.2768320326 -1.0954513071 1.1061045789
O 4.8408679332 -0.8057650765 2.1454259507
H 5.0521655213 -2.4996269075 -0.2952509165
H 4.8244283712 -0.9424613341 -2.2841186771
H 2.8777216776 -2.2808368301 -1.9248478394
C 2.1150694346 -1.0911966575 2.3959406369
H 2.3604338193 -0.1278392262 2.8658832113
H 1.022583069 -1.1601081769 2.3239929873
H 2.4900901409 -1.8561847648 3.0898908291

ts2_2a_s_up_a

Imaginary Frequency: -244.53
Pd 0.1325370518 -0.6520258278 -0.0650627797
C -1.6371957018 1.7473431337 0.4792095895

C -2.6988416126 2.5852906085 0.7849843358
C -3.995617638 2.1280968576 0.5611427744
C -4.1637798695 0.8571003993 0.0351216994
C -3.0396655551 0.0726092745 -0.2258865339
N -1.8003873255 0.4939310132 -0.0009249241
H -4.855581867 2.7538646558 0.7892487901
H -2.5077442376 3.5775887612 1.1858472301
H -3.1571205541 -0.932638067 -0.6313505229
C -5.5175250772 0.2641537747 -0.2517039512
F -6.5020487923 1.1173713246 0.0127765078
F -5.6080699304 -0.0982118805 -1.5369433971
F -5.707833683 -0.8391780851 0.4846670044
C -0.241582647 2.1820043282 0.6094559904
O 0.0062951861 3.3266308731 1.2354630502
N 0.7429745919 1.5209448204 0.1197462311
C 1.4565907107 3.3879912314 1.3410999901
H 1.755515314 4.4220179587 1.1520065059
H 1.7134917908 3.1174416101 2.3728899445
C 1.9454181922 2.3667270673 0.3037118346
H 2.7604449988 1.7478796591 0.7080639151
C 2.439461427 2.9868796443 -1.024998152
C 1.3942039034 3.9117630046 -1.645529578
H 0.4639531059 3.37484944 -1.8815441217
H 1.1449624905 4.7667410744 -1.0015524123
H 1.7769108651 4.3243184466 -2.5886463582
C 3.7233674959 3.7638347335 -0.7270056056
H 4.4904151151 3.1144664641 -0.2793878029
H 4.1373337859 4.1761010546 -1.6569110206
H 3.5610721242 4.6123051609 -0.0480375346
C 2.766363439 1.8702632409 -2.0111889882
H 3.1795846746 2.2898111265 -2.938339677
H 3.520816841 1.1866709065 -1.592253962
H 1.8721598439 1.2876490604 -2.2744370571
C 4.9319813293 -1.3577667482 -0.6012571247
C 4.0222477779 -2.3337722718 -1.328919656
C 2.6305666387 -1.7201741658 -1.4694872683
C 2.0748739095 -1.406157989 -0.1060293484
C 2.86294769 -0.9802141761 0.9098143042
H 4.4334462149 -2.5812575969 -2.3158417586
H 2.6926662342 -0.7942742815 -2.0623214786
C 0.7802244172 -2.9435959216 0.3686078034
C -0.5927260113 -2.6207690484 0.1143050345
C -1.5861228195 -2.7129012288 1.2419483563
H -2.5621475734 -2.2970593937 0.9638784854

H -1.2460600078 -2.2065263006 2.1535235616
H 5.2108294292 -0.5238457345 -1.2675500608
C -1.1148917932 -3.0528033459 -1.2378999144
H -2.0434770635 -2.5128336271 -1.4928832624
H -0.3749342621 -2.8353863959 -2.0283434044
C 4.3100224951 -0.7350814609 0.6270400778
O 4.9569087242 -0.0077414983 1.3623973387
H 5.8828134237 -1.8118249143 -0.2923473832
H 3.9473983482 -3.2806694981 -0.768888701
H 1.9622665282 -2.3838848323 -2.0344847332
C 1.2198050873 -3.473605022 1.6972845037
H 0.7479264567 -4.4618275566 1.8170863938
H 2.3057558816 -3.6075260754 1.7410815454
H 0.9097606835 -2.8628919792 2.5502575047
O -1.3498949548 -4.4412832421 -1.1236277697
H -1.7527320044 -4.7834724643 -1.9347101955
H -1.756924261 -3.7725827786 1.4886906622
H 1.263403994 -3.4475557693 -0.4729719966
C 2.4136836035 -0.6097442697 2.2873154362
H 2.7460657236 0.4079407329 2.5324022671
H 1.3228980129 -0.6529271086 2.3947355115
H 2.8777906912 -1.2578098859 3.044696255

ts2_2a_s_up_b

Imaginary Frequency: -237.73
Pd -0.1421011775 -0.6671757142 0.0938309177
C 1.6242480621 1.7430115188 -0.475356458
C 2.6804095254 2.5828401536 -0.7967170487
C 3.9807419524 2.1334379118 -0.5796665784
C 4.1580250394 0.8676388117 -0.0441031414
C 3.0384031192 0.0823868443 0.2324413306
N 1.795761184 0.494978907 0.0144174344
H 4.8362350632 2.7609154035 -0.8197709728
H 2.4810096986 3.570763189 -1.2043004513
H 3.1642116028 -0.9183210348 0.6474788577
C 5.5156077328 0.283868969 0.2405281064
F 6.4960917951 1.1335519551 -0.0490874202
F 5.620143019 -0.0551905234 1.5312469311
F 5.7028423183 -0.8336138862 -0.476682103
C 0.2268508596 2.1732509734 -0.5998246305
O -0.0243934298 3.3116008096 -1.2376302681
N -0.753051407 1.5174979595 -0.0958520456
C -1.4748866284 3.3746386986 -1.3318595182

H -1.7705282617 4.4099076599 -1.1438996277
H -1.7412301364 3.1006677287 -2.3602224375
C -1.9571067479 2.3600173732 -0.284560589
H -2.7724636949 1.7365904302 -0.6815224925
C -2.4483772415 2.9929229979 1.0393004597
C -1.4025362129 3.9259446095 1.6466608706
H -0.4717126256 3.3923000682 1.8874617781
H -1.1548195306 4.7735896019 0.9923612669
H -1.7841289813 4.3490700581 2.5855706565
C -3.7345099593 3.764498452 0.7367990972
H -4.4987806188 3.1103377329 0.2910067178
H -4.1507915184 4.1785858212 1.6648915012
H -3.5755037861 4.6112856713 0.0546630162
C -2.7708729801 1.8870120018 2.0384058118
H -3.1806181361 2.3162118149 2.9627294124
H -3.5270580451 1.1995833937 1.6294419483
H -1.8766774341 1.3059889547 2.3043873
C -4.9595868108 -1.3052521132 0.5286345992
C -4.0861336761 -2.2916392459 1.2847683468
C -2.6861311483 -1.7053148906 1.4555549346
C -2.0936100808 -1.4151749023 0.1032289319
C -2.8480082633 -0.9804596427 -0.9353458997
H -4.5248122651 -2.5222200838 2.2640581974
H -2.7477710456 -0.7697127277 2.0327601662
C -0.7981153509 -2.9367138368 -0.3093036688
C 0.5884197425 -2.6413772088 -0.0817966611
C 1.5610435649 -2.7249735746 -1.229932027
H 2.5502173971 -2.3323565005 -0.9616991856
H 1.2150483857 -2.185310079 -2.1192925329
H -5.2351649706 -0.4596879502 1.1817974478
C 1.1984773303 -3.0524913886 1.2380350259
H 1.6286203439 -4.0642513293 1.1007059058
H 2.0458904984 -2.3872389486 1.485502666
C -4.2958690453 -0.7049452631 -0.6887371548
O -4.9123118477 0.0271855316 -1.4463798234
H -5.9127638986 -1.7404167285 0.1999964339
H -4.0182695868 -3.2453678657 0.7352850484
H -2.0371229938 -2.3657122343 2.0418518281
C -1.2460196953 -3.4916057158 -1.6305506213
H -0.7872455293 -4.4859002467 -1.7468421936
H -2.3336893086 -3.6148238895 -1.6639046399
H -0.9429450878 -2.8952635414 -2.4959586919
O 0.2356930487 -3.0399596598 2.259079929
H 0.607416 -3.416285891 3.0686794713

H 1.7081187133 -3.77712964 -1.5247641505
H -1.2630727596 -3.4243189482 0.5496741411
C -2.3647362378 -0.624234106 -2.3055835625
H -2.671407562 0.4001727558 -2.5567158577
H -1.2735351803 -0.6886094822 -2.3941962728
H -2.8302494587 -1.261408279 -3.0713400114

ts2_2a_s_up_c

Imaginary Frequency: -240.47
Pd -0.1210981896 -0.6517695905 0.0833541142
C 1.6188450735 1.756437803 -0.5234610568
C 2.6650789123 2.5983122656 -0.8667051043
C 3.9697570373 2.1612697138 -0.6471910563
C 4.1562144936 0.9098757323 -0.0836312366
C 3.047362702 0.1166608691 0.2206326642
N 1.8007888054 0.5192798798 -0.0086575161
H 4.8198760285 2.7891422191 -0.9046406021
H 2.4570217181 3.5773636923 -1.2908707402
H 3.1635716778 -0.8670051488 0.6759930048
C 5.520367723 0.3426621476 0.202467508
F 6.4922551083 1.1991214774 -0.1038063394
F 5.6384263736 0.0219287893 1.4963908403
F 5.7164534535 -0.780568541 -0.5025455981
C 0.2185639477 2.1767327417 -0.6393718063
O -0.0489212914 3.3137082799 -1.2721860067
N -0.7535103807 1.5152046101 -0.1250145018
C -1.5001503324 3.3706652765 -1.3454329282
H -1.7976802324 4.4045895112 -1.1527607761
H -1.7805214075 3.09665061 -2.3700795821
C -1.9632234657 2.3530974134 -0.2926337295
H -2.7831648077 1.7294028356 -0.678315073
C -2.4377169232 2.9802570115 1.0399315341
C -1.3855917134 3.912294794 1.6373312061
H -0.4507128145 3.3790707965 1.8626250892
H -1.1483096583 4.7621947968 0.9821784321
H -1.7549133077 4.331750266 2.5827602822
C -3.7295118412 3.7501079028 0.7575395206
H -4.4992377625 3.0951086647 0.3224222739
H -4.133031101 4.1635287446 1.6915504025
H -3.581038212 4.5966929854 0.0731800967
C -2.7458898861 1.8692938534 2.0385450014
H -3.1490319864 2.2936562569 2.9680017434
H -3.5031941798 1.1799105939 1.6340183895

H -1.845866246 1.2924464435 2.2945219815
C -4.9286010367 -1.3382741307 0.5813167291
C -4.0296472647 -2.3238105495 1.308526165
C -2.6342269239 -1.7208897169 1.4549716439
C -2.067385555 -1.4065576903 0.0958676705
C -2.8501077724 -0.9693786444 -0.9207454319
H -4.4456503388 -2.5716323835 2.2933920268
H -2.6906490957 -0.7964932391 2.0503250135
C -0.7795872253 -2.9086841239 -0.3787285389
C 0.5929734502 -2.6388799958 -0.050611273
C 1.6416524119 -2.7292265401 -1.1283520643
H 2.6174180564 -2.3849950326 -0.7716931589
H 1.3794467691 -2.1584151111 -2.0270923394
H -5.1982247778 -0.5021660154 1.2486494108
C 1.0327930671 -3.1160000701 1.3130639806
H 0.21527407 -3.0026184098 2.0453955409
H 1.253450311 -4.1983035134 1.2298870243
C -4.2980348138 -0.7212028087 -0.6453275113
O -4.9416490878 0.0033188007 -1.3865145512
H -5.8839674099 -1.7815056047 0.270974333
H -3.9612347417 -3.2692264431 0.7446514027
H -1.9749951593 -2.3910274946 2.0229634401
C -1.1580085679 -3.4088374929 -1.7412401767
H -0.7135703555 -4.4089436786 -1.8631687254
H -2.2436932552 -3.5069982078 -1.8449724753
H -0.7862993377 -2.7911451047 -2.5637999791
O 2.1797550285 -2.4113218572 1.7285591955
H 2.5666351001 -2.838764634 2.5052905475
H 1.7678221678 -3.7821192572 -1.4327658969
H -1.3123004226 -3.4356797216 0.419639393
C -2.394253516 -0.5886311562 -2.293600162
H -2.7381215013 0.4255864679 -2.5356398347
H -1.3026590494 -0.6167411255 -2.3928193242
H -2.8448358589 -1.2374523928 -3.058380965

ts2_2a_s_up_d

Imaginary Frequency: -237.67
Pd -0.1745706212 -0.6457566423 0.2493033703
C 1.5255219387 1.7045640086 -0.596258323
C 2.5396108638 2.4988553788 -1.1085977792
C 3.8559164912 2.0561902566 -0.9941279283
C 4.0901593031 0.8431127609 -0.3673336855
C 3.0087413749 0.1006426294 0.109599308

N 1.7501699159 0.5079117095 -0.0043120443
H 4.6814390253 2.6479529754 -1.3834376865
H 2.2978788799 3.4460589334 -1.5844685439
H 3.1786151315 -0.8581598619 0.5998203822
C 5.4719881341 0.2779412395 -0.1752200114
F 6.4022923355 1.0299436047 -0.7556580933
F 5.7619190503 0.1819532665 1.1277771754
F 5.5469679897 -0.9565450897 -0.6899077843
C 0.1208349689 2.1297338297 -0.6060935259
O -0.2091368129 3.251202939 -1.2354814094
N -0.7981223907 1.481007056 0.010626345
C -1.6645751365 3.3038283395 -1.1615517436
H -1.9451992678 4.3449059757 -0.9834850618
H -2.0467927904 2.9818386253 -2.1382149314
C -2.0062723514 2.3262278496 -0.0280192365
H -2.871457771 1.7002035161 -0.2966445975
C -2.2817464156 2.9880556777 1.3472851273
C -1.1885046472 3.9875101549 1.7295046465
H -0.2060989417 3.5043396735 1.8337353463
H -1.0922754197 4.8163124793 1.015096032
H -1.4266219694 4.4348079916 2.7037736368
C -3.6292820485 3.7059216618 1.2579013733
H -4.4433220863 3.0085878568 1.0111536051
H -3.8742635181 4.1719076706 2.2215834575
H -3.6343146893 4.5066711339 0.5049526416
C -2.3562058805 1.9190052608 2.4365968442
H -2.5932615408 2.3871301265 3.4015974859
H -3.1442507782 1.1813816676 2.2316662803
H -1.4024078251 1.3849581766 2.5493352784
C -4.9641873673 -1.3450013648 -0.4663833454
C -4.4227943295 -0.875408606 0.87412737
C -3.065799302 -1.5165878499 1.145211305
C -2.128032752 -1.4006933967 -0.0256780376
C -2.5448041632 -1.1470159114 -1.289586945
H -2.5969837702 -1.0895982738 2.0464113826
C -0.8133563377 -2.950721348 0.2593455547
C 0.5725397051 -2.6042922003 0.375960553
C 1.5218646832 -2.9657978606 -0.7325138142
H 2.5101648886 -2.5112349122 -0.5931564167
H 1.1518619665 -2.6813496211 -1.7244766749
C 1.1377349648 -2.7296923866 1.7739001548
H 2.0928171858 -2.1829004189 1.8633473454
H 0.4352294757 -2.304912517 2.513965071
C -4.006214154 -0.9755893615 -1.5669506962

O -4.372944142 -0.5561874729 -2.6499894402
H -3.2356764831 -2.5841910212 1.3744722738
C -1.2862074042 -3.8708278521 -0.8288766702
H -0.8016017183 -4.8457796184 -0.661233476
H -2.3718452202 -4.0172879689 -0.793620042
H -1.0130101018 -3.5365926057 -1.8350929499
O 1.3247429772 -4.1151654069 1.9789111087
H 1.7491242459 -4.2757451293 2.834075077
H 1.6793666507 -4.0560214913 -0.7324999738
H -1.2655006379 -3.1535242974 1.2334989306
C -1.6701193989 -0.9650126152 -2.4895849417
H -1.7409995132 0.072267116 -2.8500347438
H -0.6130447822 -1.1719455143 -2.2777195231
H -2.0096599914 -1.5899195913 -3.3265271734
H -4.3318204653 0.2224032663 0.8598301757
H -5.1178495501 -1.1214087498 1.6873842041
H -5.0746545468 -2.4438476013 -0.4619459706
H -5.946821113 -0.9218862492 -0.7027966222

ts2_2a_r_down

Imaginary Frequency: -230.76
Pd -0.1847620296 -0.5899650971 -0.0940711576
C 1.7320722932 1.7346697052 -0.5074791319
C 2.8545380821 2.5186088007 -0.7300428581
C 4.1031010407 2.0100541392 -0.3794985676
C 4.1622875023 0.7488555767 0.1909415719
C 2.9829774846 0.0256524713 0.3731930605
N 1.7905913689 0.4909521544 0.0176838873
H 5.0105538071 2.5875201073 -0.5421013002
H 2.7442116451 3.5090732314 -1.1644513897
H 3.0155848142 -0.9660868351 0.8233062806
C 5.4577964218 0.0983162305 0.5962042117
F 6.5011225769 0.8958184574 0.3877765531
F 5.4355591029 -0.2397172873 1.8893633479
F 5.6455381585 -1.0295577537 -0.1056091574
C 0.3773453678 2.2304788559 -0.7708214924
O 0.2490678578 3.4118498555 -1.3651939265
N -0.6824111397 1.6016445318 -0.4124740016
C -1.1777935034 3.5665749304 -1.5925729371
H -1.4333622641 4.605700697 -1.3709756987
H -1.3570409023 3.3663504794 -2.6556976197
C -1.8129479661 2.5253753112 -0.6590617227
H -2.611641867 1.9737129841 -1.1754571379

C -2.3955721193 3.1073670787 0.6525860334
C -1.3758909227 3.9809817277 1.3837612716
H -0.4806417903 3.4113445107 1.6729122801
H -1.0561622432 4.8513006916 0.7939990193
H -1.8199308116 4.3695641453 2.3098402898
C -3.628723308 3.9365085553 0.2894404203
H -4.3861262895 3.3257971538 -0.2243537396
H -4.093407215 4.3349360799 1.2010416814
H -3.395814898 4.7983501758 -0.3520924772
C -2.8242404393 1.9723584886 1.5769723853
H -3.2775186131 2.3793813979 2.490874542
H -3.5750977115 1.3239860404 1.1026313219
H -1.9699375594 1.3492110935 1.8726024575
C -4.9996409681 -1.2789264398 -0.600194375
C -2.8653805192 -1.3487175632 0.8342539814
C -2.1433395441 -1.2974513722 -0.3083369486
C -2.754508435 -1.1202484208 -1.672771294
C -4.1523582744 -0.5137502119 -1.601491317
H -2.0910640799 -0.5143273207 -2.3109712299
H -4.6150637504 -0.5290739327 -2.596876162
C -1.5794527922 -3.9507284701 0.1092798891
H -2.6008787004 -4.0085308912 -0.28380383
H -1.6282010517 -3.8541843938 1.1979002038
C -0.7830253604 -2.8700336526 -0.5592975257
H -0.906422672 -2.8461981022 -1.6457232252
C 0.555241688 -2.5751657394 -0.1562023037
C 1.5569997113 -2.6106689493 -1.2947153204
H 2.5339152468 -2.2128156657 -0.9740757604
H 1.2027184181 -2.003647678 -2.1474629229
H -1.0675205508 -4.9000571396 -0.1181324225
C 1.0789248725 -3.0903546618 1.1547008488
H 1.382886246 -4.142123033 1.0295414492
H 1.9723528845 -2.5450400319 1.4848765116
H 0.3436290544 -3.0467433942 1.9649056505
O 1.659462411 -3.9761847335 -1.6470872052
H 2.3896029254 -4.1112451774 -2.2679982679
H -5.1037060865 -2.329590871 -0.9251447216
C -4.3616142365 -1.2877080861 0.7653665044
O -5.0171155693 -1.280452857 1.7906390133
C -2.322234593 -1.5013819049 2.2173633545
H -2.6555716076 -0.6645365248 2.8456740535
H -2.72214577 -2.4026962593 2.7029921948
H -1.2257809378 -1.5201937957 2.2354954175
H -6.0149454963 -0.8778682957 -0.5019753456

H -4.0952413493 0.5435590301 -1.2962445115
H -2.8304756394 -2.1024491574 -2.174203611

ts2_2a_s_down

Imaginary Frequency: -232.21
Pd -0.1580425845 -0.5838601595 -0.0470580633
C 1.7072355998 1.7817594062 -0.4936151582
C 2.7957284516 2.596802417 -0.7658800119
C 4.0771863362 2.0912985533 -0.5599999148
C 4.2021378017 0.7950055998 -0.088695566
C 3.0522667017 0.0382019501 0.1424256122
N 1.8256127269 0.5077399258 -0.0560259178
H 4.957363974 2.6978747119 -0.7614306848
H 2.6349227165 3.608987647 -1.1283604318
H 3.1391251801 -0.9863791034 0.5021584441
C 5.5360401556 0.1513697724 0.1824093492
F 6.5477563129 0.9743836158 -0.0764950168
F 5.6203017329 -0.2285094071 1.4631331235
F 5.6859052038 -0.9484617463 -0.567265447
C 0.3311317189 2.2716387488 -0.6242048672
O 0.1480381075 3.4881202208 -1.1247830622
N -0.6956918045 1.5998918091 -0.2482702667
C -1.2924355094 3.6256631878 -1.258687358
H -1.5537662367 4.6454988557 -0.9656714668
H -1.5293057279 3.4798409386 -2.3197597856
C -1.8530719729 2.5196123043 -0.3531018638
H -2.6842897096 1.9937037266 -0.847114956
C -2.3565823243 3.0052184096 1.0283616415
C -1.2908845587 3.8070690663 1.7751798442
H -0.3878430932 3.2099495932 1.9675827059
H -0.9954633627 4.7252753048 1.2491486577
H -1.6833453981 4.116941241 2.752879713
C -3.5948579108 3.8725934276 0.7929839563
H -4.3859778136 3.3110733117 0.2734308626
H -4.0087945085 4.2052767347 1.7540256803
H -3.3802872462 4.7781623299 0.2085157073
C -2.7604588435 1.8032881746 1.8749047461
H -3.1702979915 2.1393124583 2.8369702187
H -3.541667031 1.2098321698 1.3781719694
H -1.904833675 1.147028313 2.0830867731
C -4.7916322008 -0.6719103781 -1.2827450849
C -3.0595695526 -1.3036043672 0.5300688671
C -2.1018462806 -1.2465403233 -0.4233331654

C -2.386702892 -0.9445871628 -1.8703654629
C -3.8176791106 -1.300969035 -2.2626799518
H -2.2221297723 0.1344862562 -2.0406622671
H -3.9430567031 -2.3944497065 -2.2642725084
C -0.8399946127 -2.9114400388 -0.1158367247
C 0.5388058547 -2.5729965066 0.0372040653
C 1.4927297181 -2.8566217726 -1.0934450217
H 2.4918991741 -2.4480605791 -0.9016346581
H 1.1442726966 -2.4589275838 -2.0549006468
C 1.0869328087 -2.8162519709 1.4263507162
H 2.0472517049 -2.294231026 1.5785808777
H 0.3806639823 -2.4491478879 2.1910781022
H -5.8351821871 -0.9449608206 -1.4793283198
C -4.4834128548 -1.018390918 0.1532829497
O -5.3507037287 -1.0227847698 1.0075324677
C -2.8505223252 -1.6429299075 1.9708788053
H -3.4588960345 -0.9929488163 2.6100701236
H -3.1958253235 -2.6667065796 2.1842549437
H -1.8021798466 -1.5469188192 2.2751302531
H -4.7430533227 0.4303683339 -1.3608632869
H -4.0214521887 -0.9604519559 -3.2862104173
H -1.6651089503 -1.4477344486 -2.5284171731
C -1.3050909079 -3.7468863773 -1.2670490169
H -0.876622706 -4.7496157967 -1.1064662462
H -2.394496651 -3.847660349 -1.2924310124
H -0.9504144057 -3.4057677282 -2.2451270414
O 1.2561179221 -4.2169933558 1.5119018216
H 1.6531434139 -4.4584543638 2.3612344329
H 1.6171602389 -3.9454662878 -1.2038544745
H -1.3387110674 -3.1498241819 0.8241577434

ts1_2aa_r_up

Imaginary Frequency: -231.29
Pd -0.6272543565 -0.6872068383 0.0309719669
C 0.1264471295 2.2495065249 -0.486521931
C 0.7873470338 3.4417452982 -0.745764743
C 2.1636604151 3.4970276071 -0.5341521219
C 2.8053924989 2.3565816342 -0.078620413
C 2.0610251635 1.1974041981 0.1433468633
N 0.7513500052 1.1381454004 -0.0521620981
H 2.7208276844 4.4119554758 -0.7231341712
H 0.2320120012 4.3050115654 -1.1032744011
H 2.5511102504 0.2843362131 0.4806572304

C 4.2912394153 2.2871585694 0.1531304775
F 4.8680916613 3.4816979367 0.0504243405
F 4.5526534612 1.7971070873 1.3721116561
F 4.8591897834 1.4661296673 -0.7360557624
C -1.3250531941 2.1141939627 -0.6415699924
O -1.9807528615 3.1444759015 -1.1617349633
N -1.99519718 1.0760535344 -0.2809001126
C -3.3447993626 2.6845268965 -1.3312582739
H -4.0054594109 3.5082738996 -1.0505355115
H -3.4794346787 2.4573584553 -2.3955950767
C -3.42672488 1.4489680763 -0.4261822182
H -3.9585898755 0.6300000027 -0.932305586
C -4.1179007156 1.7068323325 0.9355691144
C -3.4626077291 2.8585611005 1.6976050001
H -2.412686599 2.6414943125 1.9413802812
H -3.5010326224 3.8114373321 1.1516194291
H -3.9864124679 3.0164371733 2.6497835935
C -5.5893687955 2.0286329514 0.6659351867
H -6.086278966 1.2153294506 0.1164812775
H -6.1238624726 2.1564213125 1.6165874128
H -5.7286881679 2.9575533543 0.0966415463
C -4.0487735895 0.4455294771 1.7881447544
H -4.5658778974 0.6020478163 2.7443078389
H -4.5418973831 -0.4000492188 1.284391384
H -3.0093284749 0.1676101898 2.010760074
C 3.5135807614 -2.8575833047 1.3719210587
C 1.9990116952 -1.9882713872 -0.5270081591
C 0.9426602154 -2.0672190227 0.3221283879
C 1.0935241322 -2.4371884491 1.7803312471
C 2.5075854929 -2.1894451148 2.2924035427
H 0.3680025079 -1.8774202665 2.3900305011
H 2.7060468108 -1.1068413156 2.3507413052
C -2.9399199763 -2.1437367678 -1.0795640533
H -2.5233138587 -1.6616317667 -1.9810031289
H -3.7061930744 -1.470584011 -0.658456537
C -1.8765930809 -2.3841673102 -0.0324407318
C -0.6530592372 -3.036629502 -0.4237741227
C -2.4245322125 -2.7418787561 1.3245351074
H -3.1176289201 -1.988545182 1.7104861195
H -1.6362676805 -2.9010561228 2.0693828424
H 3.3470826379 -3.9489476176 1.3673821538
C 3.3702777025 -2.3698354857 -0.0457151296
O 4.3180886042 -2.286135997 -0.8043554609
C 1.9668269351 -1.5099745756 -1.9430659034

H 0.9788844687 -1.1435043783 -2.2412150205
H 2.6906672677 -0.692339729 -2.0703652542
H 2.2998732579 -2.2894457567 -2.6413579124
H 4.5529247039 -2.6999479357 1.6822190166
H 2.6013211572 -2.5735191652 3.3161558214
H 0.8489308837 -3.5040671844 1.9306624825
H -0.342235163 -3.7901518789 0.305430205
H -2.9890353041 -3.6831765678 1.2315164406
C -0.4167124177 -3.4920539818 -1.8331512298
H -0.6079488089 -2.7253698659 -2.5908685853
H 0.5996359504 -3.8725881678 -1.9698992255
H -1.11785359 -4.3166972529 -2.0229120066
O -3.4908860355 -3.4110757564 -1.3783017405
H -4.2535604603 -3.3163239337 -1.9660228028

ts1_2aa_s_up

Imaginary Frequency: -223.73
Pd 0.693435131 -0.5549887383 0.2626712234
C -0.2713858745 2.3382224397 0.4846013972
C -1.0347937413 3.4674891628 0.7413486677
C -2.4170991102 3.3837553517 0.5781880585
C -2.9615371794 2.1789599556 0.162168052
C -2.1179960062 1.0916016748 -0.0691430384
N -0.8035729994 1.164682436 0.0843263361
H -3.0565827283 4.2425515753 0.77102838
H -0.5552781041 4.3888404641 1.0627093837
H -2.524689721 0.1287555258 -0.3848440881
C -4.4424318618 1.9714437605 -0.015233463
F -5.1146603558 3.1179321084 0.0616199528
F -4.6985830474 1.411636533 -1.2032965508
F -4.9103531007 1.1463556083 0.9262766725
C 1.19283963 2.3343211628 0.5771354042
O 1.8124412905 3.4849515486 0.8057556632
N 1.910346518 1.2863245633 0.3736633688
C 3.2247565189 3.1496167013 0.8752721088
H 3.7722131721 3.9409848861 0.3572786914
H 3.5045379159 3.1437990878 1.9353572832
C 3.3020762692 1.7652077131 0.2174190155
H 3.9845643638 1.1035965954 0.7713639247
C 3.7397315163 1.7876036881 -1.2720351725
C 2.8504888371 2.7061457672 -2.1101504303
H 1.8052520334 2.362067189 -2.1240000198
H 2.8673474754 3.7494588029 -1.7663023545

H 3.2004664674 2.7080188721 -3.1509228038
C 5.1902749866 2.2710454736 -1.3299599364
H 5.850007621 1.6396255967 -0.7166550157
H 5.5598723829 2.2258705905 -2.362995025
H 5.3106242184 3.3107052657 -0.9961010792
C 3.6809903772 0.3789367123 -1.8585720846
H 3.9807756589 0.4006947324 -2.9149138372
H 4.3719051948 -0.2989285814 -1.3377402227
H 2.6676570764 -0.0487197531 -1.8091872766
C -2.9704362412 -2.7040479646 -1.9286456807
C -1.8736559844 -2.1477137067 0.3495369158
C -0.6887774956 -2.0094006273 -0.2915535343
C -0.5420482941 -2.1739719312 -1.7818667432
C -1.5786468773 -3.1354526603 -2.356219997
H -0.6764705497 -1.1793077995 -2.2476362212
H -1.5015204406 -3.160013377 -3.4507774424
C 2.1572643852 -1.5853078771 2.5182342424
H 1.2197940564 -1.4231445701 3.0644866046
H 2.7478874799 -0.6606140825 2.5596857035
C 1.9281826173 -2.0401334447 1.0984306088
C 0.7956399466 -2.8849631626 0.8372359852
H 0.1422008143 -3.0076113729 1.7030178323
C 0.931139467 -4.1431686007 0.024538752
H -0.040523239 -4.6186289726 -0.1428640183
H 1.4321662237 -4.0029076374 -0.9393567957
H 1.5620250748 -4.8303374532 0.6054101661
C 3.1984213035 -2.2699958159 0.3175720327
H 3.8622874988 -1.3976831914 0.4388549546
H 2.9935763776 -2.3853851381 -0.7608467737
H -3.7528660877 -3.4059296129 -2.2406439312
C -3.1010478966 -2.4952996915 -0.4394874977
O -4.1827938457 -2.539635388 0.1181359264
C -2.1151661401 -1.9224791245 1.8077528395
H -1.2563160277 -1.4606422149 2.3087334092
H -2.9864689636 -1.2671246951 1.9450358041
H -2.3771460375 -2.8596547939 2.3200823597
H -3.2235218712 -1.7372229068 -2.4018595283
H -1.373200922 -4.1566960184 -2.0011418861
H 0.4768614593 -2.4841599167 -2.0504352604
H 2.7283047837 -2.3608633527 3.0522167857
O 3.7894834768 -3.4338856725 0.8602493121
H 4.6587531239 -3.5839856982 0.4626028906

ts1_2aa_r_down

Imaginary Frequency: -213.04
Pd -0.6015767088 -0.7195703451 0.0787348086
C 0.2447352144 2.1964123157 -0.2678749228
C 0.9589623527 3.3794196999 -0.3885237813
C 2.2841826579 3.3979792615 0.0432872477
C 2.8260535749 2.2372146331 0.5736107036
C 2.0416307321 1.0841125193 0.635230223
N 0.7840320715 1.0603450478 0.2138708196
H 2.8800475983 4.3051621898 -0.0290889138
H 0.48364944 4.2646769447 -0.8033716301
H 2.4454273585 0.1503457588 1.0331979365
C 4.2373903408 2.1579808256 1.0935718674
F 4.9074928607 3.288632534 0.8835653039
F 4.2390243984 1.9044614742 2.4052591244
F 4.8998485401 1.1612157758 0.4925022548
C -1.181683588 2.0990248667 -0.5985990913
O -1.7273268339 3.1085162401 -1.2673018737
N -1.931494728 1.11545958 -0.2412842741
C -3.0791178649 2.6762273235 -1.568075602
H -3.7373269001 3.5388343582 -1.4408021166
H -3.0900446949 2.3614177603 -2.6185945085
C -3.3184459863 1.5240530251 -0.5841757358
H -3.8254101862 0.6840460411 -1.0817925459
C -4.1492981626 1.9175460584 0.6629375388
C -3.5667974695 3.1363503343 1.3774216854
H -2.5389386295 2.9554228337 1.7231896026
H -3.5701515303 4.0414794563 0.7549807324
H -4.1692251634 3.362026235 2.2672999209
C -5.5764405141 2.2160699185 0.1990667013
H -6.0253559077 1.3467427648 -0.304047677
H -6.2093609606 2.4577145347 1.0632140935
H -5.6358691839 3.0718941338 -0.4875692947
C -4.1917954324 0.7483341172 1.6408028645
H -4.8376361736 0.9907908617 2.4952825734
H -4.6034404193 -0.1556497326 1.166465579
H -3.1919877581 0.5167363436 2.0324712361
C 3.8462185414 -2.3378174329 -0.79022397
C 2.8070867832 -2.6061407662 -1.8633862081
C 1.5641321275 -1.7568826439 -1.6069708952
C 1.0354970642 -1.9590347393 -0.2131572922
C 1.8269164992 -2.2943110599 0.8305466092
H 3.2125275705 -2.3835171074 -2.8588195429
H 1.824978575 -0.6895611099 -1.7263088224

C -2.9823015604 -2.1538139259 -0.9324222541
H -2.5236548607 -1.7969153476 -1.8723338608
H -3.6945578465 -1.3828936724 -0.5918265779
C -1.9416931013 -2.3609016337 0.1441009323
C -0.7593049111 -3.0982930004 -0.1611566803
C -2.5039108247 -2.4910471704 1.5343365048
H -3.0535272227 -1.5993337721 1.8520346554
H -1.733723777 -2.7104845626 2.2825832838
H 4.2337493678 -1.3080352854 -0.8903240046
C 3.3017707558 -2.4576451585 0.6124753903
O 4.0357508623 -2.6238478342 1.5696879483
H 4.7210972642 -2.994750109 -0.8664362148
H 2.5354106776 -3.6729416269 -1.8633978794
H 0.7924312248 -1.9515717096 -2.3622731568
C 1.3844137134 -2.4589514338 2.2486276134
H 1.550822208 -3.4874396667 2.6001533779
H 1.9893663298 -1.8256286831 2.9112004465
H 0.3322838891 -2.1888422074 2.3893846664
H -0.3375222207 -3.6198988434 0.6987869928
H -3.2193089971 -3.3287860306 1.5370577501
C -0.6140006695 -3.8440688471 -1.4527195546
H 0.4023862445 -4.2170270333 -1.610290512
H -1.2869876799 -4.7107021982 -1.388065545
H -0.9304987553 -3.2700370075 -2.3306655922
O -3.6262767406 -3.3983323177 -1.1101524219
H -4.3909198749 -3.3020777521 -1.6951140357

ts1_2aa_s_down

Imaginary Frequency: -224.12
Pd 0.6596831662 0.6953284812 -0.2520372776
C -0.2546889413 -2.1811047145 -0.647741662
C -0.9858793739 -3.3294985522 -0.9125985012
C -2.3503409086 -3.3244348805 -0.6262189088
C -2.9111463704 -2.1768847681 -0.0874380758
C -2.1034719927 -1.0573896743 0.1238455612
N -0.8079855068 -1.0529961035 -0.1582670705
H -2.9636254183 -4.2031146347 -0.8147438612
H -0.4936608755 -4.2093121267 -1.3195548878
H -2.5200840101 -0.1347100055 0.5314519614
C -4.3611424647 -2.1005359838 0.3129527691
F -5.0597706712 -3.1257950052 -0.1708311205
F -4.4787678248 -2.1044416287 1.6437762545
F -4.9167886967 -0.9671124844 -0.1339104703

C 1.2058550878 -2.127624954 -0.7824460822
O 1.8316786712 -3.1931101997 -1.2653354784
N 1.9156153764 -1.1361389849 -0.3692334286
C 3.2360146341 -2.8194562955 -1.3063999838
H 3.8150265395 -3.6947347846 -1.0023892039
H 3.471954322 -2.5673863882 -2.3474705642
C 3.3169302341 -1.6188437272 -0.3561329561
H 3.9772833746 -0.8379767033 -0.7623132742
C 3.7968104346 -1.9727684691 1.0785918914
C 2.9630773112 -3.0950257543 1.6982804378
H 1.9083734109 -2.8060368025 1.8169377981
H 3.0052500892 -4.0298475722 1.1234631101
H 3.3452049835 -3.3224520139 2.702232849
C 5.2628595342 -2.4031672071 0.9913270723
H 5.8836273535 -1.6260541151 0.5213002614
H 5.6624268108 -2.5763692249 1.9994043117
H 5.4046069238 -3.3362868741 0.4295792523
C 3.7105093143 -0.7470899853 1.985375179
H 4.0286232075 -1.0150840639 3.0018343512
H 4.3770967248 0.0550840827 1.6395872638
H 2.6866233621 -0.3519804233 2.0500691908
C -3.6858087889 2.7828564115 -0.5048388559
C -3.0274612652 2.0019413051 -1.6309172042
C -1.5315304791 2.2924705603 -1.66192405
C -0.8908134638 2.1030514701 -0.3101459503
C -1.5733238942 2.197875861 0.8554626342
H -3.201706526 0.9224231914 -1.4976309147
H -1.0285534745 1.6627282278 -2.4122527284
C 2.4163959765 2.0163059568 -2.1380473136
H 1.5490795677 2.0461233858 -2.8098892502
H 2.9383761148 1.0613924529 -2.2876630792
C 2.0330804476 2.2211148507 -0.6940438767
C 0.9223432236 3.0817706341 -0.403704752
H 0.4984734895 3.5243560089 -1.3081361729
C 0.9239518718 4.0163693156 0.7692521031
H -0.0466292299 4.5085981387 0.8891652982
H 1.1820171558 3.532347272 1.7165347174
H 1.6873664221 4.7833950225 0.5778910128
C 3.2051805352 2.2072544334 0.2550471544
H 3.8362542402 1.3302225618 0.0340223991
H 2.8801601811 2.1306588815 1.3062868997
H -4.7584978748 2.5755625663 -0.412413556
C -3.0417499758 2.5022247068 0.82937717
O -3.6736844583 2.5050686174 1.8700313625

H -3.5893848058 3.8657170229 -0.6977084657
H -3.4754099744 2.2669044687 -2.5973061596
H -1.3892000991 3.3344182679 -2.000319629
C -1.013699607 1.9439544685 2.2192610373
H -1.1748527095 2.803664438 2.8835806987
H -1.543666524 1.1051376371 2.6935098777
H 0.0539769694 1.6923932544 2.1894694891
O 3.9077786742 3.4113312024 0.0184923115
H 4.7069590399 3.4436931339 0.5630189204
H 3.1088404294 2.8160738147 -2.4435788358

ts2_2aa_r_up_a

Imaginary Frequency: -249.10
Pd 0.1772515708 0.6987112911 -0.0878500309
C -1.5459555878 -1.7685314293 -0.4626991466
C -2.58933109 -2.6529219805 -0.6903060989
C -3.8896818154 -2.2329667233 -0.4194719467
C -4.0792533137 -0.9491607463 0.0670589051
C -2.9735795216 -0.1160625953 0.2435625665
N -1.7312565937 -0.5043133219 -0.0208845371
H -4.735709598 -2.8970163311 -0.5816825906
H -2.3816431169 -3.6520706831 -1.0652164475
H -3.1099202335 0.901320224 0.6120438398
C -5.4393920667 -0.3935107043 0.3958765956
F -6.4014735237 -1.2938751012 0.2205549608
F -5.4804604174 0.0252054378 1.6664586842
F -5.7092342465 0.6660899443 -0.3786852805
C -0.1420816217 -2.1604685083 -0.632189431
O 0.1307804067 -3.2901020791 -1.2751621838
N 0.8306746208 -1.4713586795 -0.1563629616
C 1.5799113683 -3.2897311093 -1.4230278442
H 1.9259139802 -4.316245119 -1.2807363114
H 1.7943652957 -2.9733805885 -2.451415611
C 2.0526227587 -2.2797446102 -0.3686569682
H 2.8432070574 -1.628724825 -0.7716209077
C 2.5739229055 -2.9137942038 0.9449743166
C 1.5859183486 -3.9252369633 1.5252361148
H 0.6137315488 -3.4624997271 1.7503186637
H 1.4142658567 -4.7852042272 0.8638130293
H 1.9789294977 -4.3241428855 2.469895815
C 3.9077792107 -3.5943928511 0.632222476
H 4.6390109332 -2.8753380094 0.232267994
H 4.3335903903 -4.0287806073 1.5465849374

H 3.809106136 -4.4122424545 -0.0947744832
C 2.8188289172 -1.8201685406 1.9796252312
H 3.254299845 -2.2514588141 2.8910338148
H 3.5285058234 -1.0715859942 1.5987828223
H 1.8879794239 -1.3084426868 2.2614772183
C 4.9437324671 1.2061160823 0.4480674841
C 4.0895288665 2.1908099646 1.2271430994
C 2.6280744499 1.7435324527 1.249610675
C 2.1158925304 1.4556296479 -0.1321570235
C 2.9089775927 1.0522463253 -1.1519278495
H 4.4634826114 2.2938444381 2.2539789015
H 2.5256951073 0.8283495001 1.8549053154
C 1.4763582821 4.106701056 -0.2456344883
H 2.5380269337 4.1111932047 -0.5096143013
H 1.3661629666 4.2213031165 0.837003093
C 0.7592437648 2.9223801254 -0.8241004276
H 0.9939292182 2.7609678167 -1.8787725975
C -0.5986188942 2.6236899449 -0.4869500967
C -1.5732217251 2.4930375759 -1.6310120432
H -2.4957320104 1.9743387123 -1.3435837992
H -1.1353139018 1.9740606704 -2.4934064702
H 1.0060115915 4.9939054644 -0.6948535564
H 5.0948359907 0.2790545316 1.0274147082
C -1.1758494392 3.2181722141 0.7767309794
H -2.0688525383 2.6455141208 1.0832715487
H -0.4523131203 3.1587660786 1.6078425739
C 4.3548126491 0.7855660961 -0.8775027769
O 5.0230786906 0.1762790987 -1.6960364703
H 5.9529404178 1.5904296579 0.2521441817
H 4.1590283788 3.1880138621 0.7677851243
H 2.0065942128 2.4913149299 1.7574655406
O -1.5134069306 4.5555002222 0.4753487656
H -1.9826646785 4.960367073 1.2186513278
H -1.8680996615 3.5019197867 -1.9582678323
C 2.4599583656 0.7355388669 -2.5440291868
H 2.8986680748 1.4364282612 -3.2693490968
H 2.820979589 -0.2560230665 -2.8478672821
H 1.3682901395 0.7514517411 -2.6471912444

ts2_2aa_r_up_b

Imaginary Frequency: -246.89
Pd -0.1392328606 -0.6753321273 0.010057495
C 1.6318324546 1.7349389697 -0.4456685967

C 2.7015904175 2.5793125744 -0.7015609259
C 3.988674189 2.1258585059 -0.4204320941
C 4.1403817076 0.8535759286 0.108668447
C 3.0096765442 0.061392207 0.3134273576
N 1.7818191202 0.4799165783 0.0319839911
H 4.8549298317 2.7567750408 -0.6065267624
H 2.524513845 3.5730389596 -1.1056706669
H 3.1073222297 -0.9464953694 0.7188783541
C 5.4832176135 0.2688936357 0.4553800406
F 6.4759013431 1.1205242774 0.2177349522
F 5.5264740401 -0.0781998106 1.7471565432
F 5.7049064367 -0.8438829611 -0.2596959501
C 0.2371826644 2.1592998055 -0.6143849804
O -0.0153815933 3.272184911 -1.2943415657
N -0.7452958824 1.5128720897 -0.1007438869
C -1.4664254411 3.3046899598 -1.4208122738
H -1.7829822543 4.3442314591 -1.3070018131
H -1.7051311411 2.9600402358 -2.4348148055
C -1.9494778972 2.343299236 -0.3263047751
H -2.7618484994 1.6980712167 -0.6945116392
C -2.4369240988 3.0349050915 0.9711156751
C -1.4133392071 4.0348945045 1.5072412661
H -0.4540544458 3.549937155 1.74033016
H -1.2214810634 4.8655767175 0.8144522113
H -1.78501668 4.478203233 2.4407055271
C -3.7539729075 3.7439040982 0.6501137695
H -4.5098152764 3.0338160169 0.2813459507
H -4.1568801351 4.219021203 1.5545431368
H -3.640275323 4.5354865452 -0.1034373405
C -2.7015399951 1.9826073338 2.0432953177
H -3.1170623554 2.4554014918 2.9434258147
H -3.4339164875 1.2405681582 1.6937430819
H -1.7825786585 1.455124959 2.3344876936
C -4.8894105875 -1.0978732952 0.74065365
C -4.0057200467 -2.0660056862 1.5085594175
C -2.5446313449 -1.6215147049 1.4587424652
C -2.0962317679 -1.3792641374 0.0478045871
C -2.9263664928 -0.9911903893 -0.9477378702
H -4.3372253305 -2.1401507372 2.5524207052
H -2.4229383421 -0.6816689512 2.021931182
C -1.4904986977 -4.0328481534 -0.0306885368
H -2.5540135468 -4.0509880961 -0.2878101135
H -1.3710977482 -4.0889190285 1.0539580268
C -0.7833033716 -2.8661810738 -0.6617610178

H -1.0641754756 -2.718202684 -1.7068139662
C 0.6032827993 -2.6132592106 -0.4121287757
C 1.507743025 -2.5100151744 -1.6166573771
H 2.4384378474 -1.9680402158 -1.4082902721
H 1.0097258443 -2.0269200704 -2.46629622
H -1.0345744084 -4.9411182175 -0.4573660606
H -5.0130962202 -0.157309307 1.3044972757
C 1.2743451984 -3.2307599748 0.7904516753
H 1.3256694408 -4.326448876 0.6291296654
H 2.3202767424 -2.8801590654 0.8463372916
C -4.356055303 -0.7090178414 -0.6185355071
O -5.0590221383 -0.1194176696 -1.4230970114
H -5.9072403326 -1.4820736074 0.5953914092
H -4.0972403917 -3.0750157002 1.0795612671
H -1.8864994654 -2.3379343586 1.9636731866
O 0.5690499121 -2.9183526603 1.9691969952
H 0.9764129902 -3.3690872249 2.7226267973
H 1.7983477168 -3.5224978983 -1.9435695125
C -2.533605801 -0.7174011832 -2.3662810924
H -2.9844813418 -1.4502522376 -3.0518194034
H -2.9222460979 0.2571332908 -2.6903138553
H -1.4457660784 -0.7183944902 -2.5061993141

ts2_2aa_r_up_c

Imaginary Frequency: -245.68
Pd -0.111504125 -0.6516930808 0.0260135108
C 1.6384618392 1.770589264 -0.4895480103
C 2.6855371627 2.6298259475 -0.7828684123
C 3.9867811815 2.2010604223 -0.5286085076
C 4.1694696392 0.9378590828 0.0097025751
C 3.0604871192 0.1241341019 0.2553079338
N 1.8174904985 0.5236310357 0.0013538717
H 4.837445819 2.8438892946 -0.7434902371
H 2.4818717621 3.6152547765 -1.1944289835
H 3.1745126677 -0.8783193343 0.6703235118
C 5.530752802 0.3796370782 0.3269540089
F 6.5011730781 1.254161967 0.0716109128
F 5.610292901 0.0320715905 1.6169678072
F 5.7636688818 -0.7248604944 -0.3955324468
C 0.2362703604 2.1749971749 -0.6352694772
O -0.0437949108 3.2820957767 -1.3136809604
N -0.7315384213 1.5179318724 -0.1052774317
C -1.4958335034 3.2944463522 -1.4188925445

H -1.8257246972 4.330095874 -1.3073396703
H -1.7448199078 2.9400285046 -2.4270736535
C -1.9493861621 2.3323648066 -0.3129729245
H -2.7589358595 1.6770729379 -0.6680959531
C -2.4260442559 3.0219073845 0.989270036
C -1.4074535263 4.0372783204 1.5061121744
H -0.4377042835 3.5665436993 1.7244433029
H -1.2385680175 4.8678248257 0.8073723136
H -1.7699090413 4.4790237504 2.4439240108
C -3.7570425059 3.7126023053 0.6866318876
H -4.508965609 2.9920246715 0.3300566644
H -4.1530721005 4.1847686679 1.595649023
H -3.6642768879 4.5034423141 -0.0703987877
C -2.6609018441 1.9701710727 2.0690362884
H -3.0633777208 2.441656506 2.9757449092
H -3.394331654 1.2218793332 1.7348077205
H -1.7316030418 1.4519090075 2.343915965
C -4.8627701941 -1.1129423987 0.7416404878
C -3.9736682269 -2.0841830315 1.4992544831
C -2.5173694728 -1.6237769921 1.4556166294
C -2.0639767196 -1.353686398 0.0500291792
C -2.898614405 -0.9694149791 -0.9430364341
H -4.3064622485 -2.17494257 2.5413090559
H -2.4016530017 -0.6959093915 2.0391308555
C -1.5438104876 -3.9989493276 -0.1678533559
H -2.5795157368 -3.9738404874 -0.5205917165
H -1.5436420832 -4.1060121573 0.9216507986
C -0.7511618925 -2.8321115995 -0.6913352575
H -0.9424267262 -2.6433940307 -1.7502330668
C 0.6113818216 -2.6242719729 -0.3124793314
C 1.6263715496 -2.5344912705 -1.4254636271
H 2.5595718656 -2.0534830342 -1.1194586581
H 1.2241428019 -2.0067600417 -2.2997065807
H -1.0735084421 -4.9079076005 -0.576069793
H -4.984792059 -0.1761226007 1.3121580134
C 1.0913708042 -3.2801555618 0.9563787682
H 0.3309842945 -3.2001703306 1.7512269479
H 1.2410738081 -4.3587315094 0.7477528099
C -4.3350988891 -0.7159021907 -0.617342188
O -5.0473096477 -0.139986029 -1.4228667976
H -5.8801077666 -1.499112191 0.599523037
H -4.0543710803 -3.0875221675 1.0552994794
H -1.8622357892 -2.3518146916 1.9519451292
O 2.3004576965 -2.6819858581 1.3646054544

H 2.6839451851 -3.1833905945 2.0974665685
H 1.8907277718 -3.5562594827 -1.7460807073
C -2.5064345032 -0.6787449655 -2.3582249854
H -2.8962422273 -1.4469231536 -3.0431377484
H -2.963116584 0.2621198359 -2.6919480998
H -1.4191852722 -0.6063143255 -2.4863317469

ts2_2aa_s_up

Imaginary Frequency: -247.09
Pd 0.2218569794 -0.6377779462 -0.3212428756
C -1.3818678561 1.6752577247 0.8003615345
C -2.3632422876 2.4434679408 1.4080473911
C -3.6985744497 2.0953464947 1.2138480688
C -3.9828363371 1.0050022298 0.4080984335
C -2.9322294405 0.283719856 -0.1615451783
N -1.6562880213 0.5918663543 0.0366492883
H -4.4993378669 2.6664028186 1.6786841829
H -2.0803758537 3.2978843041 2.0180512279
H -3.1452985803 -0.5729487824 -0.8016075306
C -5.3863216381 0.5346638869 0.1353152117
F -6.2940638802 1.298146722 0.7354616305
F -5.6379910259 0.5325310384 -1.1778615532
F -5.5433663025 -0.7242648348 0.571004449
C 0.0419360362 2.0206039984 0.8861730784
O 0.4099614986 3.0486108333 1.6420534161
N 0.9424934662 1.4021406246 0.2140880538
C 1.8667031733 3.0447730818 1.590577993
H 2.1950920456 4.0857903655 1.5376540259
H 2.2187685375 2.5993517618 2.5294699682
C 2.1857206892 2.1821863733 0.3605182092
H 3.019008756 1.4940522432 0.5710774294
C 2.5129692853 2.9724559078 -0.93272964
C 1.4652373523 4.047119271 -1.2259978345
H 0.4634606246 3.6162624547 -1.3692141967
H 1.4031578958 4.8149821129 -0.4428536622
H 1.7244446542 4.5662546473 -2.1583721873
C 3.8848546099 3.6219804229 -0.7490989313
H 4.6672790614 2.8700629527 -0.5693514674
H 4.1642453258 4.1799806524 -1.6526820767
H 3.9063930958 4.3351419658 0.0867392994
C 2.5671890609 2.0171579754 -2.1242420044
H 2.846058941 2.5682759112 -3.0324546449
H 3.317816243 1.2281395416 -1.9794802309

H 1.5941842509 1.5396482155 -2.3064618464
C 4.9342140617 -1.6709329957 0.4627921042
C 4.4661541303 -1.0102697643 -0.8233334735
C 3.1019718886 -1.5589096555 -1.2251834996
C 2.1164029694 -1.5452835591 -0.0866529295
C 2.4882733072 -1.4857672848 1.2149343424
H 4.4095965633 0.0790834412 -0.6693038159
H 2.6895094948 -1.0140362729 -2.0893324993
C 0.7479133253 -2.9595926495 -0.6461565538
C -0.610672876 -2.5121894646 -0.7663084599
C -1.6101089871 -2.9624554953 0.2762859664
H -2.5109233122 -2.3276840887 0.239828808
H -1.1899112171 -2.8789345146 1.2928740434
H 5.9180584416 -1.3189261293 0.7928405711
C -1.1724425221 -2.3649202932 -2.1575953835
H -2.0133253269 -1.6591841064 -2.1991922092
H -0.4140007771 -2.0322207625 -2.8782573474
C 3.9428662129 -1.4195230435 1.5668923487
O 4.2788563236 -1.1737384058 2.7114445507
H 5.0123990375 -2.7628958303 0.3160373233
H 5.187134253 -1.17489465 -1.6346539418
H 3.2442916017 -2.5995121504 -1.5700914591
C 1.1414128945 -4.0519163737 0.3108119668
H 0.6383590297 -4.9737691962 -0.0135430049
H 2.225067063 -4.2162431819 0.3014157898
H 0.8309372993 -3.8611566192 1.3435678506
O -1.9289998041 -4.3008983525 -0.0434638366
H -2.6407213371 -4.6222248112 0.5282469561
H -1.5600754128 -3.3382112106 -2.4966797817
H 1.2329941471 -3.0412346024 -1.6219674375
C 1.5722481088 -1.4299514286 2.3958184554
H 1.8721535389 -2.1564927399 3.1626596344
H 1.6408382804 -0.4439602021 2.8795659631
H 0.5217907265 -1.5921195661 2.1208472777

ts2_2aa_r_down

Imaginary Frequency: -245.28
Pd -0.1793051796 -0.5760174679 -0.2213395286
C 1.7465854672 1.7773482539 -0.4720648658
C 2.8630509778 2.5844960041 -0.6352875064
C 4.1181916215 2.0523752589 -0.3500380943
C 4.1900468728 0.7405693427 0.0893437272
C 3.0165035908 -0.0046119564 0.2092753121

N 1.8158844331 0.4886512721 -0.0731153714
H 5.019999643 2.6502302173 -0.4622502531
H 2.7433892335 3.6106279696 -0.9736037771
H 3.0627454158 -1.0402484739 0.5453846551
C 5.4912157875 0.0672946457 0.435746843
F 6.533410748 0.8701219534 0.2416926692
F 5.4930539639 -0.3225366369 1.7159731993
F 5.661325994 -1.0309419355 -0.3126735636
C 0.3883859537 2.2921042678 -0.6705069864
O 0.2526601507 3.5133718083 -1.1759006624
N -0.6667058747 1.6395027561 -0.3413639384
C -1.1766081015 3.6791579133 -1.3769386199
H -1.4319523972 4.7036294233 -1.0952985179
H -1.36732521 3.5386537433 -2.4478020978
C -1.7980027965 2.5826948221 -0.5002548367
H -2.6103087407 2.0689684203 -1.0338194292
C -2.3535953981 3.0790632137 0.8578937853
C -1.3158257482 3.8844712771 1.6401442363
H -0.4179827279 3.2898892313 1.8629584903
H -1.0048057899 4.803517694 1.1249475197
H -1.7418317463 4.1926051269 2.6042913162
C -3.5801280066 3.9463575174 0.5691253954
H -4.3549620686 3.3791997898 0.0316099004
H -4.0243179896 4.2949588805 1.5108489634
H -3.3438331147 4.8420043308 -0.0225594724
C -2.7876716612 1.8862867296 1.7035707216
H -3.2323692156 2.2330072413 2.6460854488
H -3.5479547612 1.2808362039 1.189338909
H -1.9373330078 1.2367575398 1.9501822981
C -5.0159936318 -1.1578918936 -0.6861656363
C -2.8607342848 -1.3634073422 0.7058600452
C -2.153505414 -1.2514329335 -0.4425626708
C -2.7855545953 -0.9647138558 -1.7797002189
C -4.1699598596 -0.3395268197 -1.6452506492
H -2.1203024293 -0.3290059941 -2.3864717526
H -4.646650233 -0.277344788 -2.6321760717
C -1.6290789352 -3.925814997 -0.1971494608
H -2.6758482592 -3.9148341805 -0.5217248914
H -1.6020834437 -3.9197180728 0.8965824562
C -0.8418907213 -2.8112163461 -0.8266013925
H -1.010868975 -2.7186430271 -1.903262909
C 0.5231091543 -2.5565450143 -0.4774271781
C 1.5114459859 -2.5152390041 -1.6180319843
H 2.4664019058 -2.0555663489 -1.3375498486

H 1.1112520052 -1.984714713 -2.4918625637
H -1.1698026898 -4.8696066417 -0.5258546253
C 1.0648805722 -3.1382880348 0.8085599392
H 1.9439962068 -2.5605452427 1.1433528266
H 0.3155316292 -3.0847296425 1.6146939156
H -5.1452930757 -2.1810288941 -1.0823879684
C -4.3573577072 -1.2731062788 0.6643978412
O -4.9975692893 -1.329064451 1.6978059609
C -2.3032404908 -1.6249258749 2.0667246641
H -2.6107660181 -0.8294037468 2.7584424341
H -2.7134349399 -2.551878278 2.4918850653
H -1.2070907782 -1.6654563358 2.0669927695
H -6.0221657637 -0.7470956964 -0.5435409309
H -4.0906129732 0.6931151001 -1.2691396738
H -2.8903038572 -1.9077008418 -2.3467464907
H 1.7398562778 -3.5475364618 -1.9239861027
O 1.4216829017 -4.4736676658 0.5204892379
H 1.824253893 -4.887767892 1.2970903768

ts2_2aa_s_down_a

Imaginary Frequency: -249.00
Pd -0.1733601726 -0.5983294434 0.079274718
C 1.7007333437 1.7114815919 -0.5976450053
C 2.7913948876 2.4946853447 -0.945456363
C 4.0713532837 2.0173571722 -0.6733100865
C 4.1914746046 0.7832082297 -0.0563438594
C 3.0398574043 0.0575005997 0.2516055947
N 1.8149113048 0.4948290327 -0.018561083
H 4.9526855576 2.6001002486 -0.9319518549
H 2.6328992868 3.4624559825 -1.414658242
H 3.1296244394 -0.912827081 0.7397365564
C 5.5220504944 0.1614138535 0.274258771
F 6.5353488081 0.9804730901 0.0104257845
F 5.5768486635 -0.176533501 1.5669421792
F 5.6955052915 -0.9625498914 -0.4367159965
C 0.3256921643 2.1864714939 -0.7851399396
O 0.1459795611 3.3502693143 -1.4006470749
N -0.7023952403 1.5584094011 -0.3445081074
C -1.2943602739 3.4862912349 -1.5357850882
H -1.545561455 4.5292737398 -1.3272916382
H -1.5421024141 3.2526527123 -2.5785107491
C -1.8549473794 2.4694080709 -0.5307380238
H -2.6924055546 1.9046691735 -0.967817342

C -2.3418883381 3.08630469 0.8035222984
C -1.2616795785 3.9504155976 1.4529635939
H -0.361488496 3.3664605728 1.6941785181
H -0.9627578818 4.8044628944 0.8291483742
H -1.6390015971 4.3632006163 2.3979743129
C -3.5807810142 3.9323156794 0.5035027554
H -4.3752630784 3.3297595456 0.0379059435
H -3.9880510588 4.3433466561 1.4366253099
H -3.3711343898 4.7866982218 -0.1555112002
C -2.7369311868 1.9721964142 1.7666564298
H -3.122883685 2.3990259996 2.7022613537
H -3.5342650816 1.3437934975 1.3436419925
H -1.8823009746 1.3293521561 2.0169107757
C -4.7996118169 -0.7622996443 -1.1453072912
C -3.0704341271 -1.26026866 0.7117821145
C -2.114194844 -1.3112728486 -0.2451486935
C -2.4013290697 -1.1287176804 -1.7118723242
C -3.8400298237 -1.49114512 -2.0689437744
H -2.2187743441 -0.0706001716 -1.9733128937
H -3.9856081382 -2.5782220399 -1.9756465861
C -0.8859643551 -2.9261777945 0.2064299962
C 0.4928086791 -2.5716464484 0.3731538068
C 1.4567959381 -2.9333476585 -0.7371521501
H 2.4068985108 -2.3903578194 -0.6073421956
H 1.0481920386 -2.6483098444 -1.7230957438
C 1.0638872665 -2.6593320809 1.7659944251
H 1.8980503553 -1.9652035364 1.9313159627
H 0.3095392737 -2.464218506 2.5379272285
H -5.8483023453 -1.0340745318 -1.3142295761
C -4.4911788234 -0.9860178875 0.314635494
O -5.3545905144 -0.9026002934 1.1687245984
C -2.8625193823 -1.4651019915 2.177484175
H -3.4359541182 -0.7274081055 2.7505811245
H -3.2517468854 -2.4451953598 2.4944735991
H -1.8090789777 -1.3836210885 2.4675337258
H -4.7330031299 0.3277714295 -1.3198506828
H -4.0422578812 -1.2372592876 -3.1176545774
H -1.6915833806 -1.6992699719 -2.3255942854
C -1.3420471672 -3.8565306242 -0.8781662648
H -0.9652852158 -4.8551424723 -0.6163007973
H -2.4338892984 -3.9081446104 -0.9349872624
H -0.939670298 -3.6241100326 -1.8694372885
O 1.6627127562 -4.3278771284 -0.6448907875
H 2.3767987374 -4.6021342672 -1.2376775003

H 1.4572196716 -3.6750817383 1.9269086312
H -1.4004576423 -3.0927701728 1.1531247912

ts2_2aa_s_down_b

Imaginary Frequency: -242.29
Pd -0.1944235309 -0.6724304717 0.0888340032
C 1.6803583684 1.6471849812 -0.5668306078
C 2.7644545956 2.4368115731 -0.9168825629
C 4.0467014886 1.9699555874 -0.6354529034
C 4.1697569884 0.7430300905 -0.0074146673
C 3.0236718827 0.0073473005 0.3028047497
N 1.7975038953 0.4350511942 0.0221761005
H 4.9253871418 2.5555615739 -0.8966188406
H 2.6006734704 3.3993164763 -1.3947411434
H 3.1230990775 -0.9709962227 0.7649978203
C 5.501934761 0.1260389441 0.3199921244
F 5.5552770011 -0.2381082598 1.6056700218
F 5.6937555883 -0.9819737055 -0.4129694254
F 6.5118856856 0.95869986 0.0788426837
C 0.3044841677 2.111500844 -0.7643162165
O 0.1154318038 3.2602098937 -1.4054277721
N -0.7209648661 1.4878522937 -0.3092879469
C -1.3249430891 3.3794300087 -1.5467398127
H -1.58608699 4.4264604336 -1.3745919862
H -1.569404849 3.1083338733 -2.5815315565
C -1.8771967123 2.391002587 -0.5103445628
H -2.7181654142 1.8150831678 -0.9259129616
C -2.3557829879 3.0448062702 0.8095259226
C -1.276282162 3.9328360474 1.426788168
H -0.3675833297 3.3622765569 1.6678425082
H -0.9937952448 4.7777005162 0.7833632155
H -1.6461593698 4.3599682305 2.3683818218
C -3.6023664839 3.8741307036 0.4950586186
H -4.3979130817 3.251927323 0.0576766609
H -4.0027213013 4.3131541361 1.4183583339
H -3.4044502066 4.7074455564 -0.1939200178
C -2.7365199849 1.9580155852 1.8092827541
H -3.1256252279 2.4116014169 2.730954772
H -3.5267168372 1.3059647875 1.4093718963
H -1.8736166664 1.3344970347 2.0796804726
C -4.8192080885 -0.7719076848 -1.1921782439
C -3.1181336551 -1.2588756398 0.6948890369
C -2.1495613906 -1.3239787516 -0.2482945018

C -2.4148516551 -1.1465305729 -1.7195971848
C -3.8481367353 -1.5097827278 -2.0963137656
H -2.228329107 -0.089212936 -1.9812269404
H -3.996766385 -2.5959238456 -1.9973461896
C -0.9460753512 -2.9492419617 0.2336045588
C 0.4566748137 -2.6790765392 0.3345599882
C 1.3233506838 -3.1221448449 -0.8197953589
H 0.8937971629 -2.7941951514 -1.7823331689
H 1.3276392561 -4.2306757108 -0.823542814
C 1.0845674101 -2.7794763932 1.7014223925
H 1.9481773998 -2.1205407397 1.8322734152
H 0.3623272937 -2.5616040951 2.496663094
H -5.8652334595 -1.0483587656 -1.3699150734
C -4.529097624 -0.9743857591 0.2751459516
O -5.4035814521 -0.8674289783 1.115668551
C -2.9333333968 -1.4536872394 2.1657476302
H -3.5204972425 -0.7143128581 2.7223696773
H -3.3222662275 -2.4334298932 2.4843395438
H -1.8860485932 -1.3621159196 2.4743188732
H -4.754224661 0.3158074806 -1.3798110558
H -4.0341639584 -1.2628658538 -3.1496800457
H -1.694750247 -1.715787991 -2.3228520493
C -1.4811800054 -3.896720905 -0.8047990644
H -1.0901819388 -4.8986081968 -0.5675934123
H -2.5737949842 -3.9542524281 -0.7721870797
H -1.172539394 -3.6696313359 -1.8311102407
O 2.6298266449 -2.6260619114 -0.6582196252
H 3.2322286186 -3.0679026195 -1.2722322226
H 1.4567830279 -3.8059048844 1.8552233615
H -1.4265083399 -3.0718895345 1.2042762985

References for Computational and Correlative Studies:

1. M. J. Frisch, G. W. Trucks, H. B. Schlegel, G. E. Scuseria, M. A. Robb, J. R. Cheeseman, G. Scalmani, V. Barone, B. Mennucci, G. A. Petersson, H. Nakatsuji, M. Caricato, X. Li, H. P. Hratchian, A. F. Izmaylov, J. Bloino, G. Zheng, J. L. Sonnenberg, M. Hada, M. Ehara, K. Toyota, R. Fukuda, J. Hasegawa, M. Ishida, T. Nakajima, Y. Honda, O. Kitao, H. Nakai, T. Vreven, J. A. Montgomery, Jr., J. E. Peralta, F. Ogliaro, M. Bearpark, J. J. Heyd, E. Brothers, K. N. Kudin, V. N. Staroverov, R. Kobayashi, J. Normand, K. Raghavachari, A. Rendell, J. C. Burant, S. S. Iyengar, J. Tomasi, M. Cossi, N. Rega, J. M. Millam, M. Klene, J. E. Knox, J. B. Cross, V. Bakken, C. Adamo, J. Jaramillo, R. Gomperts, R. E. Stratmann, O. Yazyev, A. J. Austin, R. Cammi, C. Pomelli, J. W. Ochterski, R. L. Martin, K. Morokuma, V. G. Zakrzewski, G. A. Voth, P. Salvador, J. J. Dannenberg, S. Dapprich, A. D. Daniels, Ö. Farkas, J. B. Foresman, J. V. Ortiz, J. Cioslowski, and D. J. Fox, Gaussian, Inc., Wallingford CT, 2009.
2. (a) R. Valero, J. R. Gomes, D. G. Truhlar and F. Illas, *J. Chem. Phys.,* 2008, **129**, 124710. (b) Y.

Zhao and D. G. Truhlar, *Theor. Chem. Account,* 2008, **120**, 215.
3. J. A. Quinn, ChemSW, Inc.: Fairfield , CA.
4. E. D. Glendening, J, K. Badenhoop, A. E. Reed, J. E. Carpenter, J. A. Bohmann, C. M. Morales, C. R. Landis, and F. Weinhold, Theoretical Chemistry Institute, University of Wisconsin, Madison (2013).
5. In MATLAB NBO Version, 3.1 *(R2014a)*; The MathWorks, Inc.: Natick, MA, 2014.
6. S. Arlot and A. Celisse, *Statist. Surv.* 2010, **4**, 40.
7. C. Hansch, A. Leo and R. W. Taft, *Chem. Rev.*, 1991, **91**, 165.
8. Y. Zhao and D. G. Truhlar, *Theor. Chem. Acc*. 2008, **120**, 215.
9. (a) M. J. Hilton, L.-P. Xu, P.-O. Norrby, Y.-D. Wu, O. Wiest and M. S. Sigman, *J. Org. Chem.*, 2014, **79**, 11841. (b) L. Xu, M. J. Hilton, X. Zhang, P.-O. Norrby, Y.-D. Wu, M. S. Sigman and O. Wiest, *J. Am. Chem. Soc.*, 2014, **136**, 1960

Experimental section

General Considerations:

All manipulations were conducted using Schlenk techniques. ^{1}H NMR spectra were obtained at 400 MHz or 500 MHz, chemical shifts are reported in ppm, and referenced to the tetramethylsilane (δ = 0 ppm). ^{13}C NMR spectra were obtained at 100 MHz or 125 MHz and referenced to $CDCl_3$ (δ = 77.00 ppm). IR spectra were recorded using a Thermo Nicolet FT-IR. High resolution mass spectrometry (HRMS) data were obtained on a Waters LCP Premier XE instrument by ESI/TOF. SFC (supercritical fluid chromatography) analysis was performed at 40 ºC, using a Thar instrument fitted with a S2 chiral stationary phase as indicated. HPLC analysis was performed at room temperature. Optical rotations were measured (Na D line) on a Perkin Elmer Model 343 Polarimeter fitted with a micro cell with a 1 dm path length; concentrations are reported in g/100 mL. Dry ethyl pivalate was purchased from Alfa Aesar, distilled to remove any stabilizer, and stored over activated 3 Å molecular sieves (3 Å MS). Powdered 3 Å MS were activated by flowing N_2 through a glass tube of sieves maintained at 200 °C. Substrates were purchased from Aldrich, TCI, or Acros, or synthesized according to the procedures outlined below.

Synthesis of alkene substrates

These substrates were prepared according to the corresponding literature reports. Analytical data (^{1}H NMR, ^{13}C NMR) matches with the literature.

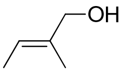

2a: (*E*)-2-methylbut-2-en-1-ol[1]

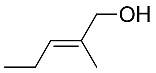

2b: (*E*)-2-methylpent-2-en-1-ol[2]

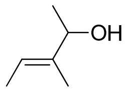

2d: (*E*)-3-methylpent-3-en-2-ol[3]

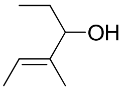

2e: (*E*)-4-methylhex-4-en-3-ol[4]

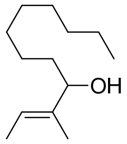

2f: (*E*)-3-methyldodec-2-en-4-ol[5]

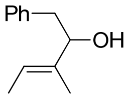

2h: (*E*)-3-methyl-1-phenylpent-3-en-2-ol[6]

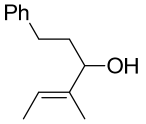

2g: (*E*)-4-methyl-1-phenylhex-4-en-3-ol[7]

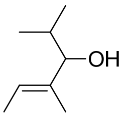

2j: (*E*)-2,4-dimethylhex-4-en-3-ol[8]

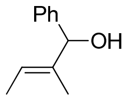

2i: (*E*)-2-methyl-1-phenylbut-2-en-1-ol[9]

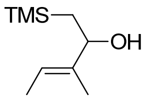

2k: (*E*)-3-methyl-1-(trimethylsilyl)pent-3-en-2-ol[10]

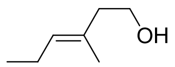

2z: (*E*)-3-methylhex-3-en-1-ol[11]

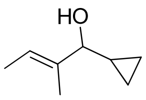

2l: (*E*)-1-cyclopropyl-2-methylbut-2-en-1-ol[12]

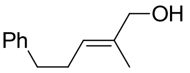

2b: (*E*)-2-methyl-5-phenylpent-2-en-1-ol[13]

Synthesis of Alkenyl Triflates

The alkenyl triflates shown below were prepared according to the corresponding literature reports. Analytical data (^{1}H NMR, ^{13}C NMR) matches with the literature.

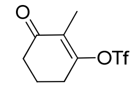

1a: 2-methyl-3-oxocyclohex-1-en-1-yl trifluoromethanesulfonate[14]

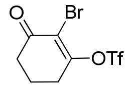

1p: 2-bromo-3-oxocyclohex-1-en-1-yl trifluoromethanesulfonate[15]

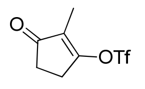

1q: 2-methyl-3-oxocyclopent-1-en-1-yl trifluoromethanesulfonate[16]

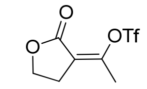

1r: (*Z*)-1-(2-oxodihydrofuran-3(2*H*)-ylidene)ethyl trifluoromethanesulfonate[17]

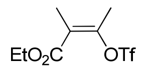

1s: (*Z*)-ethyl 2-methyl-3-(((trifluoromethyl)sulfonyl)oxy)but-2-enoate[18]

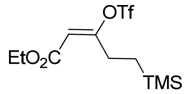

1u: (*E*)-ethyl 3-(((trifluoromethyl)sulfonyl)oxy)-5-(trimethylsilyl)pent-2-enoate[19]

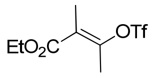

1t: (*E*)-ethyl 2-methyl-3-(((trifluoromethyl)sulfonyl)oxy)but-2-enoate[18]

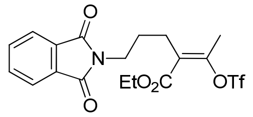

1v: (*Z*)-ethyl 5-(1,3-dioxoisoindolin-2-yl)-2-(1-(((trifluoromethyl)sulfonyl)oxy)ethylidene)pentanoate[19]

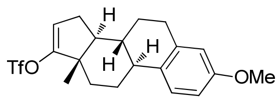

1x: (8*R*,9*S*,13*S*,14*S*)-3-methoxy-13-methyl-7,8,9,11,12,13,14,15-octahydro-6*H*-cyclopenta[*a*]phenanthren-17-yl trifluoromethanesulfonate[19]

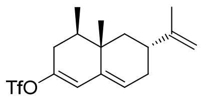

1w: (4*R*,4a*S*,6*R*)-4,4a-dimethyl-6-(prop-1-en-2-yl)-3,4,4a,5,6,7-hexahydron-aphthalen-2-yl trifluoromethanesulfonate[19]

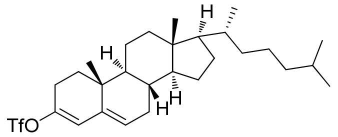

1y: (8*S*,9*S*,10*R*,13*R*,14*S*,17*R*)-10,13-dimethyl-17-((*R*)-6-methylheptan-2-yl)-2,7,8,9,10,11,12,13,14,15,16,17-dodecahydro-1*H*-cyclopenta[*a*]phenanthren-3-yl trifluoromethanesulfonate[19]

General Procedure for Alkenyl Triflate Synthesis:

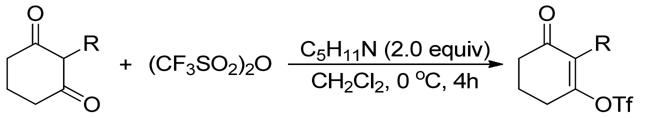

To a dry 100 mL Schlenk flask equipped with a stir bar was added the 1,3-dione substrate (2 mmol, 1.0 equiv), piperidine (4 mmol, 2.0 equiv), and CH_2Cl_2 (25 mL). The mixture was stirred for 10 min at 0 °C under an atmosphere of nitrogen. Then trifluoromethanesulfonic anhydride (2.2 mol, 2.2 equiv) was added dropwise. After the reaction was stirred for 4 hours at 0 °C, it was quenched by the addition of HCl (25 mL, 1 M). The aqueous layer was extracted with diethyl ether (2 x 50 mL), and the combined organic layers were washed with water (20 mL), and brine (20 mL). The organic layer was then dried over Na_2SO_4, and concentrated under reduced pressure. The resulting mixture was purified using silica gel flash chromatography (10–20% EtOAc/hexanes) to give the alkenyl triflate product.

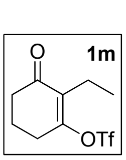

2-ethyl-3-oxocyclohex-1-en-1-yl trifluoromethanesulfonate (**1m**): This compound was prepared using the general procedure for alkenyl triflate synthesis using 2-ethylcyclohexane-1,3-dione (280 mg, 2.0 mmol). Purification of this material by chromatography on silica gel (1:10 EtOAc:hexanes) afforded alkenyl triflate **1m** as a yellow liquid (408 mg, 75% yield): R_f = 0.50 (1:10 EtOAc:hexanes); **^{1}H NMR** ($CDCl_3$, 500 MHz) δ = 2.75 (t, *J* = 5.0 Hz, 2H), 2.47 (d, *J* = 5.0 Hz, 2H), 2.38-2.35 (m, 2H), 2.10-2.04 (m, 2H), 1.02 (t, *J* = 6.0 Hz, 3H); **^{13}C NMR** ($CDCl_3$, 125 MHz) δ = 197.3, 161.5, 133.5, 119.5, 117.0, 36.9, 28.7, 20.6, 17.2, 12.8 ppm; **IR (neat)** 2943, 1687, 1413, 1206, 1136, 923 cm^{-1}; **HRMS** (ESI-TOF) *m/z* calcd for $C_9H_{12}O_4SF_3$ $(M + H)^+$: 273.0408, found 273.0407.

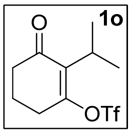

2-isopropyl-3-oxocyclohex-1-en-1-yl trifluoromethanesulfonate (**1o**): This compound was prepared using the general procedure for alkenyl triflate synthesis using 2-isopropylcyclohexane-1,3-dione (308 mg, 2.0 mmol). Purification of this material by chromatography on silica gel (1:10 EtOAc:hexanes) afforded alkenyl triflate **1o** as a yellow liquid (303 mg, 53% yield): R_f = 0.50 (1:10 EtOAc:hexanes); **^{1}H NMR** ($CDCl_3$, 500 MHz) δ 3.09-3.01 (m, 1H), 2.73 (d, *J* = 5.0 Hz, 2H), 2.44 (t, *J* = 6.0 Hz, 2H), 2.07-2.01 (m, 2H), 1.21 (d, *J* = 5.0 Hz, 6H); **^{13}C NMR** ($CDCl_3$, 125 MHz) δ 197.4, 160.8, 136.3, 119.5, 117.0, 38.0, 29.0, 26.1, 20.5, 20.1 ppm; **IR** (neat) 2940, 1689, 1136, 1036, 866, 598 cm^{-1}; **HRMS** (ESI-TOF) *m/z* calcd for $C_{10}H_{14}O_4SF_3$ $(M + H)^+$: 287.0565, found 287.0569.

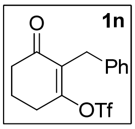

2-benzyl-3-oxocyclohex-1-en-1-yl trifluoromethanesulfonate (**1n**): This compound was prepared using the general procedure for alkenyl triflate synthesis using 2-benzylcyclohexane-1,3-dione (404 mg, 2.0 mmol). Purification of this material by chromatography on silica gel (1:10 EtOAc:hexanes) afforded alkenyl triflate **1n** as a yellow liquid (381 mg, 57% yield), R_f = 0.5 (1:10 EtOAc:hexanes); **^{1}H NMR** ($CDCl_3$, 500 MHz) δ 7.27-7.23 (m, 2H), 7.21-7.17 (m, 3H), 3.70 (s, 2H), 2.81 (t, *J* = 5.0 Hz, 2H), 2.48 (t, *J* = 6.0 Hz, 2H), 2.10-2.04 (m, 2H) ; **^{13}C NMR** ($CDCl_3$, 125 MHz) δ 196.9, 162.5, 137.9, 130.9, 128.6, 128.5, 126.5, 119.5, 117.0, 36.8, 29.1, 28.7, 20.5 ppm; **IR** (neat) 2960, 1686, 1414, 1205, 1134, 788 cm^{-1}; **HRMS** (ESI-TOF) *m/z* calcd for $C_{14}H_{14}O_4SF_3$ $(M + H)^+$ 335.0565, found 335.0563.

Ligand Synthesis

The ligands shown below were prepared according to the corresponding literature. Analytical data (^{1}H NMR, ^{13}C NMR) matches with the literature.

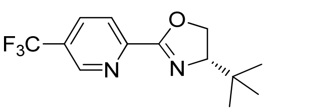

(*S*)-4-(*tert*-butyl)-2-(5-(trifluoromethyl) pyridin-2-yl)-4,5-dihydrooxazole[20]

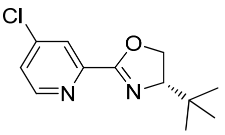

(*S*)-4-(*tert*-butyl)-2-(4-chloropyridin -2-yl)-4,5-dihydrooxazole[20]

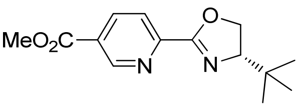

(*S*)-methyl 6-(4-(*tert*-butyl)-4,5 -dihydrooxazol-2-yl)nicotinate[21]

General Procedure for Ligand Synthesis:

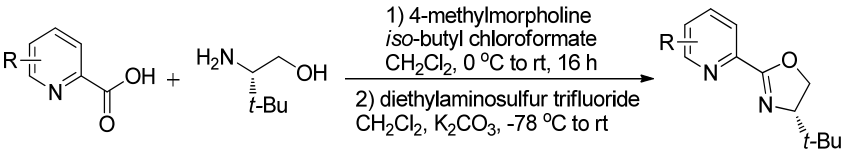

To a dry 50 mL round-bottom flask containing a stir bar was added picolinic acid substrate (1.0 mmol, 1.0 equiv). Under an N_2 atmosphere, CH_2Cl_2 (20 mL) and 4-methylmorpholine (121 mg, 1.2 mmol, 1.2 equiv) were added. Then, the reaction mixture was cooled to 0 °C, *iso*-butylchloroformate was added (1.2 mmol, 1.2 equiv) via syringe. The reaction mixture was stirred for another 30 min, then (*S*)-2-amino-3,3-dimethylbutan-1-ol (129 mg, 1.1 mmol, 1.1 equiv) was added. The mixture was allowed to slowly warm to room temperature and stirred for 16 h. The reaction was quenched via addition of water (10 mL). The aqueous layer was extracted with CH_2Cl_2 (2 x 20 mL), and the combined organic layers were washed with brine (1 x 20 mL), dried over Na_2SO_4, decanted, and concentrated under reduced pressure to give a brown solid. Under an N_2 atmosphere, CH_2Cl_2 (35 mL) was added and the reaction mixture was cooled to –78 °C, and diethylaminosulfur trifluoride (2.0 mmol, 2.0 equiv) was added dropwise. The reaction mixture was warmed up to 0 °C and stirred for 1 h. Then, potassium carbonate (2.0 mmol, 2.0 equiv) was added. The mixture was allowed to warm to room temperature. The reaction was quenched via addition of water (2 mL). The aqueous layer was extracted with CH_2Cl_2 (2 x 20 mL), the combined organic layers were washed with brine (1 x 20 mL), dried over Na_2SO_4, decanted, and concentrated under reduced pressure to give a brown solid. Purification of this material by chromatography on silica gel afforded the corresponding product.

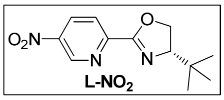

(*S*)-4-(tert-butyl)-2-(5-nitropyridin-2-yl)-4,5-dihydrooxazole (**L-NO$_2$**): This ligand was prepared using the general procedure for ligand synthesis using 5-nitropicolinic acid (168 mg, 1.0 mmol). Purification of this material by chromatography on silica gel (1:1 EtOAc:hexanes) afforded ligand **L-NO$_2$** as a white solid (132 mg, 53% yield): R_f = 0.20 (1:1 EtOAc:hexanes); **^{1}H NMR** ($CDCl_3$, 500 MHz) δ 9.49 (t, *J* = 5.0 Hz, 1H), 8.55 (t, *J* = 10.0 Hz, 1H), 8.29 (t, *J* = 7.5 Hz, 1H), 4.51 (t, *J* = 7.5 Hz, 1H), 4.37 (t, *J* = 10.0 Hz, 1H), 4.19 (t, *J* = 10.0 Hz, 1H), 0.99 (s, 9H); **^{13}C NMR** ($CDCl_3$, 125 MHz) δ 161.0, 151.5, 145.0, 131.8, 124.3, 76.9, 69.9, 34.1, 25.9 ppm; **IR** (neat) 2960, 1653, 1559, 1524, 1507, 668 cm^{-1};

$[\alpha]_D^{20}$ = –68 (c = 0.1, EtOH); **HRMS** (ESI-TOF) *m/z* calcd for $C_{12}H_{16}N_3O_3$ $(M + H)^+$: 250.1192, found 250.1198.

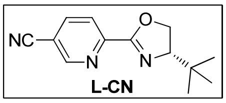

(*S*)-6-(4-(tert-butyl)-4,5-dihydrooxazol-2-yl)nicotinonitrile (**L-CN**): This ligand was prepared using the general procedure for ligand synthesis using 5-cyanopicolinic acid (148 mg, 1.0 mmol). Purification of this material by chromatography on silica gel (1:1 EtOAc:hexanes) afforded ligand **L-CN** as a white solid (131 mg, 57% yield): R_f = 0.20 (1:1 EtOAc:hexanes); **^{1}H NMR** ($CDCl_3$, 400 MHz) δ 8.97 (d, *J* = 4.0 Hz, 1H), 8.22 (d, *J* = 8.0 Hz, 1H), 8.06 (d, *J* = 4.0 Hz, 1H), 4.50 (t, *J* = 10.0 Hz, 1H), 4.35 (t, *J* = 8.0 Hz, 1H), 4.17 (t, *J* = 8.0 Hz, 1H), 0.98 (s, 9H); **^{13}C NMR** ($CDCl_3$, 100 MHz) δ 161.1, 152.2, 149.7, 139.8, 123.7, 116.1, 111.3, 76.8, 69.7, 34.0, 25.9 ppm; **IR** (neat) 2955, 2871, 1635, 1473, 1095, 949 cm^{-1}; $[\alpha]_D^{20}$ = –58 (c = 0.1, EtOH); **HRMS** (ESI-TOF) *m/z* calcd for $C_{13}H_{15}N_3ONa$ $(M + Na)^+$: 252.1113, found 252.1120.

General Procedure A for the Enantioselective Redox-Relay Heck Reaction

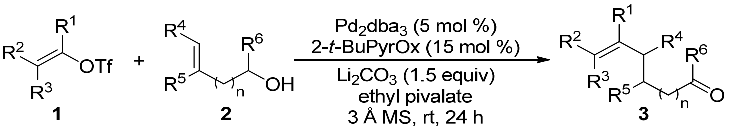

To a dry 100 mL Schlenk flask equipped with a stir bar was added Pd_2dba_3 (11.1 mg, 0.0125 mmol, 0.050 equiv) ligand (0.0375 mmol, 0.15 equiv), 3Å MS (50 mg, 200 mg/mmol **1**), and ethyl pivalate (3 mL). To this flask, a three-way adapter fitted with a balloon of N_2 was added, and the flask was evacuated via house vacuum and refilled with N_2 three times while stirring. The resulting mixture was stirred for 15 min. To this, the corresponding alkenyl alcohol (**2**, 0.50 mmol, 2.0 equiv), alkenyl triflate (**1**, 0.25 mmol, 1.0 equiv) and Li_2CO_3 (28 mg, 0.375 mmol, 1.5 equiv) were added. The resulting mixture was stirred for another 24 h at room temperature. The solvent was concentrated under reduced pressure, and the resulting residue was purified by chromatography on silica gel to afford the corresponding product (**3**).

General Procedure B for the Reduction of Aldehydes

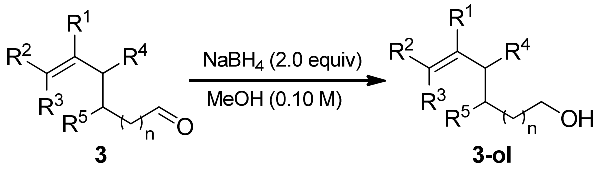

The aldehyde product was dissolved in MeOH (0.1 M) in a 20 mL scintillation vial equipped with a stir bar. The mixture was cooled to 0 °C and sodium borohydride (2.0 equiv) was added, and the resulting mixture was stirred for 30 min. The solvent was removed under reduced pressure, and the resulting residue was transferred to a separatory funnel using diethyl ether (100 mL) and water (20 mL). The aqueous layer was extracted with diethyl ether (2 x 50 mL), and the combined organic layers were washed with water (20 mL), and brine (20 mL). The organic layer was then dried over Na_2SO_4, decanted, and concentrated under reduced pressure. The resulting residue was purified by chromatography on silica gel to afford the corresponding alcohol product (**3-ol**).

General Procedure C for Esterification

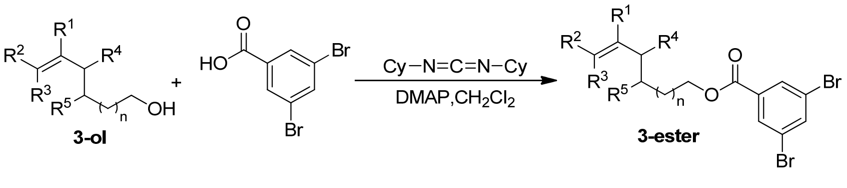

To a mixture of the alcohol (**3-ol**, 0.10 mmol), DMAP (6.1 mg, 0.050 mmol), and *3,5*-dibromobenzoic acid (280 mg, 1.0 mmol) in CH_2Cl_2 (5 mL) was added *N,N'*-dicyclohexylcarbodiimide (206 mg, 1.0 mmol) at 0 °C. The mixture was warmed to ambient temperature and stirred for 2 h. After this time, the reaction was filtered through a short plug of silica gel with CH_2Cl_2, concentrated under reduced pressure. The resulting residue was purified by chromatography on silica gel to afford the corresponding ester product (**3-ester**).

Matched and Mismatched Ligand Effects with Chiral Triflate Reagents

Table S13. The result of Late-Stage Functionalization.[a]

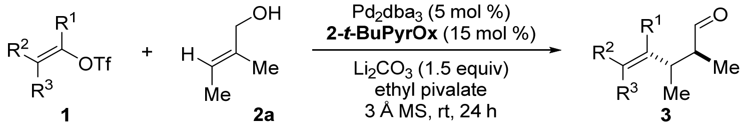

	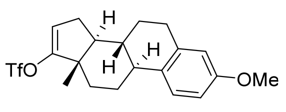	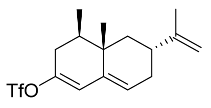	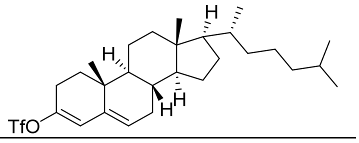
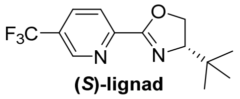 **(*S*)-lignad**	79% yield<br>dr = 1:1.8	27% yield<br>dr = 1:2.3	22% yield<br>dr = 1:2.4
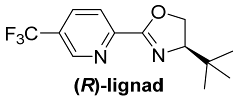 **(*R*)-lignad**	79% yield<br>dr > 20:1	31% yield<br>dr = 1:7.9	26% yield)<br>dr = 1:7.9

[a]General Procedure A was followed. Each entry represents the isolated yield on 0.25 mmol scale. dr values were determined by ^{1}H NMR.

Unsuccessful Reactions with Sterically Hindered Alkenols

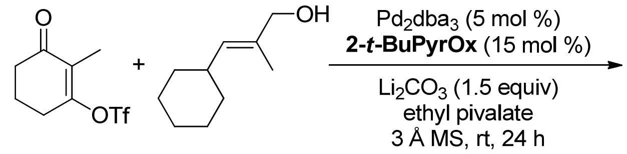

(Eq. S3)

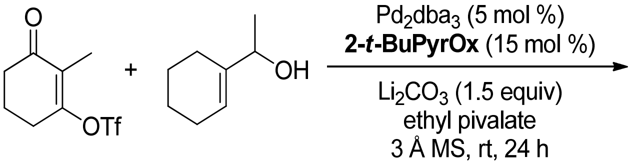

(Eq. S4)

Data for X-ray Analysis

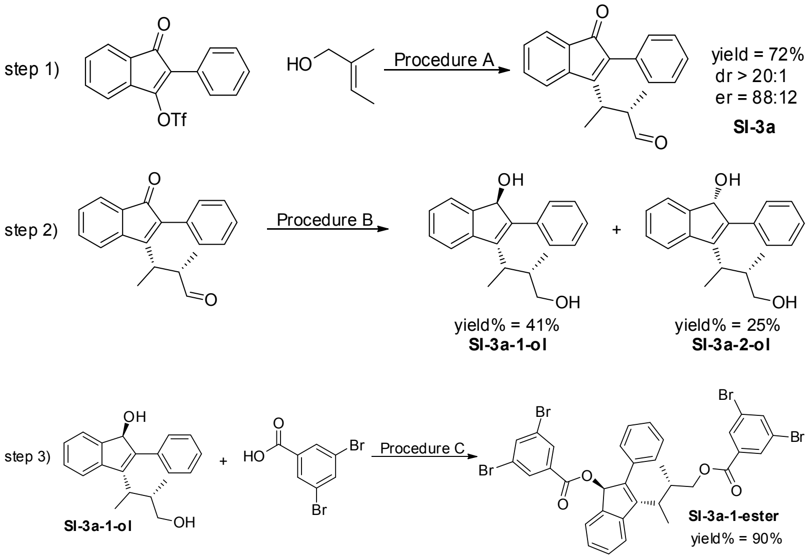

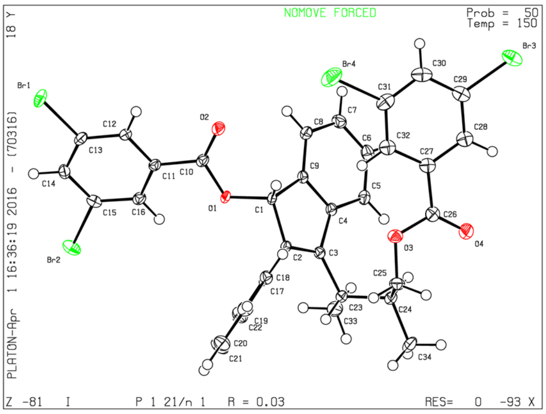

Figure S7. Single diastereomer of **SI-3a-1-ester**

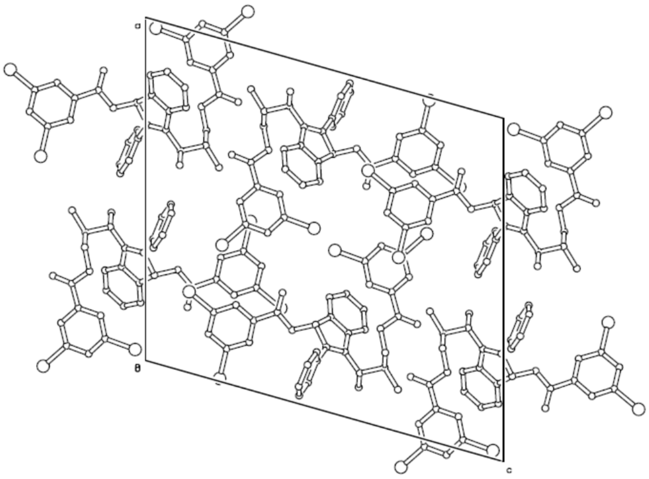

Figure S8. Crystal cell of **SI-3a-1-ester**

ECD analysis

1. ECD Methodology

The sample was dissolved in CH_3CN (0.15 mg/mL). All experiments were performed using a 0.10-mm path length quartz Starna cell. The ECD spectra were recorded using a Jasco J-810 CD spectropolarimeter (Jasco, Easton, MD), with the following instrumental parameters: 185-400 nm wavelength scan, 5 nm bandwidth, high sensitivity, 1 sec response, 1 nm data pitch, 200 nm/min scanning speed, 3 accumulations. The acetonitrile blank was collected first, and used to baseline correct the sample.

A subset of the details of the computational methodology is provided here. The applied conformational sampling workflow is analogous to what has been used previously for VCD.[22] Conformers of each test structure were geometry optimized at the B3LYP/6-31G** level and stationary points were confirmed by performing frequency calculations.[23-31] All calculations were performed using Gaussian 09.[32]

Output conformers were ranked according to DFT energy and a clustering was performed to remove duplicates. Initial identification of duplicates was performed solely on an electronic energy basis where compounds were considered identical if the difference in Hartrees was less than 0.01. Rounding the differences led to inconsistencies in identification of duplicates. It became better to cluster the DFT minima by energy and then re-cluster each energy bucket by structure using an all atom RMS of 0.6 Å. This faithfully removed only identical compounds.

To calculate ECD spectra, B3LYP geometries were used as input for CAM-B3LYP[12] calculations using the 6-31++G** basis set[33-35] *in vacuo*. Only conformers which contributed more than 2.0% to the total *in vacuo* conformer distribution were selected for ECD calculation. Time-dependent Density Functional Theory (TDDFT)[36] methodology was employed using the following keywords: TD=full,singlet, Nstates=100, and integral=ultrafinegrid. Spectral display and Boltzmann weighting were carried out using SpecDis,[37-38] and were displayed with a band broadening sigma=0.3 eV.

Figures S1 and S2 provide the overlays of the calculated and measured ECD spectra. A high degree of confidence is derived from the statistical and visual matching of the spectra. The final assignment is shown in Scheme S1.

2. Overlay of ECD Spectra

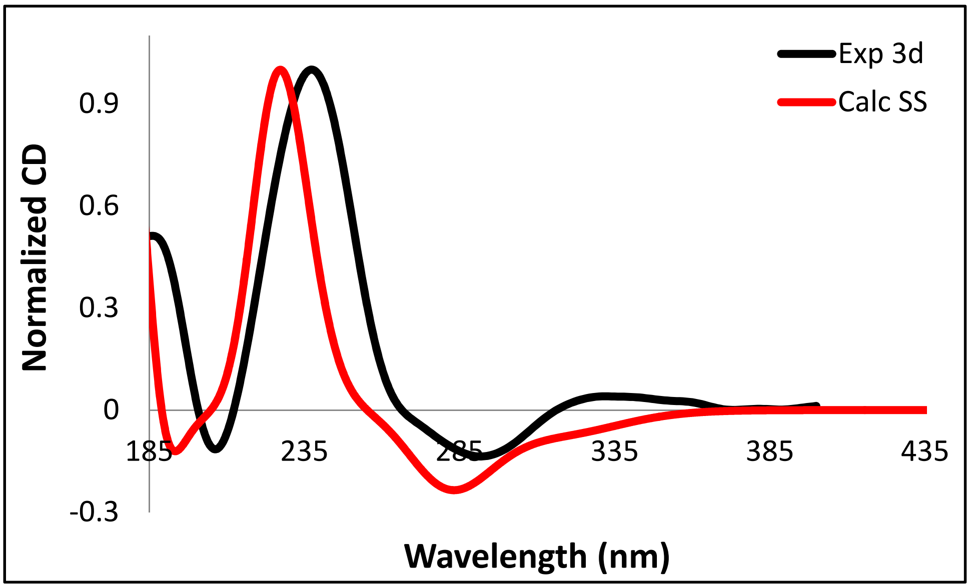

Figure S9: Overlay of unshifted measured (black) and calculated (red) ECD spectra.

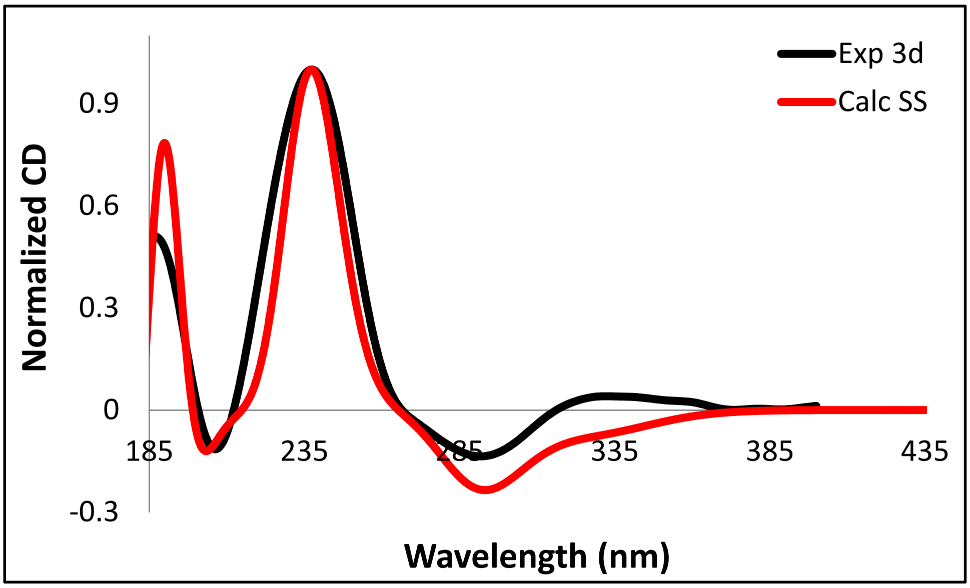

Figure S10: Overlay of shifted measured (black) and calculated (red) ECD spectra. The entire calculated spectrum was shifted by +10 nm to account for a systematic overestimation of transition energies.

3. Assigned Absolute Configuration

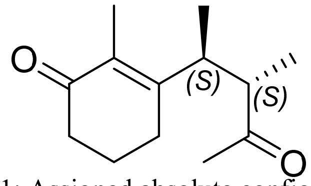

Scheme S1: Assigned absolute configuration *SS* for compound **3d**.

4.   Conformations of Global and Local Minima

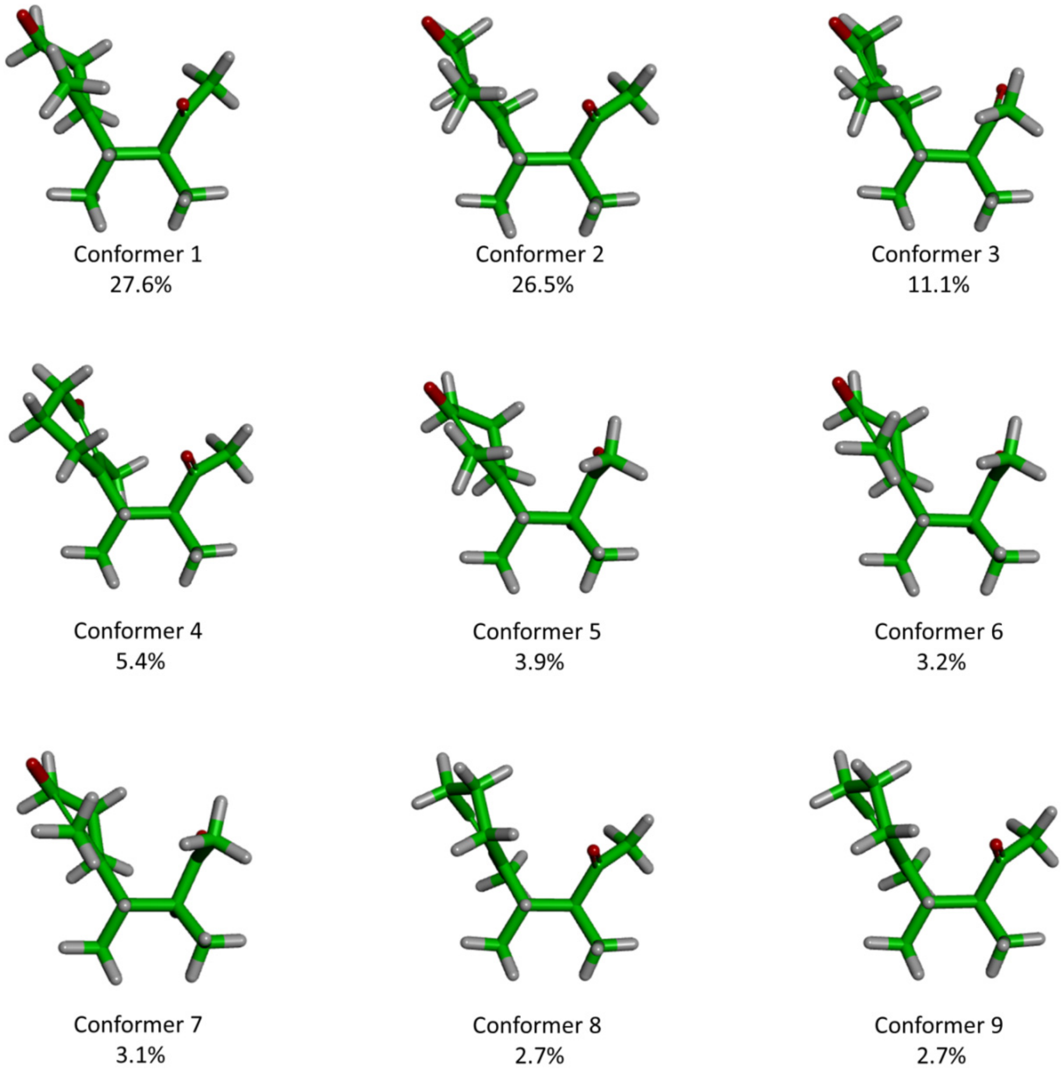

Figure S11: Conformers of the *SS* stereoisomer minima contributing >2% to the Boltzmann distribution, percentages shown above based on *in vacuo* electronic energies.

5.   Electronic Energies and Coordinates for Minima

Provided for conformers that contribute >2% to the in vacuo Boltzmann distribution.

Conformer 1:   E = -657.905771

B3LYP/6-31G**

6	-2.67405	0.439878	0.23094
6	-3.10567	-0.52206	-0.86746
6	-1.93942	-0.93867	-1.75985
6	-0.80458	-1.4961	-0.89898
6	-0.40791	-0.56841	0.235885
6	-1.27388	0.344042	0.750892

8	-3.47521	1.24127	0.69394
6	0.990947	-0.76699	0.813949
6	2.10753	-0.36772	-0.19742
6	1.15695	-2.21334	1.32679
6	3.52743	-0.55151	0.37703
6	1.96675	1.10935	-0.59807
8	1.92231	1.98257	0.249715
6	1.93632	1.43365	-2.07778
6	-0.94384	1.28621	1.88789
1	1.1848	-2.94033	0.507946
1	2.07779	-2.325	1.90443
1	0.323354	-2.48108	1.98359
1	3.74156	-1.60143	0.589809
1	4.28424	-0.2004	-0.33274
1	3.64039	0.02786	1.29797
1	2.79826	0.987698	-2.58787
1	1.03935	0.997838	-2.53422
1	1.9329	2.51427	-2.22552
1	-0.84141	0.746083	2.83712
1	-0.01273	1.82832	1.70722
1	-1.75857	2.00153	2.00383
1	-3.92198	-0.05169	-1.42223
1	-3.52825	-1.40956	-0.37244
1	-1.57788	-0.06574	-2.31894
1	-2.25906	-1.68042	-2.50004
1	0.067028	-1.72153	-1.52412
1	-1.11344	-2.46437	-0.47519
1	1.10619	-0.09522	1.66743
1	2.02126	-0.98756	-1.09827

Conformer 2: E = -657.905732
B3LYP/6-31G**

6	2.67536	-0.54368	0.120623
6	2.95807	0.086164	-1.23663
6	2.21363	1.40621	-1.42458
6	0.718872	1.19681	-1.17532
6	0.429772	0.507137	0.14601
6	1.34557	-0.28977	0.755272
8	3.51301	-1.2618	0.651218
6	-0.93777	0.771969	0.768324
6	-2.09496	0.382008	-0.19213
6	-1.03122	2.23383	1.2567
6	-3.49079	0.707851	0.383787
6	-2.07402	-1.12726	-0.48098

8	-1.84252	-1.93588	0.399056
6	-2.4051	-1.56542	-1.89503
6	1.10749	-0.99616	2.07184
1	-1.92387	2.3898	1.86797
1	-0.16221	2.48184	1.87354
1	-1.06664	2.94927	0.427784
1	-4.2848	0.335004	-0.27206
1	-3.63531	1.78553	0.490436
1	-3.61929	0.238151	1.36392
1	-1.58706	-1.27786	-2.56722
1	-2.53585	-2.64766	-1.93116
1	-3.30681	-1.06354	-2.26288
1	0.24124	-1.66185	2.0085
1	0.927021	-0.28798	2.88834
1	1.98858	-1.58703	2.3236
1	4.04179	0.189138	-1.33816
1	2.6343	-0.63481	-2.00275
1	2.59716	2.14831	-0.71299
1	2.38227	1.81022	-2.42896
1	0.191067	2.15595	-1.21864
1	0.299802	0.59457	-1.99766
1	-1.04534	0.124261	1.64045
1	-1.98929	0.921945	-1.14161

Conformer 3: E = -657.904910
B3LYP/6-31G**

6	2.64784	-0.55027	0.094886
6	2.92287	0.296242	-1.1377
6	2.18969	1.63519	-1.07949
6	0.692138	1.3985	-0.87724
6	0.389385	0.457158	0.271928
6	1.30926	-0.42106	0.749085
8	3.48777	-1.33862	0.510882
6	-1.00422	0.557961	0.885759
6	-2.136	0.463293	-0.18842
6	-1.12015	1.84353	1.73528
6	-3.54349	0.500147	0.435357
6	-1.95118	-0.78445	-1.06465
8	-1.53571	-0.68754	-2.20615
6	-2.31807	-2.14047	-0.48539
6	1.07897	-1.34735	1.92443
1	-1.08914	2.74473	1.11436
1	-2.0516	1.86244	2.30699
1	-0.2922	1.89996	2.44821

1	-3.74967	1.47602	0.88116
1	-4.31113	0.323913	-0.32523
1	-3.66378	-0.25697	1.21764
1	-1.95511	-2.26342	0.539677
1	-3.40889	-2.24531	-0.45306
1	-1.90984	-2.92483	-1.12369
1	0.69758	-0.81765	2.80275
1	0.3675	-2.14662	1.68411
1	2.02515	-1.8217	2.18755
1	2.57724	-0.27742	-2.01069
1	4.0064	0.405228	-1.23482
1	2.3628	2.21248	-1.99406
1	2.58321	2.23142	-0.2461
1	0.250893	0.980253	-1.79315
1	0.179695	2.3541	-0.71339
1	-1.14016	-0.28708	1.56783
1	-2.03759	1.30915	-0.87697

Conformer 4: E = -657.904229

B3LYP/6-31G**

6	2.46811	-0.65199	-0.42721
6	3.02899	-0.29218	0.938867
6	2.59198	1.10746	1.36486
6	1.06846	1.21371	1.32147
6	0.439142	0.688017	0.046708
6	1.11962	-0.12692	-0.80158
8	3.08261	-1.39562	-1.18262
6	-1.00224	1.17393	-0.14472
6	-2.02891	0.028464	-0.34044
6	-1.0841	2.28091	-1.21684
6	-3.48549	0.544499	-0.37223
6	-1.93038	-0.98419	0.810047
8	-1.67975	-0.63033	1.94889
6	-2.19184	-2.43888	0.471548
6	0.635124	-0.63835	-2.13955
1	-0.91656	1.89723	-2.22714
1	-2.05891	2.77631	-1.20091
1	-0.32536	3.04675	-1.02758
1	-3.66281	1.17688	-1.24561
1	-4.1991	-0.28458	-0.41999
1	-3.70706	1.12511	0.529055
1	-3.07301	-2.55096	-0.16896
1	-1.33692	-2.83145	-0.09288
1	-2.3136	-3.02106	1.38596

1	-0.18117	-0.04885	-2.55401
1	1.46777	-0.63149	-2.84694
1	0.309326	-1.68411	-2.07579
1	4.11521	-0.40908	0.898246
1	2.6514	-1.03542	1.65704
1	3.02844	1.84676	0.680767
1	2.96274	1.34534	2.36767
1	0.758215	2.25878	1.45418
1	0.612749	0.669123	2.16222
1	-1.28365	1.63698	0.807777
1	-1.84135	-0.50148	-1.27891

Conformer 5: E = -657.903923

B3LYP/6-31G**

6	2.68218	0.255539	0.386178
6	3.0344	-0.93944	-0.48526
6	1.82241	-1.82139	-0.7754
6	0.685291	-0.97999	-1.35739
6	0.370454	0.244006	-0.51701
6	1.29637	0.813667	0.298206
8	3.52866	0.764538	1.11055
6	-1.01702	0.854593	-0.68882
6	-2.1838	-0.1514	-0.40474
6	-1.14696	1.4666	-2.10196
6	-3.54456	0.560629	-0.29957
6	-1.89327	-0.99655	0.842762
8	-1.53446	-2.15616	0.735075
6	-2.0836	-0.36485	2.2112
6	1.05506	2.05118	1.13793
1	-1.11687	0.692427	-2.87539
1	-2.08414	2.01808	-2.21483
1	-0.32638	2.16501	-2.29221
1	-3.81578	1.03448	-1.24621
1	-4.33963	-0.15123	-0.05477
1	-3.53791	1.33749	0.472144
1	-1.72897	0.669452	2.24473
1	-3.15201	-0.34542	2.45756
1	-1.56587	-0.96524	2.96018
1	0.763195	2.91237	0.527074
1	0.271682	1.89898	1.88813
1	1.977	2.30274	1.66316
1	3.85056	-1.47962	0.002354
1	3.43857	-0.5394	-1.4279
1	1.47193	-2.29187	0.150771

1	2.08712	-2.63068	-1.46439
1	-0.20797	-1.60101	-1.46023
1	0.951748	-0.64997	-2.37399
1	-1.12018	1.67293	0.029724
1	-2.23141	-0.87222	-1.22768

Conformer 6: E = -657.903741
B3LYP/6-31G**

6	2.64619	0.436856	0.082416
6	3.00627	-1.02989	0.251453
6	1.78901	-1.87828	0.611122
6	0.671611	-1.64642	-0.40665
6	0.33857	-0.17558	-0.60005
6	1.26364	0.792723	-0.36482
8	3.47843	1.31376	0.278171
6	-1.06701	0.120013	-1.11487
6	-2.19098	-0.37029	-0.13727
6	-1.26023	-0.48268	-2.52342
6	-3.55821	0.246872	-0.48205
6	-1.80786	-0.11123	1.32596
8	-1.46017	-1.03247	2.04408
6	-1.88546	1.30817	1.86061
6	1.07375	2.2825	-0.52918
1	-2.22572	-0.19629	-2.94889
1	-0.47985	-0.12522	-3.2021
1	-1.21468	-1.57588	-2.50149
1	-4.32555	-0.08257	0.226066
1	-3.88722	-0.05598	-1.47944
1	-3.52619	1.34123	-0.4604
1	-2.93323	1.57992	2.03547
1	-1.34801	1.36401	2.80796
1	-1.47676	2.03652	1.15402
1	1.2405	2.79265	0.425144
1	0.089439	2.56229	-0.9042
1	1.82933	2.68127	-1.21296
1	3.42913	-1.37121	-0.70595
1	3.80949	-1.09447	0.990774
1	2.05285	-2.94067	0.64964
1	1.42019	-1.60303	1.60604
1	0.963892	-2.07207	-1.37948
1	-0.22338	-2.19001	-0.09289
1	-1.18464	1.20213	-1.20977
1	-2.27145	-1.45963	-0.2151

Conformer 7: E = -657.903718

B3LYP/6-31G**

6	-2.6482	-0.44999	-0.01133
6	-3.01338	0.945885	0.465643
6	-1.79952	1.70234	0.999406
6	-0.68129	1.69608	-0.04339
6	-0.34259	0.301489	-0.54551
6	-1.26443	-0.69757	-0.52359
8	-3.47751	-1.35116	-0.00741
6	1.06365	0.127824	-1.11066
6	2.18622	0.401598	-0.05038
6	1.2528	1.01924	-2.3576
6	3.56085	-0.09836	-0.52951
6	1.81329	-0.18718	1.31744
8	1.43622	0.540911	2.21903
6	1.93737	-1.68647	1.52408
6	-1.07029	-2.11712	-1.00296
1	1.20928	2.08217	-2.1004
1	2.21578	0.830518	-2.83966
1	0.468702	0.817113	-3.09364
1	3.88331	0.43718	-1.42596
1	4.32521	0.064384	0.237344
1	3.54409	-1.16699	-0.76808
1	1.50378	-2.24981	0.692174
1	2.99535	-1.96695	1.58571
1	1.445	-1.96199	2.45735
1	-0.08705	-2.30626	-1.43325
1	-1.23146	-2.82028	-0.1793
1	-1.82771	-2.36362	-1.75332
1	-3.81787	0.849199	1.20009
1	-3.43648	1.4813	-0.39833
1	-1.42991	1.22324	1.91348
1	-2.06711	2.73144	1.26243
1	0.211838	2.1636	0.378982
1	-0.97588	2.31791	-0.90327
1	1.18501	-0.90826	-1.43548
1	2.24951	1.48143	0.120368

Conformer 8: E = -657.903576

B3LYP/6-31G**

6	2.63743	0.240043	-0.51442
6	3.17154	-0.811	0.443896
6	2.07119	-1.77497	0.879927

6	0.886729	-1.00179	1.45874
6	0.423658	0.164956	0.603498
6	1.23057	0.716218	-0.34103
8	3.35531	0.724505	-1.3809
6	-0.98579	0.655035	0.964685
6	-2.02732	0.408199	-0.16437
6	-0.98413	2.10285	1.49704
6	-3.46398	0.779542	0.264481
6	-2.048	-1.07055	-0.57796
8	-2.00122	-1.96119	0.25262
6	-2.16884	-1.36911	-2.05853
6	0.890655	1.85906	-1.26975
1	-0.79839	2.84263	0.714375
1	-1.93847	2.3469	1.97214
1	-0.20268	2.2229	2.25366
1	-3.55699	1.85094	0.457364
1	-4.18715	0.524081	-0.51738
1	-3.7448	0.233964	1.17076
1	-2.9674	-0.77601	-2.51764
1	-1.23505	-1.08321	-2.55815
1	-2.35239	-2.43288	-2.21496
1	1.10319	1.57284	-2.30423
1	-0.14511	2.18359	-1.20204
1	1.53563	2.72048	-1.06447
1	3.57282	-0.28067	1.32092
1	4.01524	-1.31109	-0.03938
1	2.44915	-2.49732	1.61144
1	1.72862	-2.35322	0.012281
1	1.14299	-0.61127	2.45803
1	0.035828	-1.67329	1.60953
1	-1.31779	0.018921	1.79287
1	-1.77215	1.0043	-1.04615

Conformer 9: E = -657.903575
B3LYP/6-31G**

6	2.63733	0.2402	-0.5149
6	3.17203	-0.8102	0.44379
6	2.0721	-1.77444	0.880316
6	0.887367	-1.00157	1.45898
6	0.423841	0.16487	0.603581
6	1.23039	0.716121	-0.34125
8	3.35476	0.724407	-1.38188
6	-0.98568	0.654639	0.964989
6	-2.02732	0.408128	-0.16401

6	-0.98405	2.10231	1.49782
6	-3.46392	0.779466	0.265107
6	-2.04813	-1.07048	-0.578
8	-2.00055	-1.96141	0.252218
6	-2.1701	-1.36863	-2.0586
6	0.889968	1.85844	-1.27042
1	-0.7984	2.84232	0.71535
1	-1.93834	2.34619	1.97308
1	-0.20253	2.22217	2.25439
1	-3.55686	1.85085	0.458098
1	-4.18724	0.524099	-0.51664
1	-3.74462	0.233837	1.1714
1	-2.96891	-0.77534	-2.51703
1	-1.23661	-1.08275	-2.55881
1	-2.35386	-2.43234	-2.21514
1	-0.14526	2.18427	-1.20095
1	1.53645	2.71922	-1.06729
1	1.10006	1.571	-2.30507
1	3.57321	-0.27936	1.32054
1	4.01585	-1.31018	-0.03941
1	2.45043	-2.4964	1.61203
1	1.72968	-2.35314	0.012898
1	1.14342	-0.61082	2.45824
1	0.036693	-1.67336	1.60981
1	-1.31753	0.018248	1.79303
1	-1.77223	1.00446	-1.04566

Product Purification/Characterization Data

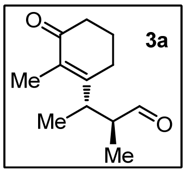

(*2S,3S*)-2-methyl-3-(2-methyl-3-oxocyclohex-1-en-1-yl)butanal (**3a**): The general procedure A was followed using 2-methyl-3-oxocyclohex-1-en-1-yl trifluoromethanesulfonate (**1a**, 65 mg, 0.25 mmol) and (*E*)-2-methylbut-2-en-1-ol (**2a**, 43 mg, 0.50 mmol). Purification of this material by chromatography on silica gel (1:5 EtOAc:hexanes) afforded product **3a** as a yellow liquid (36 mg, 74% yield): R_f = 0.20 (1:4 EtOAc:hexanes); **^{1}H NMR** ($CDCl_3$, 400 MHz) δ 9.47 (d, *J* = 2.0 Hz, 1H), 3.09-3.01 (m, 1H), 2.46-2.45 (m, 1H), 2.39 (t, *J* = 8.0 Hz, 2H), 2.28-2.24 (m, 2H), 1.94-1.89 (m, 2H), 1.81 (s, 3H), 1.16 (d, *J* = 12.0 Hz, 3H), 1.14 (d, *J* = 12.0 Hz, 3H); **^{13}C NMR** ($CDCl_3$, 100 MHz) δ 203.2, 199.1, 159.1, 131.2, 49.6, 38.4, 37.9, 25.9, 22.5, 15.3, 11.9, 10.6 ppm; **IR (neat)** 2938, 2877, 1724, 1663, 1559, 1457 cm^{-1}; **HRMS** (ESI-TOF) *m/z* calcd for $C_{12}H_{18}O_2Na$ $(M + Na)^+$: 217.1204, found 217.1206; 95:5 er; >20:1 dr; $[\alpha]_D^{20}$ = +26 (c = 0.1, EtOH).

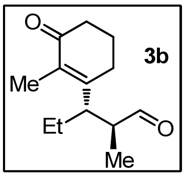

(*2S,3S*)-2-methyl-3-(2-methyl-3-oxocyclohex-1-en-1-yl)pentanal (**3b**): The general procedure A was followed using 2-methyl-3-oxocyclohex-1-en-1-yl trifluoromethanesulfonate (**1a**, 65 mg, 0.25 mmol) and (*E*)-2-methylpent-2-en-1-ol (**2b**, 50 mg, 0.50 mmol). Purification of this material by chromatography on silica gel (1:5 EtOAc:hexanes) afforded product **3b** as a yellow liquid (44 mg, 84% yield): R_f = 0.20 (1:4 EtOAc:hexanes); **^{1}H NMR** ($CDCl_3$, 400 MHz) δ 9.44 (d, *J* = 4.0 Hz, 1H), 2.91-2.86 (m, 1H), 2.42-2.39 (m, 3H), 2.23-2.20 (m, 2H), 1.93-1.90 (m, 2H), 1.82-1.77 (m, 4H), 1.44-1.39 (m, 1H), 1.18 (d, *J* = 4.0 Hz, 3H), 0.83 (t, *J* = 8.0 Hz, 3H); **^{13}C NMR** ($CDCl_3$, 100 MHz) δ 203.1, 198.9, 157.2, 133.5, 49.4, 45.9, 38.0, 25.6, 22.5, 22.4, 12.4, 11.6, 11.1 ppm; **IR** (neat) 2962, 2874, 1723, 1662, 1457, 1301 cm^{-1}; **HRMS** (ESI-TOF) *m/z* calcd for $C_{13}H_{20}O_2Na$ $(M + Na)^+$: 231.1361, found 231.1357; 94:6 er, >20:1 dr. $[\alpha]_D^{20}$ = –10 (c = 0.1, EtOH);

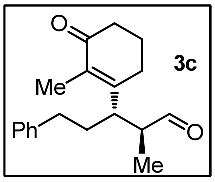

(*2S,3S*)-2-methyl-3-(2-methyl-3-oxocyclohex-1-en-1-yl)-5-phenylpentanal (**3c**): The general procedure A was followed using 2-methyl-3-oxocyclohex-1-en-1-yl trifluoromethanesulfonate (**1a**, 65 mg, 0.25 mmol) and (*E*)-2-methyl-5-phenylpent-2-en-1-ol (**2c**, 88 mg, 0.50 mmol). Purification of this material by chromatography on silica gel (1:5 EtOAc:hexanes) afforded product **3c** as a yellow liquid (29 mg, 41% yield): R_f = 0.30 (1:4 EtOAc:hexanes); **^{1}H NMR** ($CDCl_3$, 400 MHz) δ 9.43 (d, *J* = 4.0 Hz, 1H), 7.29 (t, *J* = 6.0 Hz, 2H), 7.20 (t, *J* = 8.0 Hz, 1H), 7.12 (d, *J* = 8.0 Hz, 2H), 3.04-2.98 (m, 1H), 2.56-2.40 (m, 5H), 2.27-2.24 (m, 2H), 2.1-1.90 (m, 3H), 1.82-1.72 (m, 4H), 1.17 (d, *J* = 8.0 Hz, 3H); **^{13}C NMR** ($CDCl_3$, 100 MHz) δ 202.8, 198.9, 156.8, 141.1, 133.7, 128.5, 128.2, 126.2, 49.4, 43.9, 38.0, 33.4, 31.2, 25.8, 22.4, 12.3, 11.2 ppm; **IR** (neat) 2939, 2867, 1721, 1662, 1455, 700 cm^{-1}; **HRMS** (ESI-TOF) *m/z* calcd for $C_{19}H_{24}O_2Na$ $(M + Na)^+$: 307.1674, found 307.1682; 95:5 er, >20:1 dr; $[\alpha]_D^{20}$ = –4 (c = 0.1, EtOH).

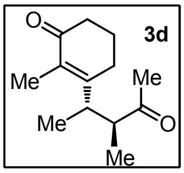

2-methyl-3-((*2S,3S*)-3-methyl-4-oxopentan-2-yl)cyclohex-2-enone (**3d**): The general procedure A was followed using 2-methyl-3-oxocyclohex-1-en-1-yl trifluoromethanesulfonate (**1a**, 65 mg, 0.25 mmol) and (*E*)-3-methylpent-3-en-2-ol (**2d**, 50 mg, 0.50 mmol). Purification of this material by chromatography on silica gel (1:5 EtOAc:hexanes) afforded product **3d** as a yellow liquid (30 mg, 60% yield): R_f = 0.40 (1:4 EtOAc:hexanes); **^{1}H NMR** ($CDCl_3$, 400 MHz) δ 3.08-3.00 (m, 1H), 2.66-2.61 (m, 1H), 2.37 (t, *J* = 6.0 Hz, 2H), 2.25-2.20 (m, 2H), 2.06 (s, 3H), 1.91-1.87 (m, 2H), 2.06 (s, 3H), 1.16 (d, *J* = 8.0 Hz, 3H), 1.08 (d, *J* = 8.0 Hz, 3H); **^{13}C NMR** ($CDCl_3$, 100 MHz) δ 211.1, 199.3, 160.2, 130.9, 50.5, 39.7, 37.9, 27.6, 25.7, 22.6, 15.3, 14.8, 10.5 ppm; **IR** (neat) 2937, 2876, 1709, 1661, 1456, 1353 cm^{-1}; **HRMS** (ESI-TOF) *m/z* calcd for $C_{13}H_{20}O_2Na$ $(M + Na)^+$: 231.1361, found 231.1367; 97:3 er, >20:1 dr; $[\alpha]_D^{20}$ = +20 (c = 0.1, EtOH).

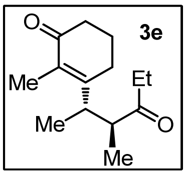

2-methyl-3-((*2S,3S*)-3-methyl-4-oxohexan-2-yl)cyclohex-2-enone (**3e**): The general procedure A was followed using 2-methyl-3-oxocyclohex-1-en-1-yl trifluoromethanesulfonate (**1a**, 65 mg, 0.25 mmol) and (*E*)-4-methylhex-4-en-3-ol (**2e**, 57 mg, 0.50 mmol). Purification of this material by chromatography on silica gel (1:5 EtOAc:hexanes) afforded product **3e** as a yellow liquid (30 mg, 54% yield): R_f = 0.40 (1:4 EtOAc:hexanes); **^{1}H NMR** ($CDCl_3$, 400 MHz) δ 3.10-3.02 (m, 1H), 2.68-2.65 (m, 1H), 2.36-2.18 (m, 6H), 1.91-1.84 (m, 2H), 1.80 (s, 3H), 1.15 (d, *J* = 4.0 Hz, 3H), 1.07 (d, *J* = 4.0 Hz, 3H), 0.98 (t, *J* = 6.0 Hz, 3H); **^{13}C NMR** ($CDCl_3$, 100 MHz) δ 213.6, 199.4, 160.3, 131.0, 49.6, 39.7, 37.9, 34.0, 25.8, 22.7, 15.4, 15.2, 10.6, 7.6 ppm; **IR** (neat) 2937, 2876, 1712, 1661, 1455, 1351 cm^{-1}; **HRMS** (ESI-TOF) *m/z* calcd for $C_{14}H_{22}O_2Na$ $(M + Na)^+$: 245.1517, found 245.1524; 98:2 er, >20:1 dr; $[\alpha]_D^{20}$ = +32 (c = 0.1, EtOH).

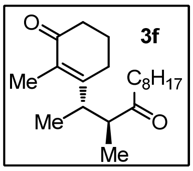

2-methyl-3-((*2S,3S*)-3-methyl-4-oxododecan-2-yl)cyclohex-2-enone (**3f**): The general procedure A was followed using 2-methyl-3-oxocyclohex-1-en-1-yl trifluoromethanesulfonate (**1a**, 65 mg, 0.25 mmol) and (*E*)-3-methyldodec-2-en-4-ol (**2f**, 99 mg, 0.50 mmol). Purification of this material by chromatography on silica gel (1:5 EtOAc:hexanes) afforded product **3f** as a yellow liquid (39 mg, 51% yield): R_f = 0.50 (1:4 EtOAc:hexanes); **^{1}H NMR** ($CDCl_3$, 400 MHz) δ 3.09-3.02 (m, 1H), 2.70-2.62 (m, 1H), 2.35-2.14 (m, 6H), 1.90-1.85 (m, 2H), 1.80 (s, 3H), 1.51-1.44 (m, 2H), 1.31-1.20 (m, 10H), 1.14 (d, *J* = 8.0 Hz, 3H), 1.06 (d, *J* = 8.0 Hz, 3H), 0.87 (t, *J* = 8.0 Hz, 3H); **^{13}C NMR** ($CDCl_3$, 100 MHz) δ 213.2, 199.3, 160.3, 131.0, 49.7, 40.9, 39.6, 37.9, 31.8, 29.4, 29.2, 29.1, 25.8, 23.4, 22.7, 22.6, 15.4 15.1, 14.1, 10.6 ppm; **IR** (neat) 2926, 2855, 1710, 1664, 1456, 1350 cm^{-1}; **HRMS** (ESI-TOF) *m/z* calcd for $C_{20}H_{34}O_2Na$ $(M + Na)^+$: 329.2456, found 329.2455; 98:2 er, >20:1 dr; $[\alpha]_D^{20}$ = +21 (c = 0.1, EtOH).

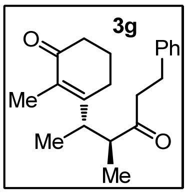

2-methyl-3-((*2S,3S*)-3-methyl-4-oxo-6-phenylhexan-2-yl)cyclohex-2-enone (**3g**): The general procedure A was followed using 2-methyl-3-oxocyclohex-1-en-1-yl trifluoromethanesulfonate (**1a**, 65 mg, 0.25 mmol) and (*E*)-4-methyl-1-phenylhex-4-en-3-ol (**2g**, 95 mg, 0.50 mmol). Purification of this material by chromatography on silica gel (1:10

EtOAc:hexanes) afforded product **3g** as a yellow liquid (37 mg, 49% yield): R$_f$ = 0.50 (1:4 EtOAc:hexanes); **^{1}H NMR** ($CDCl_3$, 400 MHz) δ 7.24 (t, *J* = 6.0 Hz, 2H), 7.18 (d, *J* = 4.0 Hz, 1H), 7.12 (d, *J* = 8.0 Hz, 2H), 3.07-2.99 (m, 1H), 2.88-2.59 (m, 5H), 2.37-2.02 (m, 4H), 1.84-1.72 (m, 5H), 1.09 (d, *J* = 8.0 Hz, 3H), 1.03 (d, *J* = 8.0 Hz, 3H); **^{13}C NMR** ($CDCl_3$, 100 MHz) δ 211.9, 199.3, 160.0, 141.1, 131.0, 128.5, 128.3, 126.1, 49.8, 42.6, 39.6, 37.9, 29.3, 25.7, 22.6, 15.4, 15.0, 10.6 ppm; **IR** (neat) 2934, 1711, 1622, 1454, 1351, 701 cm^{-1}; **HRMS** (ESI-TOF) *m/z* calcd for $C_{20}H_{26}O_2Na$ $(M + Na)^+$: 321.1830, found 321.1837; 98:2 er, >20:1 dr; $[\alpha]_D^{20}$ = -9 (c = 0.1, EtOH).

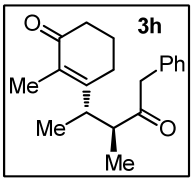

2-methyl-3-((*2S,3S*)-3-methyl-4-oxo-5-phenylpentan-2-yl)cyclohex-2-enone (**3h**): The general procedure A was followed using 2-methyl-3-oxocyclohex-1-en-1-yl trifluoromethanesulfonate (**1a**, 65 mg, 0.25 mmol) and (*E*)-3-methyl-1-phenylpent-3-en-2-ol (**2h**, 88 mg, 0.50 mmol). Purification of this material by chromatography on silica gel (1:10 EtOAc:hexanes) afforded product **3h** as a yellow liquid (32 mg, 46% yield): R$_f$ = 0.50 (1:4 EtOAc:hexanes); **^{1}H NMR** ($CDCl_3$, 400 MHz) δ 7.32-7.25 (m, 3H), 7.12 (d, *J* = 8.0 Hz, 2H), 3.65 (q, *J* = 16.0 Hz, 2H), 3.15-3.07 (m, 1H), 2.83-2.76 (m, 1H), 2.34-2.08 (m, 3H), 1.80-1.66 (m, 6H), 1.16 (d, *J* = 8.0 Hz, 3H), 1.04 (d, *J* = 8.0 Hz, 3H); **^{13}C NMR** ($CDCl_3$, 100 MHz) δ 209.8, 199.4, 160.1, 133.6, 131.1, 129.4, 128.7, 127.1, 48.6, 48.4, 39.2, 37.8, 25.5, 22.6, 15.37, 15.35, 10.6 ppm; **IR** (neat) 2937, 2875, 1715, 1662, 1456, 1327 cm^{-1}; **HRMS** (ESI-TOF) *m/z* calcd for $C_{19}H_{24}O_2Na$ $(M + Na)^+$ 307.1674, found 307.1678; 97:3 er, >20:1 dr. $[\alpha]_D^{20}$ = -90 (c = 0.1, EtOH).

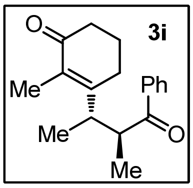

2-methyl-3-((*2S,3S*)-3-methyl-4-oxo-4-phenylbutan-2-yl)cyclohex-2-enone (**3i**): The general procedure A was followed using 2-methyl-3-oxocyclohex-1-en-1-yl trifluoromethanesulfonate (**1a**, 65 mg, 0.25 mmol) and (*E*)-2-methyl-1-phenylbut-2-en-1-ol (**2i**, 81 mg, 0.50 mmol). Purification of this material by chromatography on silica gel (1:5 EtOAc:hexanes) afforded product **3i** as a yellow liquid (27 mg, 40% yield): R$_f$ = 0.30 (1:4 EtOAc:hexanes); **^{1}H NMR** ($CDCl_3$, 400 MHz) δ 7.87 (d, *J* = 4.0 Hz, 2H), 7.56 (t, *J* = 8.0 Hz, 1H), 7.46 (d, *J* = 8.0 Hz, 2H), 3.65-3.57 (m, 1H), 3.28-3.20 (m, 1H), 2.30-2.18 (m, 2H), 2.17-1.93 (m, 2H), 1.84 (s, 3H), 1.75-1.55 (m, 1H), 1.50-1.42 (m, 1H), 1.28 (d, *J* = 8.0 Hz, 3H), 1.15 (d, *J* = 4.0 Hz, 3H); **^{13}C NMR** ($CDCl_3$, 100 MHz) δ 203.2, 199.3, 160.0, 136.7, 133.1, 131.2, 128.8, 127.9, 43.5, 40.2, 37.8, 25.9, 22.4, 16.3, 15.6, 10.6 ppm; **IR** (neat) 2934, 2873, 1679, 1661, 1215, 710 cm^{-1}; **HRMS** (ESI-TOF) *m/z* calcd for $C_{18}H_{22}O_2Na$ $(M + Na)^+$: 293.1517, found 293.1521; 96:4 er, >20:1 dr; $[\alpha]_D^{20}$ = –43 (c = 0.1, EtOH).

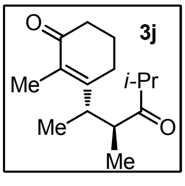

3-((*2S,3S*)-3,5-dimethyl-4-oxohexan-2-yl)-2-methylcyclohex-2-enone (**3j**): The general procedure A was followed using 2-methyl-3-oxocyclohex-1-en-1-yl trifluoromethanesulfonate (**1a**, 65 mg, 0.25 mmol) and (*E*)-2,4-dimethylhex-4-en-3-ol (**2j**, 64 mg, 0.50 mmol). Purification of this material by chromatography on silica gel (1:5 EtOAc:hexanes) afforded product **3j** as a yellow liquid (27 mg, 45% yield): R$_f$ = 0.30 (1:4 EtOAc:hexanes); **^{1}H NMR** ($CDCl_3$, 400 MHz): δ = 3.12-3.06 (m, 1H), 2.88 (m, 1H), 2.66-2.57 (m, 1H), 2.38-2.17 (m, 4H), 1.96-1.80 (m, 5H), 1.13 (d, *J* = 8.0 Hz, 3H), 1.06 (d, *J* = 8.0 Hz, 6H), 0.98 (d, *J* = 6.0 Hz, 3H); **^{13}C NMR** ($CDCl_3$, 100 MHz) δ 216.7, 199.3, 160.2, 131.2, 47.9, 40.0, 39.5, 38.0, 25.9, 22.7, 18.5,

18.1, 15.5, 15.4, 10.6 ppm; **IR** (neat) 2969, 2935, 1701, 1663, 1507, 1457 cm^{-1}; **HRMS** (ESI-TOF) *m/z* calcd for $C_{15}H_{24}O_2Na$ $(M + Na)^+$: 259.1674, found 259.1680; 98:2 er, >20:1 dr; $[\alpha]_D^{20}$ = +42 (c = 0.1, EtOH).

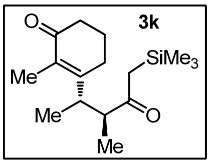

2-methyl-3-((*2S,3S*)-3-methyl-4-oxo-5-(trimethylsilyl)pentan-2-yl)cyclohex-2-enone (**3k**): The general procedure A was followed using 2-methyl-3-oxocyclohex-1-en-1-yl trifluoromethanesulfonate (**1a**, 65 mg, 0.25 mmol) and (*E*)-3-methyl-1-(trimethylsilyl)pent-3-en-2-ol (**2k**, 86 mg, 0.50 mmol). Purification of this material by chromatography on silica gel (1:5 EtOAc:hexanes) afforded product **3k** as a yellow liquid (13 mg, 19% yield): R_f = 0.40 (1:4 EtOAc:hexanes); **^{1}H NMR** ($CDCl_3$, 400 MHz) δ 3.08-2.99 (m, 1H), 2.64-2.56 (m, 1H), 2.41-2.25 (m, 4H), 2.11 (s, 2H), 1.96-1.86 (m, 2H), 1.79 (s, 3H), 1.09 (d, *J* = 8.0 Hz, 3H), 1.05 (d, *J* = 8.0 Hz, 3H), 0.10 (s, 9H); **^{13}C NMR** ($CDCl_3$, 100 MHz) δ 211.8, 199.4, 160.4, 131.2, 50.5, 40.0, 38.0, 36.8, 26.2, 22.7, 15.2, 14.1, 10.8, 0.8 ppm; **IR** (neat) 2926, 2855, 1686, 1664, 1251, 853 cm^{-1}; **HRMS** (ESI-TOF) *m/z* calcd for $C_{16}H_{28}O_2NaSi$ $(M + Na)^+$: 303.1756, found 303.1758; 97:3 er, >20:1 dr; $[\alpha]_D^{20}$ = +55 (c = 0.1, EtOH).

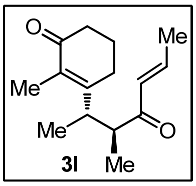

2-methyl-3-((*2S,3S,E*)-3-methyl-4-oxohept-5-en-2-yl)cyclohex-2-enone (**3l**): The general procedure A was followed using 2-methyl-3-oxocyclohex-1-en-1-yl trifluoromethanesulfonate (**1a**, 65 mg, 0.25 mmol) and (*E*)-1-cyclopropyl-2-methylbut-2-en-1-ol (**2l**, 63 mg, 0.50 mmol). Purification of this material by chromatography on silica gel (1:5 EtOAc:hexanes) afforded product **3l** as a yellow liquid (23 mg, 40% yield): R_f = 0.30 (1:4 EtOAc:hexanes); **^{1}H NMR** ($CDCl_3$, 500 MHz) δ 6.89-6.81 (m, 1H), 6.13 (dd, J_1 = 15.0 Hz, J_2 = 2.5 Hz, 1H), 3.11-3.05 (m, 1H), 2.86-2.80 (m, 1H), 2.35-2.32 (m, 2H), 2.31-2.09 (m, 2H), 1.89-1.83 (m, 5H), 1.79 (s, 3H), 1.16 (d, *J* = 8.0 Hz, 3H), 1.08 (d, *J* = 8.0 Hz, 3H); **^{13}C NMR** ($CDCl_3$, 100 MHz) δ 202.0, 199.5, 160.3, 143.1, 131.0, 129.8, 47.2, 39.8, 37.9, 25.8, 22.6, 18.3, 15.5, 15.4, 10.5 ppm; **IR** (neat) 2936, 2875, 1662, 1627, 1456, 1350 cm^{-1}; **HRMS** (ESI-TOF) *m/z* calcd for $C_{15}H_{22}O_2Na$ $(M + Na)^+$: 257.1517, found 257.1523; 97:3 er, >20:1 dr; $[\alpha]_D^{20}$ = -23 (c = 0.1, EtOH).

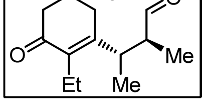

(*2S,3S*)-3-(2-ethyl-3-oxocyclohex-1-en-1-yl)-2-methylbutanal (**3m**): The general procedure A was followed using 2-ethyl-3-oxocyclohex-1-en-1-yl trifluoromethanesulfonate (**1m**, 68 mg, 0.25 mmol) and (*E*)-2-methylbut-2-en-1-ol (**2a**, 43 mg, 0.5 mmol). Purification of this material by chromatography on silica gel (1:5 EtOAc:hexanes) afforded product **3m** as a yellow liquid (44 mg, 85% yield): R_f = 0.20 (1:4 EtOAc:hexanes); **^{1}H NMR** ($CDCl_3$, 400 MHz) δ 9.84 (d, *J* = 4.0 Hz, 1H), 3.05-2.96 (m, 1H), 2.49-2.18 (m, 7H), 1.95-1.84 (m, 2H), 1.17 (t, *J* = 8.0 Hz, 6H), 0.93 (t, *J* = 6.0 Hz, 3H); **^{13}C NMR** ($CDCl_3$, 100 MHz) δ 203.3, 198.7, 158.7, 137.3, 49.4, 38.17, 38.15, 25.6, 22.6, 18.2, 16.2, 14.0, 12.1 ppm; **IR** (neat) 2967, 2874, 1723, 1661, 1455, 1184 cm^{-1}; **HRMS** (ESI-TOF) *m/z* calcd for $C_{13}H_{20}O_2Na$ $(M + Na)^+$: 231.1361, found 231.1360; 96:4 er, >20:1 dr; $[\alpha]_D^{20}$ = +29 (c = 0.1, EtOH).

(*2S,3S*)-3-(2-benzyl-3-oxocyclohex-1-en-1-yl)-2-methylbutanal (**3n**): The general procedure A was followed using 2-benzyl-3-oxocyclohex-1-en-1-yl trifluoromethanesulfonate (**1n**, 84 mg, 0.25 mmol) and (*E*)-2-methylbut-2-en-1-ol (**2a**, 43 mg, 0.5 mmol). Purification of this material by chromatography on silica gel (1:5 EtOAc:hexanes) afforded product **3n** as a yellow liquid (46 mg, 68% yield): R_f = 0.40 (1:4 EtOAc:hexanes); **^{1}H NMR** ($CDCl_3$, 400 MHz) δ 8.92 (d, J = 4.0 Hz, 1H), 7.26-7.21 (m, 2H), 7.15-7.07 (m, 3H), 3.74 (q, J = 16.0 Hz, 2H), 3.09-3.03 (m, 1H), 2.47 (t, J = 6.0 Hz, 2H), 2.37-2.24 (m, 3H), 2.01-1.89 (m, 2H), 1.06 (d, J = 8.0 Hz, 3H), 1.01 (d, J = 8.0 Hz, 3H); **^{13}C NMR** ($CDCl_3$, 100 MHz) δ 203.4, 198.8, 161.4, 140.7, 134.8, 128.3, 129.1, 125.8, 49.6, 38.6, 38.1, 30.0, 25.7, 22.5, 15.5, 12.0 ppm; **IR** (neat) 2937, 2875, 1720, 1662, 1453, 726 cm^{-1}; **HRMS** (ESI-TOF) *m/z* calcd for $C_{18}H_{22}O_2Na$ (M + Na)$^+$: 293.1517, found 293.1521; 93:7 er, 20:1 dr; $[\alpha]_D^{20}$ = -14 (c = 0.1, EtOH).

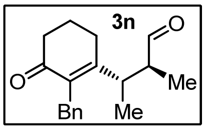

(*2S,3S*)-3-(2-isopropyl-3-oxocyclohex-1-en-1-yl)-2-methylbutanal (**3o**): The general procedure A was followed using 2-isopropyl-3-oxocyclohex-1-en-1-yl trifluoromethanesulfonate (**1o**, 72 mg, 0.25 mmol) and (*E*)-2-methylbut-2-en-1-ol (**2a**, 43 mg, 0.5 mmol). Purification of this material by chromatography on silica gel (1:5 EtOAc:hexanes) afforded product **3o** as a yellow liquid (30 mg, 54% yield): R_f = 0.30 (1:4 EtOAc:hexanes); **^{1}H NMR** ($CDCl_3$, 400 MHz) δ 9.49 (d, J = 4.0 Hz, 1H), 3.16-3.09 (m, 1H), 3.01-2.94 (m, 1H), 2.50-2.40 (m, 1H), 2.34-2.16 (m, 4H), 1.90-1.81 (m, 2H), 1.19-1.13 (m, 12H); **^{13}C NMR** ($CDCl_3$, 100 MHz) δ 203.4, 199.0, 158.2, 140.4, 49.5, 39.3, 37.7, 27.3, 26.3, 22.4, 21.2, 20.7, 16.2, 12.1 ppm; **IR** (neat) 2937, 1724, 1663, 1457, 1298, 668 cm^{-1}; **HRMS** (ESI-TOF) *m/z* calcd for $C_{14}H_{22}O_2Na$ (M + Na)$^+$: 245.1517, found 245.1509; 96:4 er, >20:1 dr; $[\alpha]_D^{20}$ = +28 (c = 0.1, EtOH).

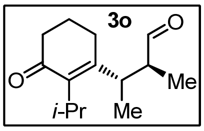

(*2S,3S*)-3-(2-bromo-3-oxocyclohex-1-en-1-yl)-2-methylbutanal (**3p**): The general procedure A was followed using 2-bromo-3-oxocyclohex-1-en-1-yl trifluoromethanesulfonate (**1p**, 81 mg, 0.25 mmol) and (*E*)-2-methylbut-2-en-1-ol (**2a**, 43 mg, 0.5 mmol). Purification of this material by chromatography on silica gel (1:5 EtOAc:hexanes) afforded product **3p** as a yellow liquid (14 mg, 22% yield): R_f = 0.20 (1:4 EtOAc:hexanes) **^{1}H NMR** ($CDCl_3$, 500 MHz) δ 9.53 (d, J = 4.0 Hz, 1H), 3.53-3.47 (m, 1H), 2.61-2.51 (m, 3H), 2.46-2.35 (m, 2H), 2.0-1.95 (m, 2H), 1.21 (d, J = 4.0 Hz, 3H), 1.16 (d, J = 4.0 Hz, 3H); **^{13}C NMR** ($CDCl_3$, 125 MHz) δ 202.8, 190.9, 164.1, 123.6, 49.7, 42.6, 38.1, 28.5, 22.0, 14.5, 11.6 ppm; **IR (neat)**: ν = 2936, 2876, 1723, 1685, 1585, 1268 cm^{-1}; **HRMS** (ESI-TOF) *m/z* calcd for $C_{11}H_{16}O_2Br$ (M + H)$^+$: 259.0334, found 259.0338; 95:5 er, >20:1 dr; $[\alpha]_D^{20}$ = –3 (c = 0.1, EtOH).

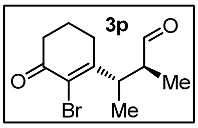

(*2S,3S*)-2-methyl-3-(2-methyl-3-oxocyclopent-1-en-1-yl)butanal (**3q**): The general procedure A was followed using 2-methyl-3-oxocyclopent-1-en-1-yl trifluoromethanesulfonate (**1q**, 61 mg, 0.25 mmol) and (*E*)-2-methylbut-2-en-1-ol (**2a**, 43 mg, 0.5 mmol). Purification of this material by chromatography on silica gel (1:5 EtOAc:hexanes) afforded product **3q** as a yellow liquid (39 mg, 87% yield): R_f = 0.20 (1:4 EtOAc:hexanes); **^{1}H NMR** ($CDCl_3$, 400 MHz) δ 9.54 (d, J = 4.0 Hz, 1H), 3.18-3.10 (m, 1H), 2.59-2.52 (m, 2H), 2.49-2.36 (m, 3H), 1.74-1.72 (m, 3H), 1.19 (d, J =

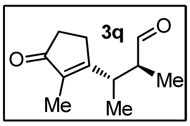

8.0 Hz, 3H), 1.18 (d, J = 4.0 Hz, 3H); **^{13}C NMR** ($CDCl_3$, 100 MHz) δ 209.7, 202.9, 173.8, 136.5, 49.8, 35.6, 33.8, 26.0, 15.4, 11.6, 8.3 ppm; **IR** (neat) 2971.2, 2924.0, 1722.9, 1696.1, 1640.5, 1328.4 cm^{-1}; **HRMS** (ESI-TOF) *m/z* calcd for $C_{11}H_{16}O_2Na$ $(M + Na)^+$: 203.1048, found 203.1045; 94:6 er, >20:1 dr; $[\alpha]_D^{20}$ = +35 (c = 0.1, EtOH).

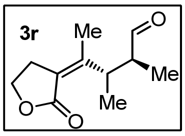

(*2S,3S,Z*)-2,3-dimethyl-4-(2-oxodihydrofuran-3(*2H*)-ylidene)pentanal (**3r**): The general procedure A was followed using (*Z*)-1-(2-oxodihydrofuran-3(*2H*)-ylidene)ethyl trifluoromethanesulfonate (**1r**, 65 mg, 0.25 mmol) and (*E*)-2-methylbut-2-en-1-ol (**2a**, 43 mg, 0.5 mmol). Purification of this material by chromatography on silica gel (1:4 EtOAc:hexanes) afforded product **3r** as a yellow liquid (42 mg, 86% yield): R_f = 0.10 (1:4 EtOAc:hexanes); **^{1}H NMR** ($CDCl_3$, 400 MHz) δ 9.43 (d, J = 4.0 Hz, 1H), 4.59-4.51 (m, 1H), 4.31 (t, J = 6.0 Hz, 2H), 2.85 (t, J = 6.0 Hz, 2H), 2.35-2.28 (m, 1H), 1.78 (s, 3H), 1.16 (d, J = 4.0 Hz, 3H), 1.08 (d, J = 4.0 Hz, 3H); **^{13}C NMR** ($CDCl_3$, 100 MHz) δ 204.5, 169.8, 155.4, 120.0, 64.3, 50.4, 33.7, 27.9, 17.1, 15.3, 11.7 ppm; **IR** (neat) 2974, 2917, 1735, 1653, 1194, 1066 cm^{-1}; **HRMS** (ESI-TOF) *m/z* calcd for $C_{11}H_{16}O_3Na$ $(M + Na)^+$: 219.0997, found 219.0998; 92:8 er, 20:1 dr; $[\alpha]_D^{20}$ = -12 (c = 0.1, EtOH).

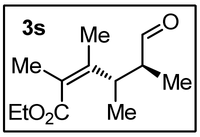

(*4S,5S,Z*)-ethyl 2,3,4,5-tetramethyl-6-oxohex-2-enoate (**3s**): The general procedure A was followed using (*Z*)-ethyl 2-methyl-3-(((trifluoromethyl)sulfonyl)oxy)but-2-enoate (**1s**, 69 mg, 0.25 mmol) and (*E*)-2-methylbut-2-en-1-ol (**2a**, 43 mg, 0.5 mmol). Purification of this material by chromatography on silica gel (1:100 EtOAc:toluene) afforded product **3s** as a yellow liquid (38 mg, 72% yield): R_f = 0.60 (1:10 toluene:hexanes); **^{1}H NMR** ($CDCl_3$, 400 MHz) δ 9.84 (d, J = 4.0Hz, 1H), 4.22-4.14 (m, 2H), 3.38-3.31 (m, 1H), 2.32-2.26 (m, 1H), 1.82 (s, 3H), 1.67 (s, 3H), 1.29 (t, J = 8.0 Hz, 3H), 1.11-1.06 (m, 6H); **^{13}C NMR** ($CDCl_3$, 100 MHz) δ 205.1, 169.8, 145.0, 124.9, 60.3, 49.8, 38.8, 16.2, 15.9, 14.23, 14.19, 12.0 ppm; **IR** (neat) 2975, 2932, 1718, 1701, 1218, 1106 cm^{-1}; **HRMS** (ESI-TOF) *m/z* calcd for $C_{12}H_{20}O_3Na$ $(M + Na)^+$: 235.1310, found 235.1306; 91:9 er, >20:1 dr; $[\alpha]_D^{20}$ = +2 (c = 0.1, EtOH).

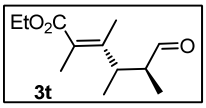

(*4S,5S,E*)-ethyl 2,3,4,5-tetramethyl-6-oxohex-2-enoate (**3t**): The general procedure A was followed using (*E*)-ethyl 2-methyl-3-(((trifluoromethyl)sulfonyl)oxy)but-2-enoate (**1t**, 69 mg, 0.25 mmol) and (*E*)-2-methylbut-2-en-1-ol (**2a**, 43 mg, 0.5 mmol). Purification of this material by chromatography on silica gel (1:100 EtOAc:toluene) afforded product **3t** as a yellow liquid (14 mg, 27% yield): R_f = 0.60 (1:10 toluene:hexanes); **^{1}H NMR** ($CDCl_3$, 500 MHz) δ 9.52 (d, J = 2.5 Hz, 1H), 4.20-4.16 (m, 2H), 2.94-2.88 (m, 1H), 2.48-2.42 (m, 1H), 1.89 (s, 3H), 1.85 (s, 3H), 1.29 (t, J = 5.0 Hz, 3H), 1.12 (d, J = 5.0 Hz, 3H), 1.08 (d, J = 10.0 Hz, 3H); **^{13}C NMR** ($CDCl_3$, 125 MHz) δ 203.9, 170.0, 144.8, 124.3, 60.2, 49.2, 38.0, 15.53, 15.48, 15.4, 14.2, 11.9 ppm; **IR** (neat) 2981, 2931, 1720, 1777, 1218, 1101 cm^{-1}; **HRMS** (ESI-TOF) *m/z* calcd for $C_{12}H_{20}O_3Na$ $(M + Na)^+$: 235.1310, found 235.1312; 97:3 er, 20:1 dr; $[\alpha]_D^{20}$ = +23 (c = 0.1, EtOH).

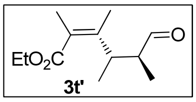

(*4S,5S,Z*)-ethyl 2,3,4,5-tetramethyl-6-oxohex-2-enoate (**3t'**): The general procedure A was followed using (*E*)-ethyl 2-methyl-3-(((trifluoromethyl)sulfonyl)oxy)but-2-enoate (**1t**, 69 mg, 0.25

mmol) and (*E*)-2-methylbut-2-en-1-ol (**2a**, 43 mg, 0.5 mmol). Purification of this material by chromatography on silica gel (1:100 EtOAc:toluene) afforded product **3t'** as a yellow liquid (16 mg, 29% yield): R_f = 0.60 (1:10 toluene:hexanes); **^{1}H NMR** ($CDCl_3$, 500 MHz) δ 9.48 (d, *J* = 5.0 Hz, 1H), 4.22-4.13 (m, 2H), 3.38-3.32 (m, 1H), 2.33-2.25 (m, 1H), 1.83 (s, 3H), 1.66 (s, 3H), 1.29 (t, *J* = 7.5 Hz, 3H), 1.10-1.06 (m, 6H); **^{13}C NMR** ($CDCl_3$, 125 MHz) δ 205.0, 169.7, 144.9, 124.9, 60.3, 49.7, 38.8, 16.2, 15.9, 14.21, 14.16, 12.0 ppm; **IR** (neat) 2976, 2935, 1718, 1276, 1219, 1106 cm^{-1}; **HRMS** (ESI-TOF) *m/z* calcd for $C_{12}H_{20}O_3Na$ $(M + Na)^+$ 235.1310, found 235.1312; 91:9 er, >20:1 dr; $[\alpha]_D^{20}$ = +2 (c = 0.1, EtOH).

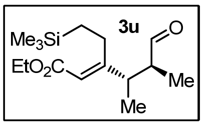

(*4S,5S,E*)-ethyl 4,5-dimethyl-6-oxo-3-(2-(trimethylsilyl)ethyl)hex-2-enoate (**3u**): The general procedure A was followed using (*E*)-ethyl 3-(((trifluoromethyl)sulfonyl)oxy)-5-(trimethylsilyl)pent-2-enoate (**1u**, 87 mg, 0.25 mmol) and (*E*)-2-methylbut-2-en-1-ol (**2a**, 43 mg, 0.5 mmol). Purification of this material by chromatography on silica gel (1:50 EtOAc:hexanes) afforded product **3u** as a yellow liquid (61 mg, 86% yield): R_f = 0.60 (1:20 toluene:hexanes); **^{1}H NMR** ($CDCl_3$, 400 MHz) δ 9.66 (d, *J* = 4.0 Hz, 1H), 5.58 (s, 1H), 4.18-4.12 (m, 2H), 2.81-2.70 (m, 2H), 2.57-2.50 (m, 1H), 2.37-2.30 (m, 1H), 1.28 (t, *J* = 8.0 Hz, 3H), 1.06-1.04 (m, 6H), 0.78-0.62 (m, 2H), 0.05 (s, 9H); **^{13}C NMR** ($CDCl_3$, 100 MHz) δ 203.9, 168.2, 166.2, 144.9, 59.6, 49.1, 40.3, 26.4, 16.3, 15.5, 14.3, 9.5, 1.9 ppm; **IR** (neat) 2954, 1715, 1636.7, 1154, 861, 835 cm^{-1}; **HRMS** (ESI-TOF) *m/z* calcd for $C_{15}H_{28}O_3NaSi$ $(M + Na)^+$: 307.1705, found 307.1708; 90:10 er, >20:1 dr; $[\alpha]_D^{20}$ = +32 (c = 0.1, EtOH).

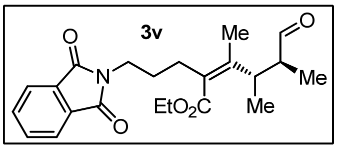

(*4S,5S,Z*)-ethyl 2-(3-(1,3-dioxoisoindolin-2-yl)propyl)-3,4,5-trimethyl-6-oxohex-2-enoate (**3v**): The general procedure A was followed using (*Z*)-ethyl 5-(1,3-dioxoisoindolin-2-yl) -2-(1-(((trifluoromethyl)sulfonyl)oxy)ethylidene) pentanoate (**1v**, 112 mg, 0.25 mmol) and (*E*)-2-methylbut-2-en-1-ol (**2a**, 43 mg, 0.5 mmol). Purification of this material by chromatography on silica gel (1:20 EtOAc:toluene) afforded product **3v** as a yellow liquid (56 mg, 58% yield): R_f = 0.30 (1:5 toluene:hexanes); **^{1}H NMR** ($CDCl_3$, 500 MHz) δ 9.47 (d, *J* = 5.0 Hz, 1H), 7.85-7.82 (m, 2H), 7.72-7.69 (m, 2H), 4.19-4.12 (m, 2H), 3.70-3.67 (m, 2H), 3.25-3.16 (m, 1H), 2.32-2.25 (m, 3H), 1.83-1.64 (m, 5H), 1.25 (t, *J* = 7.5 Hz, 3H), 1.09 (d, *J* = 5.0 Hz, 3H), 1.05 (d, *J* = 10.0 Hz, 3H); **^{13}C NMR** ($CDCl_3$, 125 MHz) δ 204.7, 169.3, 168.3, 144.8, 133.9, 132.1, 129.0, 123.2, 60.4, 49.6, 39.2, 37.7, 27.5, 27.2, 16.2, 14.2, 13.6, 12.0 ppm; **IR** (neat) 2973, 1712, 1396, 1369, 1215, 721 cm^{-1}; **HRMS** (ESI-TOF) *m/z* calcd for $C_{22}H_{27}NO_5Na$ $(M + Na)^+$: 408.1787, found 408.1778; 91:9 er, 16:1 dr; $[\alpha]_D^{20}$ = +5 (c = 0.1, EtOH).

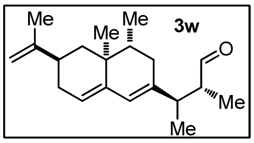

(*2R,3R*)-3-((*4R,4aS,6R*)-4,4a-dimethyl-6-(prop-1-en-2-yl)-3,4,4a,5,6,7-hexahydronaphthalen-2-yl)-2-methylbutanal (**3w**): The general procedure A was followed using (*R*)-4-(tert-butyl)-2-(5-(trifluoromethyl)pyridin-2-yl)-4,5-dihydrooxazole (10.2 mg, 0.0375 mmol), (*4R,4aS,6R*)-4,4a-dimethyl-6-(prop-1-en-2-yl)-3,4,4a,5,6,7-hexahydronaphthalen-2-yl trifluoromethanesulfonate (**1w**, 88 mg, 0.25 mmol) and (*E*)-2-methylbut-2-en-1-ol (**2a**, 86 mg, 1.0 mmol). Purification of this material by chromatography on silica gel (1:20 EtOAc:hexanes) afforded product **3w** as a white waxy solid (22 mg, 31% yield): R_f = 0.60 (1:4 EtOAc:hexanes);

^{1}**H NMR** ($CDCl_3$, 500 MHz) δ 9.55 (d, *J* = 2.5 Hz, 1H), 5.80 (m, 1H), 5.40 (d, *J* = 5.0 Hz, 1H), 4.74 (t, *J* = 2.5 Hz, 2H), 2.51-2.39 (m, 3H), 2.26-2.20 (m, 1H), 2.00-1.90 (m, 2H), 1.83-1.70 (m, 5H), 1.53-1.44 (m, 1H), 1.18-1.13 (m, 1H), 1.07-1.03 (m, 6H), 0.90 (d, *J* = 10.0 Hz, 3H), 0.83 (s, 3H); ^{13}**C NMR** ($CDCl_3$, 125 MHz) δ 205.1, 150.3, 142.0, 138.7, 125.1, 122.4, 108.6, 49.5, 41.5, 40.1, 39.1, 37.3, 36.0, 32.5, 31.1, 20.7, 17.3, 16.1, 14.8, 11.1 ppm; **IR (neat)** 2960, 2872, 1730, 1721, 1562, 1453 cm^{-1}; **HRMS** (ESI-TOF) *m/z* calcd for $C_{20}H_{31}O$ $(M + H)^+$: 287.2375, found 287.2373; 1:7.9 dr; $[\alpha]_D^{20}$ = –197 (c = 0.1, EtOH).

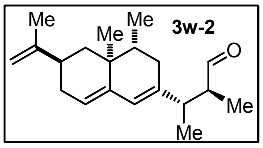

(*2S,3S*)-3-((*4R,4aS,6R*)-4,4a-dimethyl-6-(prop-1-en-2-yl)-3,4,4a,5,6,7-hexahydronaphthalen-2-yl)-2-methylbutanal (**3w-2**): The general procedure A was followed using (*S*)-4-(tert-butyl)-2-(5-(trifluoromethyl)pyridin-2-yl)-4,5-dihydrooxazole (10.2 mg, 0.0375 mmol), (*4R,4aS,6R*)-4,4a-dimethyl-6-(prop-1-en-2-yl)-3,4,4a,5,6,7-hexahydronaphthalen-2-yl trifluoromethanesulfonate (**1w**, 88 mg, 0.25 mmol) and (*E*)-2-methylbut-2-en-1-ol (**2a**, 86 mg, 1.0 mmol). Purification of this material by chromatography on silica gel (1:20 EtOAc:hexanes) afforded product **3w'** as a white waxy solid (19 mg, 27% yield): R_f = 0.60 (1:4 EtOAc:hexanes); ^{1}**H NMR** ($CDCl_3$, 400 MHz) δ 9.55 (d, *J* = 2.0 Hz, 1H), 5.80 (t, *J* = 6.0 Hz, 1H), 5.40 (d, *J* = 8.0 Hz, 1H), 4.7 (s, 2H), 2.54-2.37 (m, 3H), 2.27-2.19 (m, 1H), 2.01-1.89 (m, 2H), 1.84-1.70 (m, 5H), 1.56-1.43 (m, 1H), 1.28-0.98 (m, 7H), 0.91-0.83 (m, 6H); ^{13}**C NMR** ($CDCl_3$, 100 MHz) δ 205.1, 150.3, 142.0, 138.7, 125.1, 122.4, 108.6, 49.5, 41.5, 40.1, 39.1, 37.3, 36.0, 32.5, 31.1 ppm; **IR** (neat) 2967, 2877, 1733, 1725, 1559, 1457 cm^{-1}; **HRMS** (ESI-TOF) *m/z* calcd for $C_{20}H_{31}O$ $(M + H)^+$: 287.2375, found 287.2373; dr = 1:2.3; $[\alpha]_D^{20}$ = -151 (c = 0.1, EtOH).

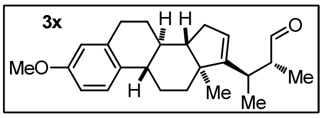

(*2R,3R*)-3-((*8S,9S,13S,14S*)-3-methoxy-13-methyl-7,8,9,11,12,13,14,15-octahydro-6H-cyclopenta[*a*]phenanthren-17-yl)-2-methylbutanal (**3x**): The general procedure A was followed using (*R*)-4-(tert-butyl)-2-(5-(trifluoromethyl)pyridin-2-yl)-4,5-dihydrooxazole (10.2 mg, 0.0375 mmol), (*8R,9S,13S,14S*)-3-methoxy-13-methyl-7,8,9,11,12,13,14,15-octahydro-6H-cyclopenta[*a*]phenanthren-17-yl trifluoromethanesulfonate (**1x**, 104 mg, 0.25 mmol) and (*E*)-2-methylbut-2-en-1-ol (**2a**, 86 mg, 1.0 mmol). Purification of this material by chromatography on silica gel (1:20 EtOAc:hexanes) afforded product **3x** as a white waxy solid (70 mg, 79% yield): R_f = 0.50 (1:4 EtOAc:hexanes); ^{1}**H NMR** ($CDCl_3$, 500 MHz) δ 9.69 (d, *J* = 2.5 Hz, 1H), 7.18 (d, *J* = 10.0 Hz, 1H), 6.70 (d, *J* = 10.0 Hz, 1H), 6.63 (s, 1H), 5.46 (s, 1H), 3.77 (s, 3H), 2.90-2.75 (m, 3H), 2.53-2.51 (m, 1H), 2.36-2.18 (m, 3H), 1.93 (t, *J* = 10.0 Hz, 3H), 1.62-1.42 (m, 5H), 1.04-1.02 (m, 6H), 0.84 (s, 3H); ^{13}**C NMR** ($CDCl_3$, 125 MHz) δ 205.2, 157.5, 157.4, 138.0, 132.8, 125.9, 124.5, 113.8, 111.4, 55.9, 55.2, 50.1, 47.8, 44.3, 37.4, 35.1, 31.4, 31.1, 29.7, 27.7, 26.5, 16.7, 16.5, 8.8 ppm; **IR** (neat) 2922, 2852, 1727, 1495, 1254, 1045 cm^{-1}; **HRMS** (ESI-TOF) *m/z* calcd for $C_{24}H_{33}O_2$ $(M + H)^+$: 353.2481, found 353.2483; 20:1 dr; $[\alpha]_D^{20}$ = +76 (c = 0.1, EtOH).

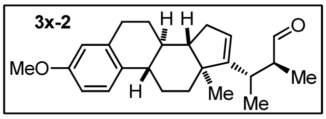

(*2S,3S*)-3-((*8S,9S,13S,14S*)-3-methoxy-13-methyl-7,8,9,11,12,13,14,15-octahydro-6H-cyclopenta[*a*]phenanthren-17-yl)-2-methylbutanal (**3x-2**): The general procedure A was followed using

(*S*)-4-(tert-butyl)-2-(5-(trifluoromethyl)pyridin-2-yl)-4,5-dihydrooxazole (10.2 mg, 0.0375 mmol), (*8R,9S,13S,14S*)-3-methoxy-13-methyl-7,8,9,11,12,13,14,15-octahydro-6H-cyclopenta[*a*]phenanthren-17-yl trifluoromethanesulfonate (**1x**, 104 mg, 0.25 mmol) and (*E*)-2-methylbut-2-en-1-ol (**2a**, 86 mg, 1.0 mmol). Purification of this material by chromatography on silica gel (1:20 EtOAc:hexanes) afforded product **3x-2** as a white waxy solid (70 mg, 79% yield): R_f = 0.50 (1:4 EtOAc:hexanes); **^{1}H NMR** ($CDCl_3$, 400 MHz) δ 9.58 (d, *J* = 2.0 Hz, 1H), 7.18 (d, *J* = 8.0 Hz, 1H), 6.70 (d, *J* = 8.0 Hz, 1H), 6.63 (s, 1H), 5.44 (d, *J* = 16.0 Hz, 1H), 3.77 (s, 3H), 2.93-2.53 (m, 4H), 2.36-2.14 (m, 3H), 1.93 (t, *J* = 8.0 Hz, 3H), 1.67-1.39 (m, 5H), 1.07-1.02 (m, 6H), 0.83 (d, *J* = 8.0 Hz, 3H); **^{13}C NMR** ($CDCl_3$, 100 MHz) δ 205.4, 205.2, 157.9, 157.5, 157.4, 138.0, 132.8, 125.93, 125.90, 124.5, 124.3, 113.8, 111.4, 56.7, 55.9, 55.2, 49.9, 47.3, 44.3, 44.2, 37.3, 34.9, 32.9, 30.9, 29.7, 27.7, 26.4, 17.6, 16.7, 16.5, 10.9, 8.8 ppm; **IR** (neat) 2927, 2848, 1722, 1498, 1254, 1049 cm^{-1}; **HRMS** (ESI-TOF) *m/z* calcd for $C_{24}H_{33}O_2$ $(M + H)^+$: 353.2481, found 353.2483; 1:1.8 dr; $[\alpha]_D^{20}$ = +56 (c = 0.1, EtOH).

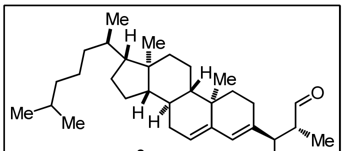

3y

(*2R,3R*)-3-((*8S,9S,10R,13R,14S,17R*)-10,13-dimethyl-17-((*R*)-6-methylheptan-2-yl)-2,7,8,9,10,11,12,13,14,15,16,17-dodecahydro-1H-cyclopenta[a]phenanthren-3-yl)-2-methylbutanal (**3y**): The general procedure A was followed using (*R*)-4-(tert-butyl)-2-(5-(trifluoromethyl)pyridin-2-yl)-4,5-dihydrooxazole (10.2 mg, 0.0375 mmol), (*8S,9S,10R,13R,14S,17R*)-10,13-dimethyl-17-((*R*)-6-methylheptan-2-yl)-2,7,8,9,10,11,12,13,14,15,16,17-dodecahydro-*1H*-cyclopenta[*a*]phenanthren-3-yl trifluoromethanesulfonate (**1y**, 129 mg, 0.25 mmol) and (*E*)-2-methylbut-2-en-1-ol (**2a**, 86 mg, 1.0 mmol). Purification of this material by chromatography on silica gel (1:30 EtOAc:hexanes) afforded product **3y** as a white waxy solid (29 mg, 26% yield): R_f = 0.70 (1:4 EtOAc:hexanes); **^{1}H NMR** ($CDCl_3$, 500 MHz) δ 9.56 (d, *J* = 2.5 Hz, 1H), 5.77 (s, 1H), 5.35 (s, 1H), 2.52-2.38 (m, 2H), 2.16 (d, *J* = 15.0 Hz, 1H), 2.05-2.00 (m, 3H), 1.84-1.79 (m, 2H), 1.68-1.42 (m, 5H), 1.35-1.23 (m, 7H), 1.14-1.03 (m, 14 H), 0.98-0.86 (m, 12 H), 0.7 (3H); **^{13}C NMR** ($CDCl_3$, 125 MHz) δ 205.1, 141.4, 137.9, 125.5, 122.8, 57.0, 56.2, 49.5, 48.3, 42.5, 41.6, 39.8, 39.5, 36.2, 35.8, 35.0, 34.2, 31.8, 28.2, 28.0, 24.2, 23.8, 23.4, 22.8, 22.6, 21.1, 18.8, 18.7, 16.1, 12.0, 11.0 ppm; **IR** (neat) 2936, 2864, 1725, 1461, 1459, 1382 cm^{-1}; **HRMS** (ESI-TOF) *m/z* calcd for $C_{32}H_{53}O$ $(M + H)^+$: 453.4096, found 453.4098; 1:7.9 dr; $[\alpha]_D^{20}$ = –147 (c = 0.1, EtOH).

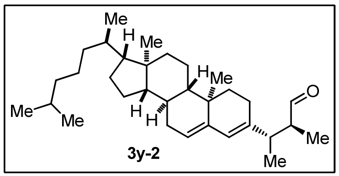

3y-2

(*2S,3S*)-3-((*8S,9S,10R,13R,14S,17R*)-10,13-dimethyl-17-((*R*)-6-methylheptan-2-yl)-2,7,8,9,10,11,12,13,14,15,16,17-dodecahydro-1H-cyclopenta[*a*]phenanthren-3-yl)-2-methylbutanal (**3y-2**): The general procedure A was followed using (*S*)-4-(tert-butyl)-2-(5-(trifluoromethyl)pyridin-2-yl)-4,5-dihydrooxazole (10.2 mg, 0.0375 mmol), (*8S,9S,10R,13R,14S,17R*)-10,13-dimethyl-17-((*R*)-6-methylheptan-2-yl)-2,7,8,9,10,11,12,13,14,15,16,17-dodecahydro-*1H*-cyclopenta[*a*]phenanthren-3-yl trifluoromethanesulfonate (**1y**, 129 mg, 0.25 mmol) and (*E*)-2-methylbut-2-en-1-ol (**2a**, 86 mg, 1.0 mmol). Purification of this material by chromatography on silica gel (1:30 EtOAc:hexanes) afforded product **3y-2** as a white waxy solid (23 mg, 22% yield): R_f = 0.70 (1:4 EtOAc:hexanes); **^{1}H NMR** ($CDCl_3$, 400 MHz) δ 9.56 (d, *J* =

2.0 Hz, 1H), 5.77 (s, 1H), 5.36 (s, 1H), 2.53-1.70 (m, 7H), 1.61-1.43 (m, 5H), 1.35-1.20 (m, 6H), 1.17-0.98 (m, 15 H), 0.93-0.85 (m, 13 H), 0.7 (s, 3H); **^{13}C NMR** ($CDCl_3$, 100 MHz) δ 205.2, 141.4, 137.9, 125.5, 122.8, 57.0, 56.2, 49.5, 48.3, 42.5, 41.6, 39.8, 39.5, 36.2, 35.8, 35.0, 34.2, 31.8, 28.2, 28.0, 24.2, 23.8, 23.4, 22.8, 22.6, 21.1, 18.8, 18.7, 16.1, 12.0, 11.0 ppm; **IR** (neat) 2933, 2868, 1726, 1466, 1457, 1380 cm^{-1}; **HRMS** (ESI-TOF) *m/z* calcd for $C_{32}H_{53}O$ $(M + H)^+$: 453.4096, found 453.4098; 1:2.4 dr; $[\alpha]_D^{20}$ = –134 (c = 0.1, EtOH).

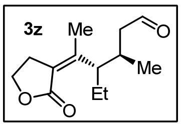

(*3R,4S,Z*)-4-ethyl-3-methyl-5-(2-oxodihydrofuran-3(2H)-ylidene)hexanal (**3z**): The general procedure A was followed using (*Z*)-1-(2-oxodihydrofuran-3(2H)-ylidene)ethyl trifluoromethanesulfonate (**1z**, 65 mg, 0.25 mmol) and (*E*)-3-methylhex-3-en-1-ol (**2z**, 57 mg, 0.5 mmol). Purification of this material by chromatography on silica gel (1:4 EtOAc:hexanes) afforded product **3z** as a yellow liquid (17 mg, 31% yield): R_f = 0.10 (1:4 EtOAc:hexanes); **^{1}H NMR** ($CDCl_3$, 500 MHz) δ 9.70 (d, *J* = 2.5 Hz, 1H), 4.34-4.29 (m, 2H), 3.96 (t, *J* = 10.0 Hz, 1H), 2.88 (t, *J* = 7.5 Hz, 2H), 2.37-2.09 (m, 3H), 1.88-1.72 (m, 4H), 1.31-1.21 (m, 1H), 1.06 (d, *J* = 5.0 Hz, 3H), 0.75 (t, *J* = 7.5 Hz, 3H); **^{13}C NMR** ($CDCl_3$, 100 MHz) δ 202.6, 170.1, 155.6, 122.3, 64.2, 49.7, 45.2, 30.8, 28.1, 22.6, 18.4, 16.5, 11.9 ppm; **IR** (neat) 2964, 2875, 1736, 1722, 1188, 1074 cm^{-1}; **HRMS** (ESI-TOF) *m/z* calcd for $C_{13}H_{20}O_3Na$ $(M + Na)^+$ 247.1310, found 247.1311; 89:11 er, 6:1 dr; $[\alpha]_D^{20}$ = –9 (c = 0.1, EtOH).

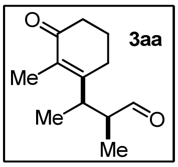

(*2S,3R*)-2-methyl-3-(2-methyl-3-oxocyclohex-1-en-1-yl)butanal (**3aa**): The general procedure A was followed using 2-methyl-3-oxocyclohex-1-en-1-yl trifluoromethanesulfonate (**1a**, 65 mg, 0.25 mmol) and (*Z*)-2-methylbut-2-en-1-ol (**2aa**, 43 mg, 0.5 mmol). Purification of this material by chromatography on silica gel (1:4 EtOAc:hexanes) afforded product **3aa** as a yellow liquid (33 mg, 68% yield): R_f = 0.20 (1:4 EtOAc:hexanes); **^{1}H NMR** ($CDCl_3$, 400 MHz) δ 9.66 (d, *J* = 2.0 Hz, 1H), 3.19-3.09 (m, 1H), 2.45-2.39 (m, 3H), 2.33-2.13 (m, 2H), 1.98-1.91 (m, 2H), 1.84 (s, 3H), 1.10 (d, *J* = 8.0 Hz, 3H), 0.98 (d, *J* = 8.0 Hz, 3H); **^{13}C NMR** ($CDCl_3$, 100 MHz) δ 203.7, 199.2, 158.3, 132.2, 49.7, 38.0, 37.8, 25.2, 22.6, 16.6, 12.8, 10.8 ppm; **IR** (neat) 2933, 2871, 1723, 1660, 1625, 1457,cm^{-1}; **HRMS** (ESI-TOF) *m/z* calcd for $C_{12}H_{18}O_2Na$ $(M + Na)^+$: 217.1204, found 217.1206; 57:43 er, 20:1 dr; $[\alpha]_D^{20}$ = -17 (c = 0.1, EtOH).

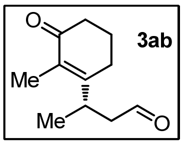

(*S*)-3-(2-methyl-3-oxocyclohex-1-en-1-yl)butanal (**3ab**): The general procedure A was followed using 2-methyl-3-oxocyclohex-1-en-1-yl trifluoromethanesulfonate (**1a**, 65 mg, 0.25 mmol) and (*E*)-but-2-en-1-ol (**2ab**, 36 mg, 0.50 mmol). Purification of this material by chromatography on silica gel (1:4 EtOAc:hexanes) afforded product **3ab** as a yellow liquid (28 mg, 62% yield): R_f = 0.20 (1:4 EtOAc:hexanes); **^{1}H NMR** ($CDCl_3$, 500 MHz) δ 9.72 (d, *J* = 2.5 Hz, 1H); 3.51-3.42 (m, 1H), 2.56 (d, *J* = 5.0 Hz, 2H), 2.40 (t, *J* = 6.0 Hz, 2H), 2.32-2.12 (m, 2H), 1.95-1.89 (m, 2H), 1.84 (s, 3H), 1.13 (d, *J* = 5.0 Hz, 3H); **^{13}C NMR** ($CDCl_3$, 125 MHz) δ 200.4, 199.3, 159.3, 130.8, 48.0, 37.8, 31.5, 25.2, 22.5, 18.0, 10.3 ppm; **IR** (neat) 2935, 1722, 1661, 1617, 1457, 1327 cm^{-1}; **HRMS** (ESI-TOF) *m/z* calcd for $C_{11}H_{16}O_2Na$ $(M + Na)^+$: 203.1048, found 203.1043; 89:11 er; $[\alpha]_D^{20}$ = +29 (c = 0.1, EtOH).

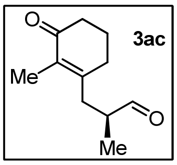
3ac

(*S*)-2-methyl-3-(2-methyl-3-oxocyclohex-1-en-1-yl)propanal (**3ac**): The general procedure A was followed using 2-methyl-3-oxocyclohex-1-en-1-yl trifluoromethanesulfonate (**1a**, 65 mg, 0.25 mmol) and 2-methylprop-2-en-1-ol (**2ac**, 36 mg, 0.5 mmol). Purification of this material by chromatography on silica gel (1:4 EtOAc:hexanes) afforded product **3ac** as a yellow liquid (40 mg, 89% yield): R_f = 0.20 (1:4 EtOAc:hexanes); **^{1}H NMR** ($CDCl_3$, 500 MHz) δ 9.68 (d, *J* = 2.5 Hz, 1H), 2.67-2.61 (m, 2H), 2.43-2.32 (m, 5H), 1.99-1.92 (m, 2H), 1.79 (s, 3H), 1.15 (d, *J* = 5.0 Hz, 3H); **^{13}C NMR** ($CDCl_3$, 125 MHz) δ 203.2, 199.0, 154.8, 132.8, 44.9, 37.7, 35.6, 30.9, 22.3, 13.4, 11.1 ppm; **IR** (neat) 2933, 2871, 1723, 1660, 1625, 1457 cm^{-1}; **HRMS** (ESI-TOF) *m/z* calcd for $C_{11}H_{16}O_2Na$ $(M + Na)^+$: 203.1048, found 203.1051; 79:21 er; $[\alpha]_D^{20}$ = -17 (c = 0.1, EtOH)

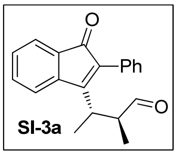
SI-3a

(*2S,3S*)-2-methyl-3-(1-oxo-2-phenyl-1H-inden-3-yl)butanal (**SI-3a**): The general procedure A was followed using 1-oxo-2-phenyl-*1H*-inden-3-yl trifluoromethanesulfonate (**SI-1a**, 89 mg, 0.25 mmol) and (*E*)-2-methylbut-2-en-1-ol (**2a**, 43 mg, 0.5 mmol). Purification of this material by chromatography on silica gel (1:4 EtOAc:hexanes) afforded product **SI-3a** as a yellow liquid (52 mg, 72% yield): R_f = 0.40 (1:4 EtOAc:hexanes); **^{1}H NMR** ($CDCl_3$, 400 MHz) δ 9.43 (d, *J* = 4.0 Hz, 1H), 7.52 (d, *J* = 8.0 Hz, 1H), 7.45-7.35 (m, 4H), 7.30-7.23 (m, 4H), 3.30-3.22 (m, 1H), 2.90-2.83 (m, 1H), 1.45 (d, *J* = 8.0 Hz, 3H), 1.16 (d, *J* = 8.0 Hz, 3H); **^{13}C NMR** ($CDCl_3$, 100 MHz) δ 202.9, 196.2, 159.5, 143.3, 134.5, 133.5, 131.1, 130.96, 129.6, 128.9, 128.4, 128.1, 122.9, 121.5, 49.2, 34.5, 16.5, 12.3 ppm; **IR** (neat) 2974, 2935, 1707, 1456, 753, 701 cm^{-1}; **HRMS** (ESI-TOF) *m/z* calcd for $C_{20}H_{18}O_2Na$ $(M + Na)^+$: 313.1204, found 313.1204; 88:12 er, >20:1 dr; $[\alpha]_D^{20}$ = +83.7 (c = 0.1, EtOH).

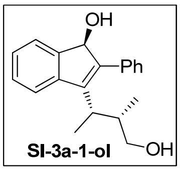
SI-3a-1-ol

(*R*)-3-((*2S,3S*)-4-hydroxy-3-methylbutan-2-yl)-2-phenyl-1H-inden-1-ol (**SI-3a-1-ol**): The general procedure B was followed using **SI-3a** (58 mg, 0.20 mmol). Purification of this material by chromatography on silica gel (1:1 EtOAc:hexanes) afforded product **SI-3a-1-ol** as a yellow liquid (24 mg, 41% yield): R_f = 0.30 (1:2 EtOAc:hexanes); **^{1}H NMR** ($CDCl_3$, 500 MHz) δ 7.57 (d, *J* = 10.0 Hz, 1H), 7.46-7.41 (m, 3H), 7.35-7.31 (m, 3H), 7.29-7.21 (m, 2H), 5.45 (s, 1H), 3.31 (d, *J* = 10.0 Hz, 1H), 3.10-3.07 (m, 1H), 2.83-2.78 (m, 1H), 2.15-2.09 (m, 1H), 1.47 (d, *J* = 5.0 Hz, 3H), 1.27-1.23 (m, 1H), 1.02 (d, *J* = 5.0 Hz, 3H); **^{13}C NMR** ($CDCl_3$, 125 MHz) δ 145.3, 145.0, 143.0, 141.7, 134.9, 129.0, 128.8, 128.3, 127.6, 125.9, 123.8, 121.9, 77.7, 66.8, 38.5, 34.8, 17.1, 15.3 ppm; **IR** (neat) 2967, 2930, 2875, 1458, 1017, 731, 699 cm^{-1}; **HRMS** (ESI-TOF) *m/z* calcd for $C_{20}H_{22}O_2Na$ $(M + Na)^+$: 317.1517, found 317.1517; $[\alpha]_D^{20}$ = +148 (c = 0.1, EtOH).

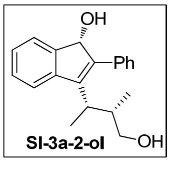
SI-3a-2-ol

(S)-3-((2S,3S)-4-hydroxy-3-methylbutan-2-yl)-2-phenyl-1H-inden-1-ol (**SI-3a-2-ol**): The general procedure B was followed using **SI-3a** (58 mg, 0.20 mmol). Purification of this material by chromatography on silica gel (1:1 EtOAc:hexanes) afforded product **SI-3a-2-ol** as a yellow liquid (14 mg, 25% yield): R_f = 0.20 (1:2 EtOAc:hexanes); **^{1}H NMR** ($CDCl_3$, 500 MHz) δ 7.54 (d, *J* = 5.0 Hz, 1H), 7.43-7.35 (m, 5H), 7.33-7.32 (m, 1H), 7.30-7.27 (m, 1H), 7.25-7.20 (m, 1H), 5.27 (s, 1H), 3.51 (d, *J* = 10.0 Hz, 1H), 3.36-3.31 (m, 1H), 7.72-7.60 (m, 1H), 2.20-2.14 (m, 1H), 1.27-1.23 (m, 4H), 1.05 (d, *J* = 10.0 Hz, 3H); **^{13}C NMR** ($CDCl_3$, 125 MHz) δ 145.3, 144.0, 143.4,

141.9, 135.7, 129.1, 128.5, 128.3, 127.5 125.9, 123.7, 121.6, 78.6, 66.9, 38.7, 35.2, 17.4, 15.5 ppm; **IR** (neat) 2965, 2930, 1458, 1011, 731, 701 cm^{-1}; **HRMS** (ESI-TOF) *m/z* calcd for $C_{20}H_{22}O_2Na$ (M + Na)$^+$: 317.1517, found 317.1524; $[\alpha]_D^{20}$ = –63 (c = 0.1, EtOH).

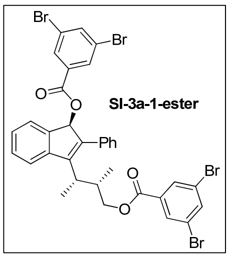

(2S,3S)-3-((R)-1-((3,5-dibromobenzoyl)oxy)-2-phenyl-1H-inden-3-yl)-2-methylbutyl 3,5-dibromobenzoate (**SI-3a-1-ester**): The general procedure C was followed using **SI-3a-1-ol** (29 mg, 0.10 mmol). Purification of this material by chromatography on silica gel (1:10 EtOAc:hexanes) afforded product **SI-3a-1-ester** as a yellow solid (73 mg, 90% yield): R_f = 0.50 (1:5 EtOAc:hexanes); ^{1}**H NMR** ($CDCl_3$, 400 MHz) δ 7.88 (s, 2H), 7.75 (t, *J* = 8.0 Hz, 2H), 7.60 (d, *J* = 2.0 Hz, 2H), 7.51-7.43 (m, 1H), 7.41-7.36 (m, 1H), 7.34-7.32 (m, 1H), 7.21-7.12 (m, 5H), 7.04 (t, *J* = 6.0 Hz, 1H), 6.94 (s, 1H), 4.05 (d, *J* = 12.0 Hz, 1H), 3.88 (d, *J* = 4.0 Hz, 1H), 3.21-3.13 (m, 1H), 2.57-2.45 (m, 1H), 1.62 (d, *J* = 8.0 Hz, 3H), 1.18 (d, *J* = 8.0 Hz, 3H); ^{13}**C NMR** ($CDCl_3$, 100 MHz) δ 164.4, 163.7, 144.6, 142.5, 141.8, 141.7, 138.2, 137.8, 133.7, 133.1, 133.0, 131.4, 130.8, 128.9, 128.5, 128.2, 127.7, 126.2, 124.6, 122.8, 122.7, 122.0, 78.1, 69.3, 35.4, 35.2, 17.0, 16.0 ppm; **IR** (neat) 2969, 1722, 1559, 1258, 763, 747 cm^{-1}; **HRMS** (ESI-TOF) *m/z* calcd for $C_{34}H_{26}O_2NaBr_4$ (M + Na)$^+$: 836.8462, found 836.8481; $[\alpha]_D^{20}$ = +47 (c = 0.1, EtOH).

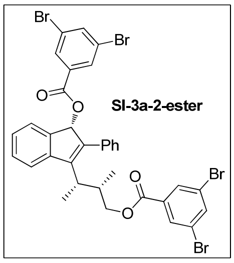

(2S,3S)-3-((S)-1-((3,5-dibromobenzoyl)oxy)-2-phenyl-1H-inden-3-yl)-2-methylbutyl 3,5-dibromobenzoate (**SI-3a-2-ester**): The general procedure C was followed using **SI-3a-2-ol** (29 mg, 0.10 mmol). Purification of this material by chromatography on silica gel (1:10 EtOAc:hexanes) afforded product **SI-3a-2-ester** as a yellow solid (73 mg, 89% yield): R_f = 0.50 (1:5 EtOAc:hexanes); ^{1}**H NMR** ($CDCl_3$, 400 MHz) δ 8.06 (d, *J* = 2.0 Hz, 2H), 7.84 (d, *J* = 2.0 Hz, 1H), 7.75-7.70 (m, 3H), 7.49-7.45 (m, 2H), 7.35-7.31 (m, 1H), 7.22-7.21 (m, 4H), 6.61 (s, 1H), 4.22 (s, 2H), 2.91 (t, *J* = 8.0 Hz, 1H), 2.61-2.51 (m, 1H), 1.43 (d, *J* = 8.0 Hz, 3H), 1.17 (d, *J* = 4.0 Hz, 3H) ; ^{13}**C NMR** ($CDCl_3$, 100 MHz) δ 164.4, 163.9, 146.1, 142.6, 141.7, 140.0, 138.5, 137.9, 134.9, 133.3, 133.1, 131.5, 131.0, 129.0, 128.6, 128.4, 127.8, 126.4, 124.9, 123.1, 122.7, 121.7, 79.6, 69.8, 36.0, 35.7, 17.3, 16.1 ppm; **IR** (neat) 2967, 1723, 1558, 1253, 762, 742 cm^{-1}; **HRMS** (ESI-TOF) *m/z* calcd for $C_{34}H_{26}O_2NaBr_4$ (M + Na)$^+$: 836.8462, found 836.8481; $[\alpha]_D^{20}$ = +77 (c = 0.1, EtOH).

References for Experimental section:

1. A. D. Fotiadou and A. L. Zografos, *Org. Lett.*, 2011, **13**, 4592.

2. D. A. DeGoey, H.-J. Chen, W. J. Flosi , D. J. Grampovnik, C. M. Yeung, L. L. Klein and Dale J. Kempf, *J. Org. Chem.*, 2002, **67**, 5445.

3. F. F. Fleming, W. Liu, S. Ghosh and O. W. Steward, *J. Org. Chem.*, 2008, **73**, 2803.

4. M. Hatano, T. Miyamoto and K. Ishihara, *J. Org. Chem.*, 2006, **71**, 6474.

5. D. M. Hodgson and R. S. D. Persaud, *Org. Biomol. Chem.*, 2012, **10**, 7949.

6. G. Sudhakar and K. Satish, *Chem. Eur. J.* 2015, **21**, 6475.

7. M. Takahashi, M. McLaughlin and G. C. Micalizio, *Angew. Chem. Int. Ed.* 2009, **48**, 3648.

8. D. C. Miller, G. J. Choi, H. S. Orbe and R. R. Knowles, *J. Am. Chem. Soc.*, 2015, **137**, 13492.

9. Y.-C. Qin and L. Pu, *Angew. Chem. Int. Ed.* 2006, **45**, 273.

10. R. H. Munday, R. M. Denton and J. C. Anderson, *J. Org. Chem.*, 2008, **73**, 8033.

11. K. K. Wan, K. Iwasaki, J. C. Umotoy, D. W. Wolan and R. A. Shenvi, *Angew. Chem. Int. Ed.* 2015, **54**, 2410.

12. D. Niu and T. R. Hoye, *Org. Lett.*, 2012, **14**, 828.

13. M. Reiter, H. Turner, R. Mills-Webb and V. Gouverneur, *J. Org. Chem.*, 2005, **70**, 8478.

14. D. M. Jones, M. P. Lisboa, S. Kamijo and G. B. Dudley, *J. Org. Chem.*, 2010, **75**, 3260.

15. P. von Zezschwitz, F. Petry and A. de Meijere, *Chem. Eur. J.* 2001, **7**, 4035.

16. A. Abad, C. Agullo, A. C. Cunat, D. Jimenez and R. H. Perni, *Tetrahedron*, 2001, **57**, 9727.

17. R. H. Pouwer, H. Schill, C. M. Williams and P. V. Bernhardt, *Eur. J. Org. Chem.* 2007, 4699.

18. D. Babinski, O. Soltani and Doug E. Frantz, *Org. Lett.*, 2008, **10**, 2901.

19. V. Saini, M. O'Dair and M. S. Sigman, *J. Am. Chem. Soc.*, 2015, **137**, 608.

20. E. W. Werner, T.-S. Mei, A. J. Burckle and M. S. Sigman, *Science*, 2012, **338**, 1455.

21. J. A. Schiffner, T. H. Wöste and M. Oestreich, *Eur. J. Org. Chem.* 2010, 174.

22. E.C. Sherer, C.H. Lee, J. Shpungin, J.F. Cuff, C.X. Da, R. Ball, R. Bach, A. Crespo, X.Y. Gong, and C.J. Welch, *J. Med. Chem.*, 2014, **57**, 477.

23. G.A. Petersson, and M.A. Allaham, *J. Chem. Phys.*, 1991, **94**, 6081.

24. G.A. Petersson, A. Bennett, T.G. Tensfeldt, M.A. Allaham, W.A. Shirley, and J. Mantzaris, *J. Chem. Phys.*, 1988, **89**, 2193.

25. V.A. Rassolov, J.A. Pople, M.A. Ratner, and T.L. Windus, *J. Chem. Phys.*, 1998, **109**, 1223.

26. V.A. Rassolov, M.A. Ratner, J.A. Pople, P.C. Redfern, and L.A. Curtiss, *J. Comput. Chem.*, 2001, **22**, 976.

27. M.M. Francl, W.J. Pietro, W.J. Hehre, J.S. Binkley, M.S. Gordon, D.J. Defrees, and J.A. Pople, *J. Chem. Phys.*, 1982, **77**, 3654.

28. W.J. Hehre, R. Ditchfield, and J.A. Pople, *J. Chem. Phys.*, 1972, **56**, 2257.

29. A.D. Becke, *J. Chem. Phys.*, 1993, **98**, 5648.

30. C.T. Lee, W.T. Yang, and R.G. Parr, *J. Phys. Chem. B*, 1988, **37**, 785.

31. B. Miehlich, A. Savin, H. Stoll, and H. Preuss, *Chem. Phys. Lett.*, 1989, **157**, 200.

32. M.J. Frisch, G.W. Trucks, H.B. Schlegel, G.E. Scuseria, M.A. Robb, J.R. Cheeseman, G. Scalmani, V. Barone, B. Mennucci, G.A. Petersson, H. Nakatsuji, M. Caricato, X. Li, H.P. Hratchian, A.F. Izmaylov, J. Bloino, G. Zheng, J.L. Sonnenberg, M. Hada, M. Ehara, K. Toyota, R. Fukuda, J. Hasegawa, M. Ishida, T. Nakajima, Y. Honda, O. Kitao, H. Nakai, T. Vreven, J. Montgomery, J.A., J.E. Peralta, F. Ogliaro, M. Bearpark, J.J. Heyd, E. Brothers, K.N. Kudin, V.N. Staroverov, R. Kobayashi, J. Normand, K. Raghavachari, A. Rendell, J.C. Burant, S.S. Iyengar, J.

Tomasi, M. Cossi, N. Rega, J.M. Millam, M. Klene, J.E. Knox, J.B. Cross, V. Bakken, C. Adamo, J. Jaramillo, R. Gomperts, R.E. Stratmann, O. Yazyev, A.J. Austin, R. Cammi, C. Pomelli, J.W. Ochterski, R.L. Martin, K. Morokuma, V.G. Zakrzewski, G.A. Voth, P. Salvador, J.J. Dannenberg, S. Dapprich, A.D. Daniels, Ö. Farkas, J.B. Foresman, J.V. Ortiz, J. Cioslowski, and D.J. Fox. Gaussian 09, Revision A.02. Wallingford, CT: Gaussian, Inc.; 2009.

33. T. Yanai, D. P. Tew and N. C. Handy. *Chem. Phys. Lett.* 2004, **393**, 51.

34. M. J. Frisch, C. J. Cramer and D. G. Truhlar, *J. Phys. Chem. B,* 2009, **113**, 6378.

35. T. Clark, J. Chandrasekhar, G. W. Spitznagel and P. v. R. Schleyer, *J. Comp. Chem.* 1983, **4**, 294.

36. R. Bauernschmitt and R. Ahlrichs, *Chem. Phys. Lett.* 1996, **256**, 454.

37. T. Bruhn, A. Schaumloeffel, Y. Hemberger and G. Bringmann, *Chirality*, 2013, **25**, 243.

38. G. Mazzeo, E. Santoro, A. Andolfi, A. Cimmino, P. Trosejl, A. G. Petrovic and S. Superchi, *J. Nat. Prod.* 2013, **76**, 588.

Enantiomeric ratio of products

Entry	Compound	Conditions	Retention time (min)	er
1	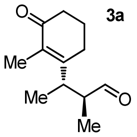 3a	HPLC, Column Whelko, 25 °C,<br>Hexane : *i*-PrOH = 95:5, 1mL/min	t_1 = 43.9, t_2 = 49.3	95:5
2	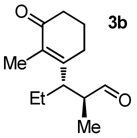 3b	HPLC, Column Whelko, 25 °C,<br>Hexane : *i*-PrOH = 95:5, 1mL/min	t_1 = 44.6, t_2 = 49.7	94:6
3	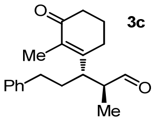 3c	HPLC, Column Whelko, 25 °C,<br>Hexane : *i*-PrOH = 95:5, 1mL/min	t_1 = 67.7, t_2 = 76.2	95:5
4	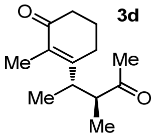 3d	HPLC, Column Whelko, 25 °C,<br>Hexane : *i*-PrOH = 95:5, 1mL/min	t_1 = 50.3, t_2 = 57.3	97:3
5	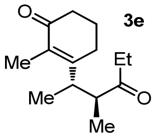 3e	HPLC, Column Whelko, 25 °C,<br>Hexane : *i*-PrOH = 98:2, 1mL/min	t_1 = 61.3, t_2 = 67.5	98:2
6	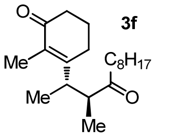 3f	HPLC, Column Whelko, 25 °C,<br>Hexane : *i*-PrOH = 98:2, 1mL/min	t_1 = 134.1, t_2 = 142.5	98:2
7	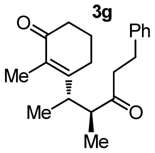 3g	HPLC, Column Whelko, 25 °C,<br>Hexane : *i*-PrOH = 98:2, 1mL/min	t_1 = 71.7, t_2 = 75.9	98:2
8	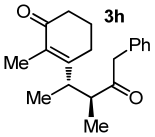 3h	HPLC, Column AY-H, 25 °C,<br>Hexane : *i*-PrOH = 95:5, 1mL/min	t_1 = 39.8, t_2 = 54.5	97:3
9	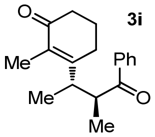 3i	SFC, Column OJ-H, 40 °C,<br>^{i}PrOH:CO_2 = 5:95 to 50:50 (50 min),<br>2mL/min, 180bar,	t_1 = 10.8, t_2 = 19.1	96:4
10	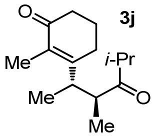 3j	SFC, Column OZ-H, 40 °C,<br>^{i}PrOH:CO_2 = 5:95, 2mL/min, 180bar	t_1 = 14.8, t_2 = 16.8	98:2

11	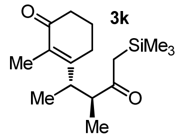 3k	HPLC, Column Whelko, 25 °C, Hexane : *i*-PrOH = 98:2, 1mL/min	t_1 = 56.8, t_2 = 62.6	97:3
12	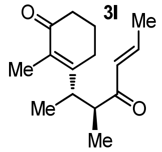 3l	HPLC, Column Whelko, 25 °C, Hexane : *i*-PrOH = 98:2, 1mL/min	t_1 = 106.4, t_2 = 114.0	97:3
13	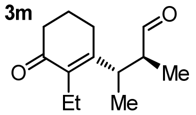 3m	HPLC, Column Whelko, 25 °C, Hexane : *i*-PrOH = 98:2, 1mL/min	t_1 = 49.3, t_2 = 60.4	96:4
14	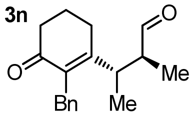 3n	HPLC, Column Whelko, 25 °C, Hexane : *i*-PrOH = 90:10, 1mL/min	t_1 = 52.7, t_2 = 66.9	93:7
15	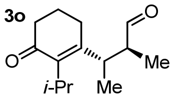 3o	HPLC, Column Whelko, 25 °C, Hexane : *i*-PrOH = 98:2, 1mL/min	t_1 = 31.6, t_2 = 40.2	96:4
16	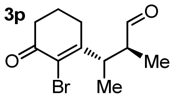 3p	HPLC, Column Whelko, 25 °C, Hexane : *i*-PrOH = 90:10, 1mL/min	t_1 = 38.8, t_2 = 47.0	95:5
17	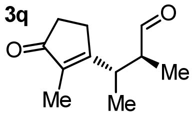 3q	HPLC, Column AS-H, 25 °C, Hexane : *i*-PrOH = 95:5, 0.5mL/min	t_1 = 40.5, t_2 = 45.7	94:6
18	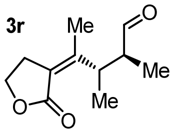 3r	HPLC, Column Whelko, 25 °C, Hexane : *i*-PrOH = 90:10, 1mL/min	t_1 = 43.5, t_2 = 47.8	92:8
19	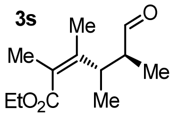 3s	HPLC, Column Whelko, 25 °C, Hexane : *i*-PrOH = 98:2, 1mL/min	t_1 = 11.9, t_2 = 15.1	91:9
20	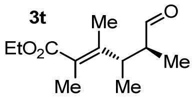 3t	HPLC, Column Whelko, 25 °C, Hexane : *i*-PrOH = 99.8:0.2, 1mL/min	t_1 = 61.6, t_2 = 68.7	97:3
21	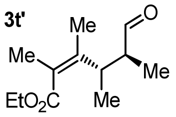 3t'	HPLC, Column Whelko, 25 °C, Hexane : *i*-PrOH = 98:2, 1mL/min	t_1 = 11.2, t_2 = 14.2	91:9
22	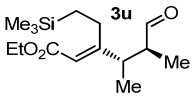 3u	HPLC, Column Whelko, 25 °C, Hexane : *i*-PrOH = 99.9:0.1, 1mL/min	t_1 = 38.4, t_2 = 42.9	90:10
23	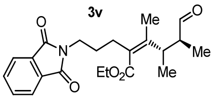 3v	HPLC, Column Whelko, 25 °C, Hexane : *i*-PrOH = 80:20, 1mL/min	t_1 = 36.3, t_2 = 41.0	91:9

24	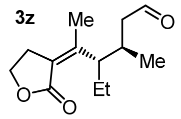 3z	HPLC, Column Whelko, 25 °C, Hexane : *i*-PrOH = 95:5, 1mL/min	t_1 = 72.7, t_2 = 80.3	89:11
25	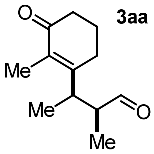 3aa	HPLC, Column AY-H, 25 °C, Hexane : *i*-PrOH = 98:2, 1mL/min	t_1 = 39.0, t_2 = 44.4	57:43
26	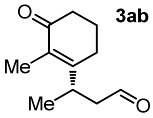 3ab	HPLC, Column AY-H, 25 °C, Hexane : *i*-PrOH = 95:5, 1mL/min	t_1 = 13.7, t_2 = 19.5	89:11
27	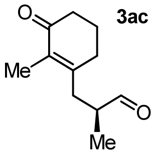 3ac	SFC, Column AY-H, 40 °C, ^{i}-PrOH:CO_2 = 4:96, 2mL/min, 180bar	t_1 = 10.5, t_2 = 11.5	79:21
28	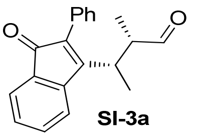 SI-3a	SFC, Column AY-H, 40 °C, ^{i}-PrOH:CO_2 = 4:96, 2mL/min, 180bar	t_1 = 36.9, t_2 = 42.2	88:12

Enantiomeric ratio of products

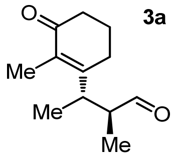

3a

Separation of enantiomers by HPLC, Chiralcel® Column Whelko, 25 °C, Hexane : *i*-PrOH = 95:5, 1mL/min, minor retention time: t_1 = 43.9, major retention time: t_2 = 49.3; er = 95:5.

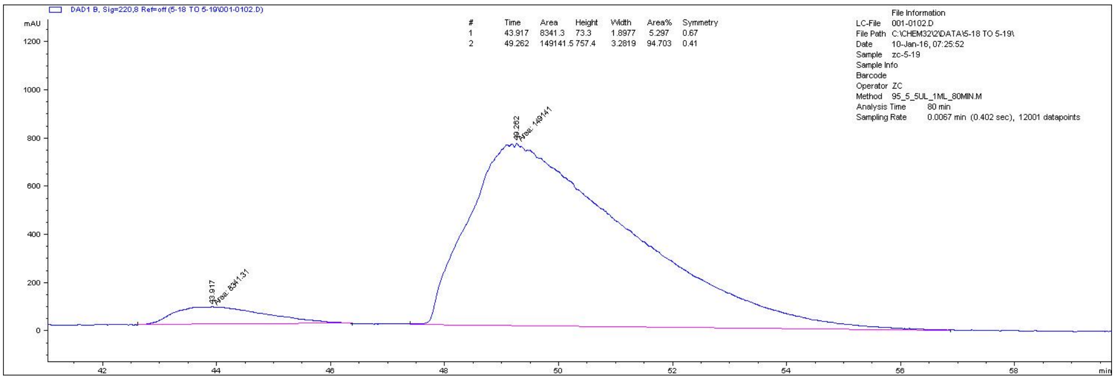

product

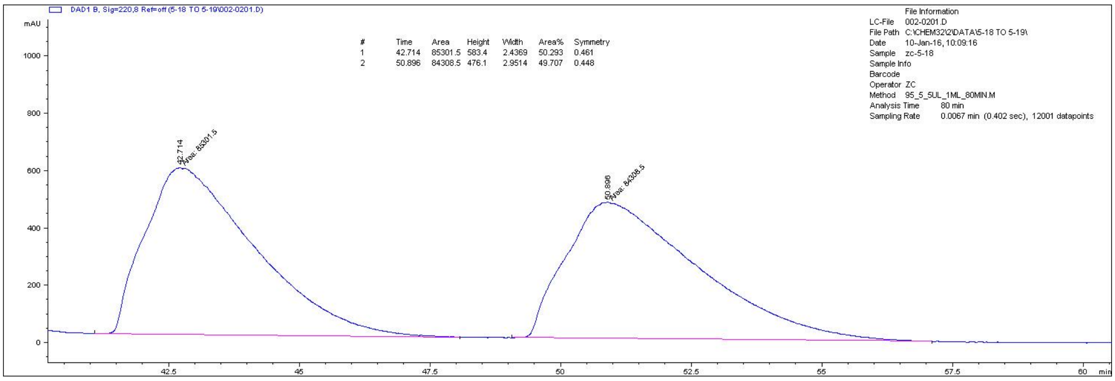

Rac

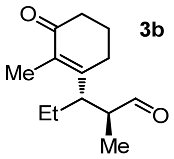

3b

Separation of enantiomers by HPLC, Chiralcel® Column Whelko, 25 ºC, Hexane : *i*-PrOH = 95:5, 1mL/min, minor retention time: t_1 = 44.6, major retention time: t_2 = 49.7; er = 94:6.

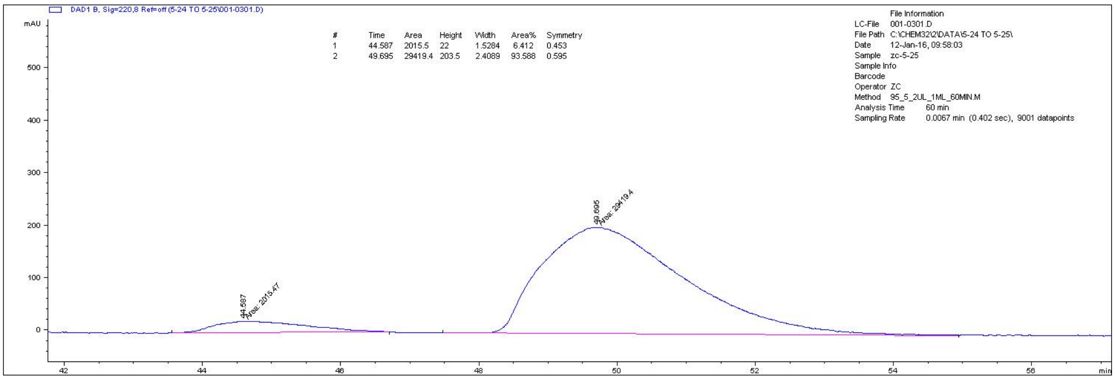

product

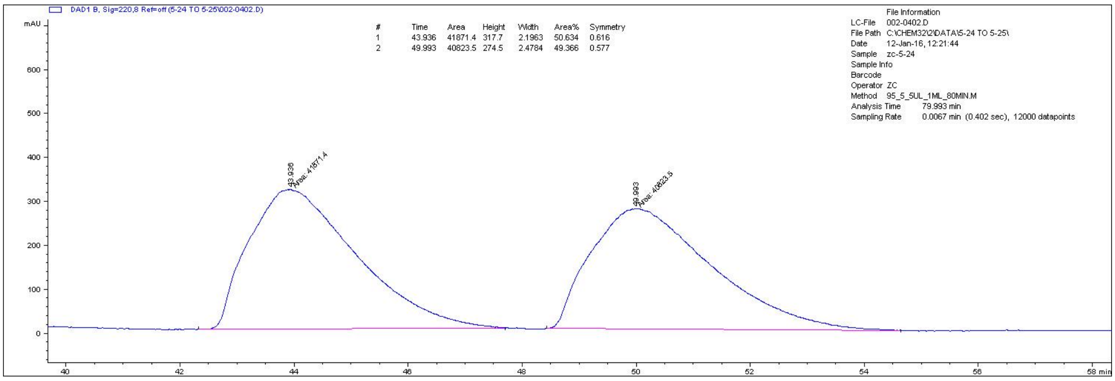

Rac

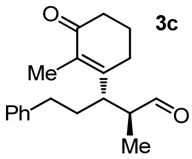

3c

Separation of enantiomers by HPLC, Chiralcel® Column Whelko, 25 ºC, Hexane : *i*-PrOH = 95:5, 1mL/min, minor retention time: t_1 = 67.7, major retention time: t_2 = 76.2; er = 95:5.

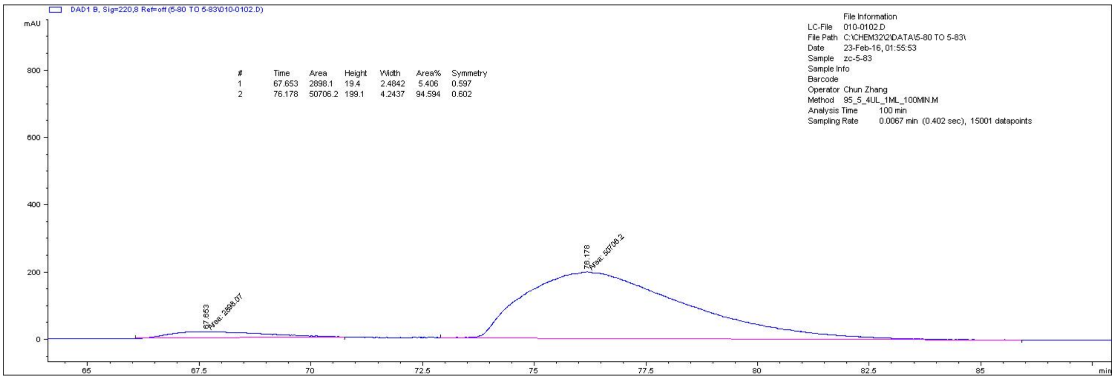

product

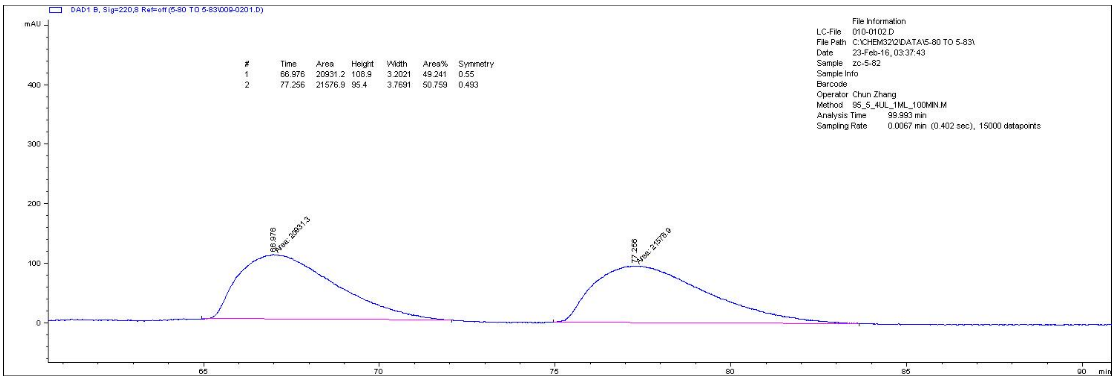

Rac

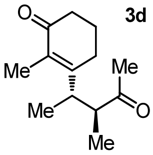

3d

Separation of enantiomers by HPLC, Chiralcel® Column Whelko, 25 °C, Hexane : *i*-PrOH = 95:5, 1mL/min, minor retention time: t_1 = 50.3, major retention time: t_2 = 57.3; er = 97:3.

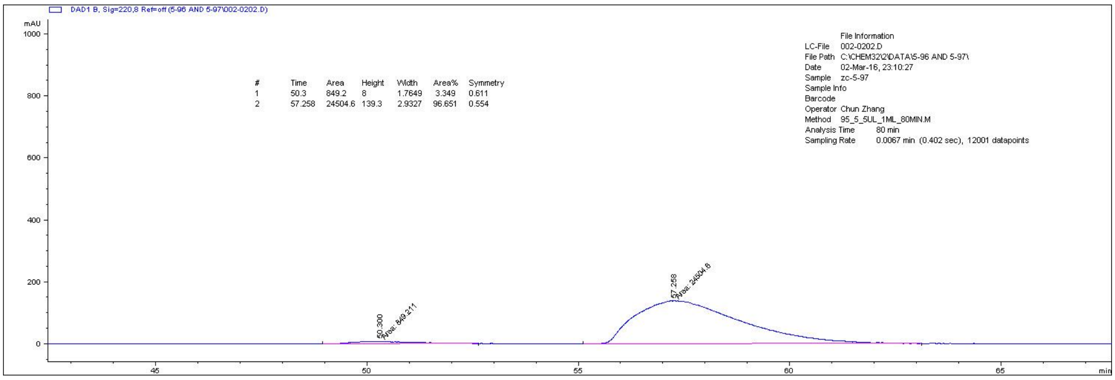

product

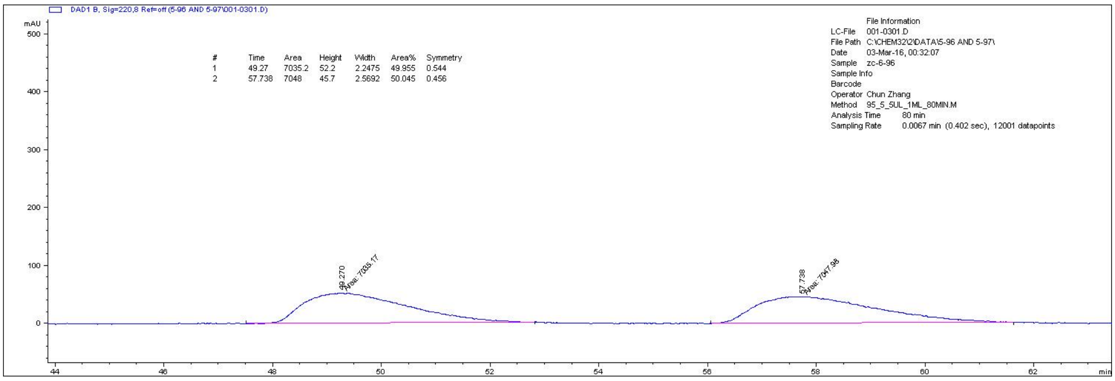

Rac

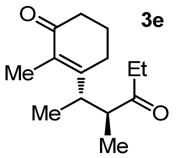

3e

Separation of enantiomers by HPLC, Chiralcel® Column Whelko, 25 °C, Hexane : *i*-PrOH = 98:2, 1mL/min, minor retention time: t_1 = 61.3, major retention time: t_2 = 67.5; er = 98:2.

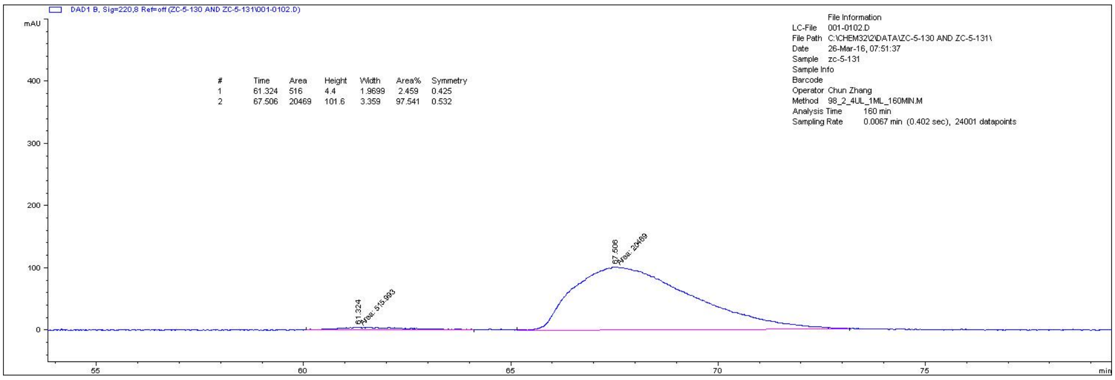

product

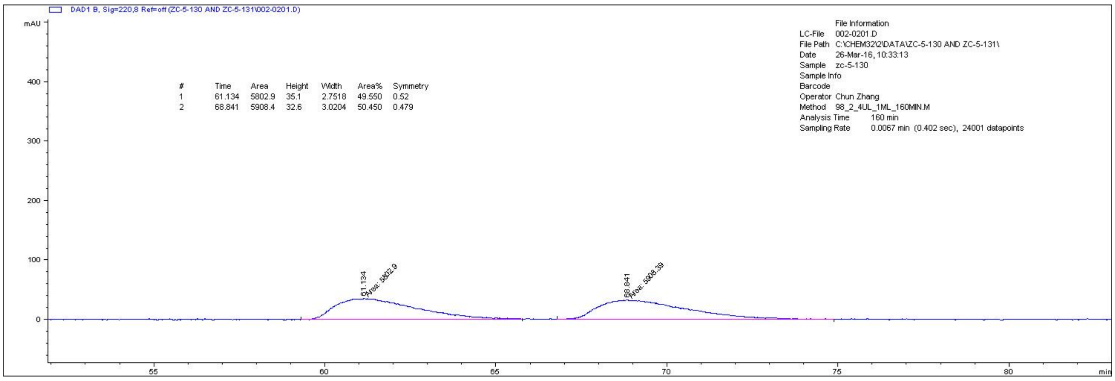

Rac

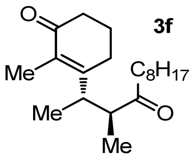

Separation of enantiomers by HPLC, Chiralcel® Column Whelko, 25 °C, Hexane : *i*-PrOH = 98:2, 1mL/min, minor retention time: t_1 = 134.1, major retention time: t_2 = 142.5; er = 98:2.

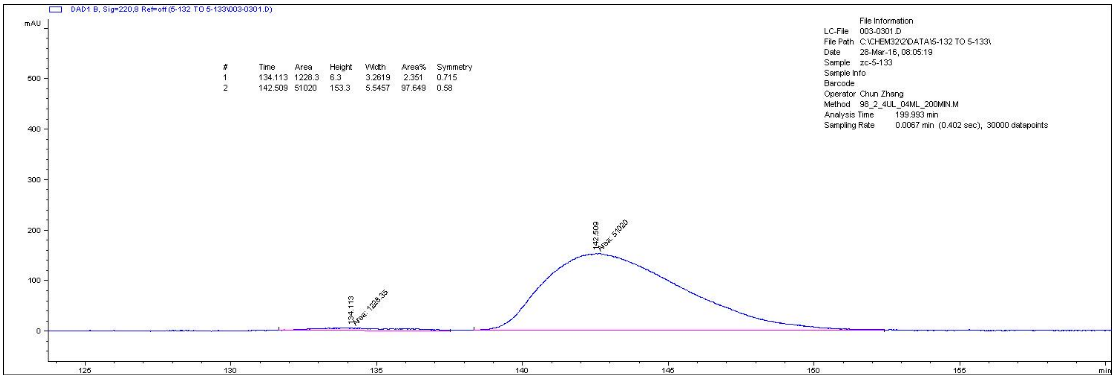

product

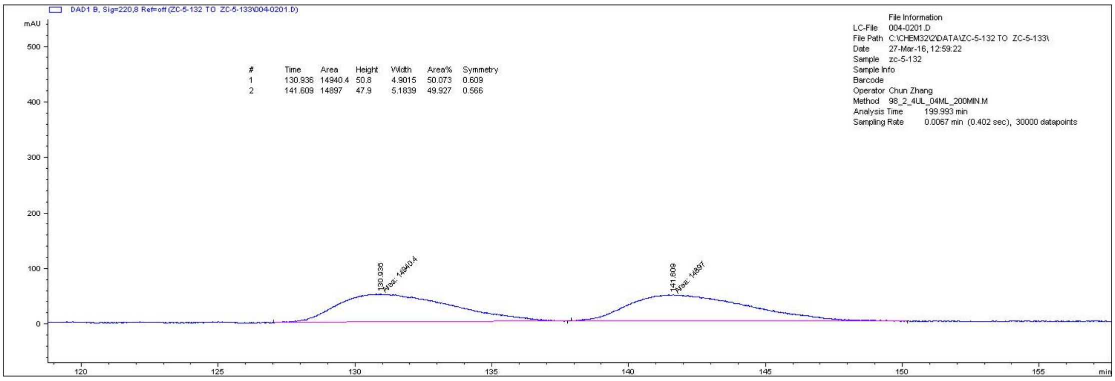

Rac

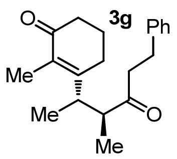

Separation of enantiomers by HPLC, Chiralcel® Column Whelko, 25 °C, Hexane : *i*-PrOH = 98:2, 1mL/min, minor retention time: t_1 = 71.7, major retention time: t_2 = 75.9; er = 98:2.

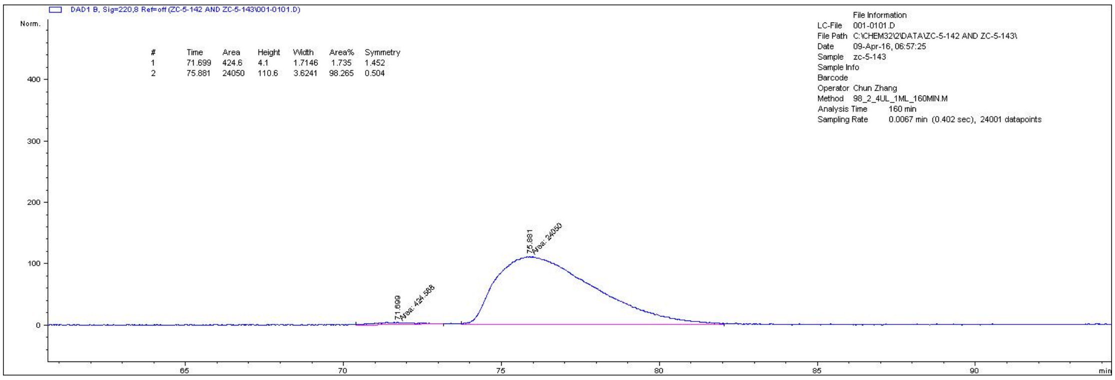

product

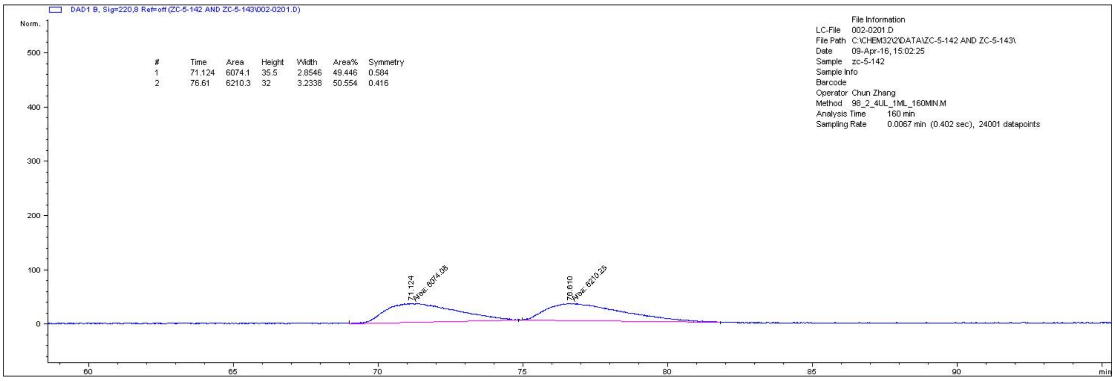

Rac

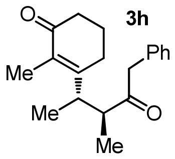

3h

Separation of enantiomers by HPLC, Chiralcel® Column AY-H, 25 ^{o}C, Hexane : *i*-PrOH = 95:5, 1mL/min, minor retention time: t_1 = 39.8, major retention time: t_2 = 54.5; er = 97:3.

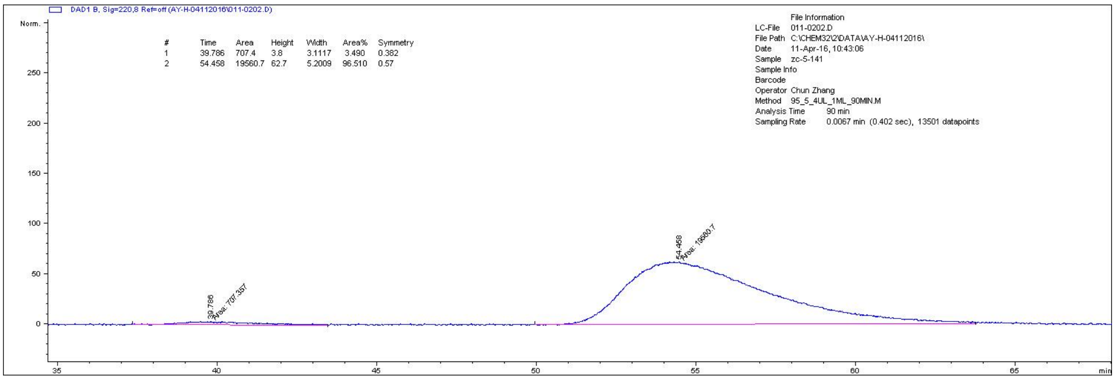

product

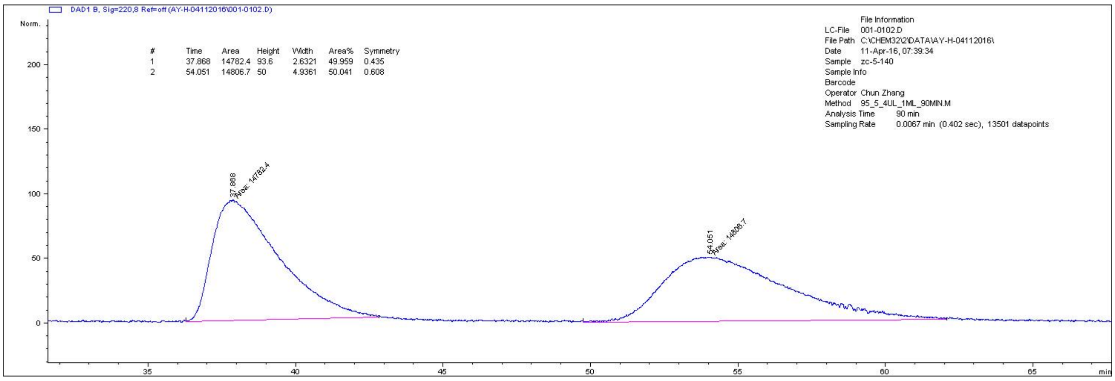

Rac

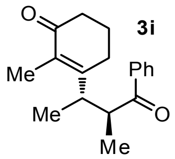

3i

Separation of enantiomers by SFC. Chiralcel® Column OJ-H, 40 °C, $^{i-}$PrOH:CO_2 = 5:95 to 50:50 (50 min), 2mL/min, 180bar, minor retention time: t_1 = 10.8, major retention time: t_2 = 19.1; er = 96:4.

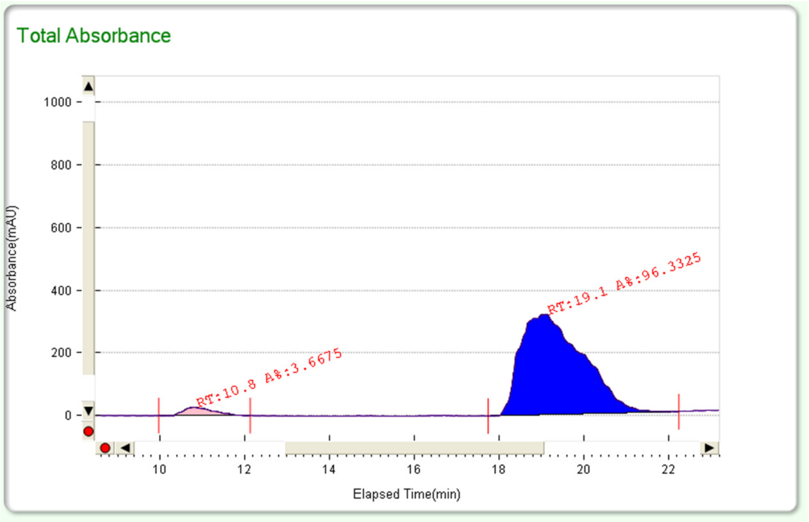

product

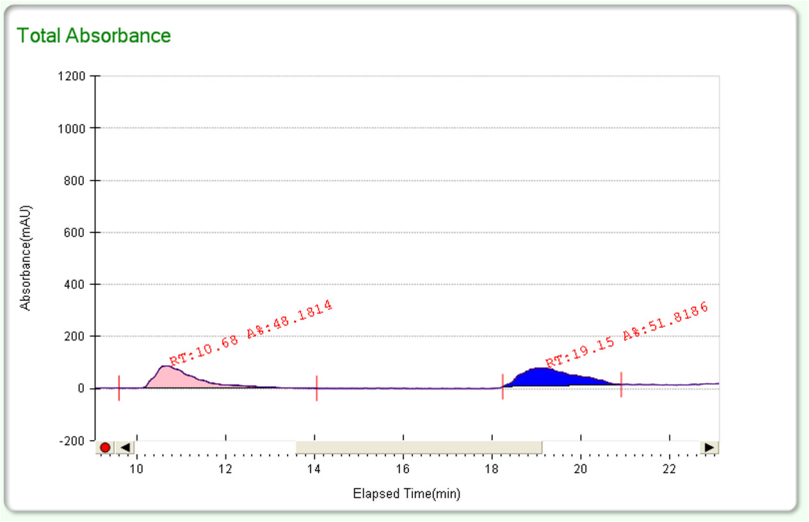

Rac

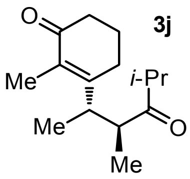

Separation of enantiomers by SFC. Chiralcel® Column OZ-H, 40 °C, ^{i}PrOH:CO_2 = 5:95, 2mL/min, 180bar, minor retention time: t_1 = 14.8, major retention time: t_2 = 16.8; er = 98:2.

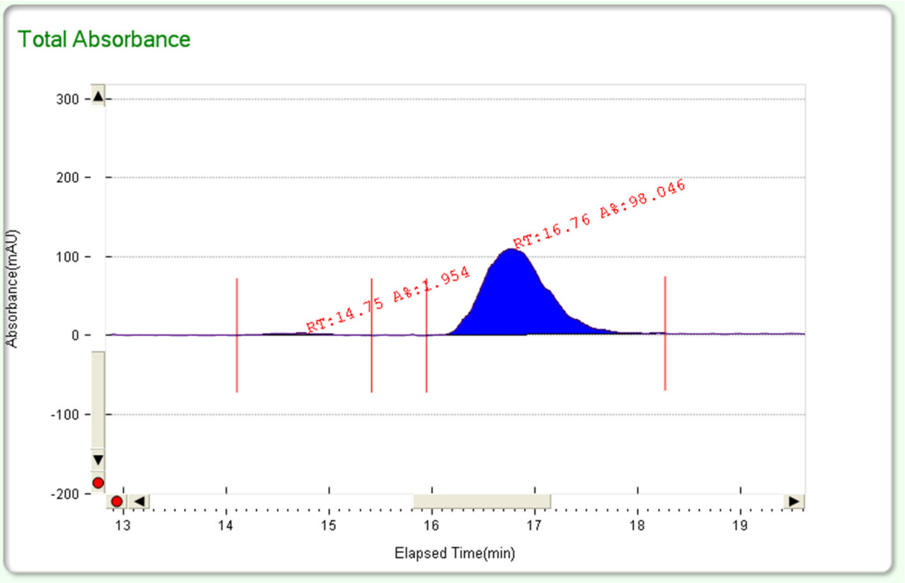

product

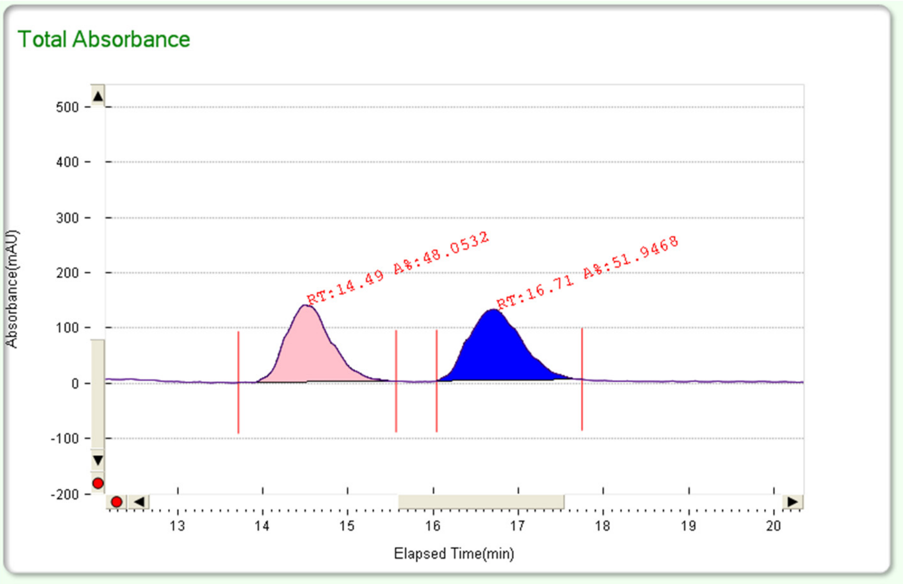

Rac

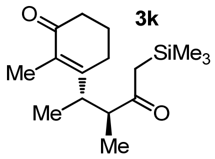

3k

Separation of enantiomers by HPLC, Chiralcel® Column Whelko, 25 °C, Hexane : *i*-PrOH = 98:2, 1mL/min, major retention time: t_1 = 56.8, minor retention time: t_2 = 62.6; er = 97:3.

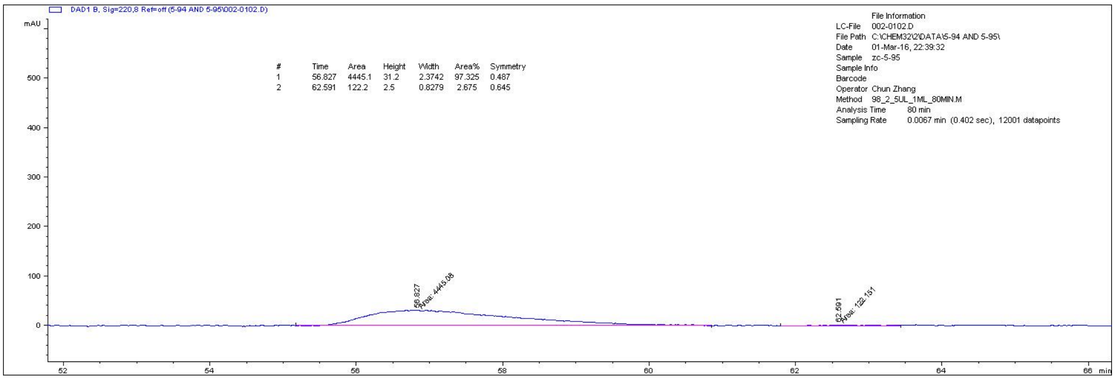

product

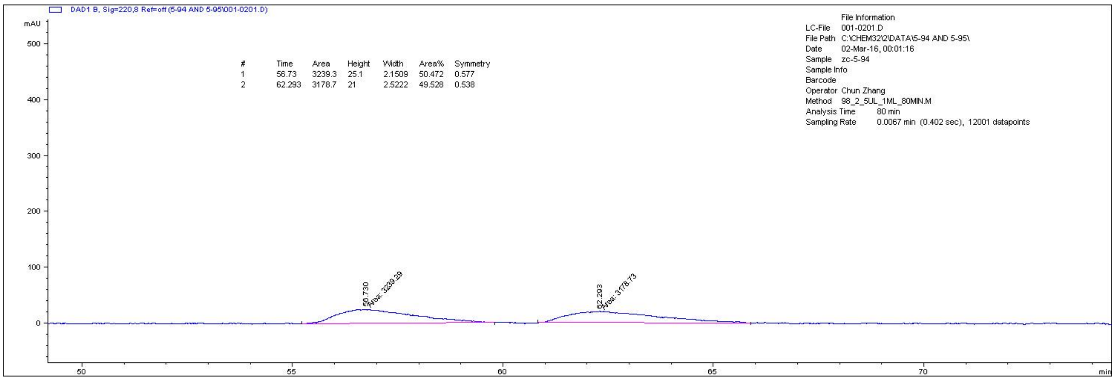

Rac

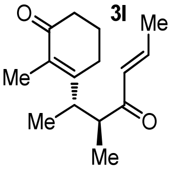

Separation of enantiomers by HPLC, Chiralcel® Column Whelko, 25 ºC, Hexane : *i*-PrOH = 98:2, 1mL/min, minor retention time: t_1 = 106.4, major retention time: t_2 = 114.0; er = 97:3.

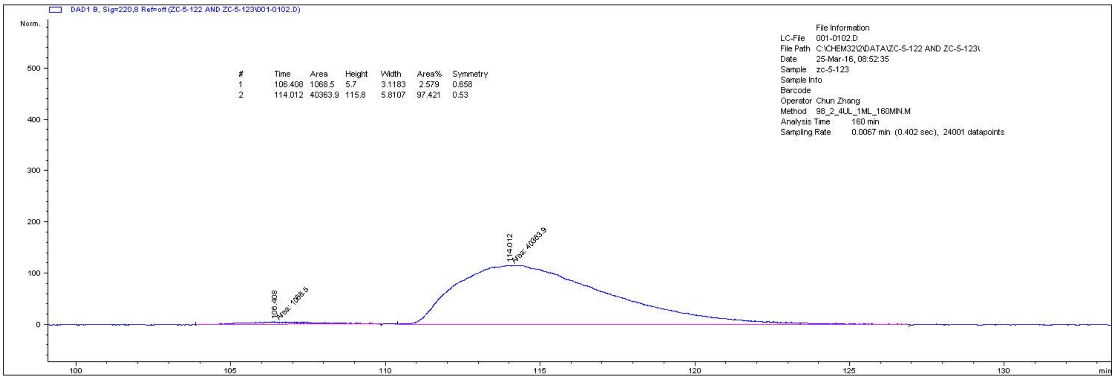

product

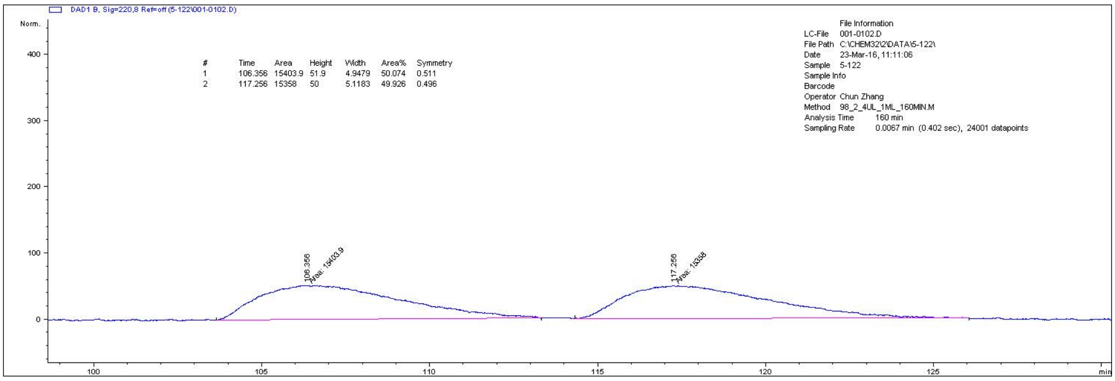

Rac

3m

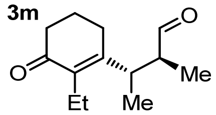

Separation of enantiomers by HPLC, Chiralcel® Column Whelko, 25 °C, Hexane : *i*-PrOH = 98:2, 1mL/min, minor retention time: t_1 = 49.3, major retention time: t_2 = 60.4; er = 96:4.

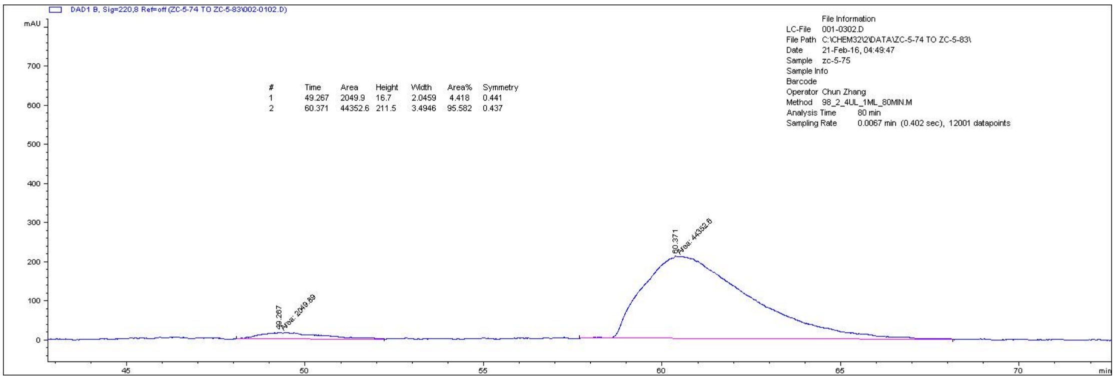

product

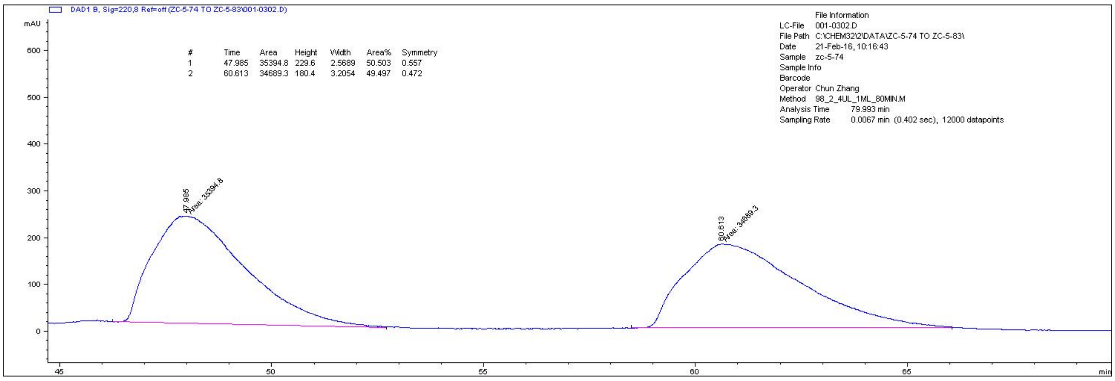

Rac

3n

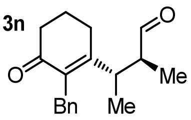

Separation of enantiomers by HPLC, Chiralcel® Column Whelko, 25 °C, Hexane : *i*-PrOH = 90:10, 1mL/min, major retention time: t_1 = 52.7, minor retention time: t_2 = 66.9; er = 93:7.

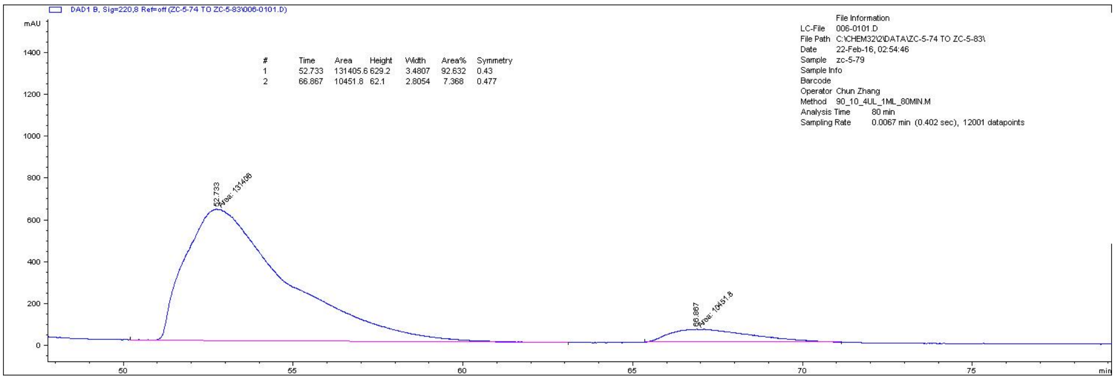

product

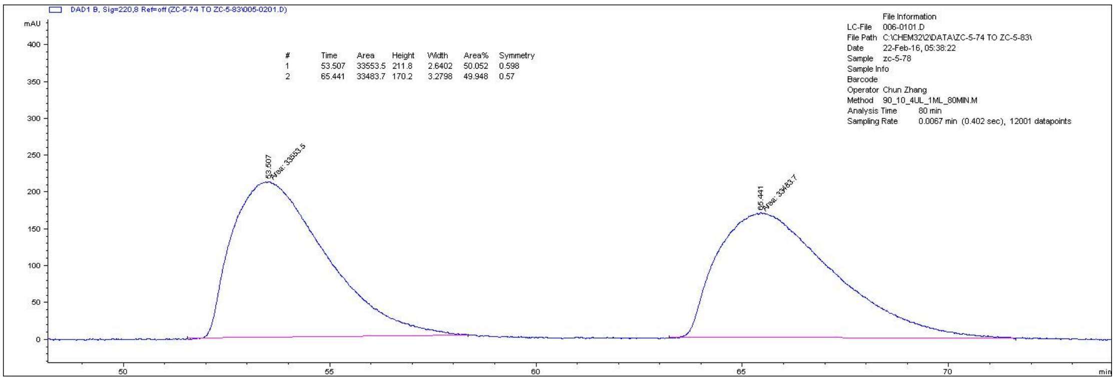

Rac

3o

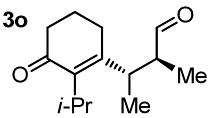

Separation of enantiomers by HPLC, Chiralcel® Column Whelko, 25 °C, Hexane : *i*-PrOH = 98:2, 1mL/min, minor retention time: t_1 = 31.6, major retention time: t_2 = 40.2; er = 96:4.

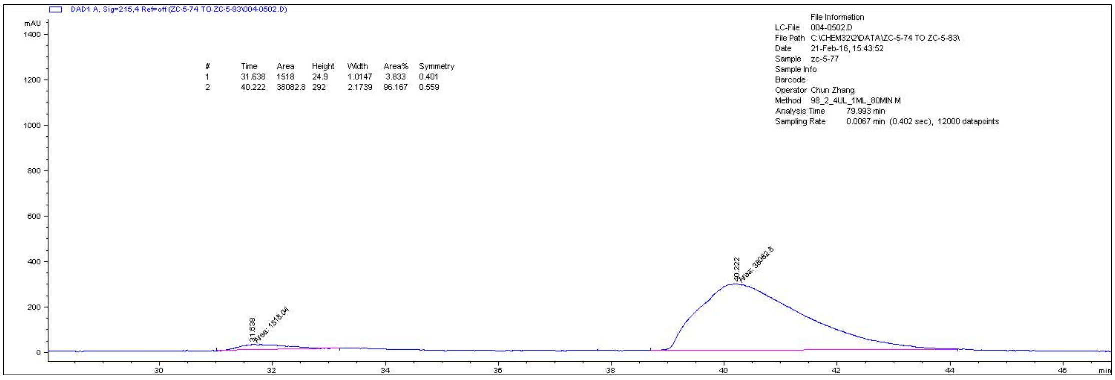

product

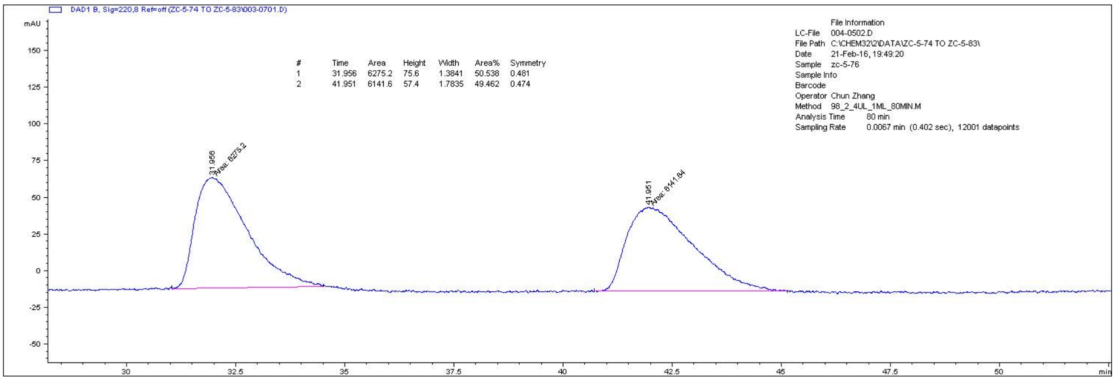

Rac

3p

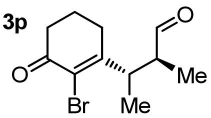

Separation of enantiomers by HPLC, Chiralcel® Column Whelko, 25 °C, Hexane : *i*-PrOH = 90:10, 1mL/min, minor retention time: t_1 = 38.8, major retention time: t_2 = 47.0; er = 95:5.

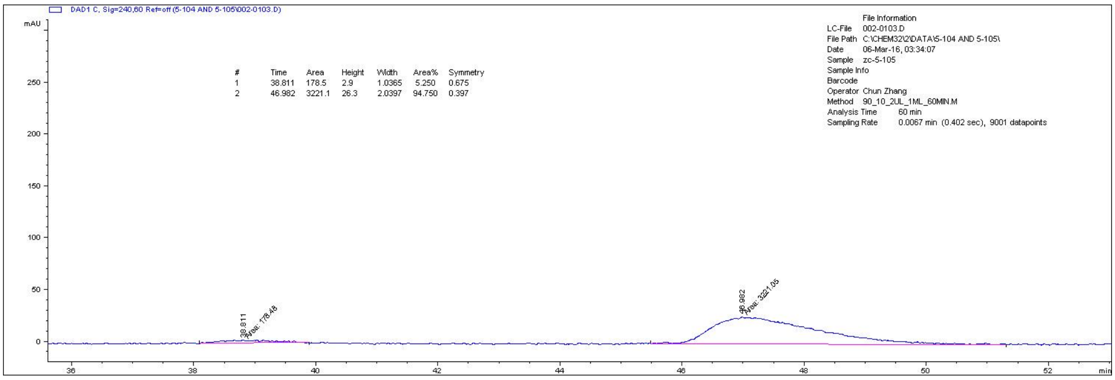

#	Time	Area	Height	Width	Area%	Symmetry
1	38.811	178.5	2.9	1.0365	5.250	0.675
2	46.982	3221.1	26.3	2.0397	94.750	0.397

product

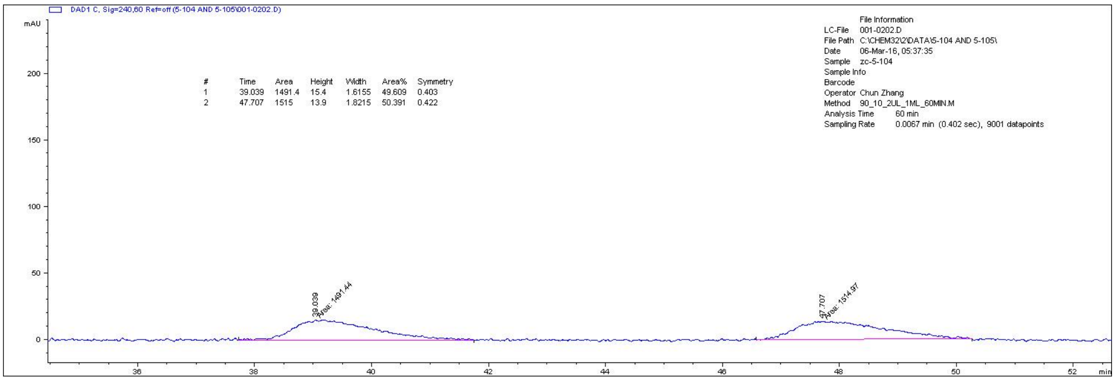

#	Time	Area	Height	Width	Area%	Symmetry
1	39.039	1491.4	15.4	1.6155	49.609	0.403
2	47.707	1515	13.9	1.8215	50.391	0.422

Rac

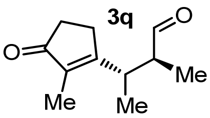

3q

Separation of enantiomers by HPLC, Chiralcel® Column AS-H, 25 °C, Hexane : *i*-PrOH = 95:5, 0.5mL/min, minor retention time: t_1 = 40.5, major retention time: t_2 = 45.7; er = 94:6.

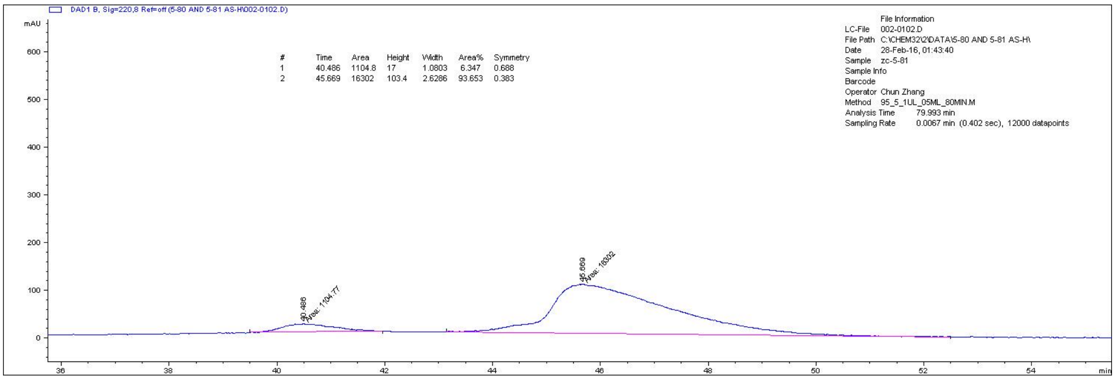

product

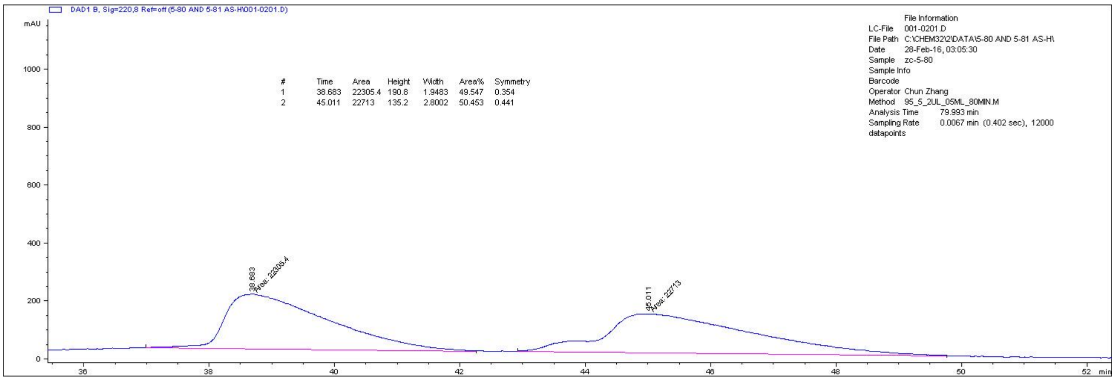

Rac

3r

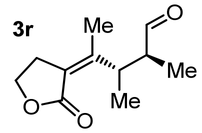

Separation of enantiomers by HPLC, Chiralcel® Column Whelko, 25 °C, Hexane : *i*-PrOH = 90:10, 1mL/min, minor retention time: t_1 = 43.5, major retention time: t_2 = 47.8; er = 92:8.

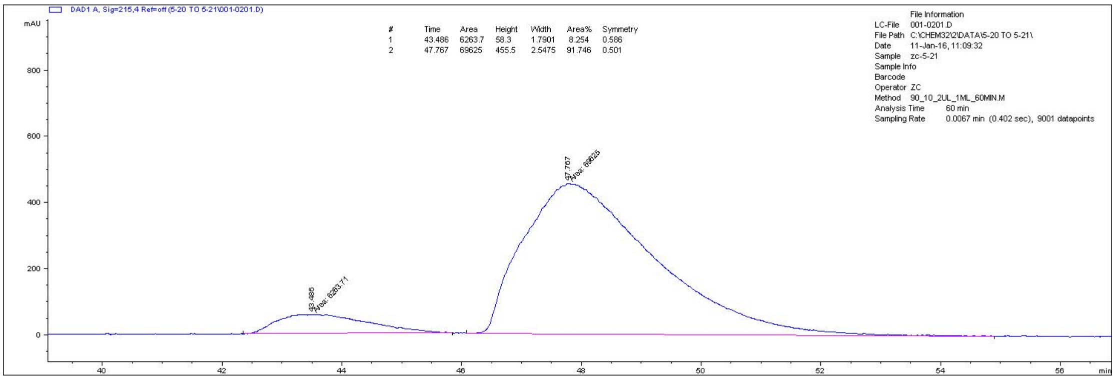

product

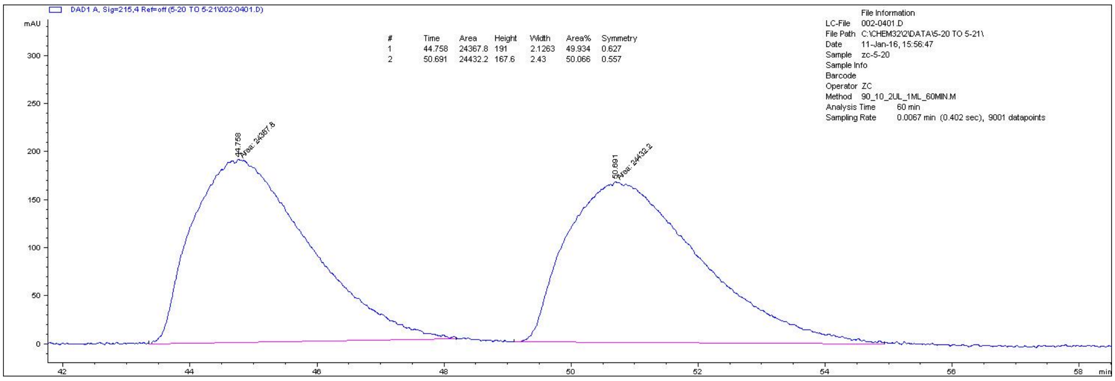

Rac

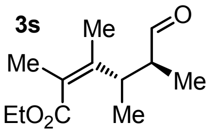

Separation of enantiomers by HPLC, Chiralcel® Column Whelko, 25 °C, Hexane : *i*-PrOH = 98:2, 1mL/min, minor retention time: t_1 = 11.9, major retention time: t_2 = 15.1; er = 91:9.

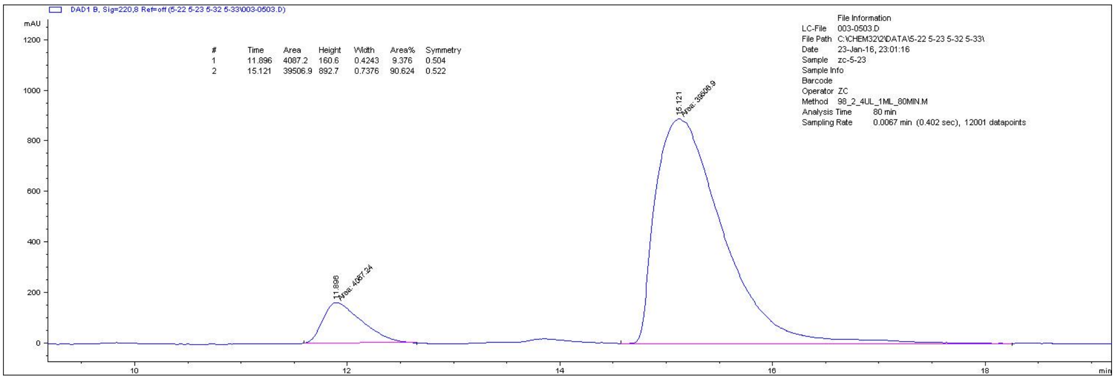

product

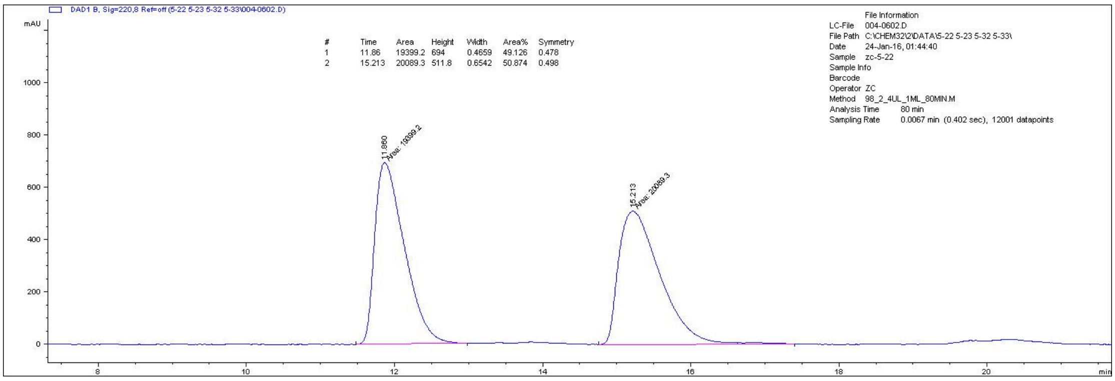

Rac

3t

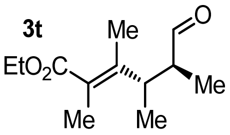

Separation of enantiomers by HPLC, Chiralcel® Column Whelko, 25 °C, Hexane : *i*-PrOH = 99.8:0.2, 1mL/min, major retention time: t_1 = 61.6, minor retention time: t_2 = 68.7; er = 97:3.

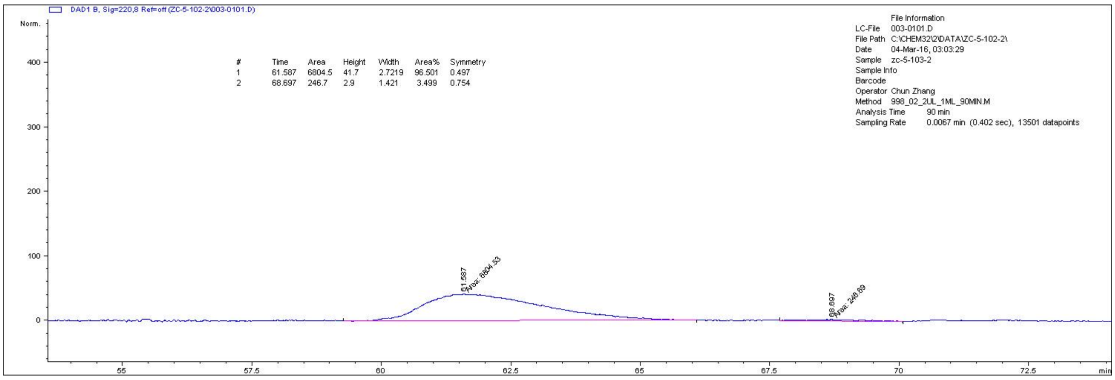

product

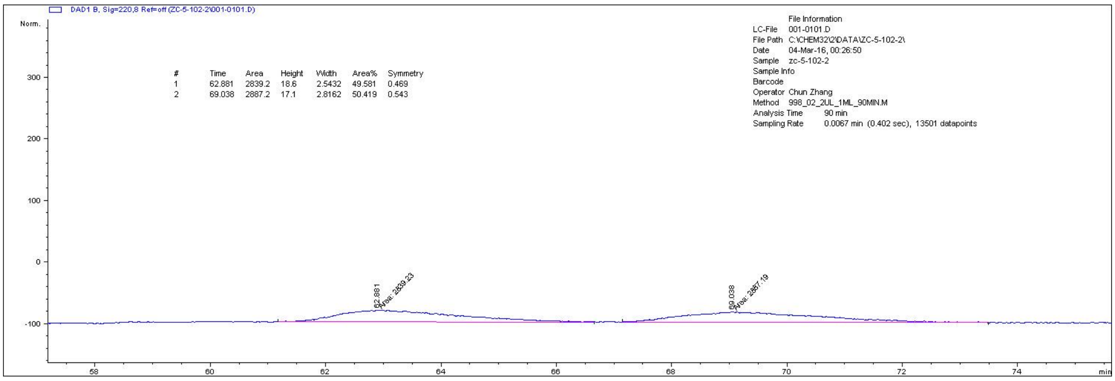

Rac

3t'

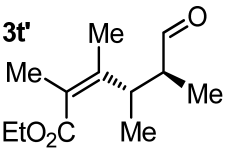

Separation of enantiomers by HPLC, Chiralcel® Column Whelko, 25 °C, Hexane : *i*-PrOH = 98:2, 1mL/min, minor retention time: $t_1 = 11.2$, major retention time: $t_2 = 14.2$; er = 91:9.

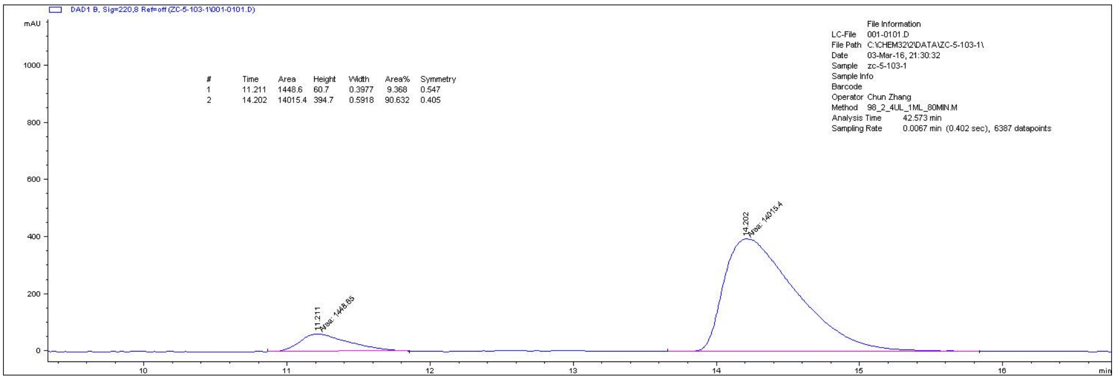

product

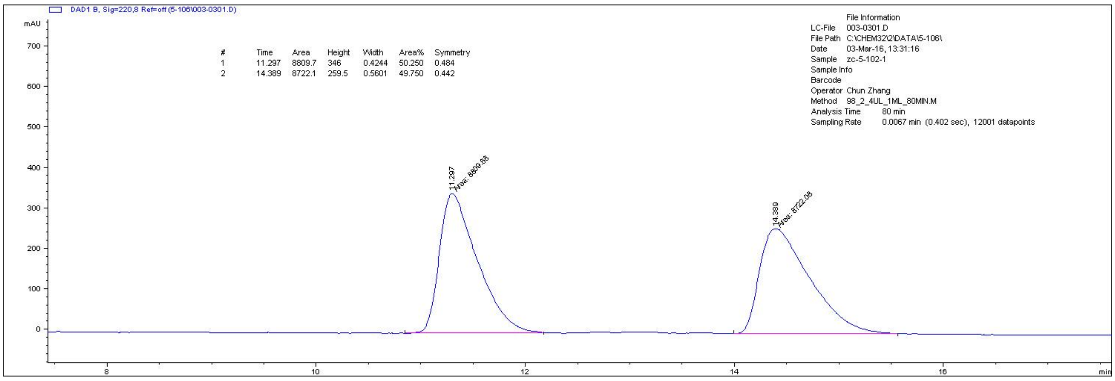

Rac

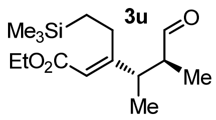

Separation of enantiomers by HPLC, Chiralcel® Column Whelko, 25 °C, Hexane : *i*-PrOH = 99.9:0.1, 1mL/min, major retention time: t_1 = 38.4, minor retention time: t_2 = 42.9; er = 90:10.

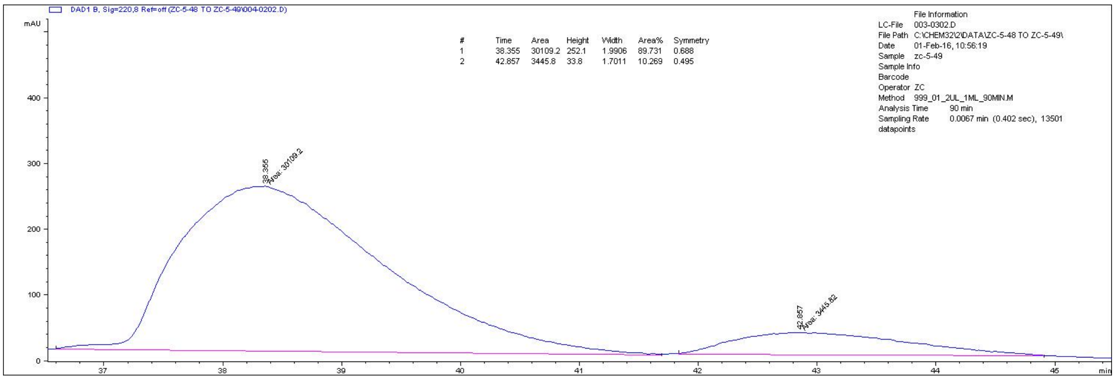

product

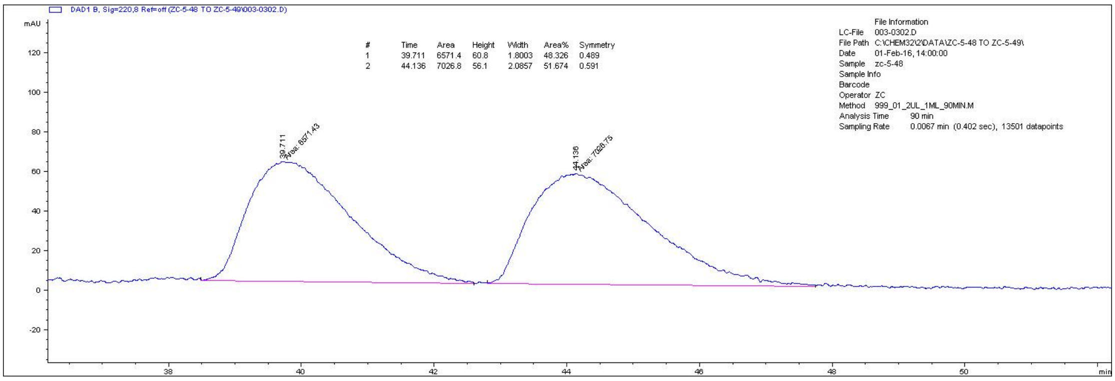

Rac

3v

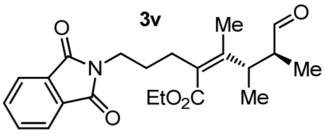

Separation of enantiomers by HPLC, Chiralcel® Column Whelko, 25 °C, Hexane : *i*-PrOH = 80:20, 1mL/min, minor retention time: t_1 = 36.3, major retention time: t_2 = 41.0; er = 91:9.

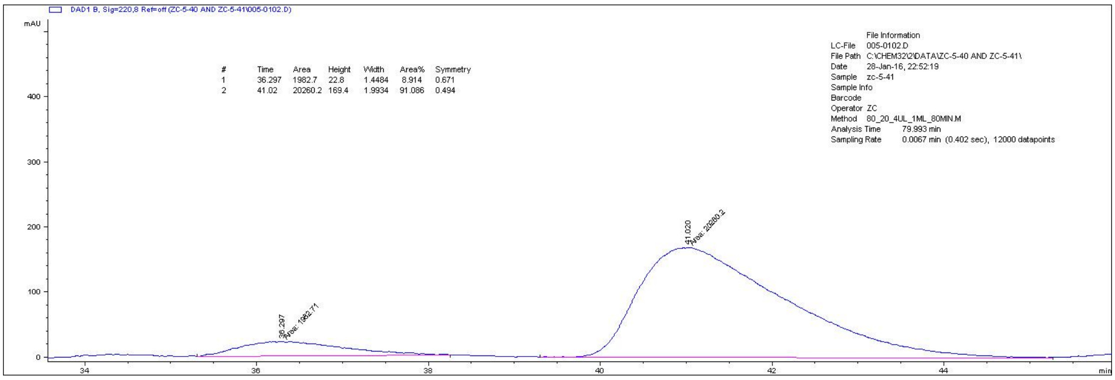

product

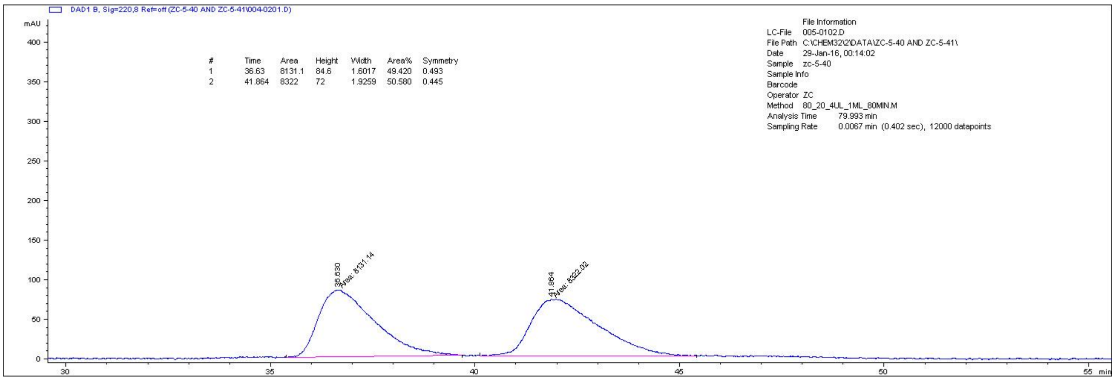

Rac

3z

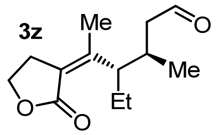

Separation of enantiomers by HPLC, Chiralcel® Column Whelko, 25 °C, Hexane : *i*-PrOH = 95:5, 1mL/min, minor retention time: t_1 = 72.7, major retention time: t_2 = 80.3; er = 89:11.

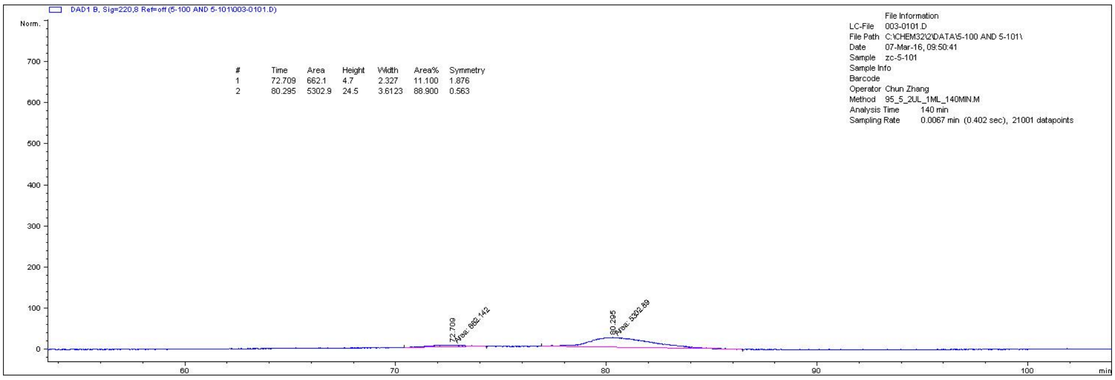

product

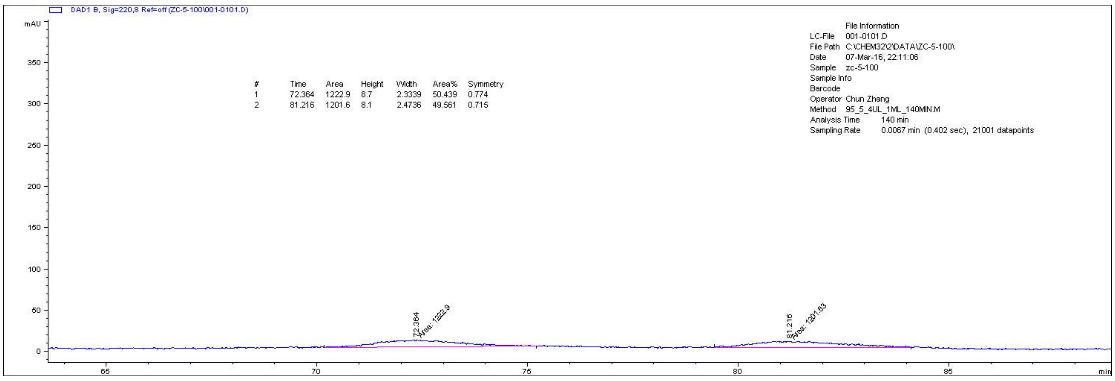

Rac

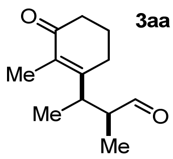

3aa

Separation of enantiomers by HPLC, Chiralcel® Column AY-H, 25 °C, Hexane : *i*-PrOH = 98:2, 1mL/min, major retention time: t_1 = 39.0, minor retention time: t_2 = 44.4; er = 57:43.

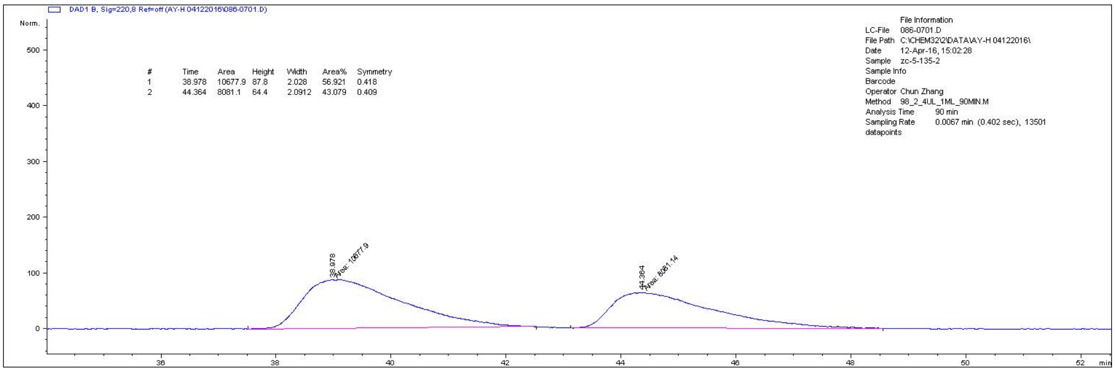

product

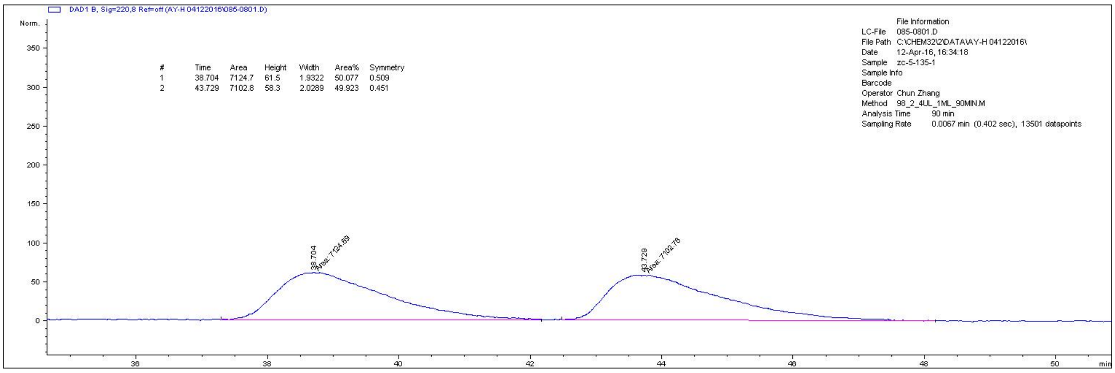

Rac

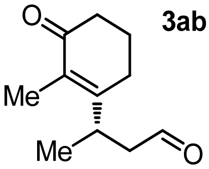

3ab

Separation of enantiomers by HPLC, Chiralcel® Column AY-H, 25 °C, Hexane : *i*-PrOH = 95:5, 1mL/min, minor retention time: t_1 = 13.7, major retention time: t_2 = 19.5; er = 89:11.

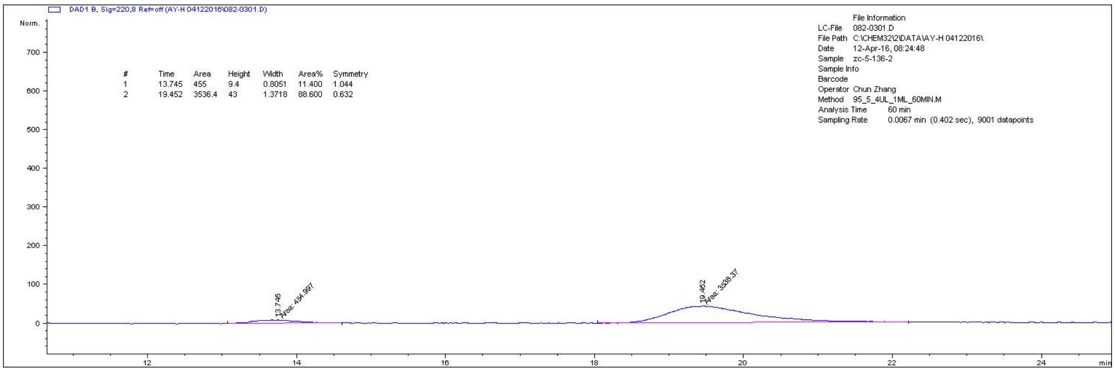

product

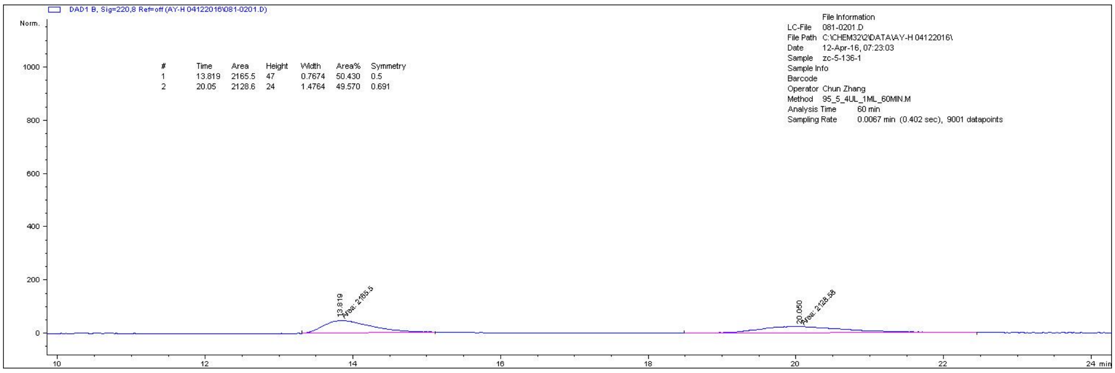

Rac

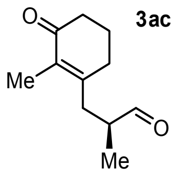

3ac

Separation of enantiomers by SFC, Chiralcel® Column AY-H, 40 ºC, $^{i-}$PrOH:CO_2 = 4:96, 2mL/min, 180bar, minor retention time: t_1 = 10.5, major retention time: t_2 = 11.5; er = 79:21.

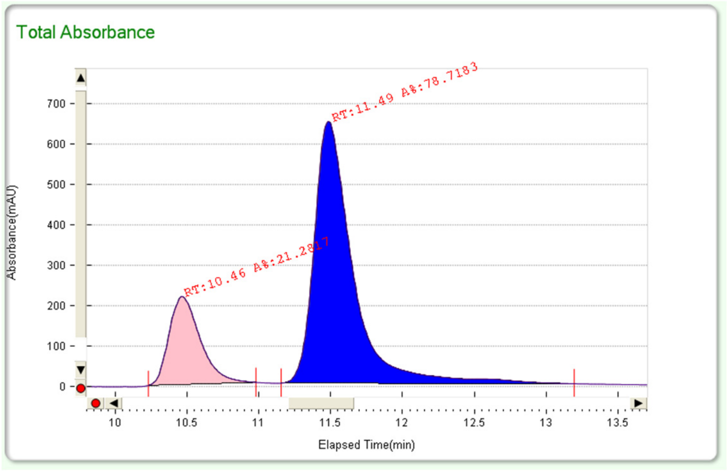

product

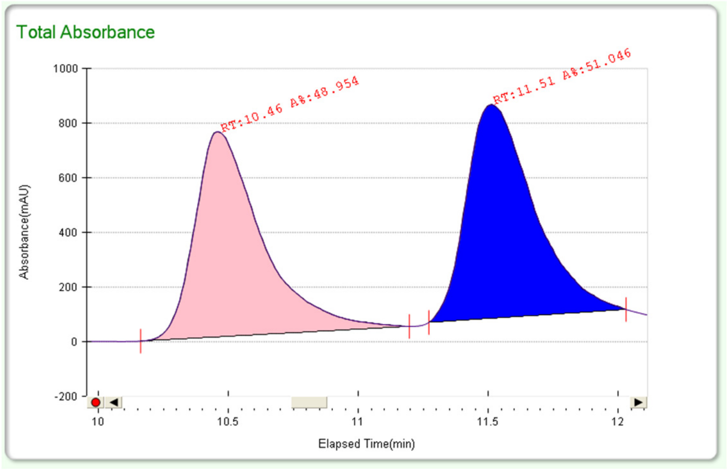

Rac

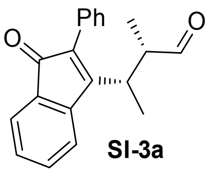

SI-3a

Separation of enantiomers by SFC, Chiralcel® Column AY-H, 40 °C, ^{i}PrOH:CO_2 = 4:96, 2mL/min, 180bar, major retention time: t_1 = 36.9, minor retention time: t_2 = 42.2; er = 88:12.

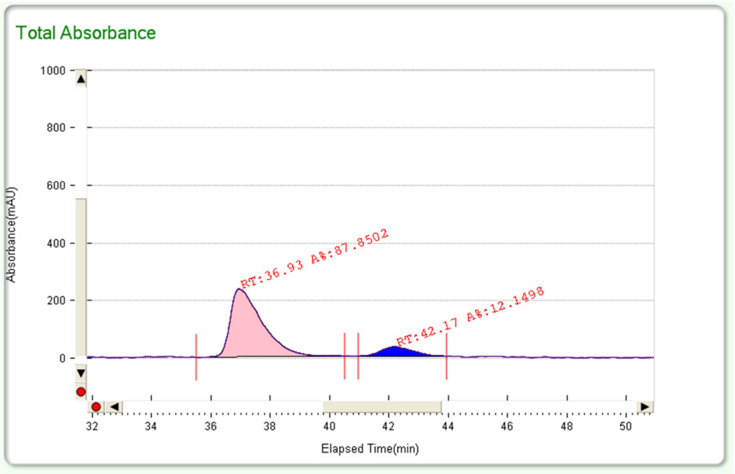

product

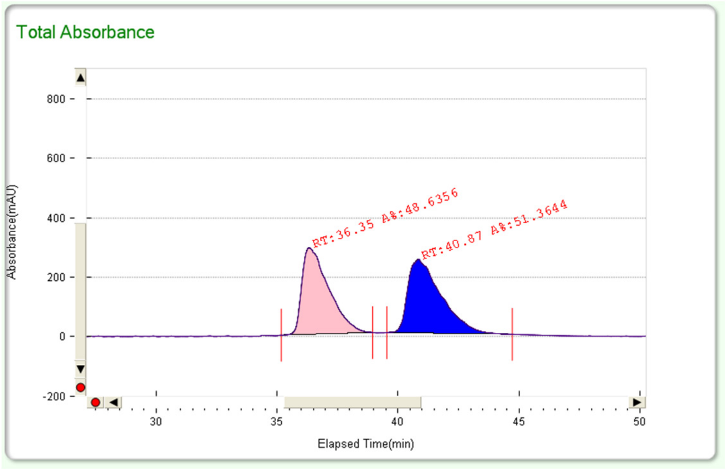

Rac

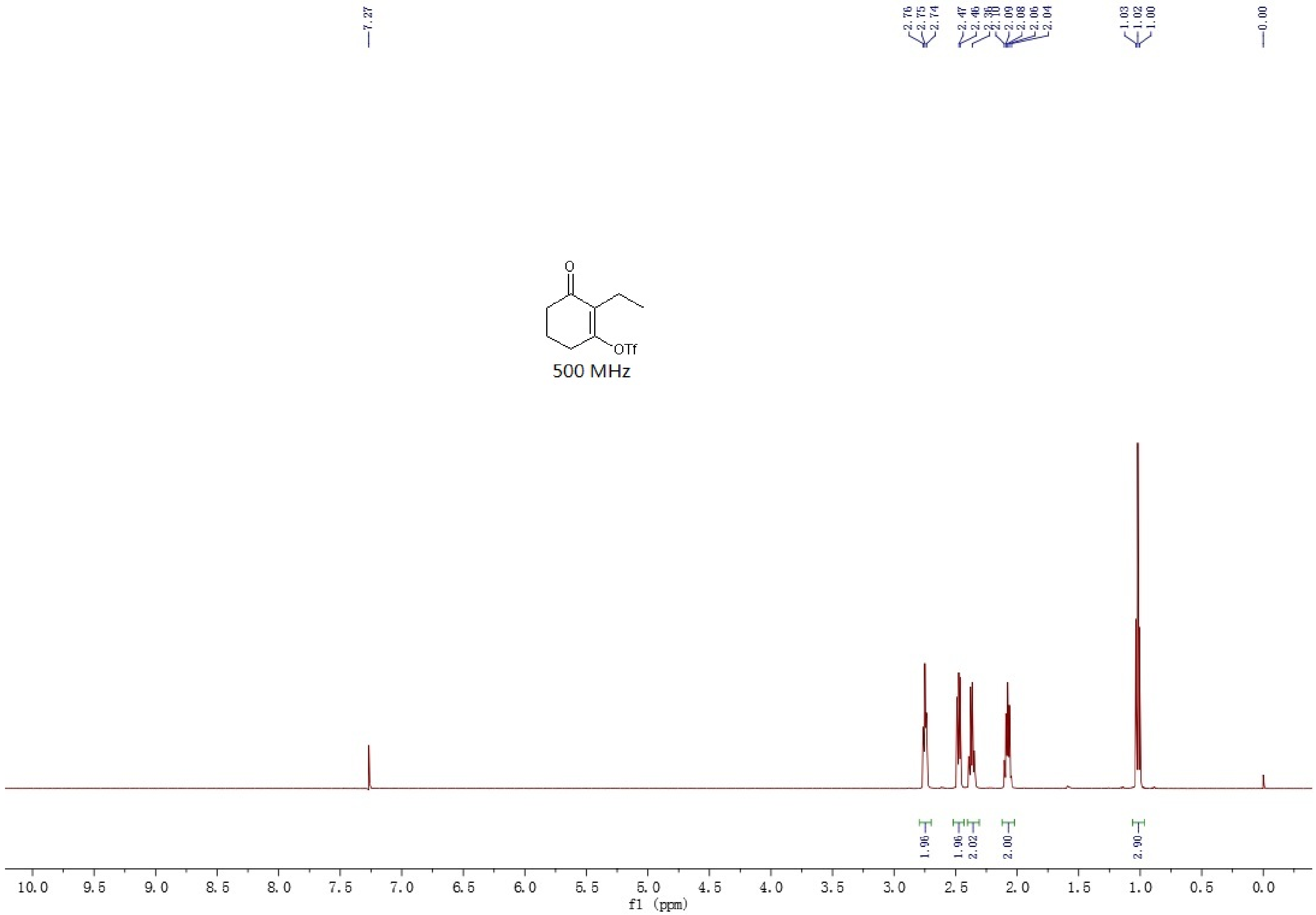
7.27
2.76
2.75
2.74
2.47
2.46
2.10
2.09
2.08
2.06
2.04
1.03
1.02
1.00
0.00
O
OTf
500 MHz
1.96
1.96
2.02
2.00
2.90
10.0
9.5
9.0
8.5
8.0
7.5
7.0
6.5
6.0
5.5
5.0
4.5
4.0
3.5
3.0
2.5
2.0
1.5
1.0
0.5
0.0
f1 (ppm)

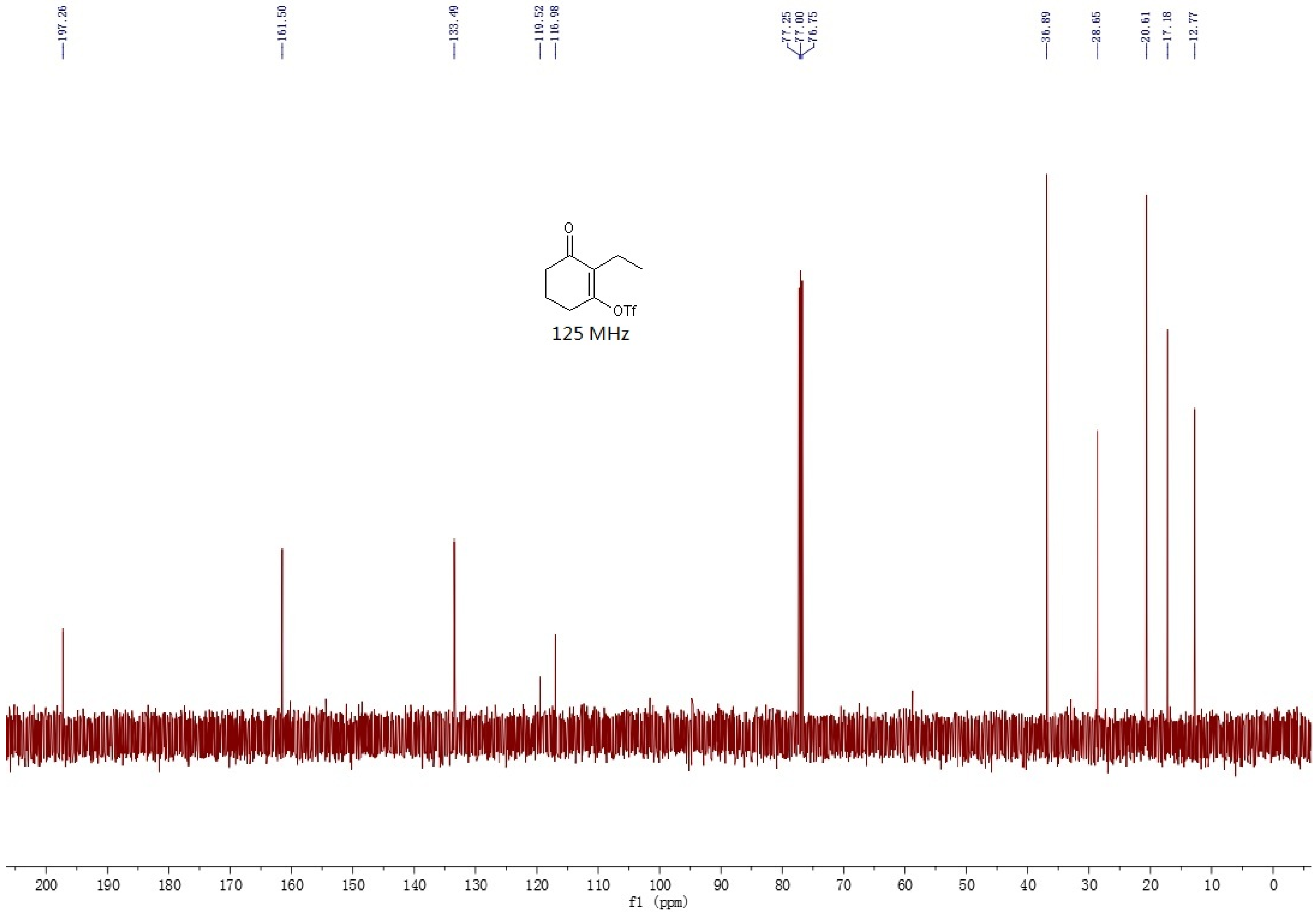

197.26
161.50
133.49
119.52
116.98
77.25
77.00
76.75
36.89
28.65
20.61
17.18
12.77
O
OTf
125 MHz
200 190 180 170 160 150 140 130 120 110 100 90 80 70 60 50 40 30 20 10 0
f1 (ppm)

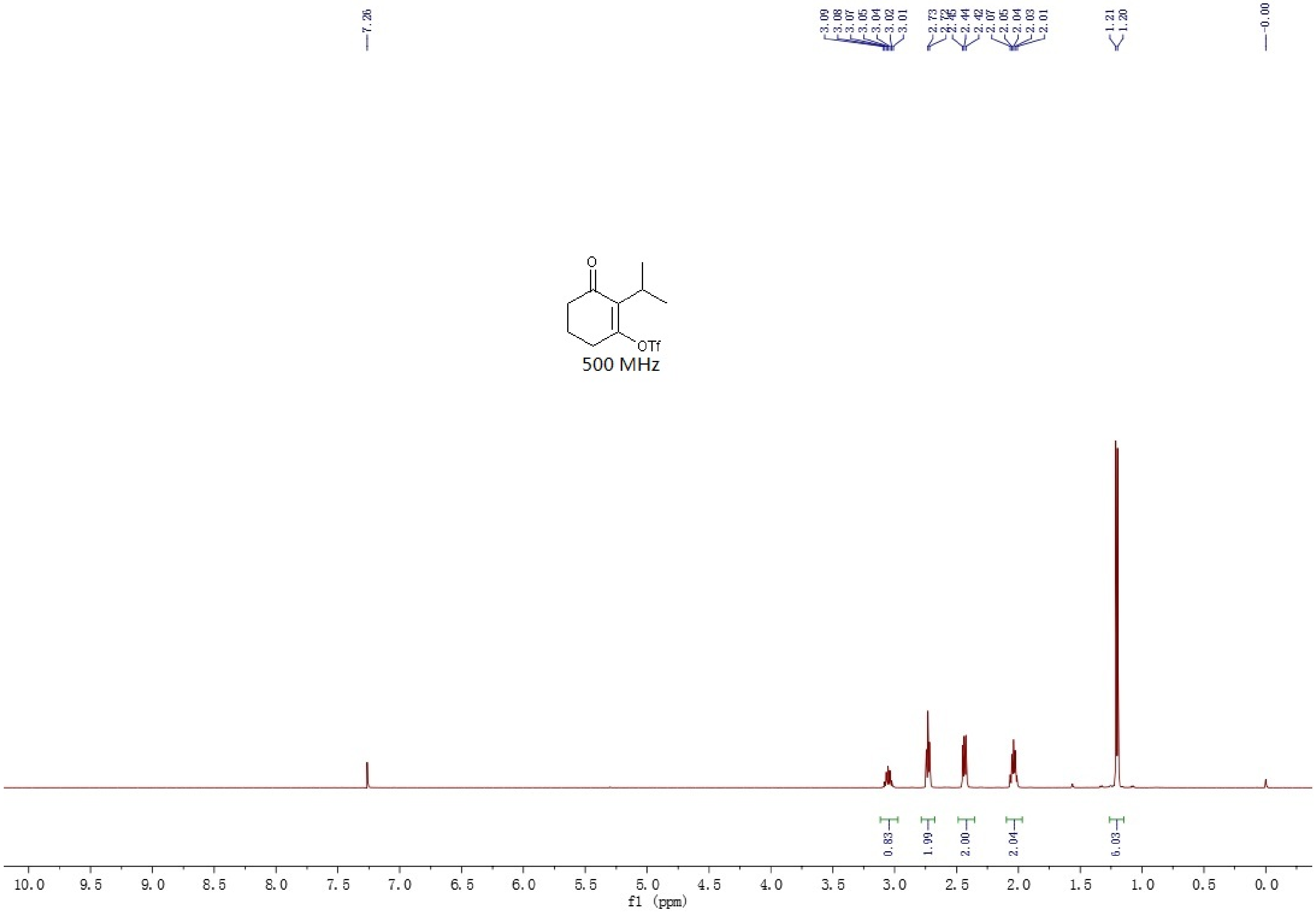

7.26
3.09
3.08
3.07
3.05
3.04
3.02
3.01
2.73
2.72
2.45
2.44
2.42
2.07
2.05
2.04
2.03
2.01
1.21
1.20
0.00
O
OTf
500 MHz
0.83
1.99
2.00
2.04
6.03
10.0
9.5
9.0
8.5
8.0
7.5
7.0
6.5
6.0
5.5
5.0
4.5
4.0
3.5
3.0
2.5
2.0
1.5
1.0
0.5
0.0
f1 (ppm)

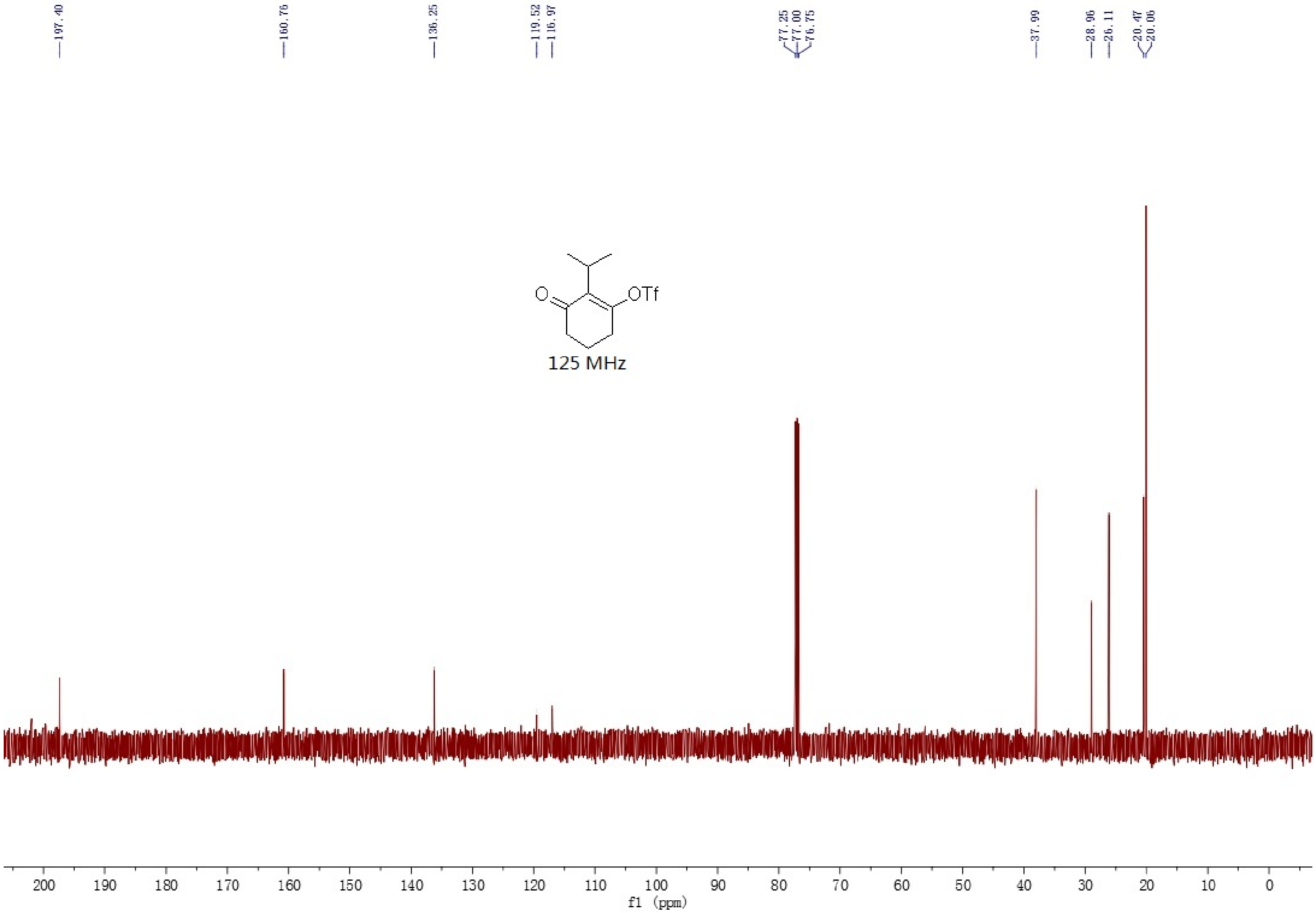

197.40
160.76
136.25
119.52
116.97
77.25
77.00
76.75
37.99
28.96
26.11
20.47
20.06
O
OTf
125 MHz
200
190
180
170
160
150
140
130
120
110
100
90
80
70
60
50
40
30
20
10
0
f1 (ppm)

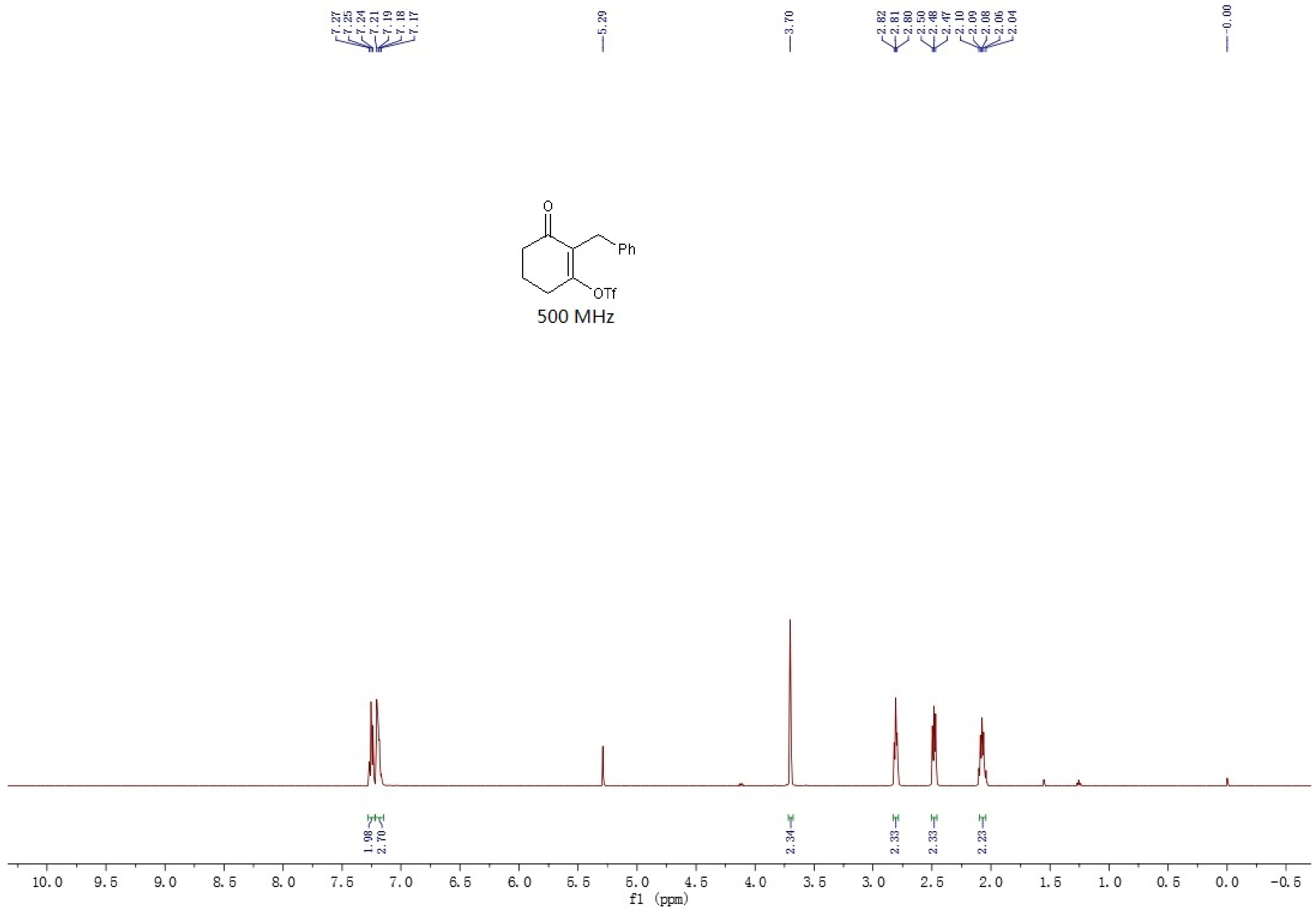

7.27
7.25
7.24
7.21
7.19
7.18
7.17
5.29
3.70
2.82
2.81
2.80
2.50
2.48
2.47
2.10
2.09
2.08
2.06
2.04
0.00
O
Ph
OTf
500 MHz
1.98
2.70
2.34
2.33
2.33
2.23
10.0 9.5 9.0 8.5 8.0 7.5 7.0 6.5 6.0 5.5 5.0 4.5 4.0 3.5 3.0 2.5 2.0 1.5 1.0 0.5 0.0 -0.5
f1 (ppm)

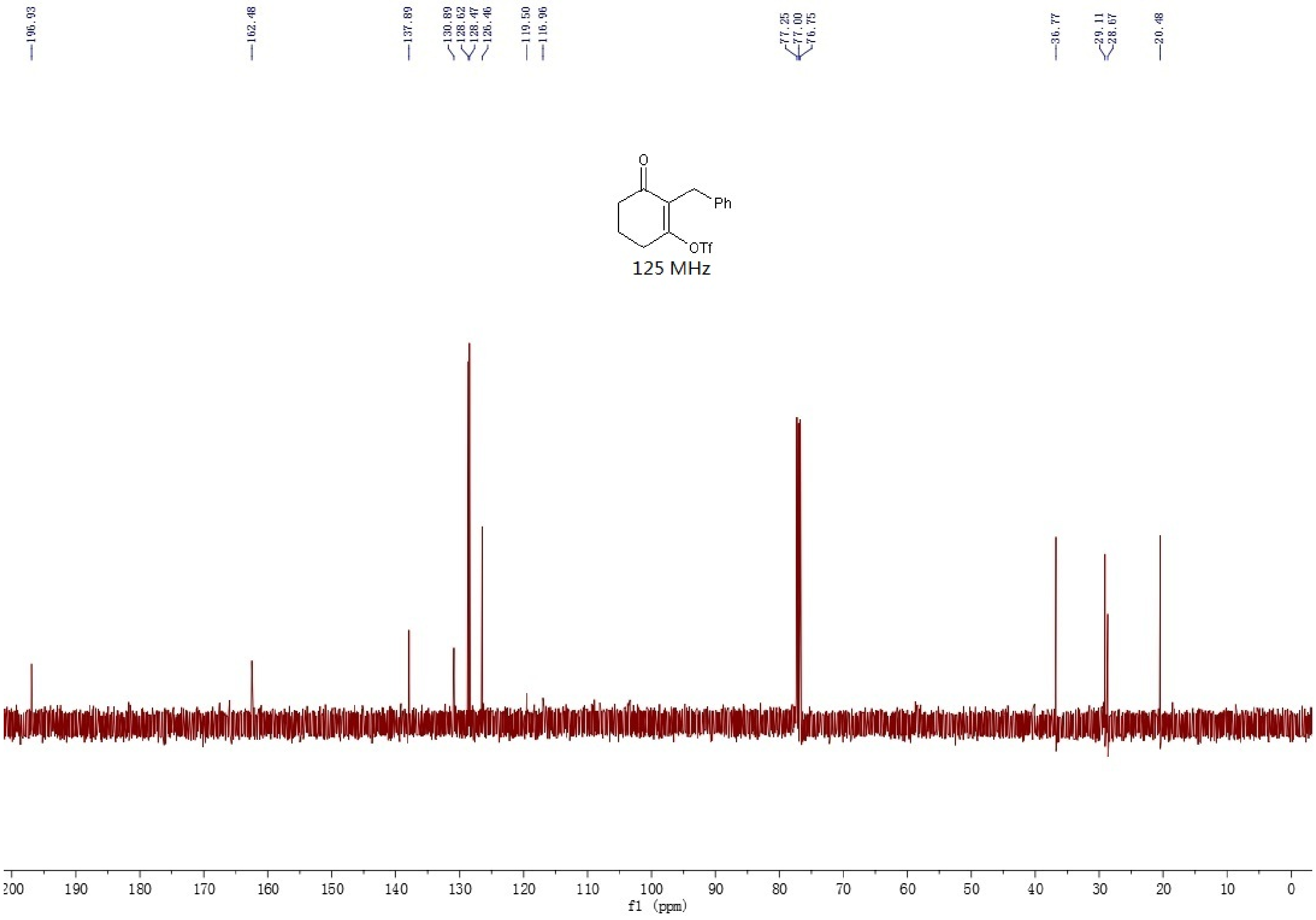
196.93
162.48
137.89
130.89
128.62
128.47
126.46
119.50
116.96
77.25
77.00
76.75
36.77
29.11
28.67
20.48
O
Ph
OTf
125 MHz
200
190
180
170
160
150
140
130
120
110
100
90
80
70
60
50
40
30
20
10
0
f1 (ppm)

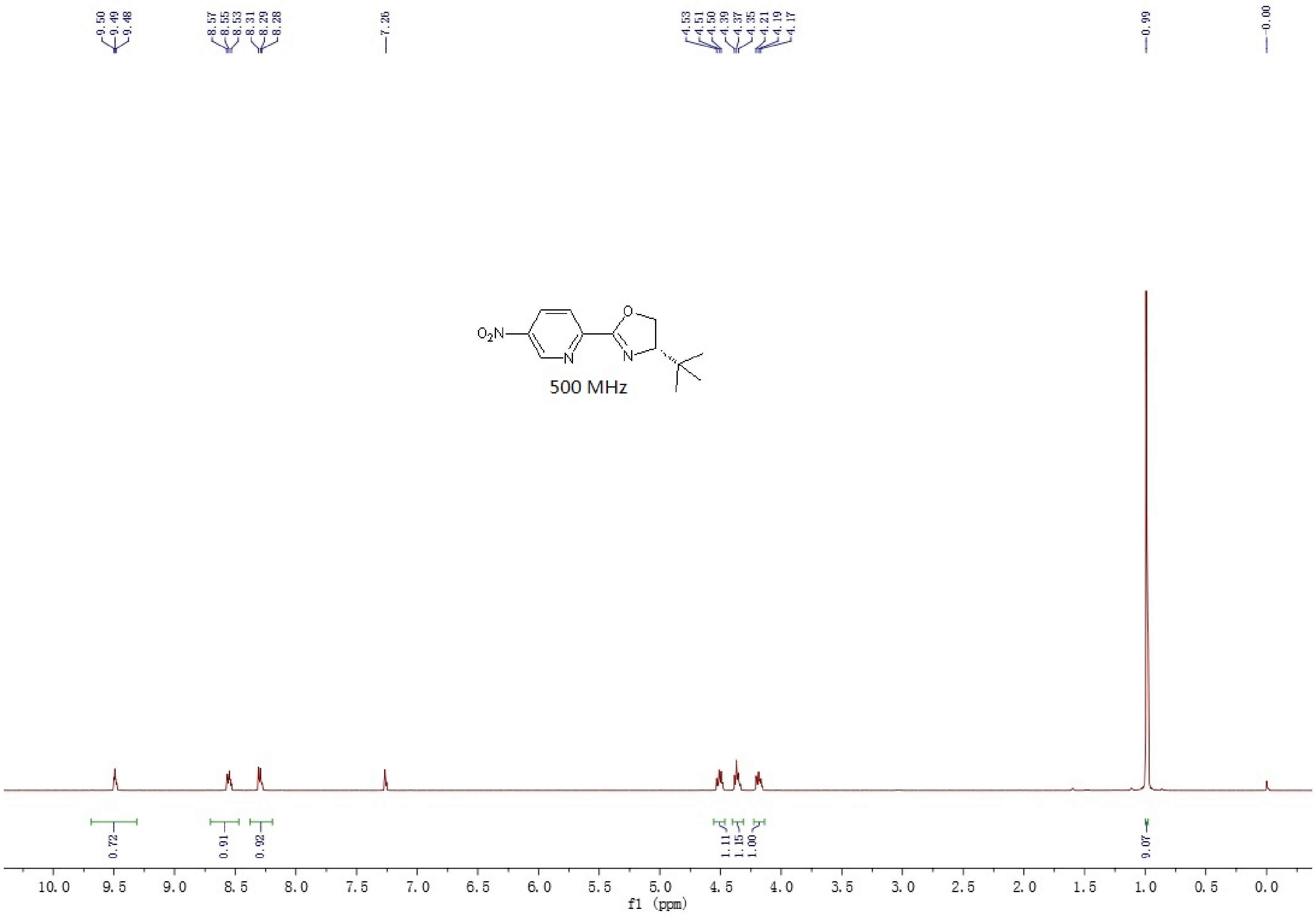
9.50
9.49
9.48
8.57
8.55
8.53
8.31
8.29
8.28
7.26
4.53
4.51
4.50
4.39
4.37
4.35
4.21
4.19
4.17
0.99
0.00
O2N
O
N
N
500 MHz
0.72
0.91
0.92
1.11
1.15
1.00
9.07
10.0 9.5 9.0 8.5 8.0 7.5 7.0 6.5 6.0 5.5 5.0 4.5 4.0 3.5 3.0 2.5 2.0 1.5 1.0 0.5 0.0
f1 (ppm)

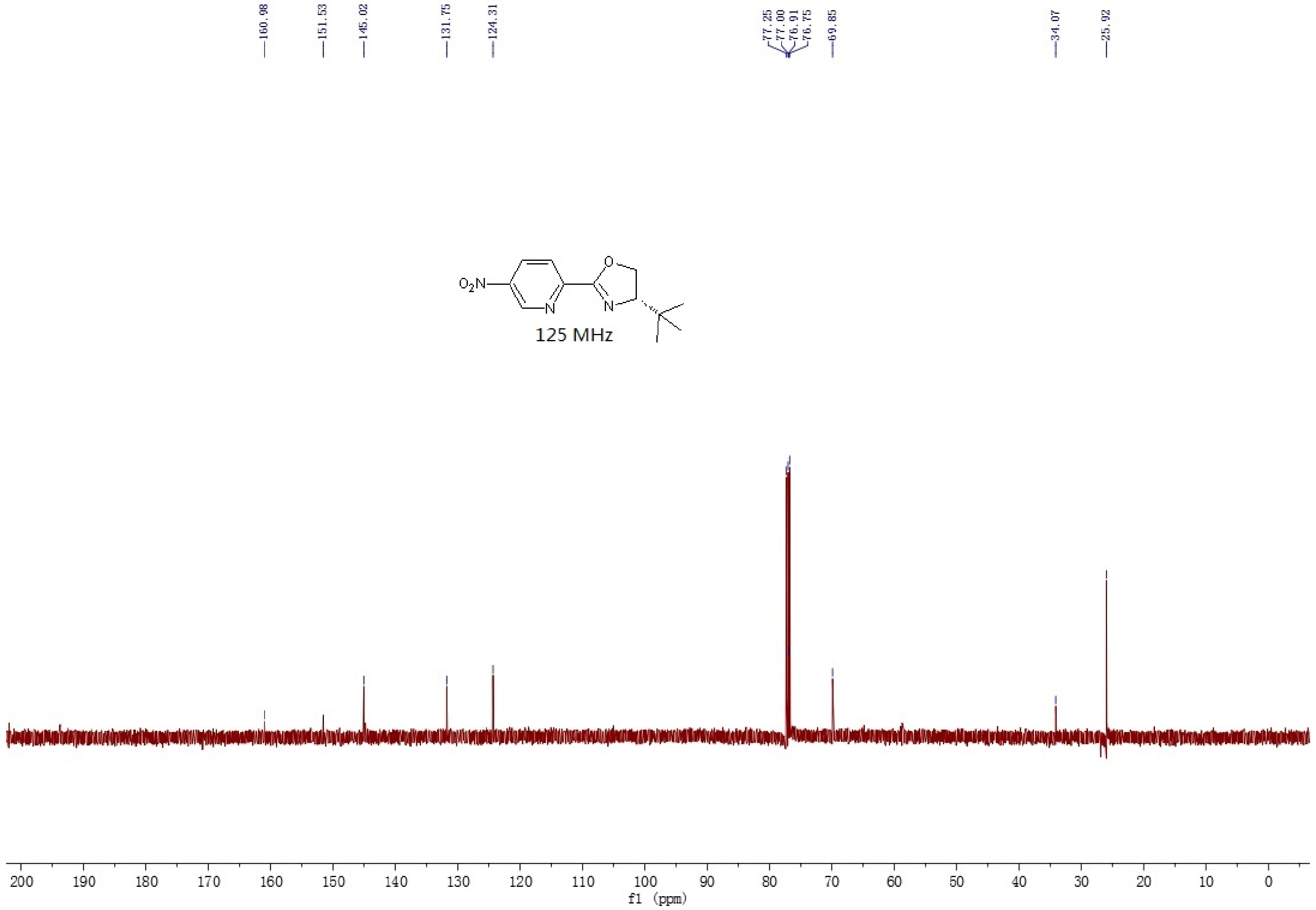
160.98
151.53
145.02
131.75
124.31
77.25
77.00
76.91
76.75
69.85
34.07
25.92
O_2N
N
O
N
125 MHz
200
190
180
170
160
150
140
130
120
110
100
90
80
70
60
50
40
30
20
10
0
f1 (ppm)

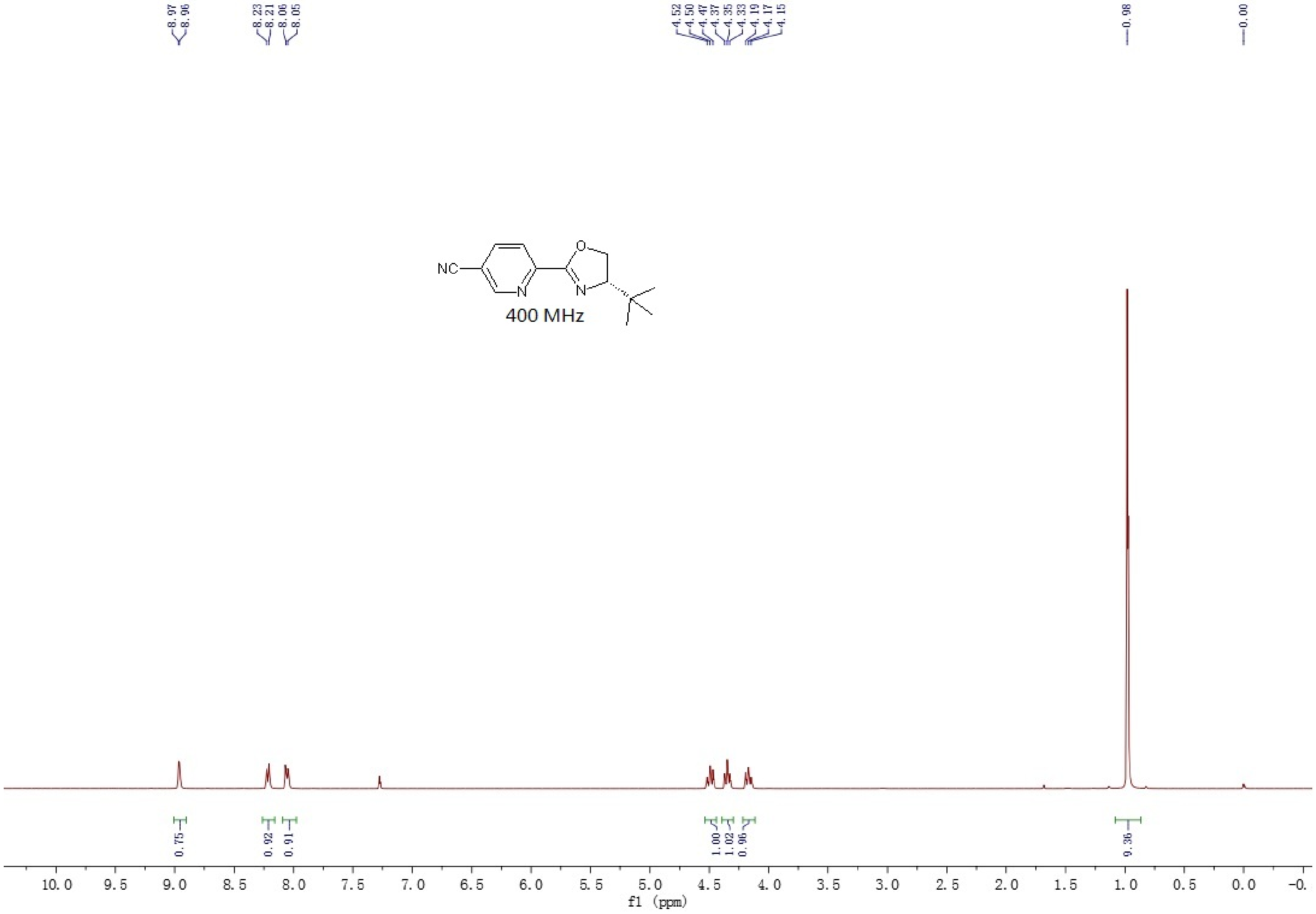
8.97
8.96
8.23
8.21
8.06
8.05
4.52
4.50
4.47
4.37
4.35
4.33
4.19
4.17
4.15
0.98
0.00
NC
O
N
N
400 MHz
0.75
0.92
0.91
1.00
1.02
0.96
9.36
10.0 9.5 9.0 8.5 8.0 7.5 7.0 6.5 6.0 5.5 5.0 4.5 4.0 3.5 3.0 2.5 2.0 1.5 1.0 0.5 0.0
f1 (ppm)

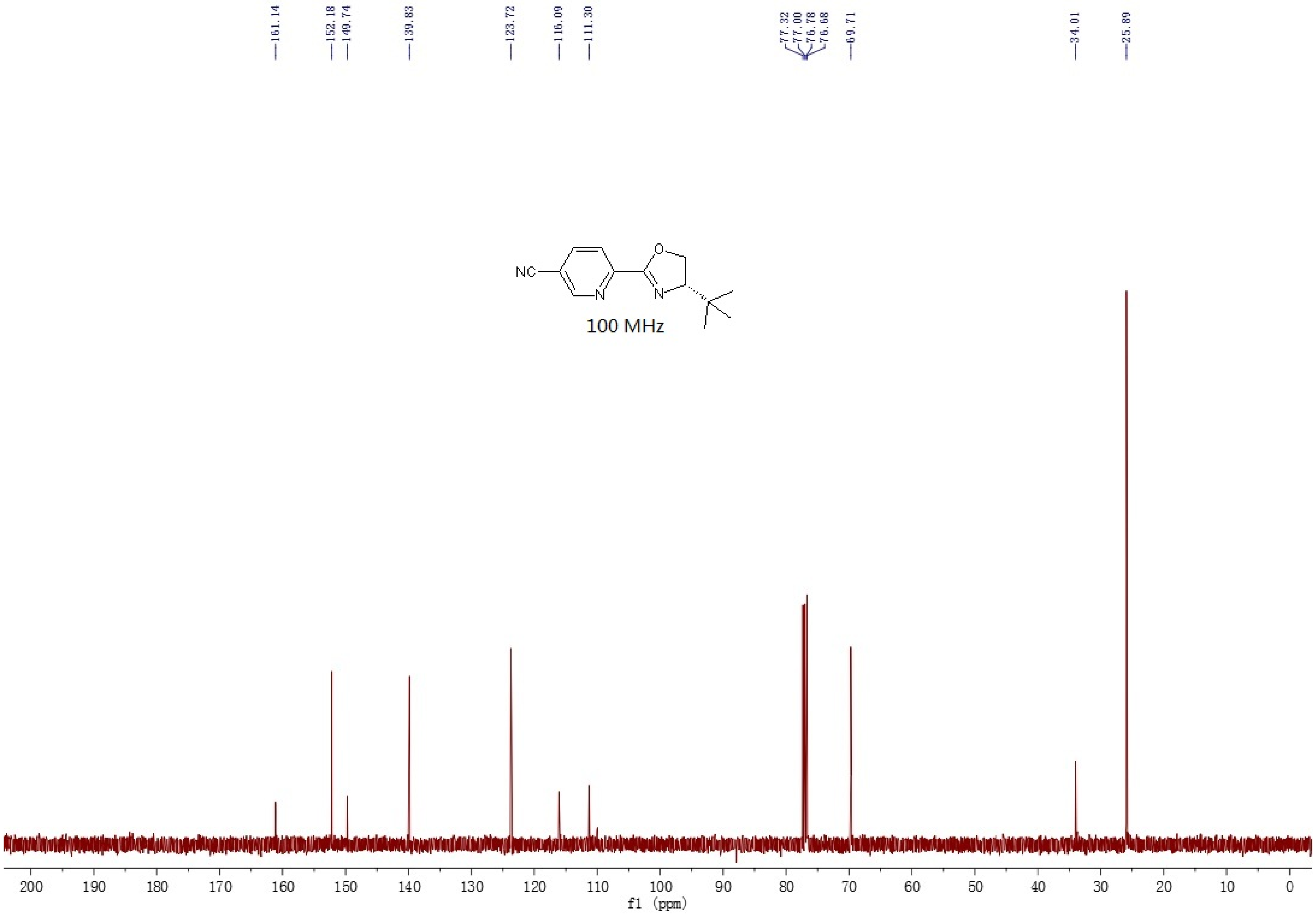
161.14
152.18
149.74
139.83
123.72
116.09
111.30
77.32
77.00
76.78
76.68
69.71
34.01
25.89
NC
O
N
N
100 MHz
f1 (ppm)

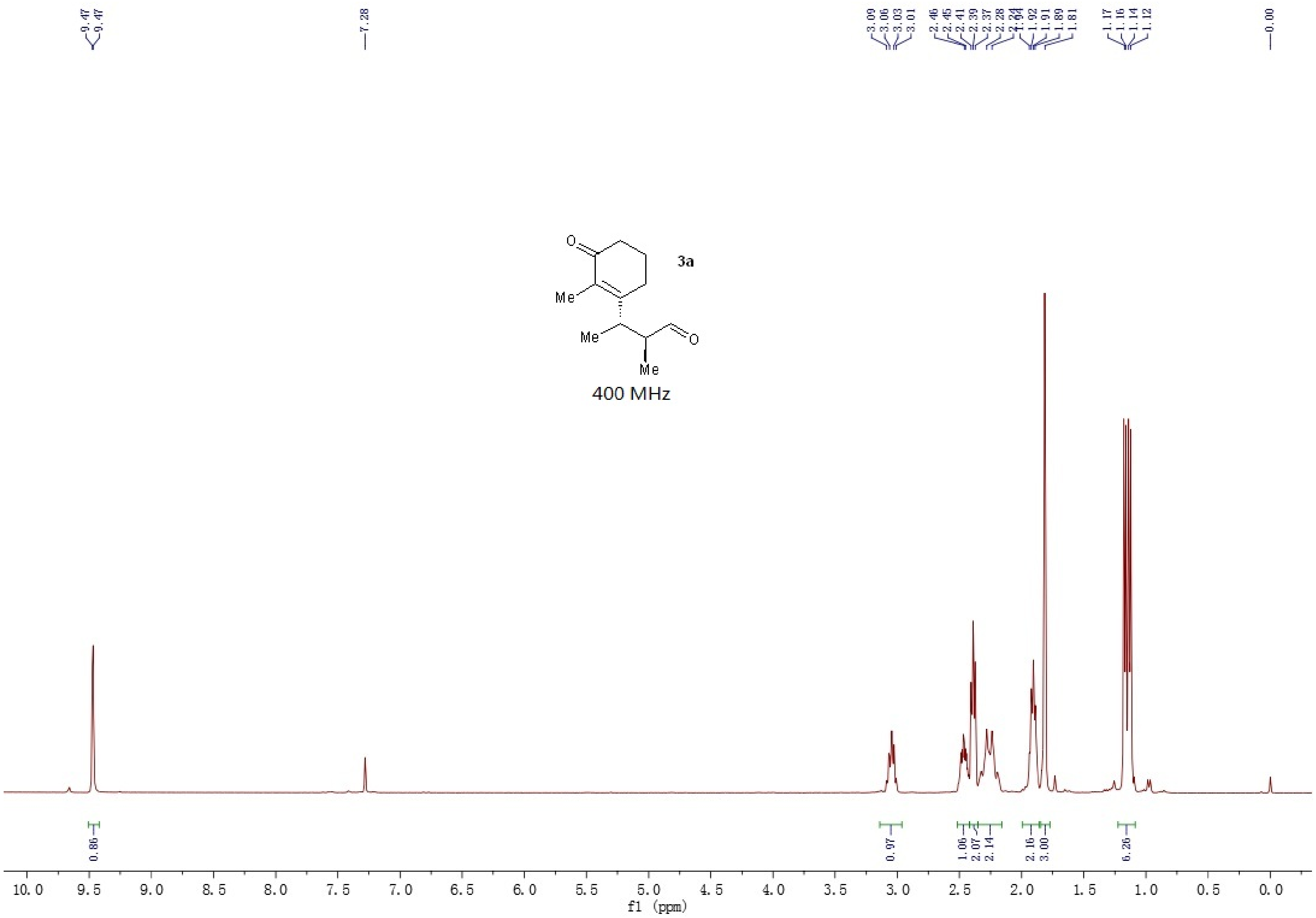
9.47
9.47
7.28
3.09
3.06
3.03
3.01
2.46
2.45
2.41
2.39
2.37
2.28
1.92
1.91
1.89
1.81
1.17
1.16
1.14
1.12
0.00
O
3a
Me
Me
O
Me
400 MHz
0.86
0.97
1.06
2.07
2.14
2.16
3.00
6.26
10.0
9.5
9.0
8.5
8.0
7.5
7.0
6.5
6.0
5.5
5.0
4.5
4.0
3.5
3.0
2.5
2.0
1.5
1.0
0.5
0.0
f1 (ppm)

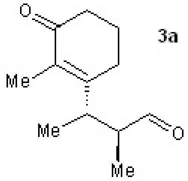
O
Me
Me
Me
O

3a

100 MHz

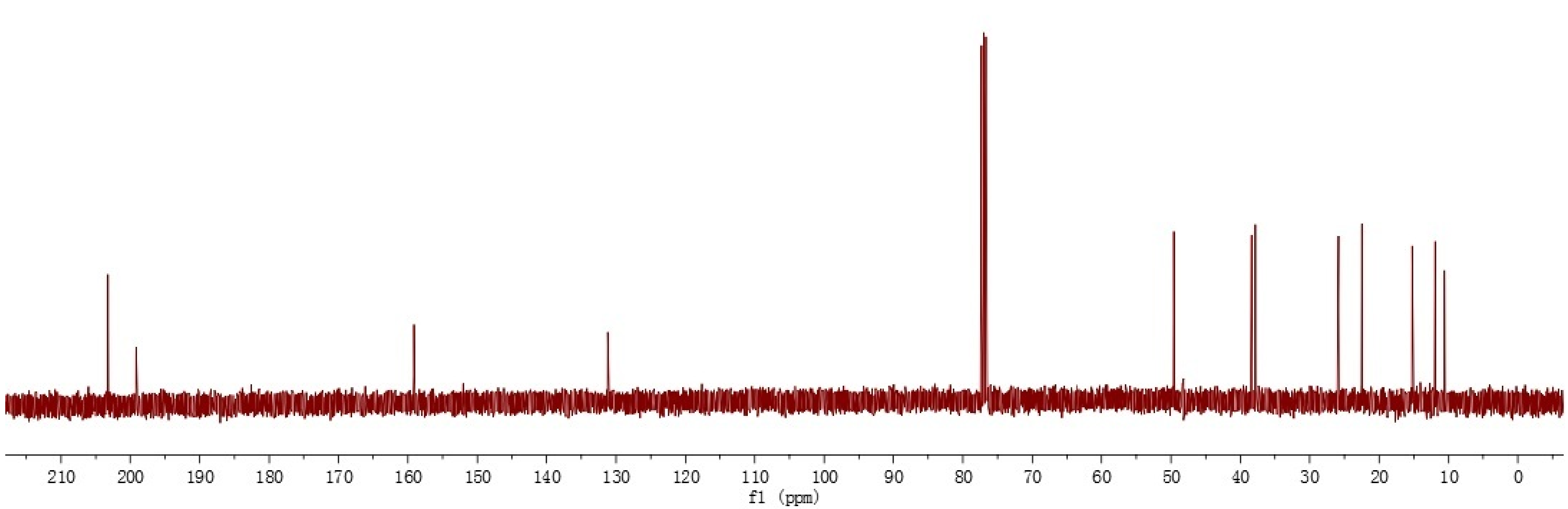
203.20
199.14
159.12
131.19
77.32
77.00
76.68
49.58
38.41
37.87
25.85
22.48
15.25
11.94
10.62
210 200 190 180 170 160 150 140 130 120 110 100 90 80 70 60 50 40 30 20 10 0
f1 (ppm)

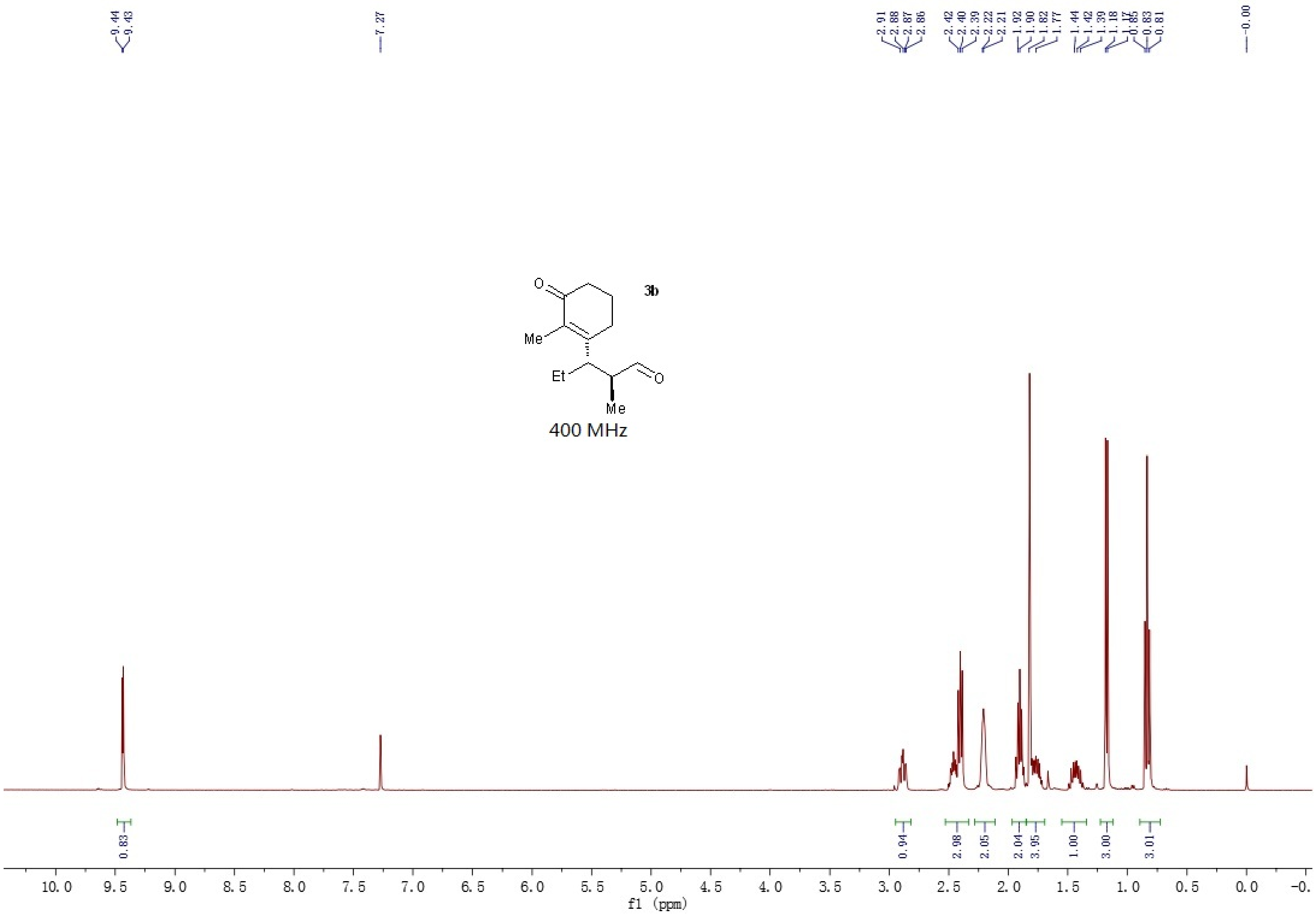

9.44
9.43
7.27
2.91
2.88
2.87
2.86
2.42
2.40
2.39
2.22
2.21
1.92
1.90
1.82
1.77
1.44
1.42
1.39
1.18
1.17
0.85
0.83
0.81
0.00
3b
O
Me
Et
O
Me
400 MHz
0.83
0.94
2.98
2.05
2.04
3.95
1.00
3.00
3.01
10.0
9.5
9.0
8.5
8.0
7.5
7.0
6.5
6.0
5.5
5.0
4.5
4.0
3.5
3.0
2.5
2.0
1.5
1.0
0.5
0.0
-0.
f1 (ppm)

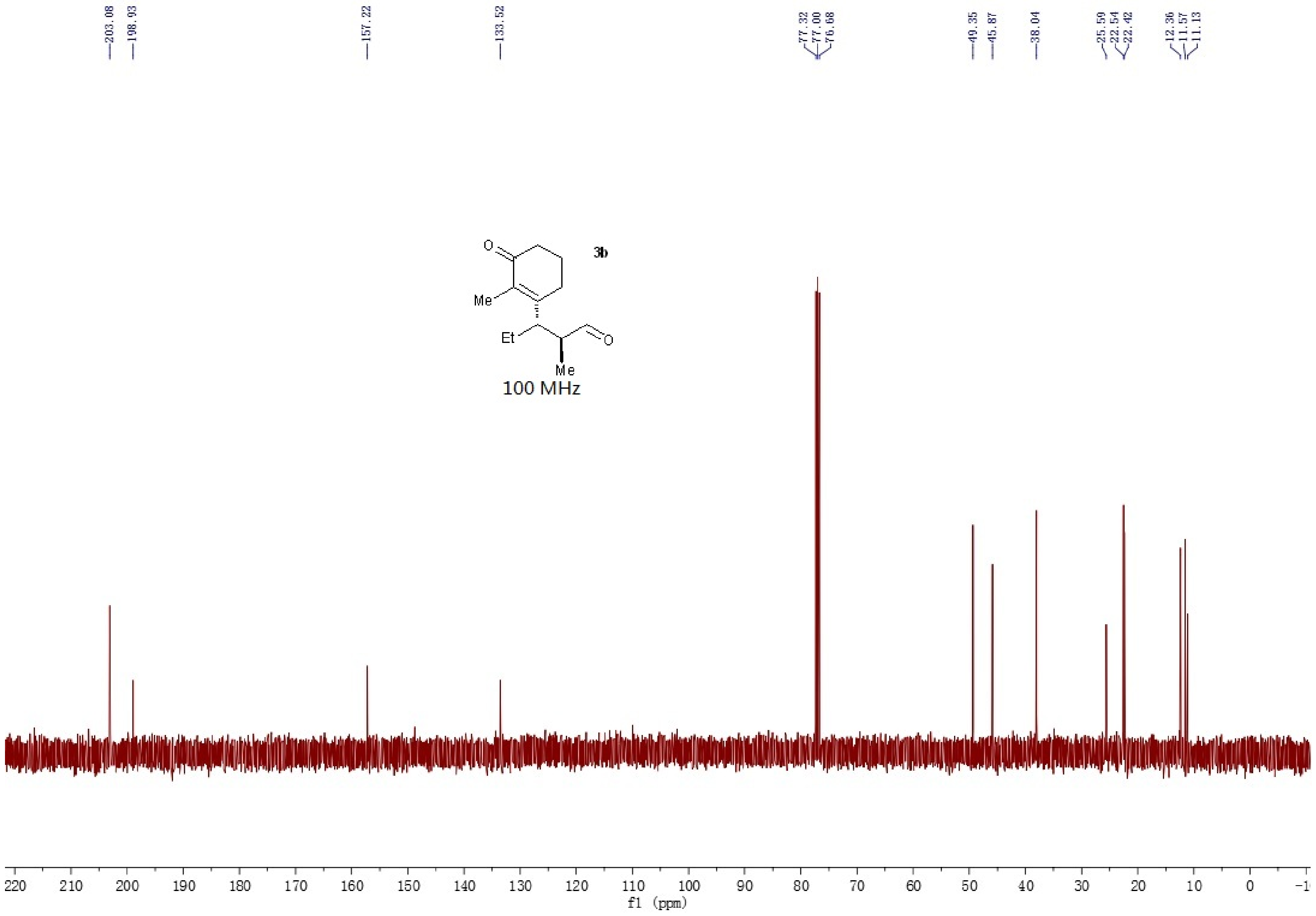

203.08
198.93
157.22
133.52
77.32
77.00
76.68
49.35
45.87
38.04
25.59
22.54
22.42
12.36
11.57
11.13
3b
O
Me
Et
O
Me
100 MHz
f1 (ppm)

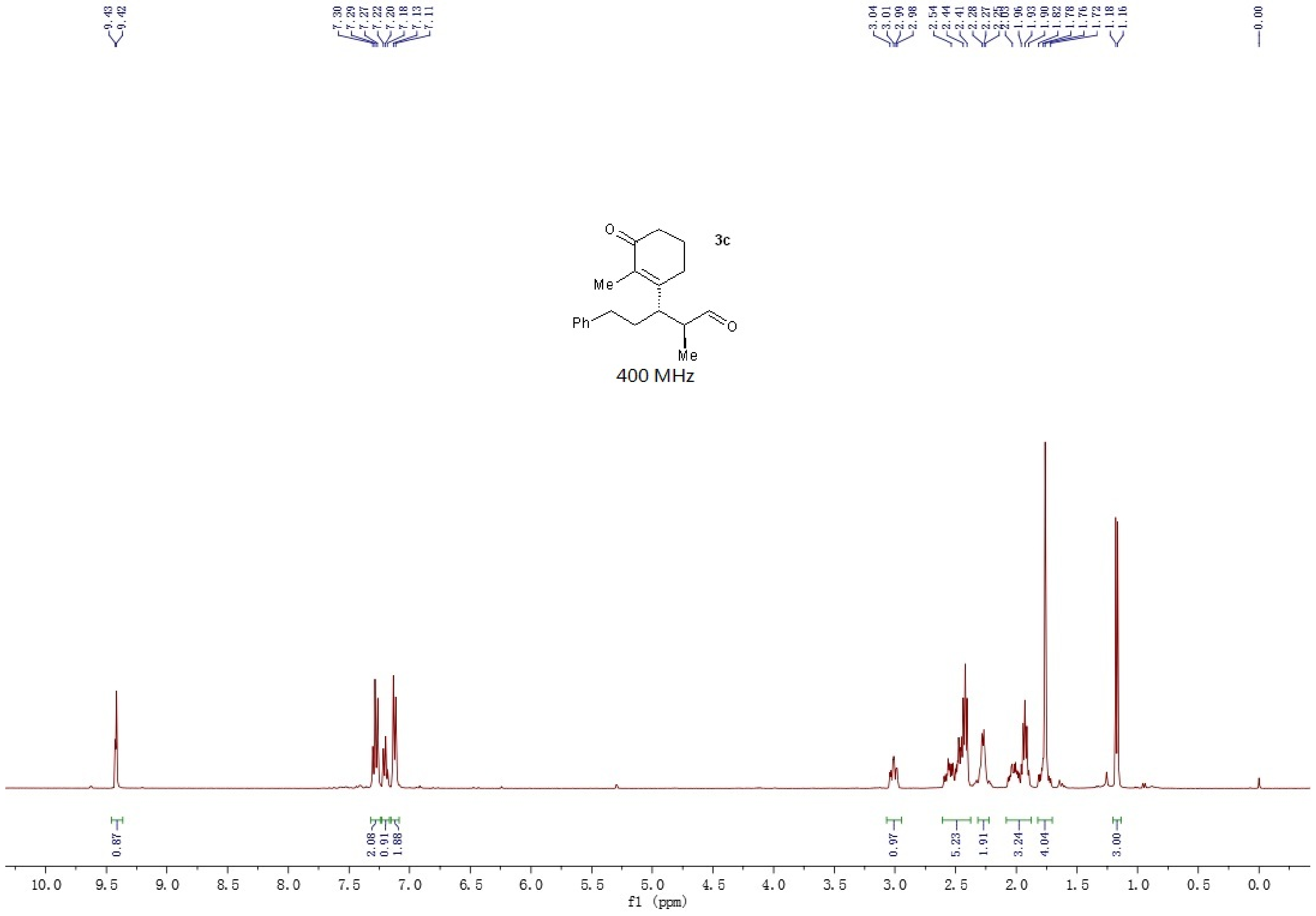

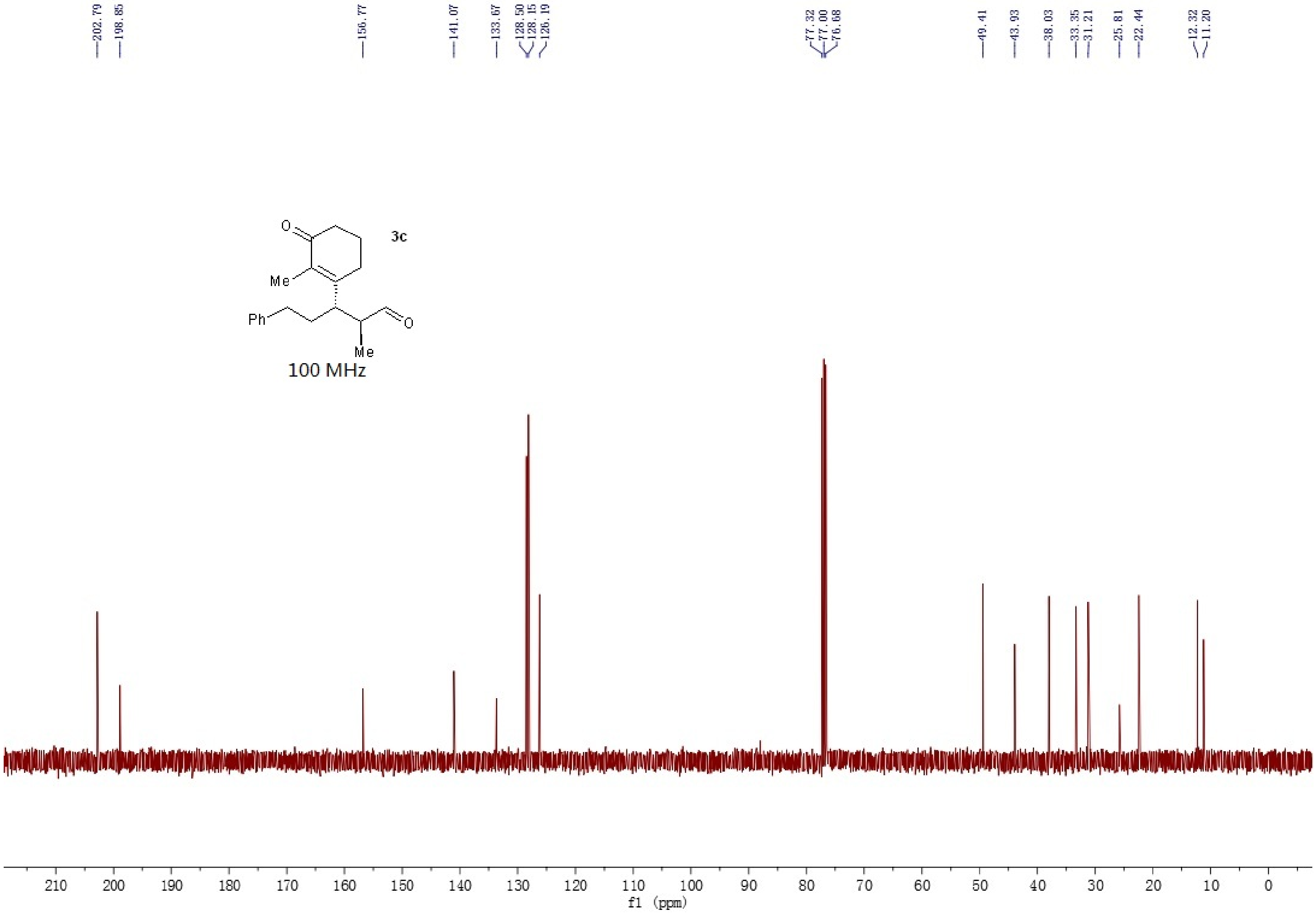
202.79
198.85
156.77
141.07
133.67
128.50
128.15
126.19
77.32
77.00
76.68
49.41
43.93
38.03
33.35
31.21
25.81
22.44
12.32
11.20
O
Me
Ph
Me
O
3c
100 MHz
210 200 190 180 170 160 150 140 130 120 110 100 90 80 70 60 50 40 30 20 10 0
f1 (ppm)

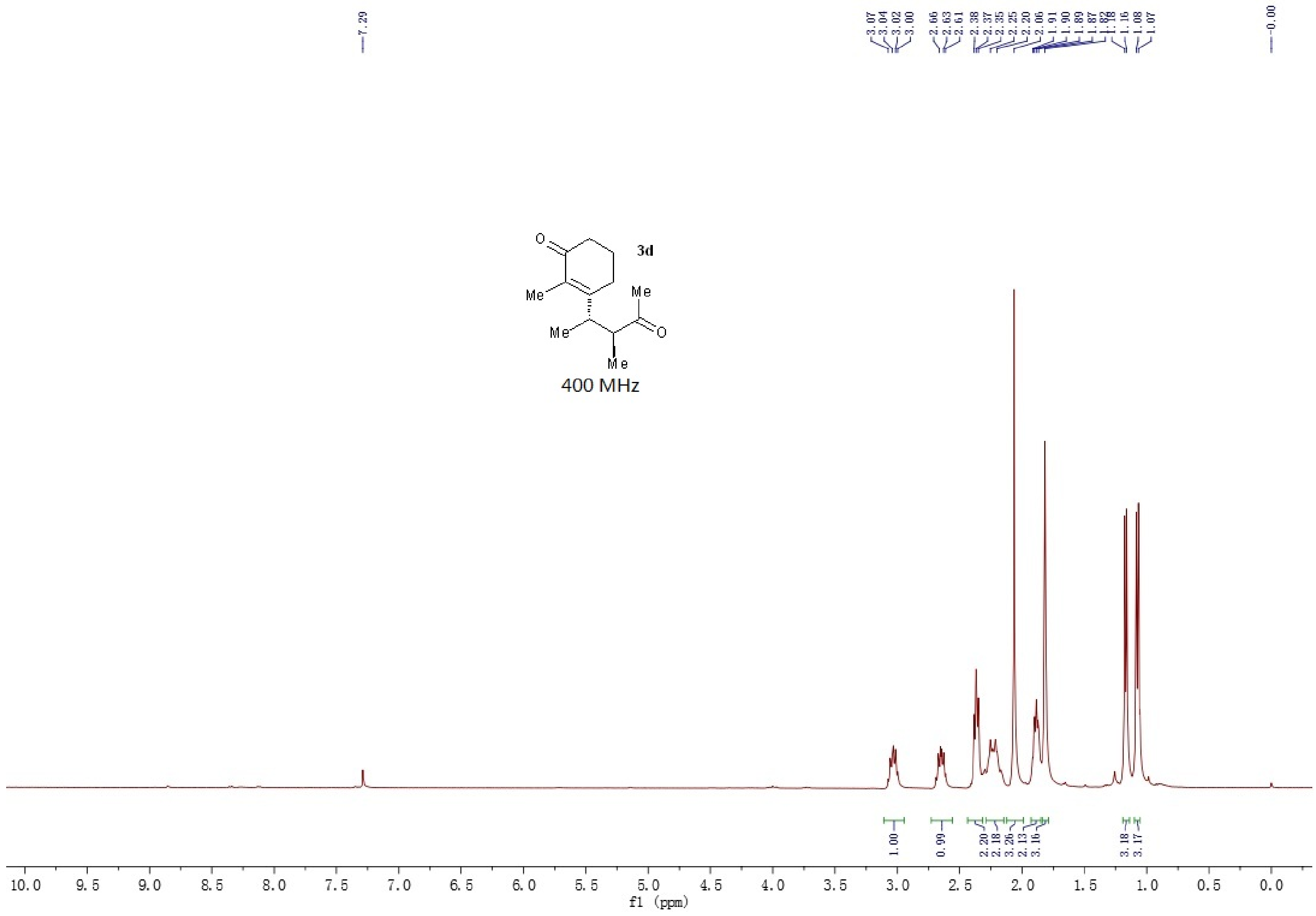
7.29
3.07 3.04 3.02 3.00 2.66 2.63 2.61 2.38 2.37 2.35 2.25 2.20 2.06 1.91 1.90 1.89 1.87 1.82 1.18 1.16 1.08 1.07
0.00
O
Me
Me
Me
Me
O
3d
400 MHz
1.00 0.99 2.20 2.18 3.26 2.13 3.16 3.18 3.17
10.0 9.5 9.0 8.5 8.0 7.5 7.0 6.5 6.0 5.5 5.0 4.5 4.0 3.5 3.0 2.5 2.0 1.5 1.0 0.5 0.0
f1 (ppm)

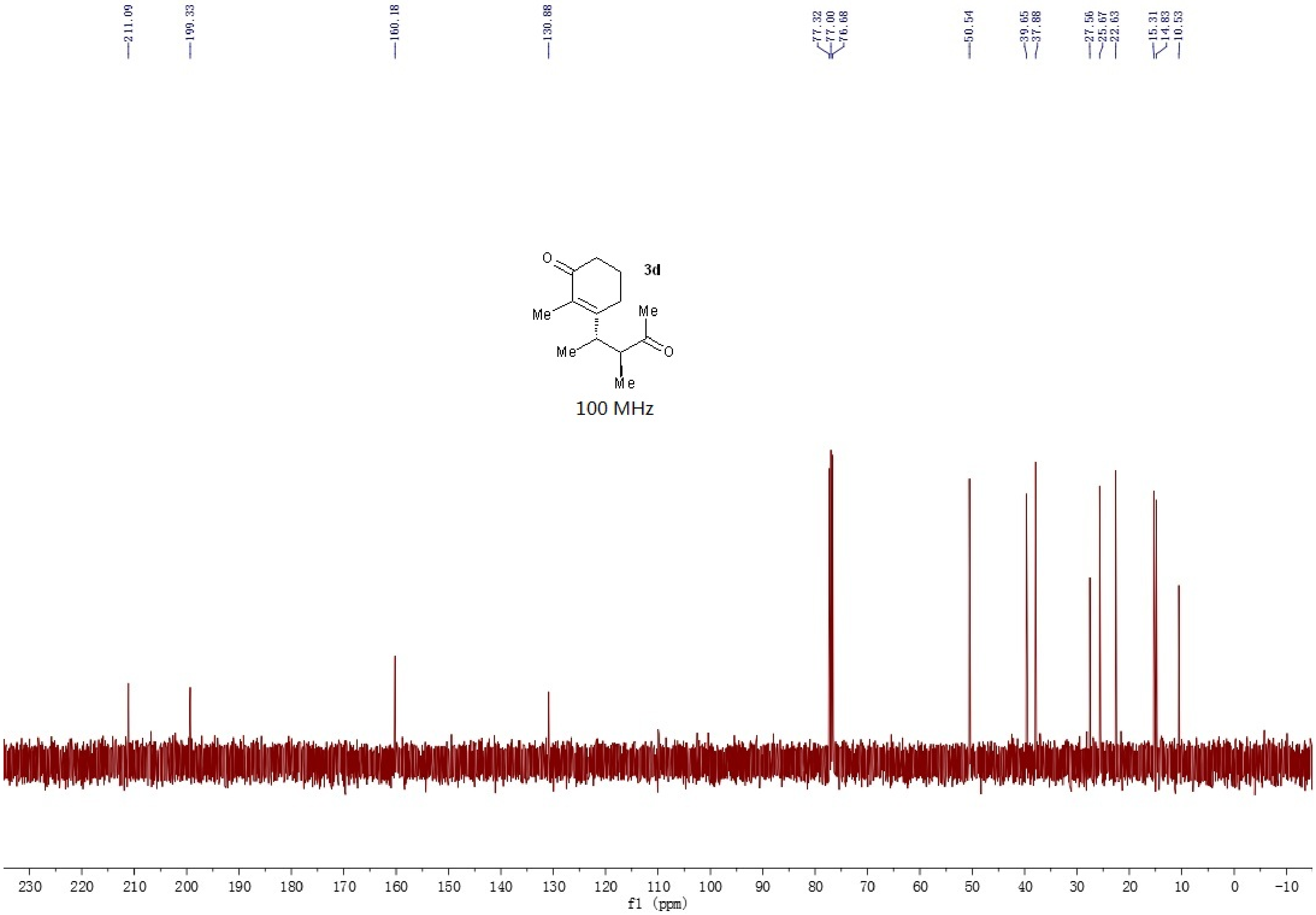
211.09
199.33
160.18
130.88
77.32
77.00
76.68
50.54
39.65
37.88
27.56
25.67
22.63
15.31
14.83
10.53
O
3d
Me
Me
Me
O
Me
100 MHz
230 220 210 200 190 180 170 160 150 140 130 120 110 100 90 80 70 60 50 40 30 20 10 0 -10
f1 (ppm)

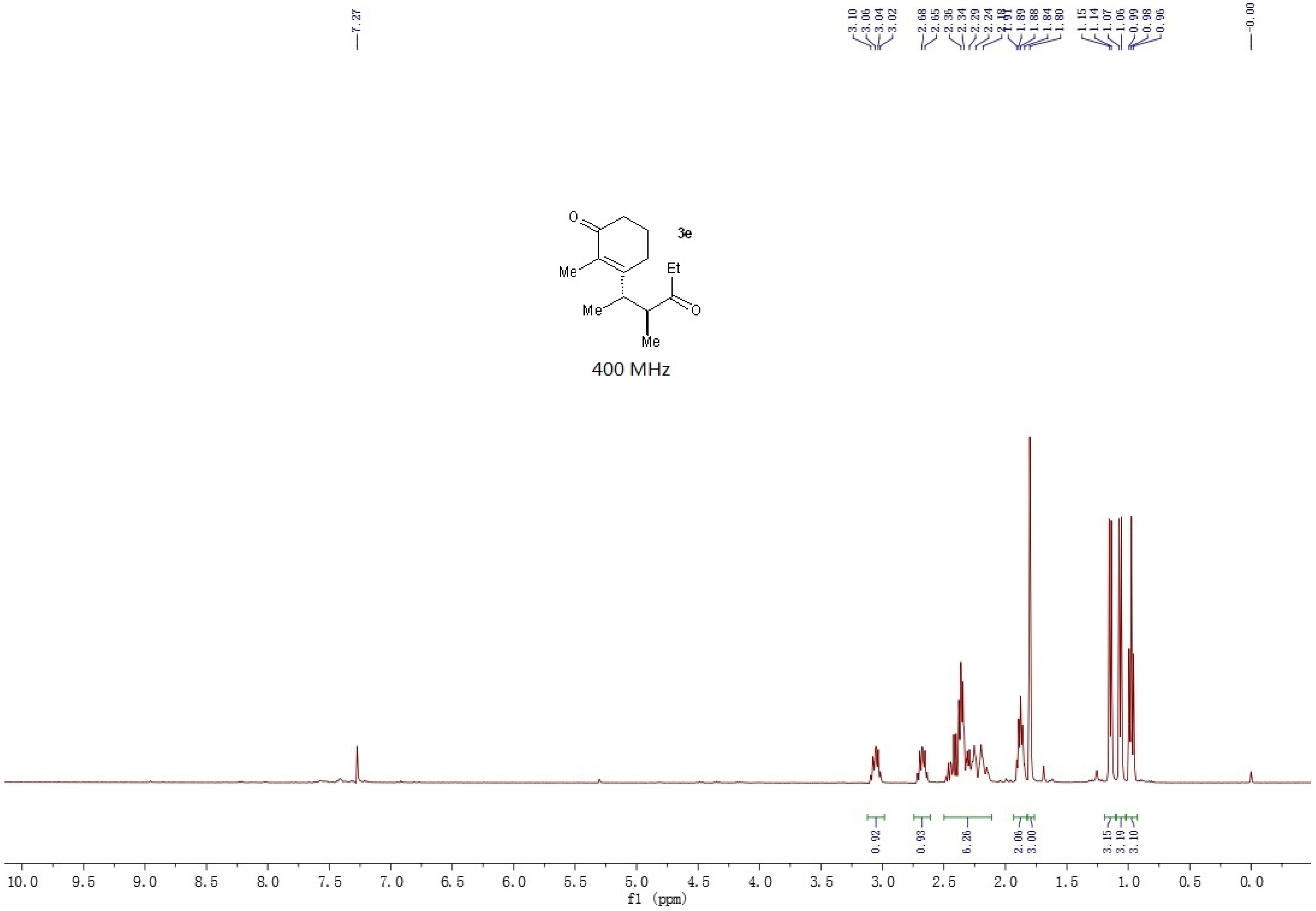
7.27
3.10
3.06
3.04
3.02
2.68
2.65
2.36
2.34
2.29
2.24
2.18
1.91
1.89
1.88
1.84
1.80
1.15
1.14
1.07
1.06
0.99
0.98
0.96
0.00
O
Me
Me
Et
O
Me
3e
400 MHz
0.92
0.93
6.26
2.06
3.00
3.15
3.19
3.10
10.0 9.5 9.0 8.5 8.0 7.5 7.0 6.5 6.0 5.5 5.0 4.5 4.0 3.5 3.0 2.5 2.0 1.5 1.0 0.5 0.0
f1 (ppm)

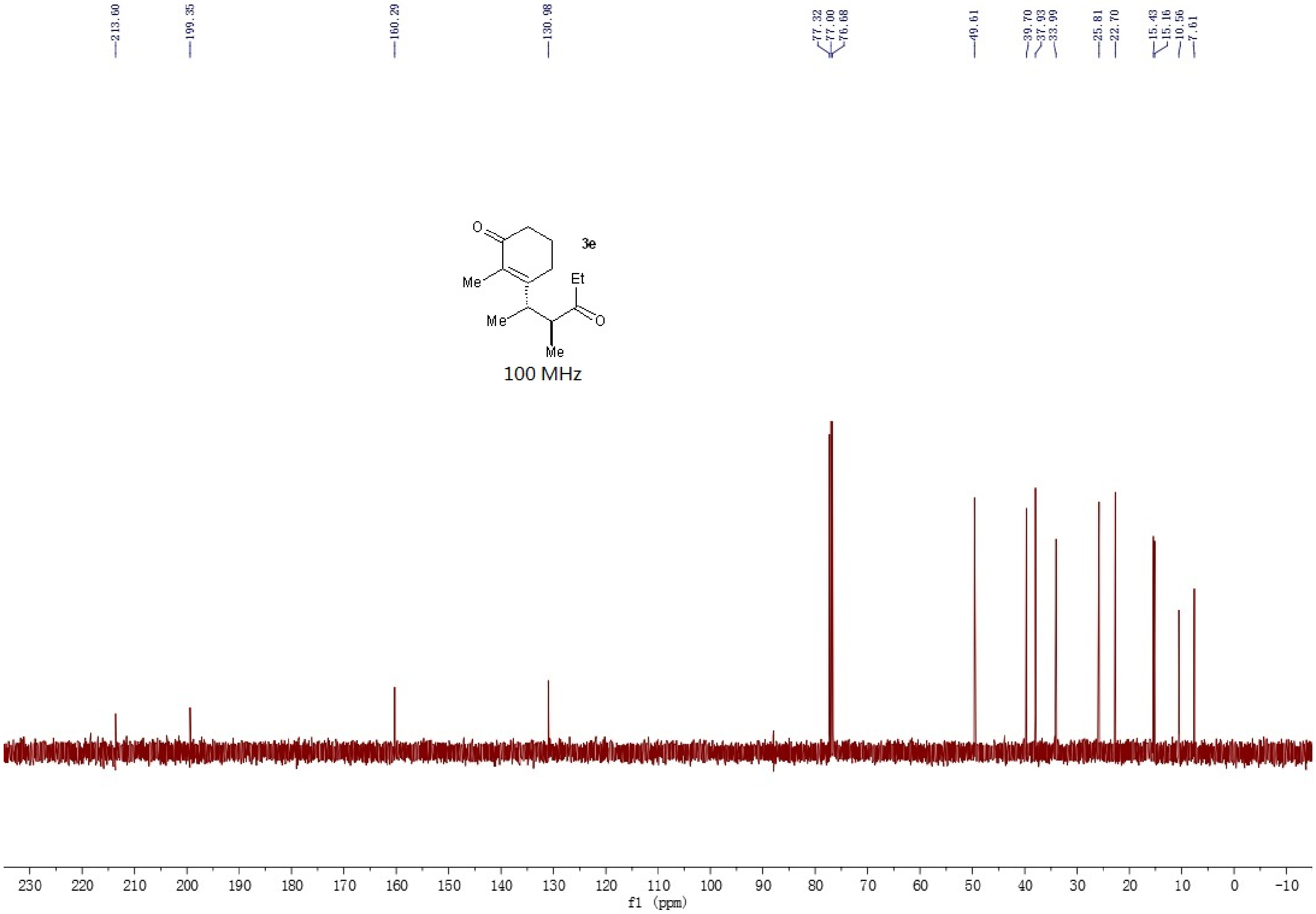
213.60
199.35
160.29
130.98
77.32
77.00
76.68
49.61
39.70
37.93
33.99
25.81
22.70
15.43
15.16
10.56
7.61
O
3e
Me
Et
Me
O
Me
100 MHz
230 220 210 200 190 180 170 160 150 140 130 120 110 100 90 80 70 60 50 40 30 20 10 0 -10
f1 (ppm)

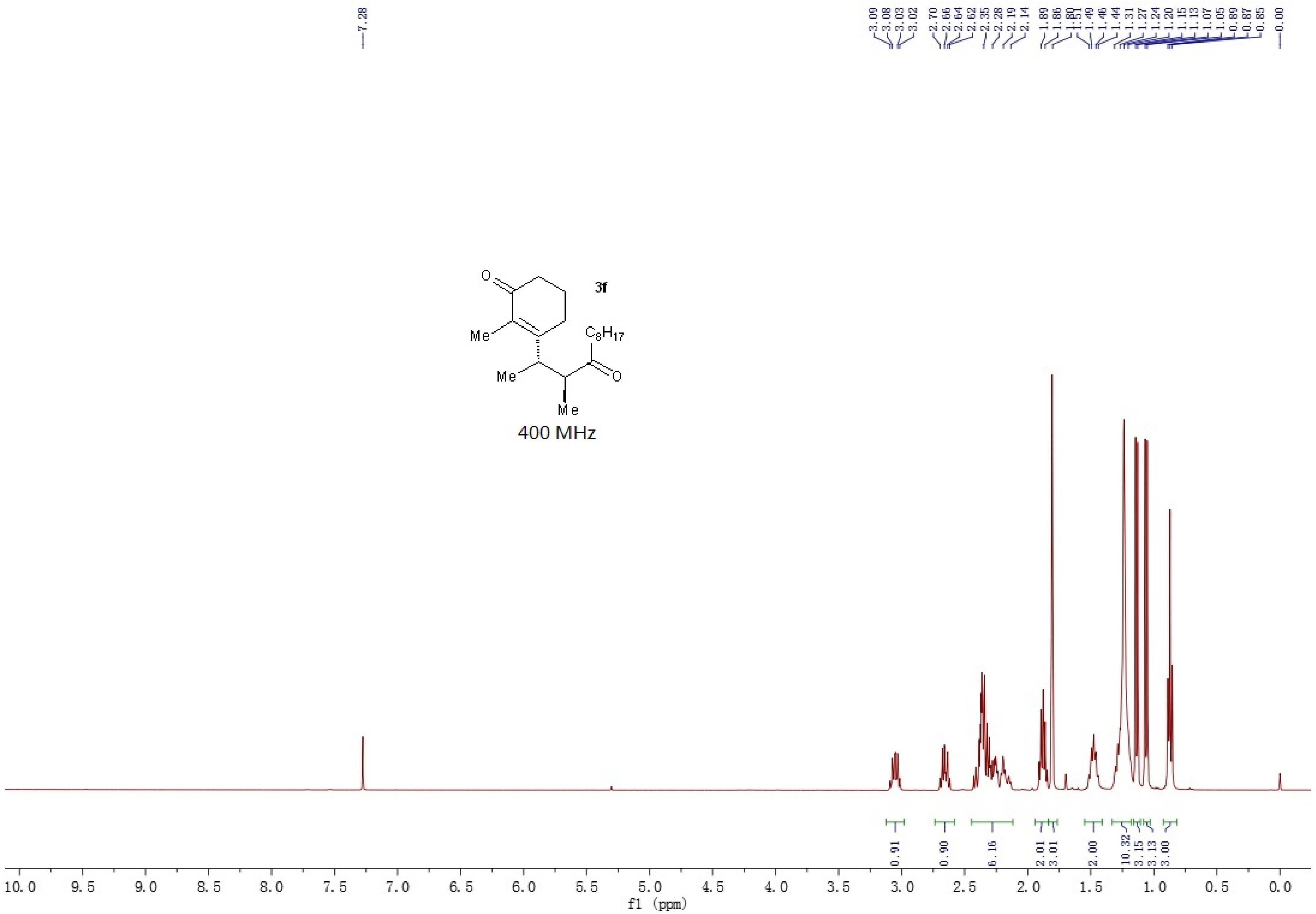
3f
400 MHz
f1 (ppm)

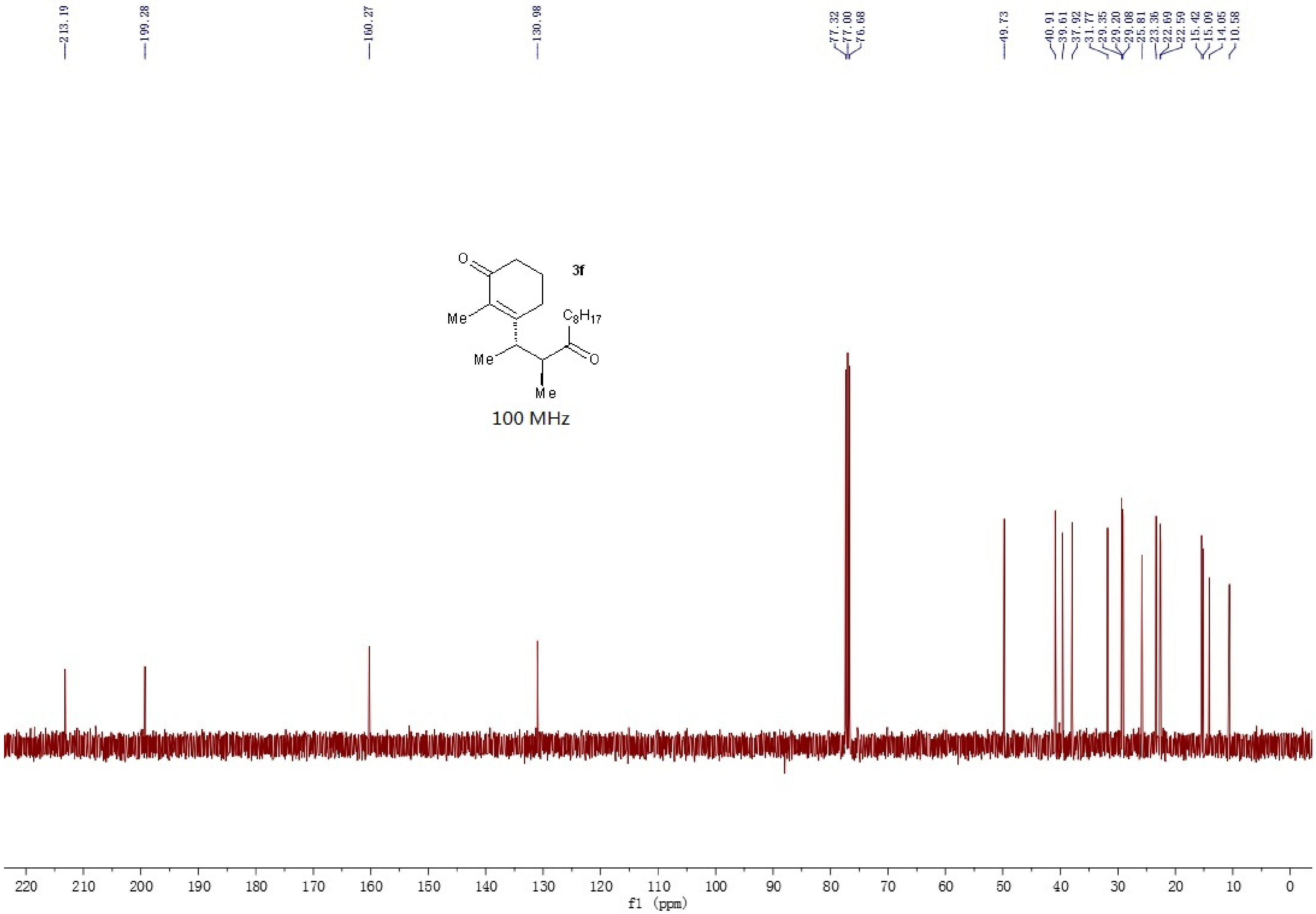

213.19
199.28
160.27
130.98
77.32
77.00
76.68
49.73
40.91
39.61
37.92
31.77
29.35
29.20
29.08
25.81
23.36
22.69
22.59
15.42
15.09
14.05
10.58
3f
Me
C_8H_{17}
Me
Me
100 MHz
220
210
200
190
180
170
160
150
140
130
120
110
100
90
80
70
60
50
40
30
20
10
0
f1 (ppm)

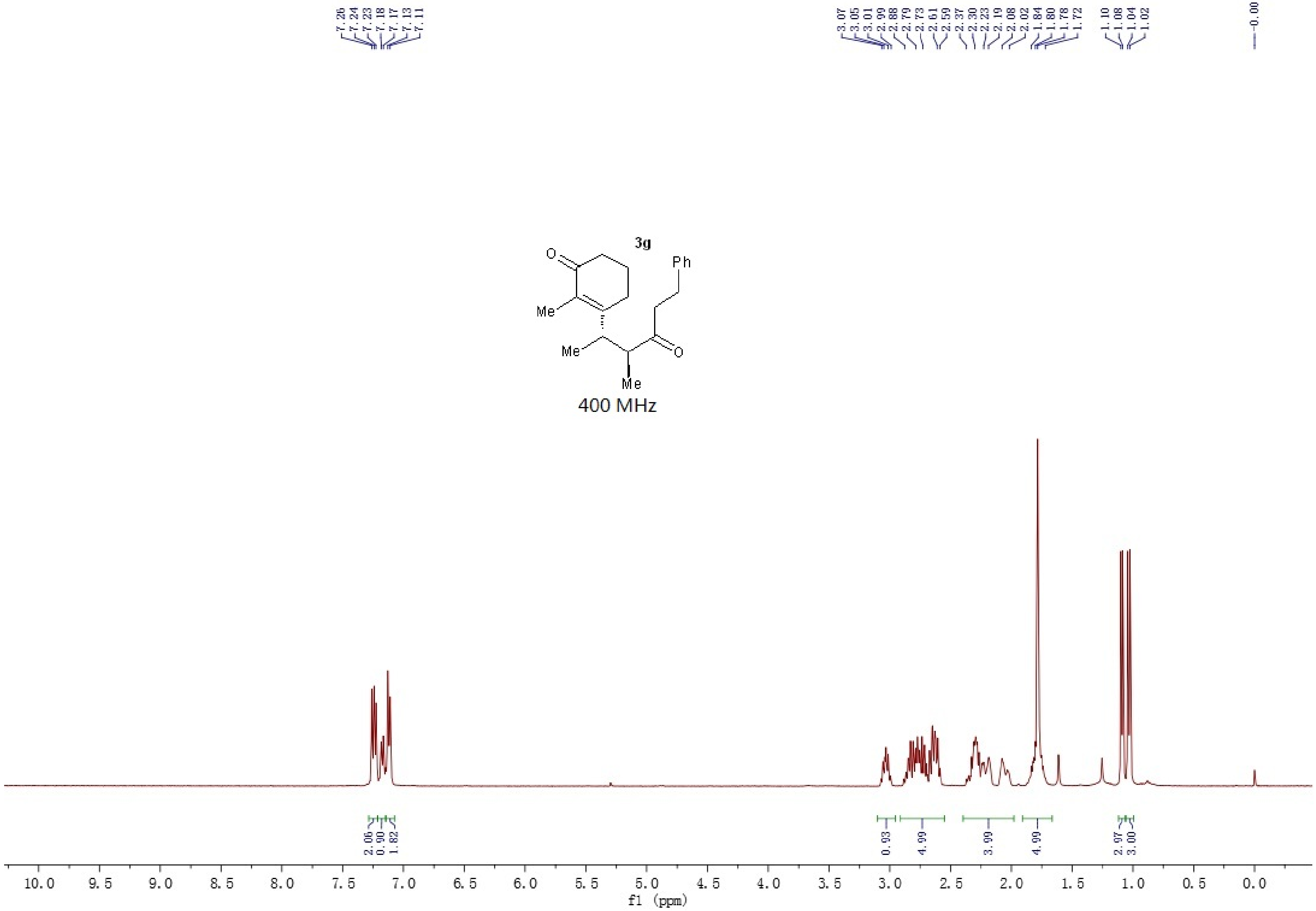

7.26
7.24
7.23
7.18
7.17
7.13
7.11
3.07
3.05
3.01
2.99
2.88
2.79
2.73
2.61
2.59
2.37
2.30
2.23
2.19
2.08
2.02
1.84
1.80
1.78
1.72
1.10
1.08
1.04
1.02
0.00
3g
Ph
O
Me
Me
O
Me
400 MHz
2.06
0.90
1.82
0.93
4.99
3.99
4.99
2.97
3.00
10.0
9.5
9.0
8.5
8.0
7.5
7.0
6.5
6.0
5.5
5.0
4.5
4.0
3.5
3.0
2.5
2.0
1.5
1.0
0.5
0.0
f1 (ppm)

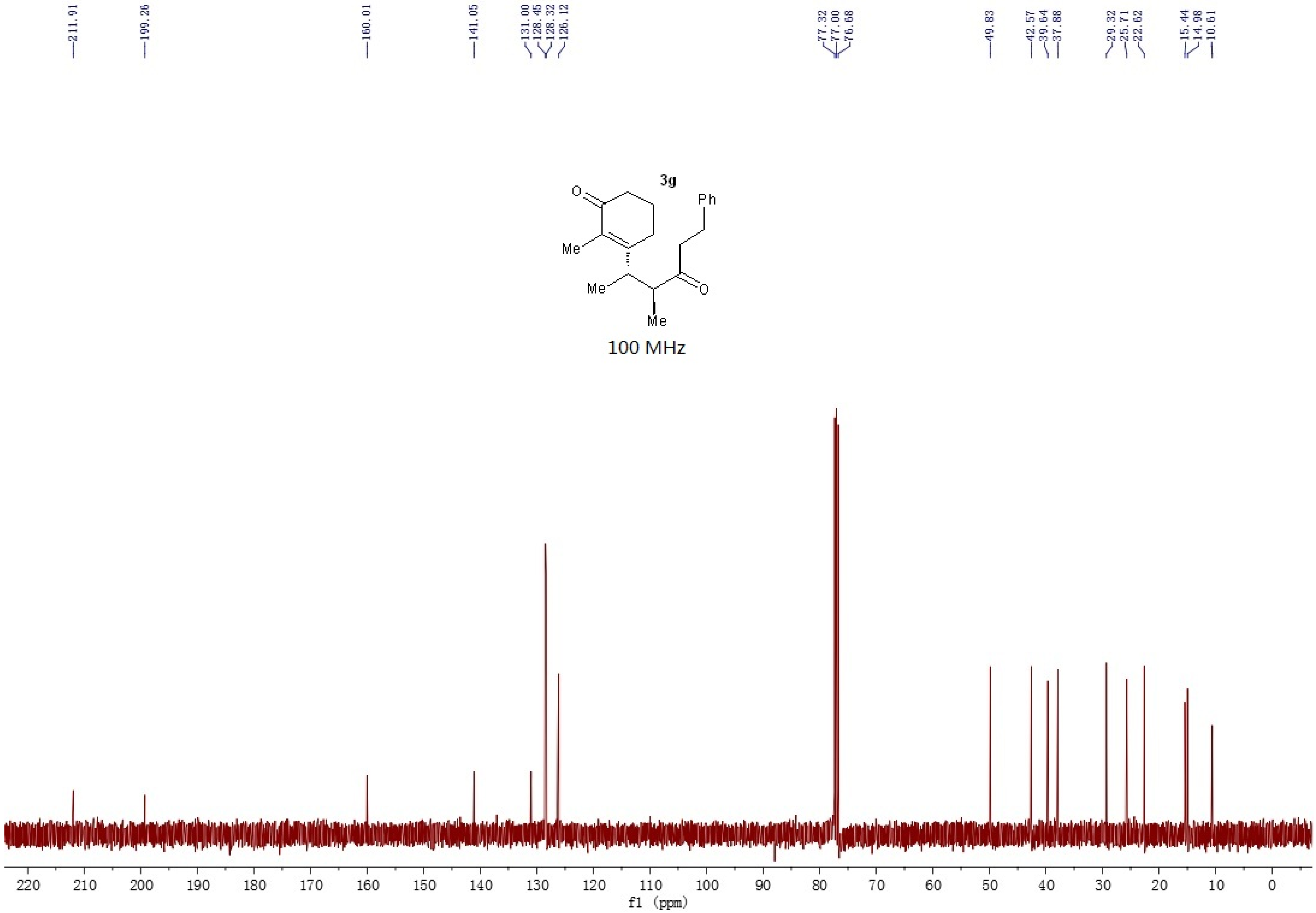
211.91
199.26
160.01
141.05
131.00
128.45
128.32
126.12
77.32
77.00
76.68
49.83
42.57
39.64
37.88
29.32
25.71
22.62
15.44
14.98
10.61
3g
Ph
O
Me
Me
O
Me
100 MHz
220
210
200
190
180
170
160
150
140
130
120
110
100
90
80
70
60
50
40
30
20
10
0
f1 (ppm)

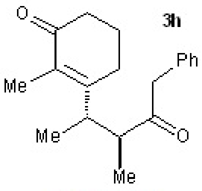
3h
Ph
O
Me
Me
O
Me

400 MHz

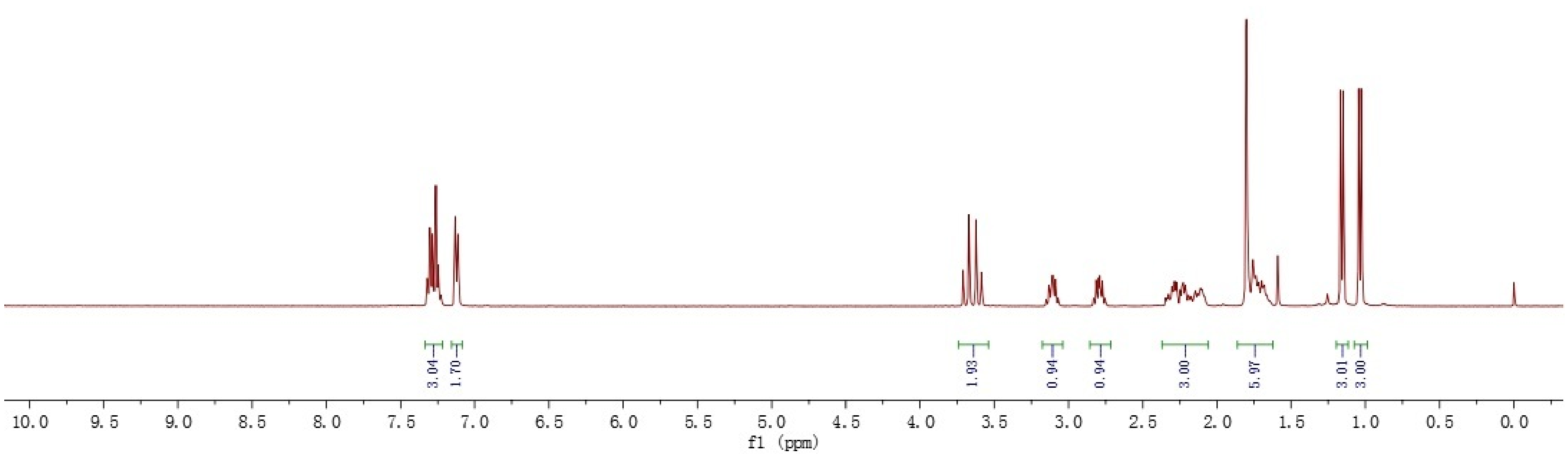
7.32 7.30 7.28 7.26 7.25 7.13 7.11
3.71 3.67 3.62 3.59 3.15 3.13 3.09 3.07 2.83 2.79 2.77 2.76 2.34 2.21 2.15 2.08 1.80 1.74 1.70 1.66 1.17 1.15 1.05 1.03
0.00
3.04 1.70 1.93 0.94 0.94 3.00 5.97 3.01 3.00
10.0 9.5 9.0 8.5 8.0 7.5 7.0 6.5 6.0 5.5 5.0 4.5 4.0 3.5 3.0 2.5 2.0 1.5 1.0 0.5 0.0
f1 (ppm)

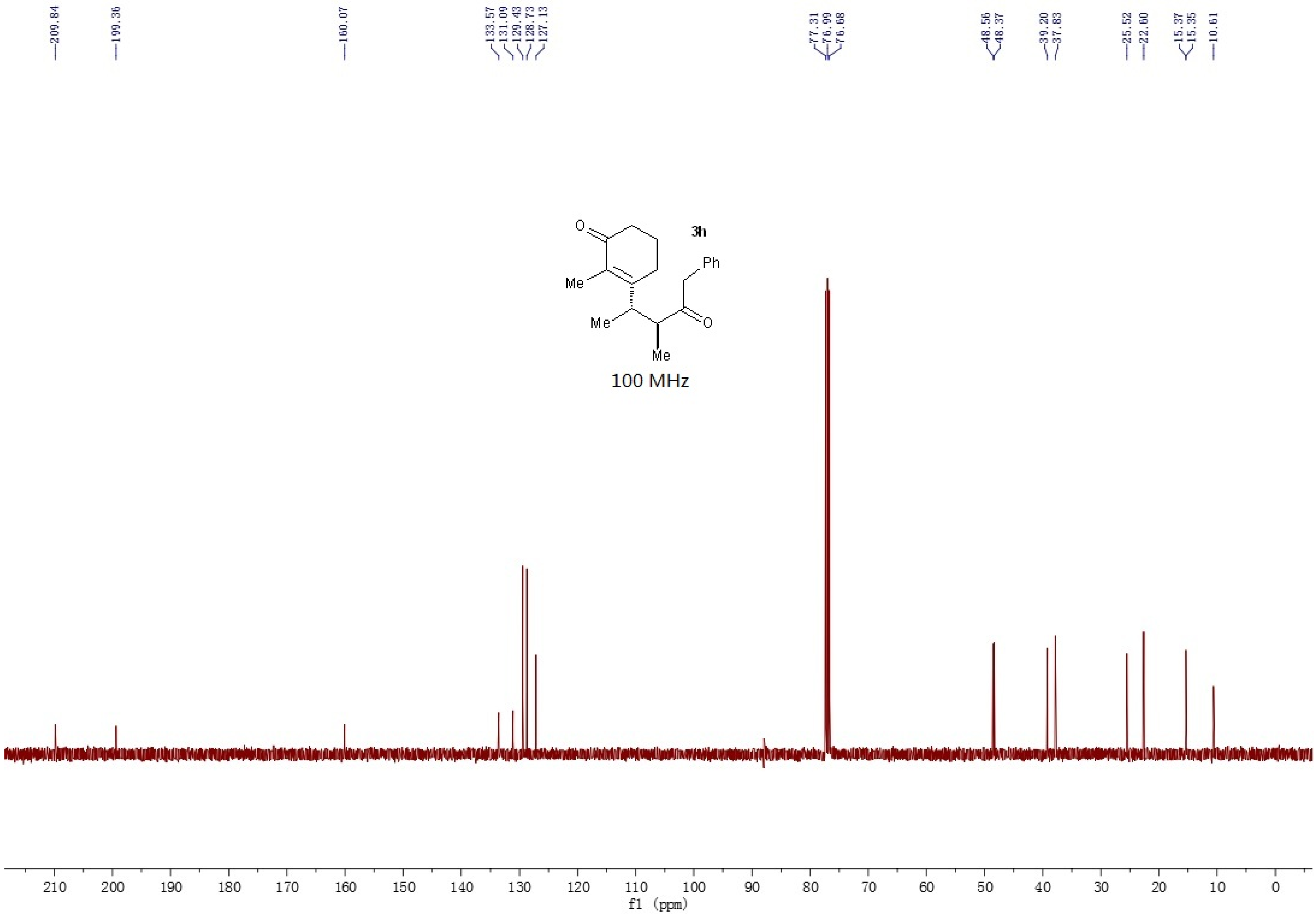
209.84
199.36
160.07
133.57
131.09
129.43
128.73
127.13
77.31
76.99
76.68
48.56
48.37
39.20
37.83
25.52
22.60
15.37
15.35
10.61
3h
O
Me
Ph
Me
O
Me
100 MHz
210 200 190 180 170 160 150 140 130 120 110 100 90 80 70 60 50 40 30 20 10 0
f1 (ppm)

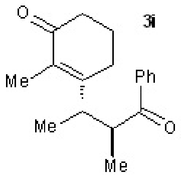
O
Me
Ph
O
Me
Me

3i

400 MHz

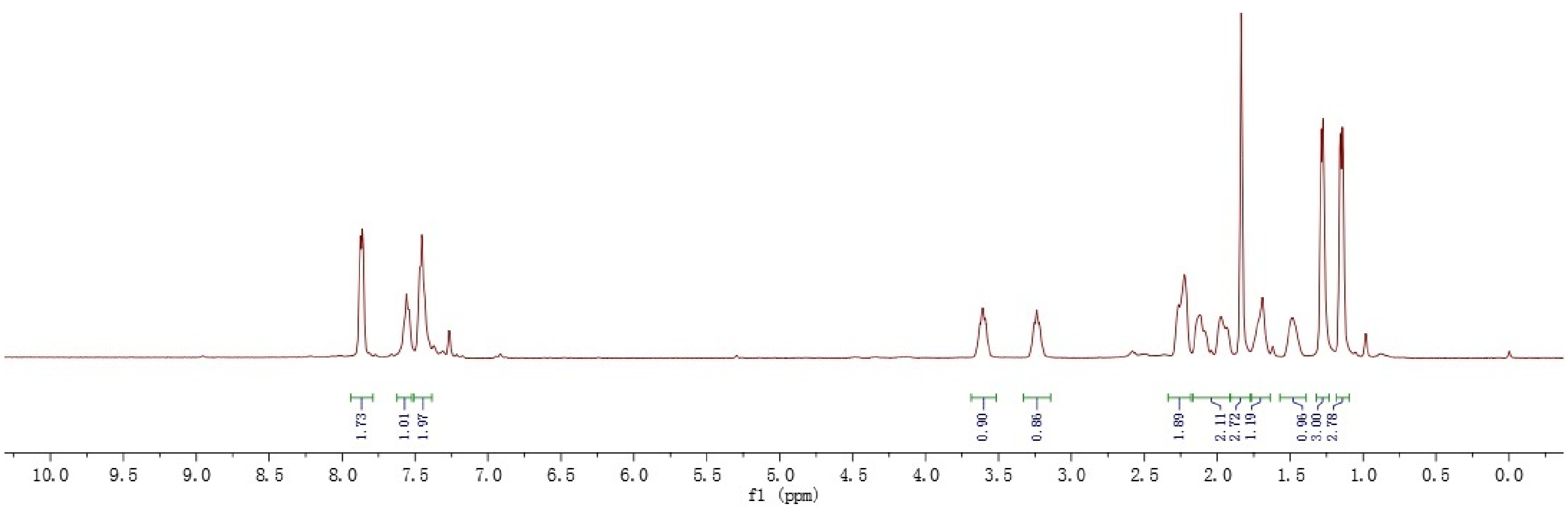
7.87 7.86 7.60 7.56 7.54 7.47 7.45 7.26
3.65 3.61 3.59 3.57 3.28 3.24 3.22 3.20
2.30 2.27 2.22 2.18 2.17 2.08 1.98 1.93 1.84 1.75 1.72 1.69 1.64 1.55 1.50 1.47 1.42 1.29 1.27 1.15 1.14
0.00
1.73
1.01
1.97
0.90
0.86
1.89
2.11
2.72
1.19
0.96
3.00
2.78
10.0 9.5 9.0 8.5 8.0 7.5 7.0 6.5 6.0 5.5 5.0 4.5 4.0 3.5 3.0 2.5 2.0 1.5 1.0 0.5 0.0
f1 (ppm)

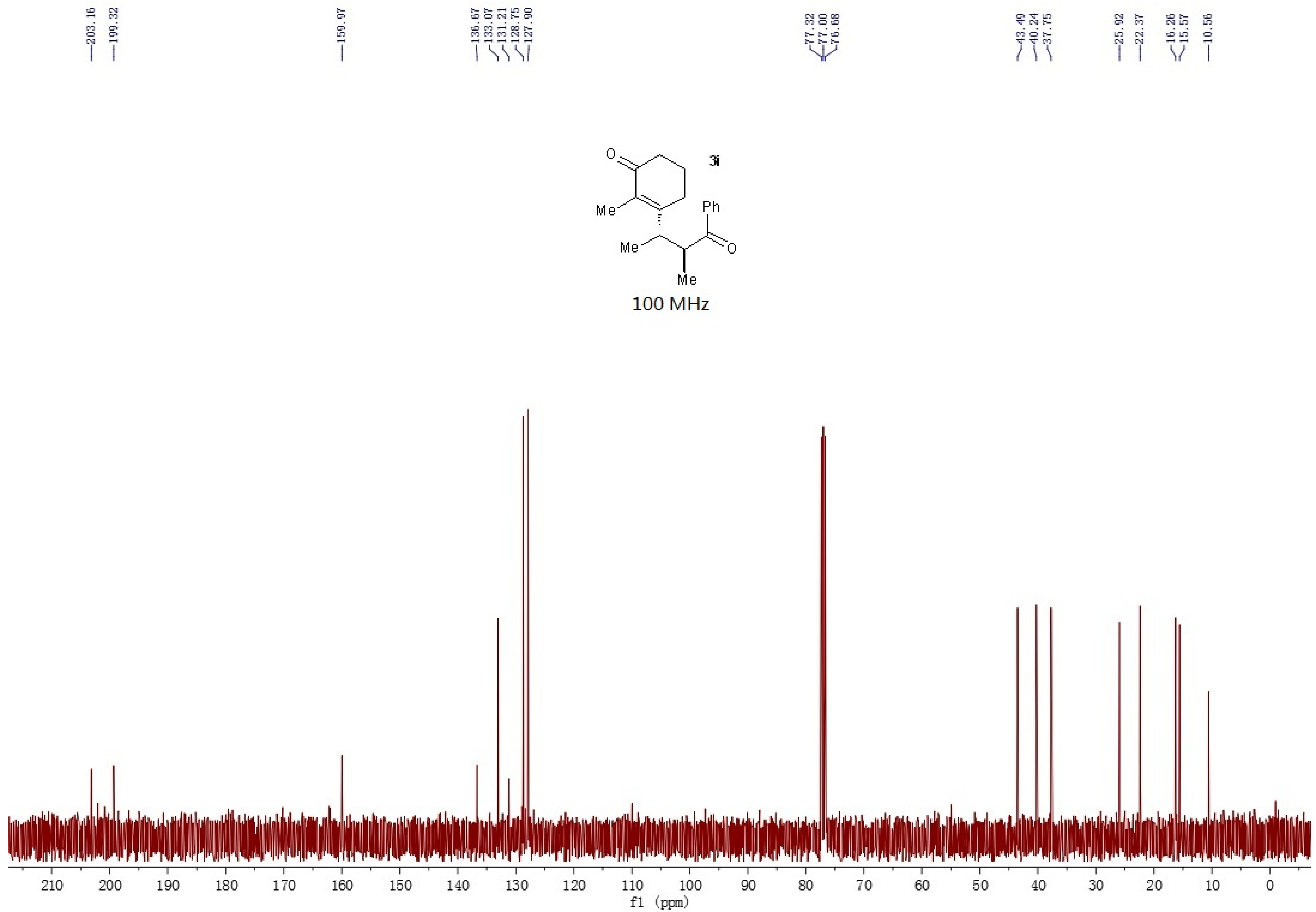

203.16
199.32
159.97
136.67
133.07
131.21
128.75
127.90
77.32
77.00
76.68
43.49
40.24
37.75
25.92
22.37
16.26
15.57
10.56
3i
Me
Me
Me
Ph
O
O
100 MHz
f1 (ppm)

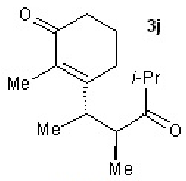

3j

400 MHz

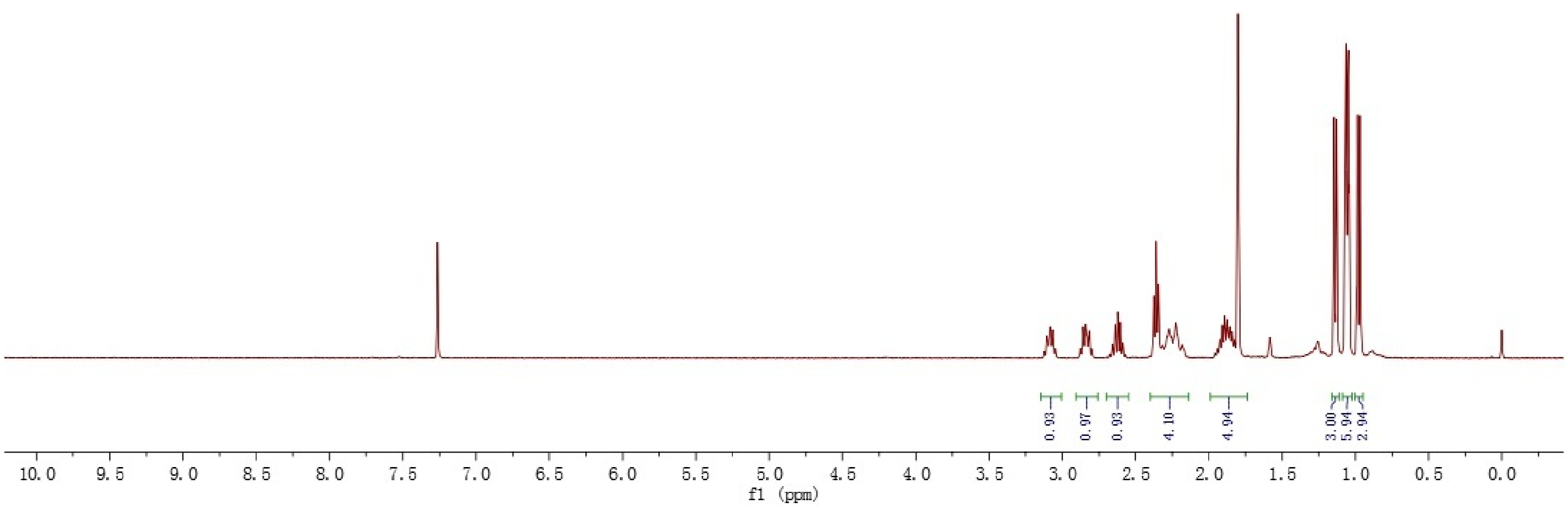
7.26
3.12
3.11
3.08
3.06
2.88
2.84
2.82
2.80
2.66
2.64
2.60
2.57
2.38
2.36
2.27
2.17
1.96
1.92
1.88
1.84
1.80
1.14
1.12
1.07
1.05
0.99
0.97
0.00
0.93
0.97
0.93
4.10
4.94
3.00
5.94
2.94
10.0
9.5
9.0
8.5
8.0
7.5
7.0
6.5
6.0
5.5
5.0
4.5
4.0
3.5
3.0
2.5
2.0
1.5
1.0
0.5
0.0
f1 (ppm)

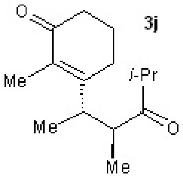
Me
Me
i-Pr
Me
O
O

3j

100 MHz

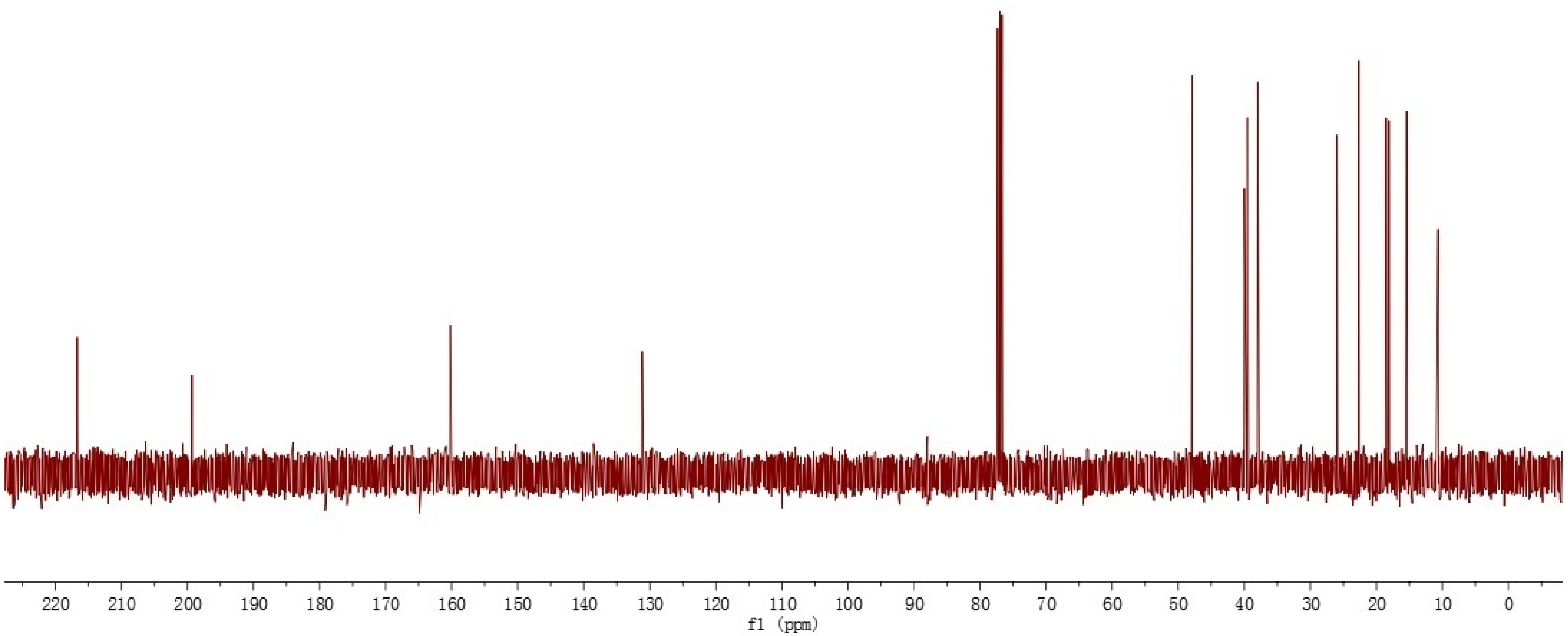
216.68
199.32
160.20
131.16
77.32
77.00
76.68
47.89
39.99
39.51
37.95
25.94
22.66
18.52
18.10
15.47
15.41
10.63
220 210 200 190 180 170 160 150 140 130 120 110 100 90 80 70 60 50 40 30 20 10 0
f1 (ppm)

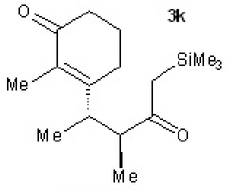

3k

400 MHz

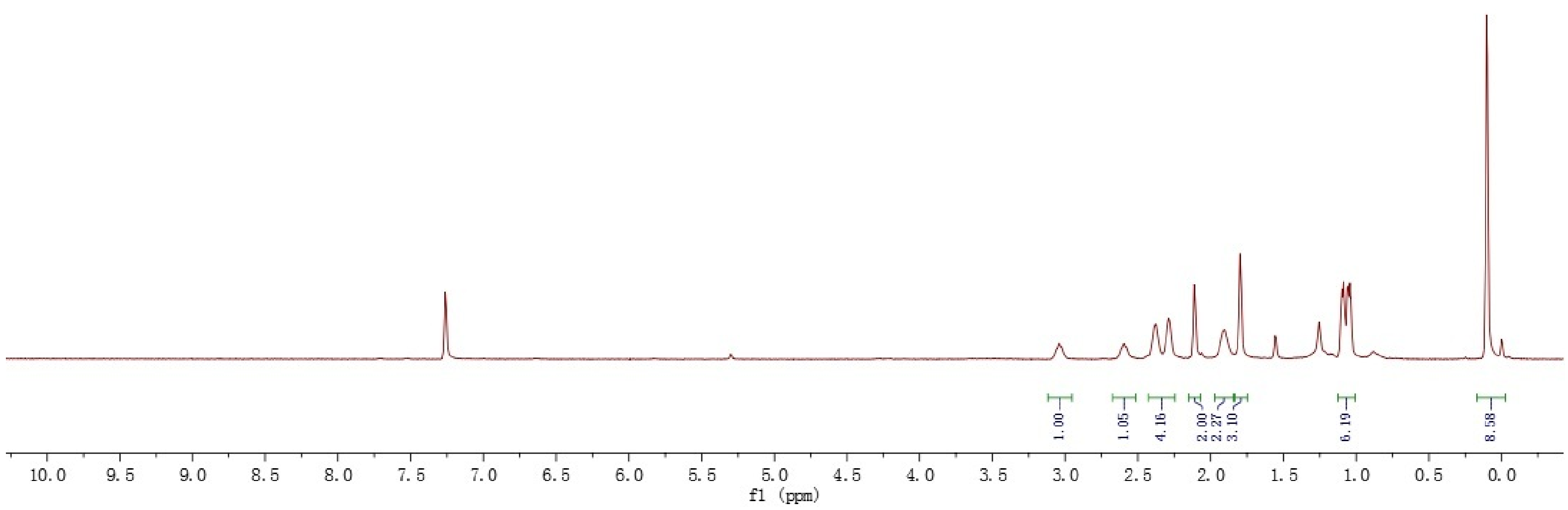
7.26
3.08
3.04
3.02
2.99
2.64
2.61
2.58
2.56
2.41
2.37
2.29
2.25
2.11
1.96
1.92
1.88
1.86
1.79
1.10
1.08
1.06
1.04
0.10
0.00
1.00
1.05
4.16
2.00
2.27
3.10
6.19
8.58
10.0
9.5
9.0
8.5
8.0
7.5
7.0
6.5
6.0
5.5
5.0
4.5
4.0
3.5
3.0
2.5
2.0
1.5
1.0
0.5
0.0
f1 (ppm)

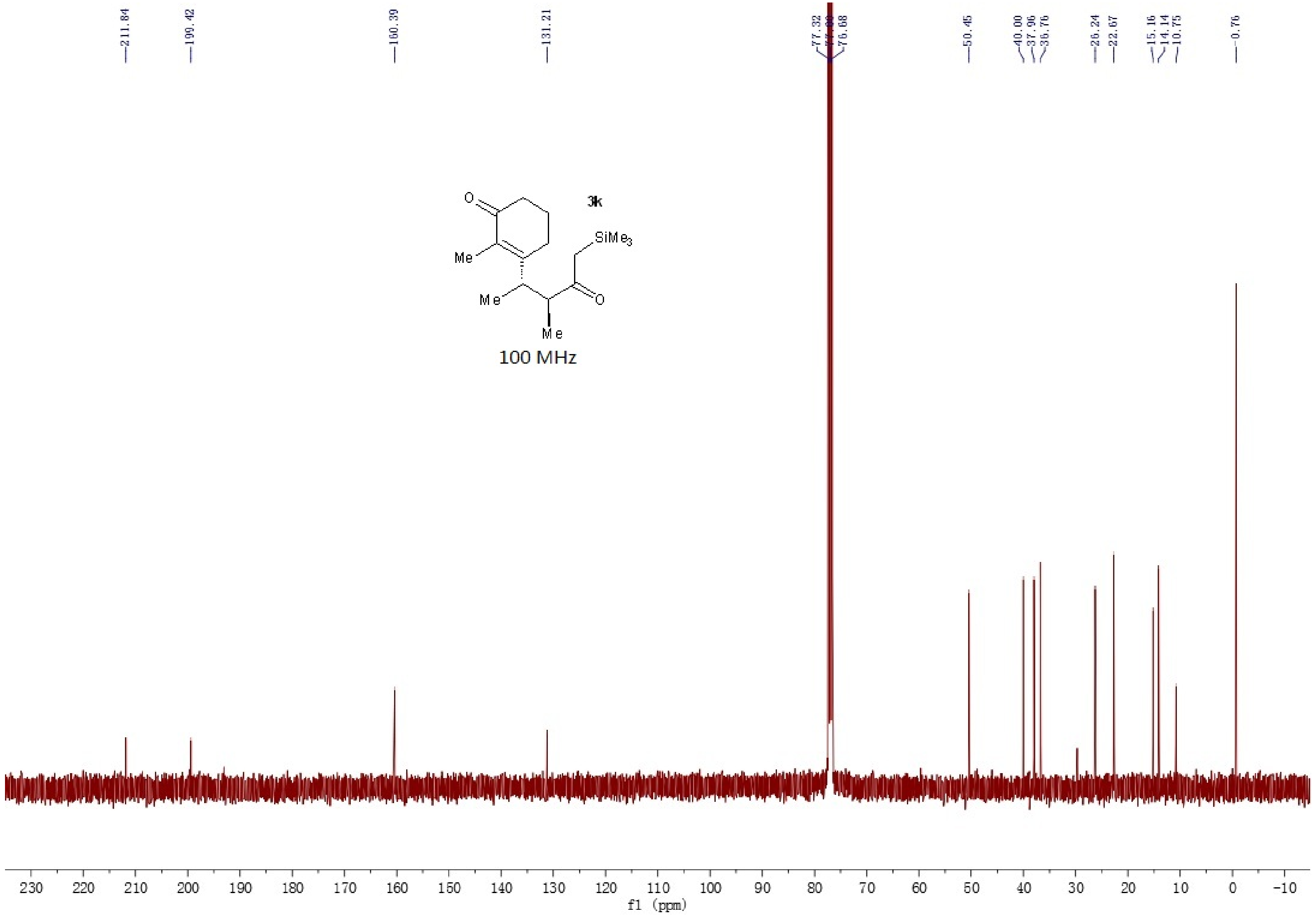
211.84
199.42
160.39
131.21
77.32
76.68
50.45
40.00
37.96
36.76
26.24
22.67
15.16
14.14
10.75
0.76
O
Me
SiMe3
Me
O
Me
3k
100 MHz
f1 (ppm)

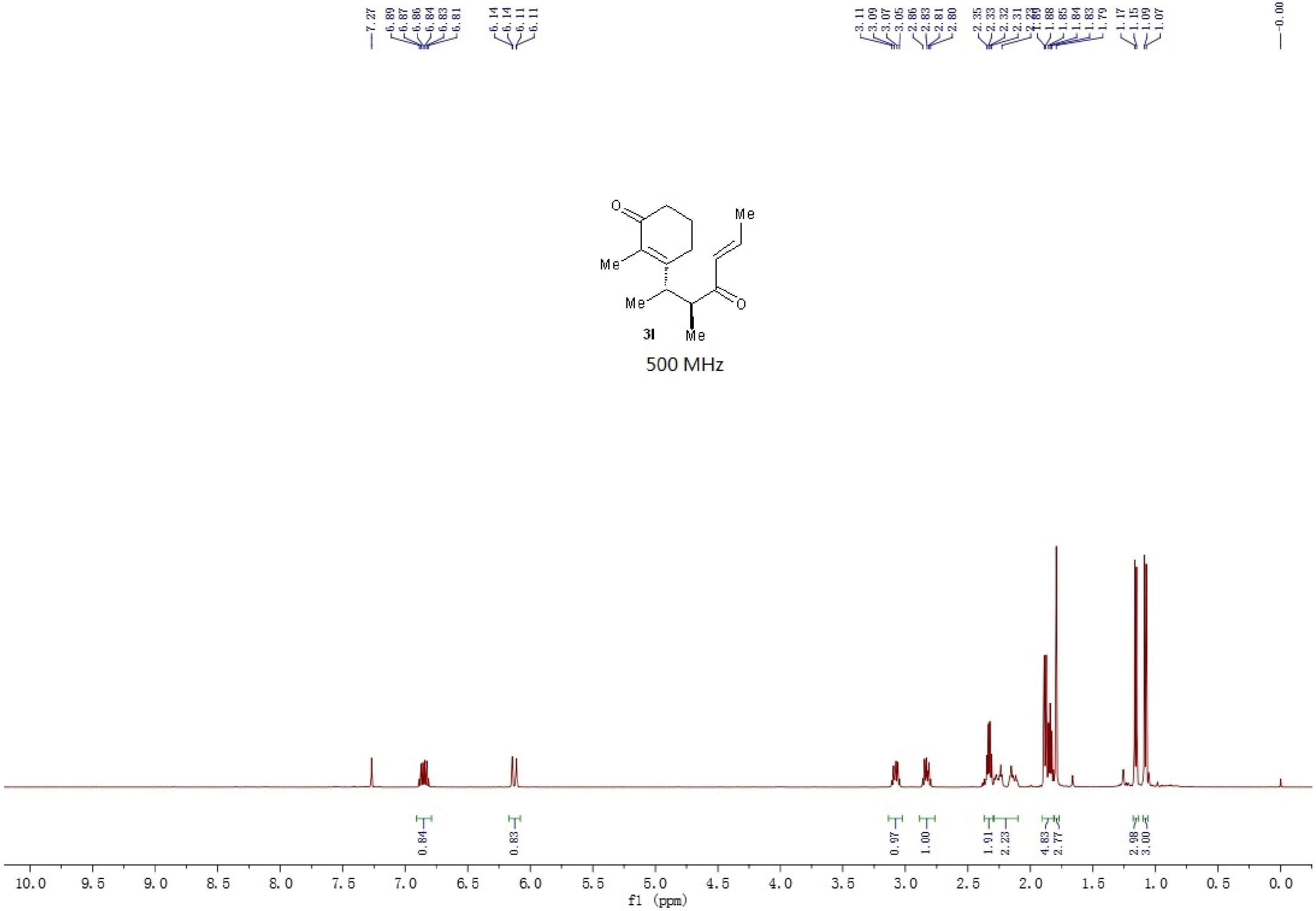
7.27
6.89
6.87
6.86
6.84
6.83
6.81
6.14
6.14
6.11
6.11
3.11
3.09
3.07
3.05
2.86
2.83
2.81
2.80
2.35
2.33
2.32
2.31
1.88
1.85
1.84
1.83
1.79
1.17
1.15
1.09
1.07
0.00
O
Me
Me
Me
O
Me
3l
500 MHz
0.84
0.83
0.97
1.00
1.91
2.23
4.83
2.77
2.98
3.00
10.0
9.5
9.0
8.5
8.0
7.5
7.0
6.5
6.0
5.5
5.0
4.5
4.0
3.5
3.0
2.5
2.0
1.5
1.0
0.5
0.0
f1 (ppm)

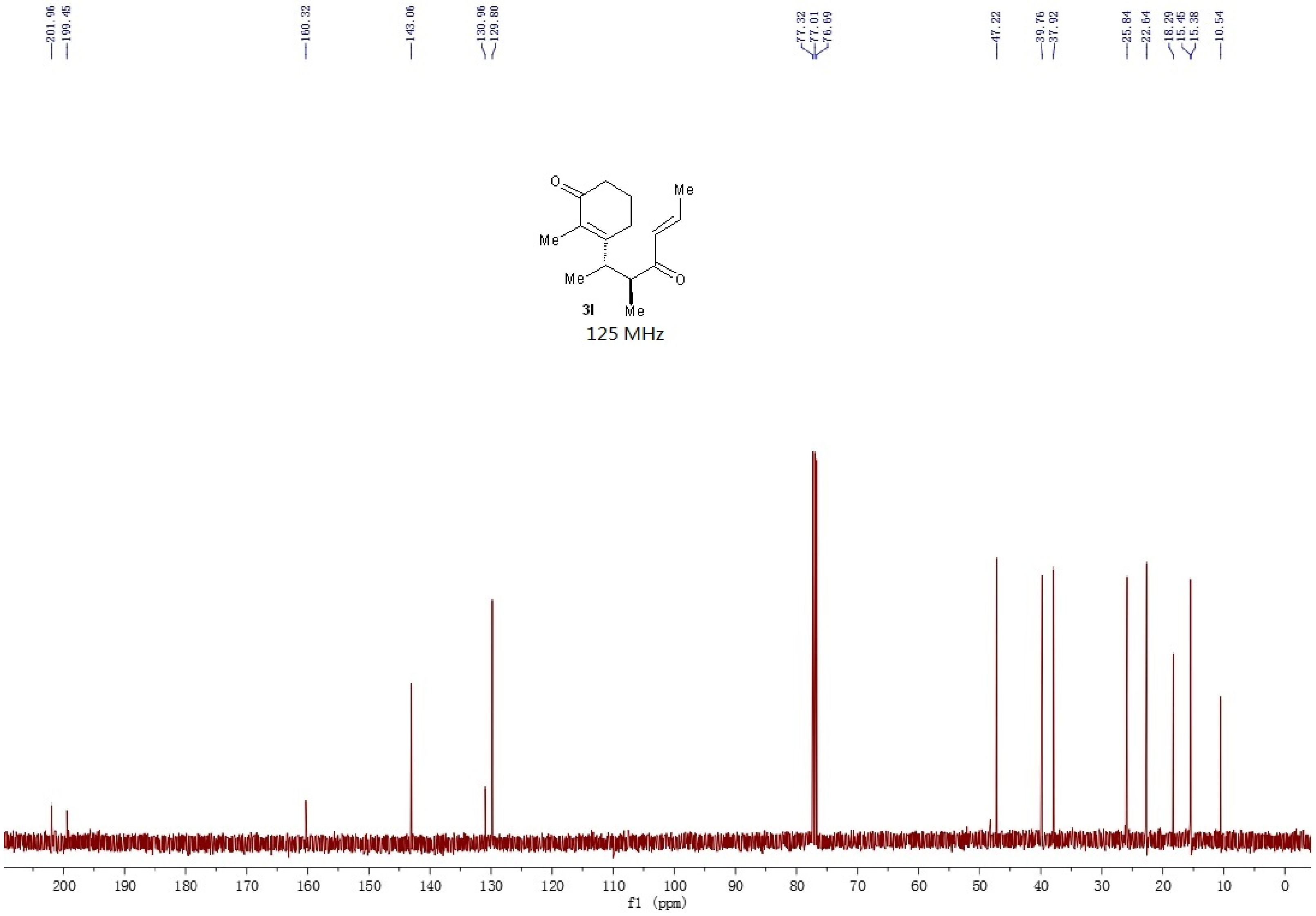

201.96
199.45
160.32
143.06
130.96
129.80
77.32
77.01
76.69
47.22
39.76
37.92
25.84
22.64
18.29
15.45
15.38
10.54
O
Me
Me
Me
O
Me
3l
125 MHz
200
190
180
170
160
150
140
130
120
110
100
90
80
70
60
50
40
30
20
10
0
f1 (ppm)

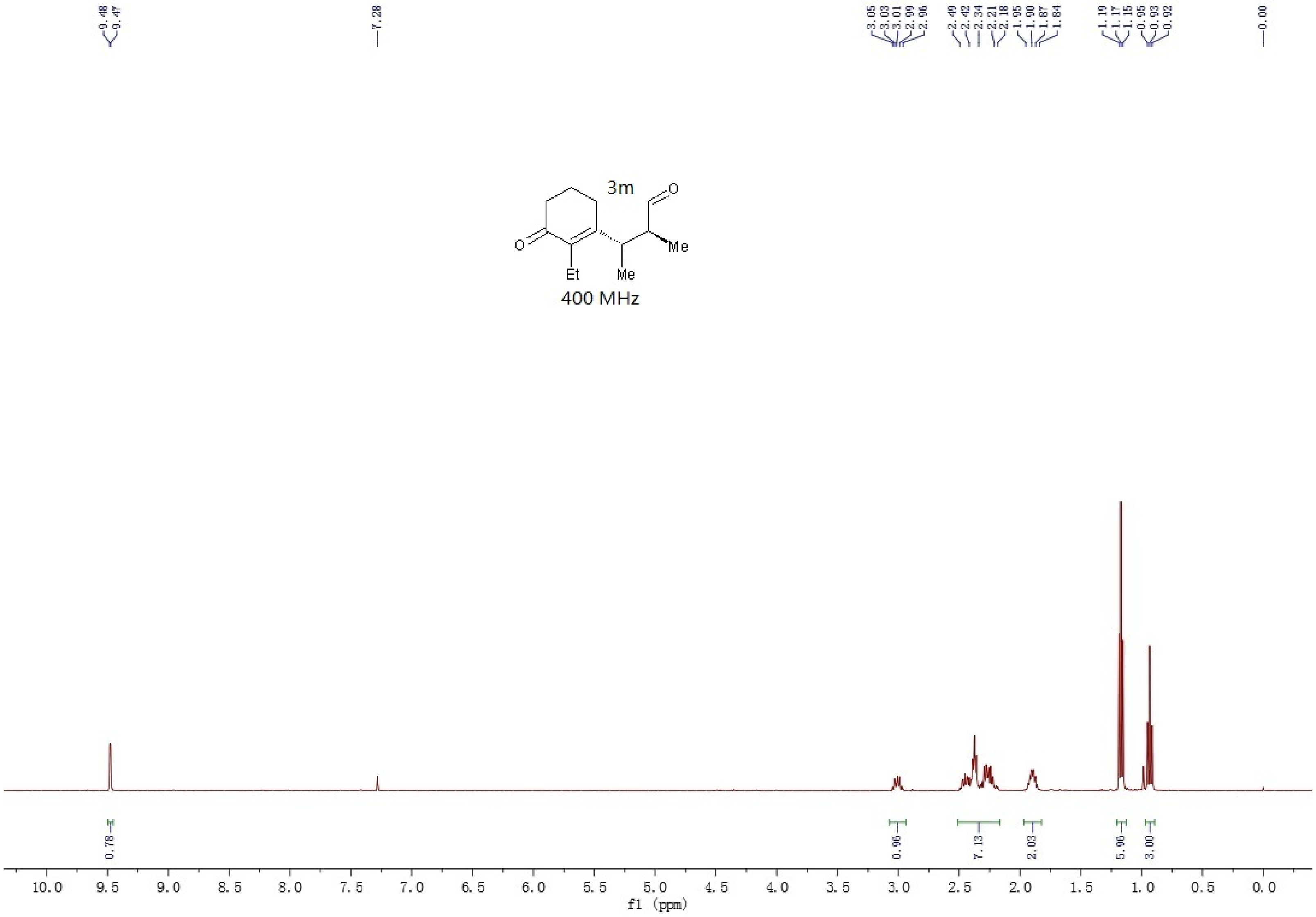
9.48
9.47
7.28
3.05
3.03
3.01
2.99
2.96
2.49
2.42
2.34
2.21
2.18
1.95
1.90
1.87
1.84
1.19
1.17
1.15
0.95
0.93
0.92
0.00
3m
O
O
Et
Me
Me
400 MHz
0.78
0.96
7.13
2.03
5.96
3.00
10.0
9.5
9.0
8.5
8.0
7.5
7.0
6.5
6.0
5.5
5.0
4.5
4.0
3.5
3.0
2.5
2.0
1.5
1.0
0.5
0.0
f1 (ppm)

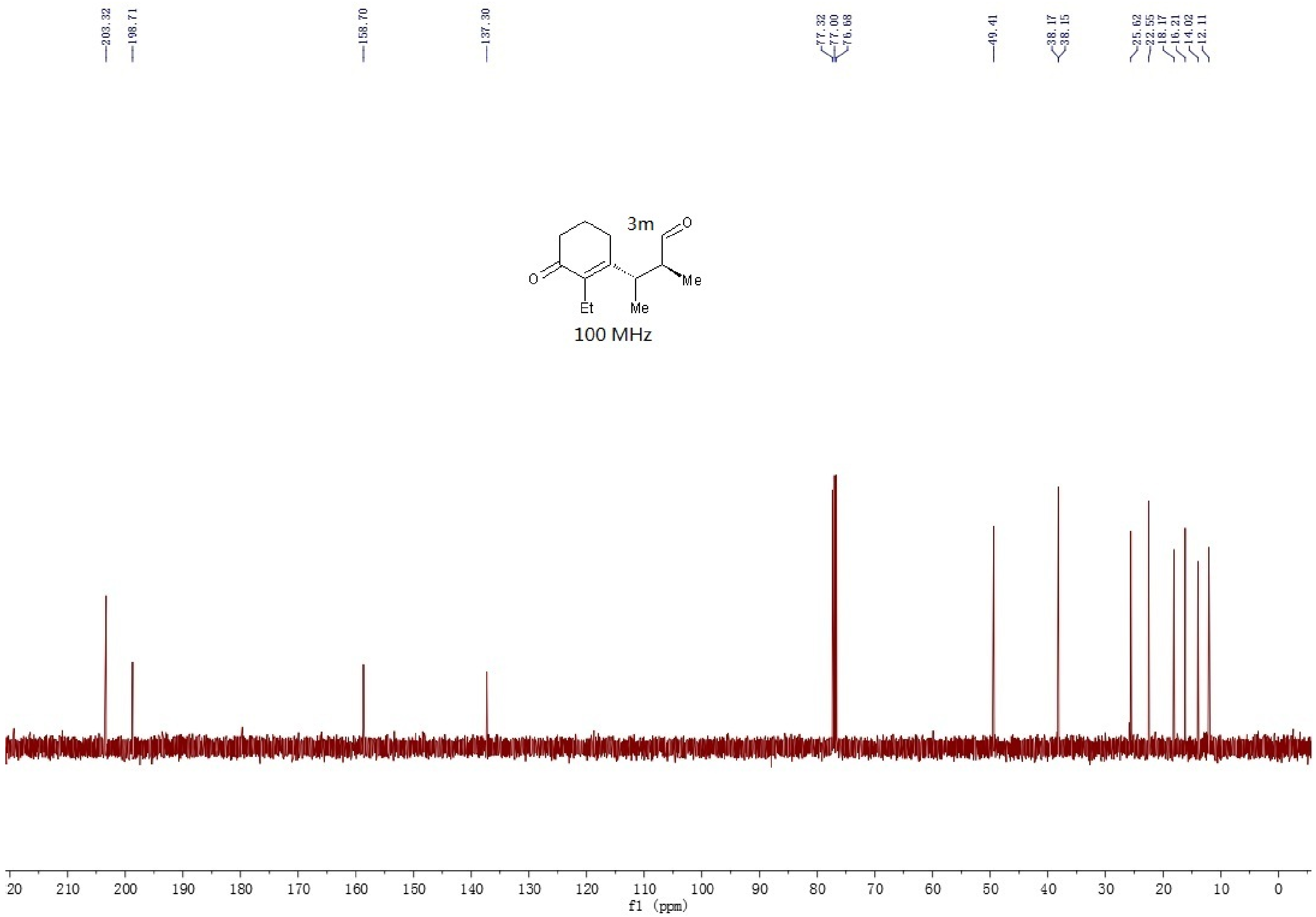
203.32
198.71
158.70
137.30
77.32
77.00
76.68
49.41
38.17
38.15
25.62
22.55
18.17
16.21
14.02
12.11
3m
O
O
Et
Me
Me
100 MHz
20 210 200 190 180 170 160 150 140 130 120 110 100 90 80 70 60 50 40 30 20 10 0
f1 (ppm)

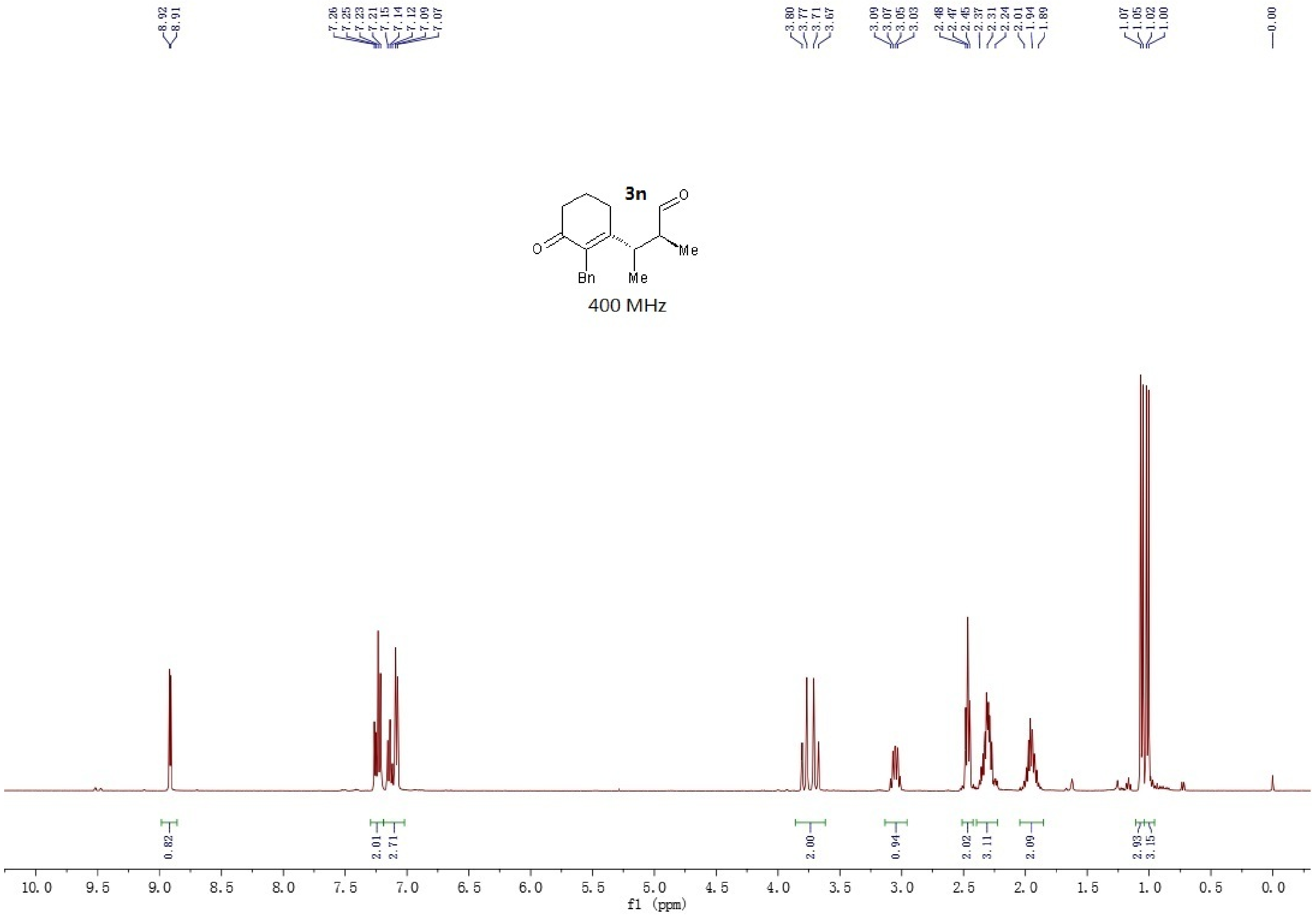
8.92
8.91
7.26
7.25
7.23
7.21
7.15
7.14
7.12
7.09
7.07
3.80
3.77
3.71
3.67
3.09
3.07
3.05
3.03
2.48
2.47
2.45
2.37
2.31
2.24
2.01
1.94
1.89
1.07
1.05
1.02
1.00
0.00
3n
O
O
Bn
Me
Me
400 MHz
0.82
2.01
2.71
2.00
0.94
2.02
3.11
2.09
2.93
3.15
10.0
9.5
9.0
8.5
8.0
7.5
7.0
6.5
6.0
5.5
5.0
4.5
4.0
3.5
3.0
2.5
2.0
1.5
1.0
0.5
0.0
f1 (ppm)

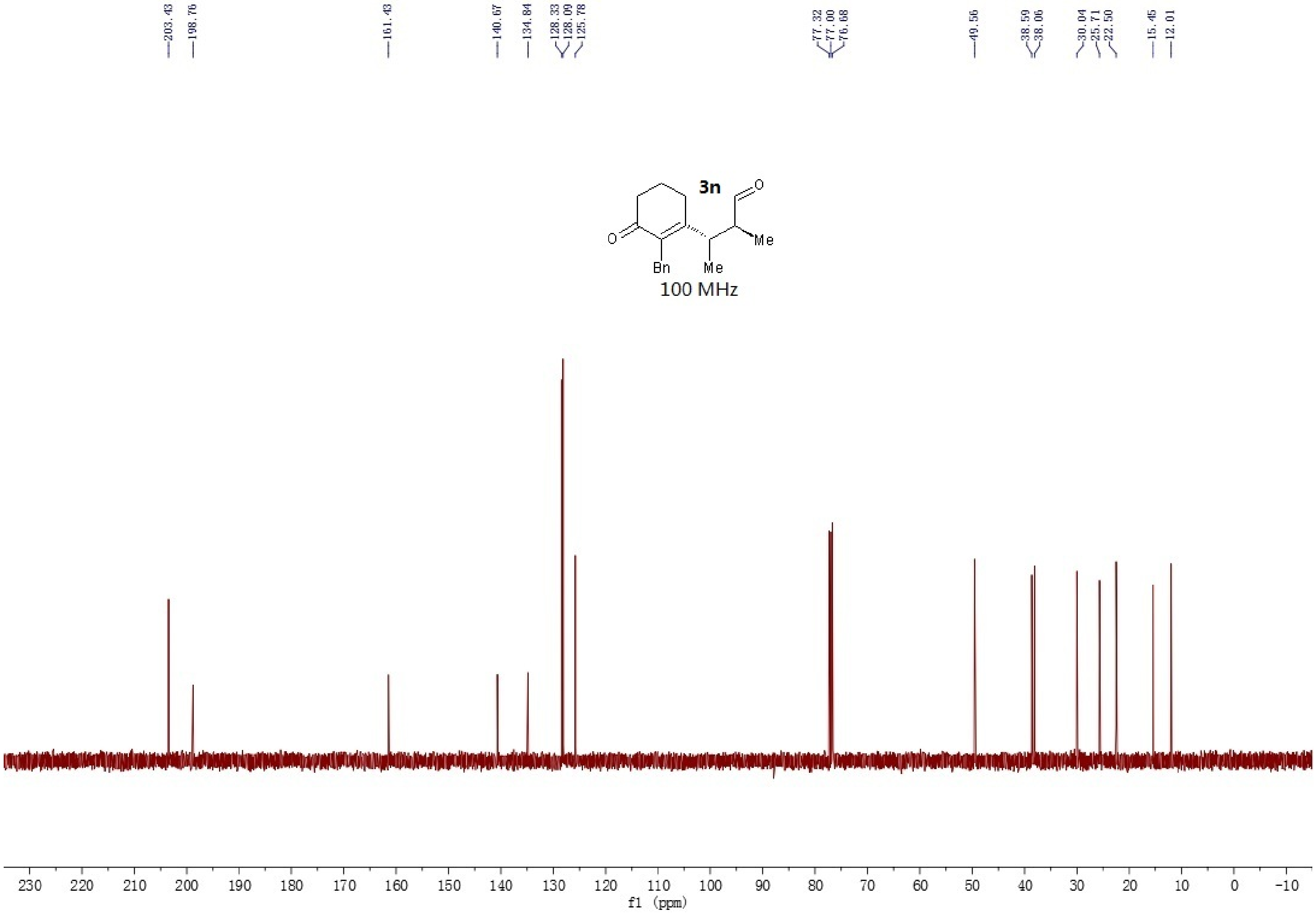
203.43
198.76
161.43
140.67
134.84
128.33
128.09
125.78
77.32
77.00
76.68
49.56
38.59
38.06
30.04
25.71
22.50
15.45
12.01
3n
O
O
Bn
Me
Me
100 MHz
230 220 210 200 190 180 170 160 150 140 130 120 110 100 90 80 70 60 50 40 30 20 10 0 -10
f1 (ppm)

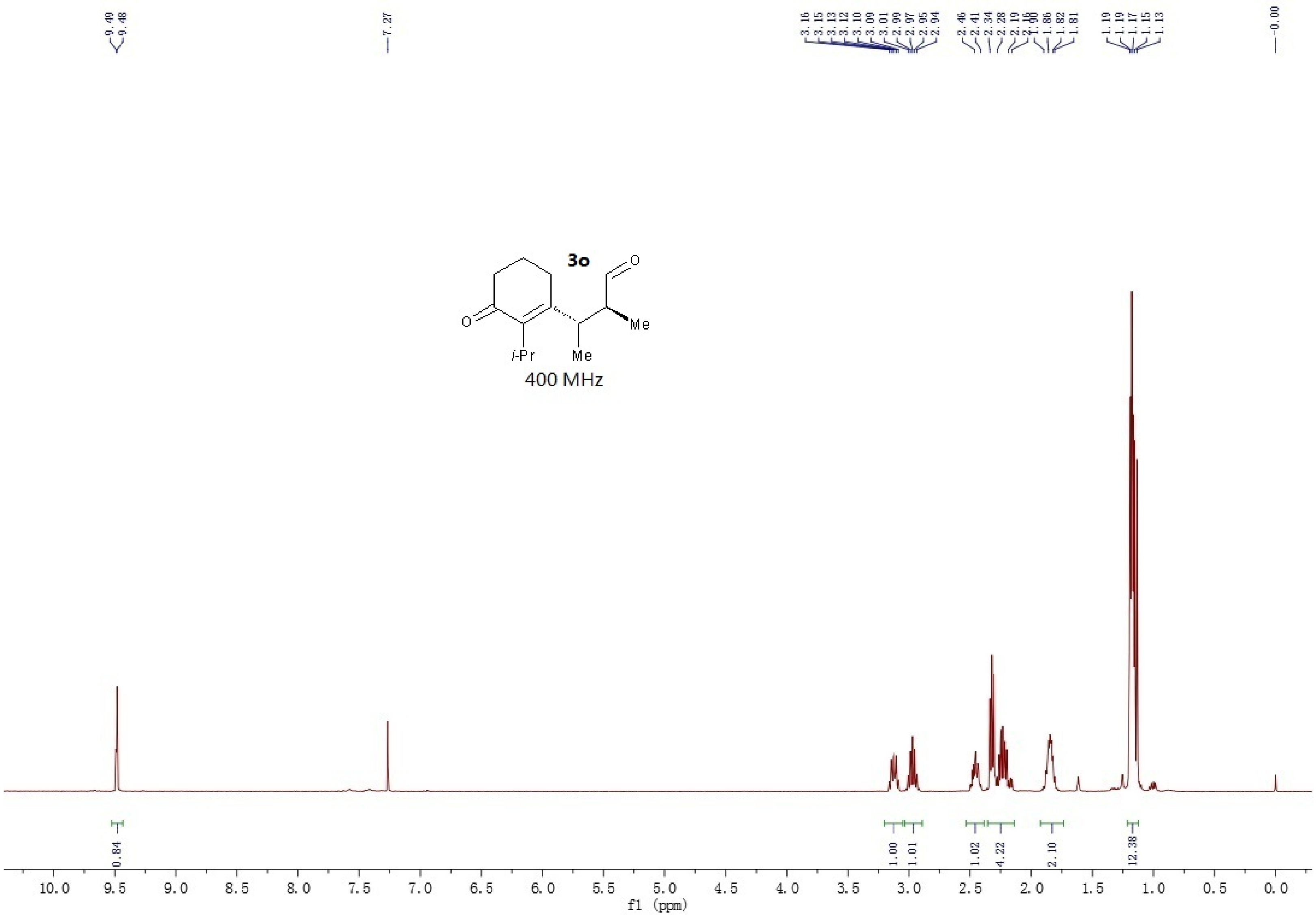
9.49
9.48
7.27
3.16
3.15
3.13
3.12
3.10
3.09
3.01
2.99
2.97
2.95
2.94
2.46
2.41
2.34
2.28
2.19
1.86
1.82
1.81
1.19
1.19
1.17
1.15
1.13
0.00
3o
O
O
Me
Me
i-Pr
400 MHz
0.84
1.00
1.01
1.02
4.22
2.10
12.38
10.0
9.5
9.0
8.5
8.0
7.5
7.0
6.5
6.0
5.5
5.0
4.5
4.0
3.5
3.0
2.5
2.0
1.5
1.0
0.5
0.0
f1 (ppm)

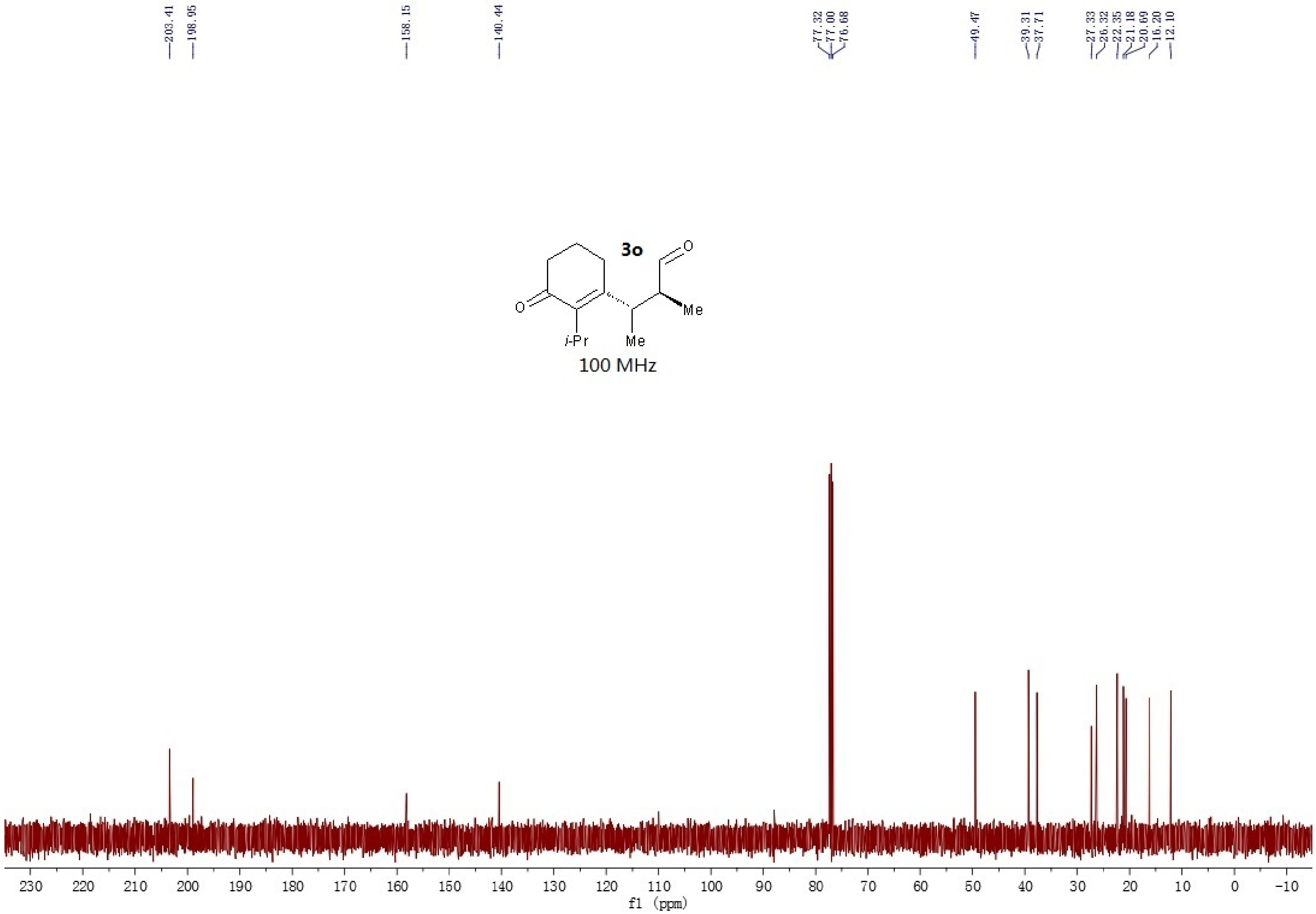

203.41
198.95
158.15
140.44
77.32
77.00
76.68
49.47
39.31
37.71
27.33
26.32
22.35
21.18
20.69
16.20
12.10
3o
O
O
Me
Me
i-Pr
100 MHz
230 220 210 200 190 180 170 160 150 140 130 120 110 100 90 80 70 60 50 40 30 20 10 0 -10
f1 (ppm)

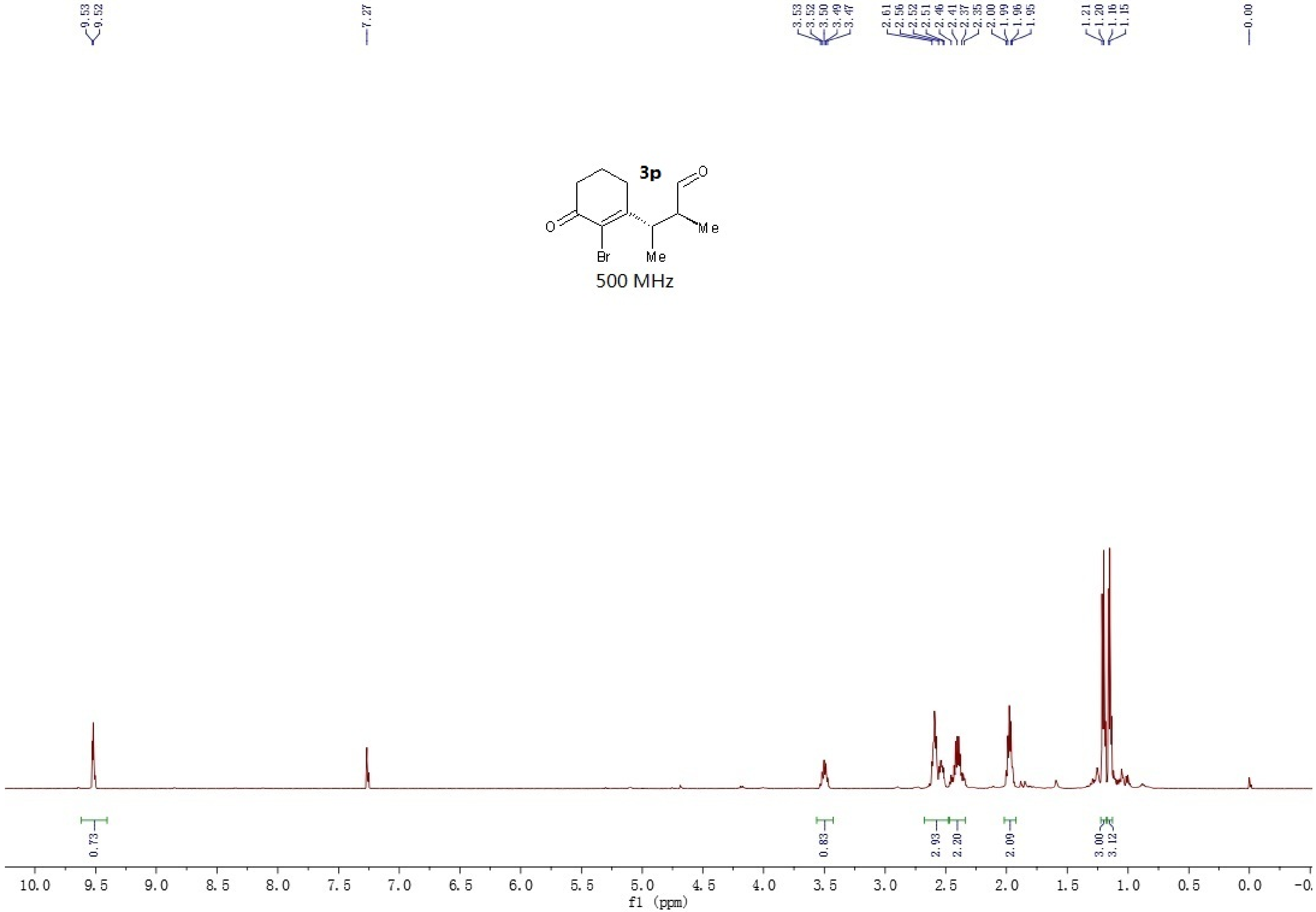
3p
O
Br
Me
Me
O
500 MHz
f1 (ppm)

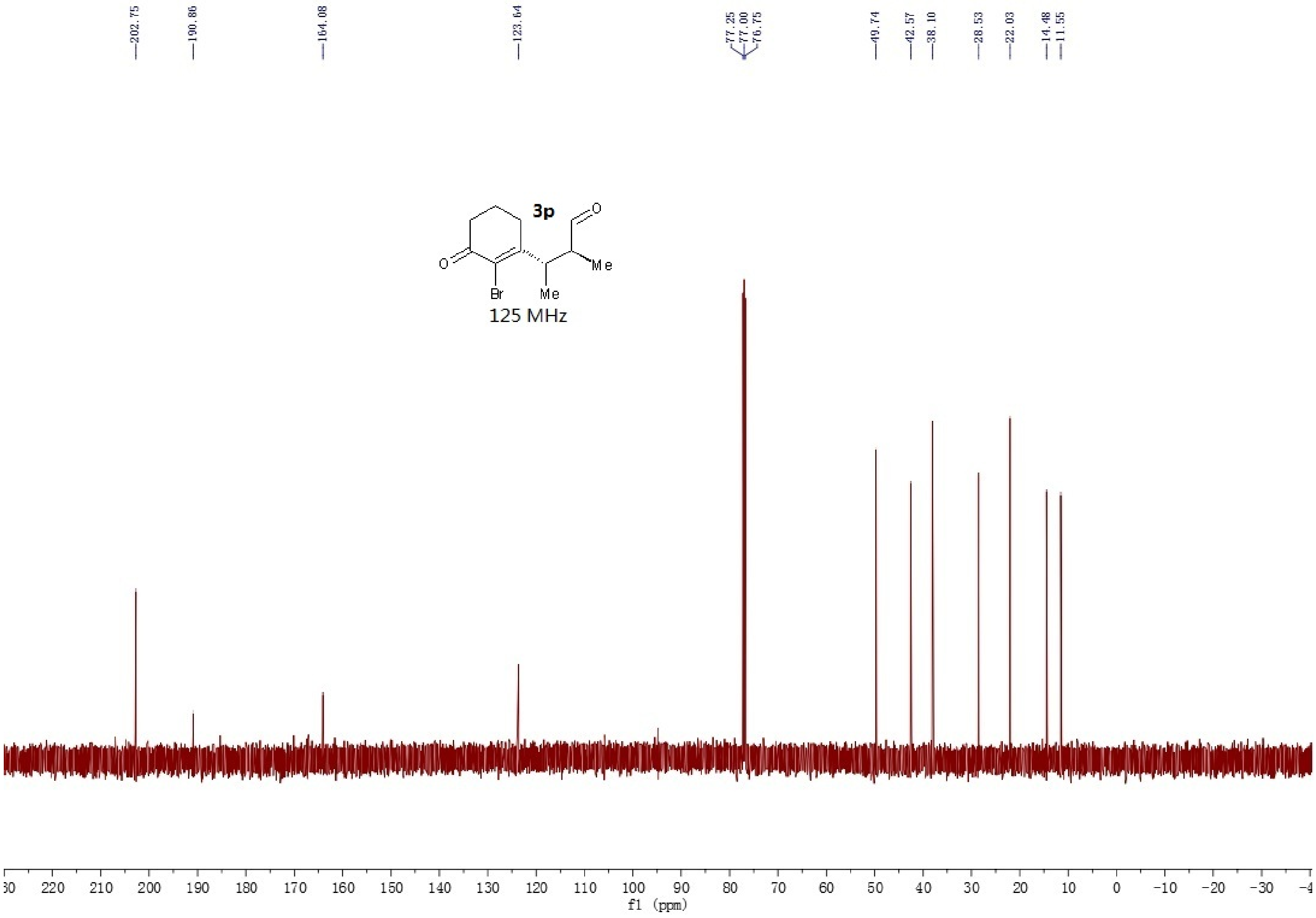
202.75
190.86
164.08
123.64
77.25
77.00
76.75
49.74
42.57
38.10
28.53
22.03
14.48
11.55
3p
O
O
Br
Me
Me
125 MHz
f1 (ppm)

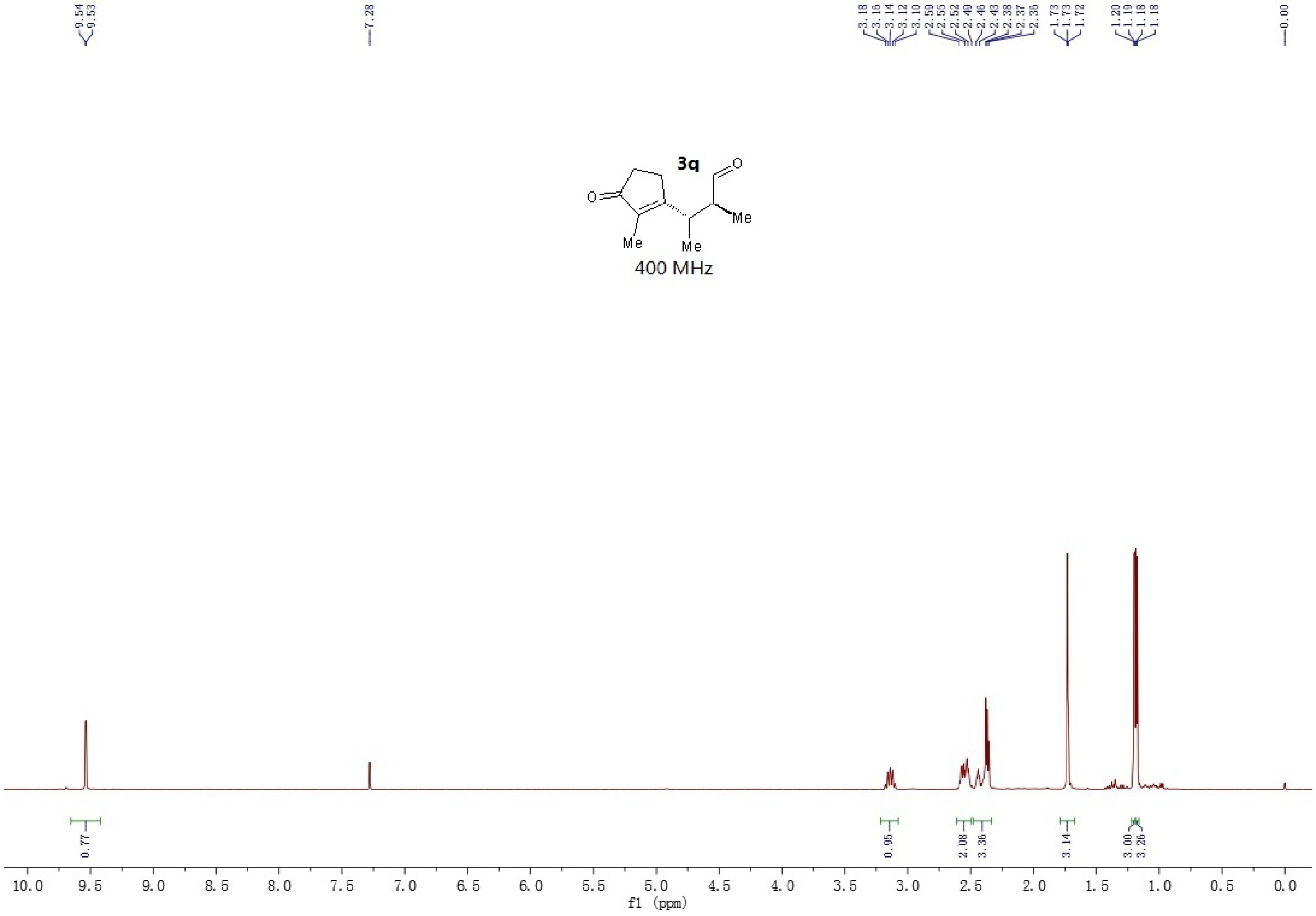
3q
400 MHz
10.0 9.5 9.0 8.5 8.0 7.5 7.0 6.5 6.0 5.5 5.0 4.5 4.0 3.5 3.0 2.5 2.0 1.5 1.0 0.5 0.0
f1 (ppm)

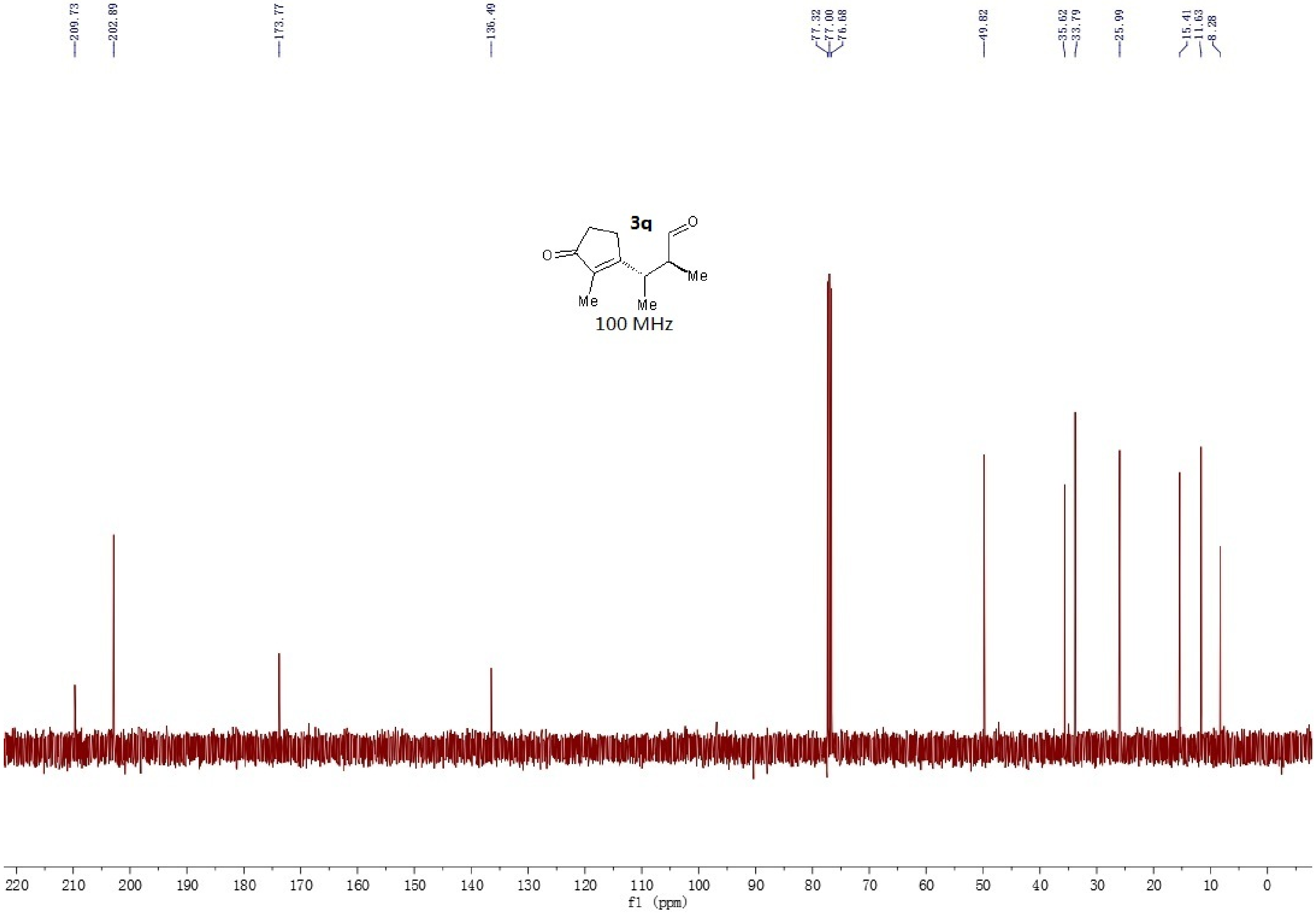
209.73
202.89
173.77
136.49
77.32
77.00
76.68
49.82
35.62
33.79
25.99
15.41
11.63
8.28
3q
O
O
Me
Me
Me
100 MHz
220 210 200 190 180 170 160 150 140 130 120 110 100 90 80 70 60 50 40 30 20 10 0
f1 (ppm)

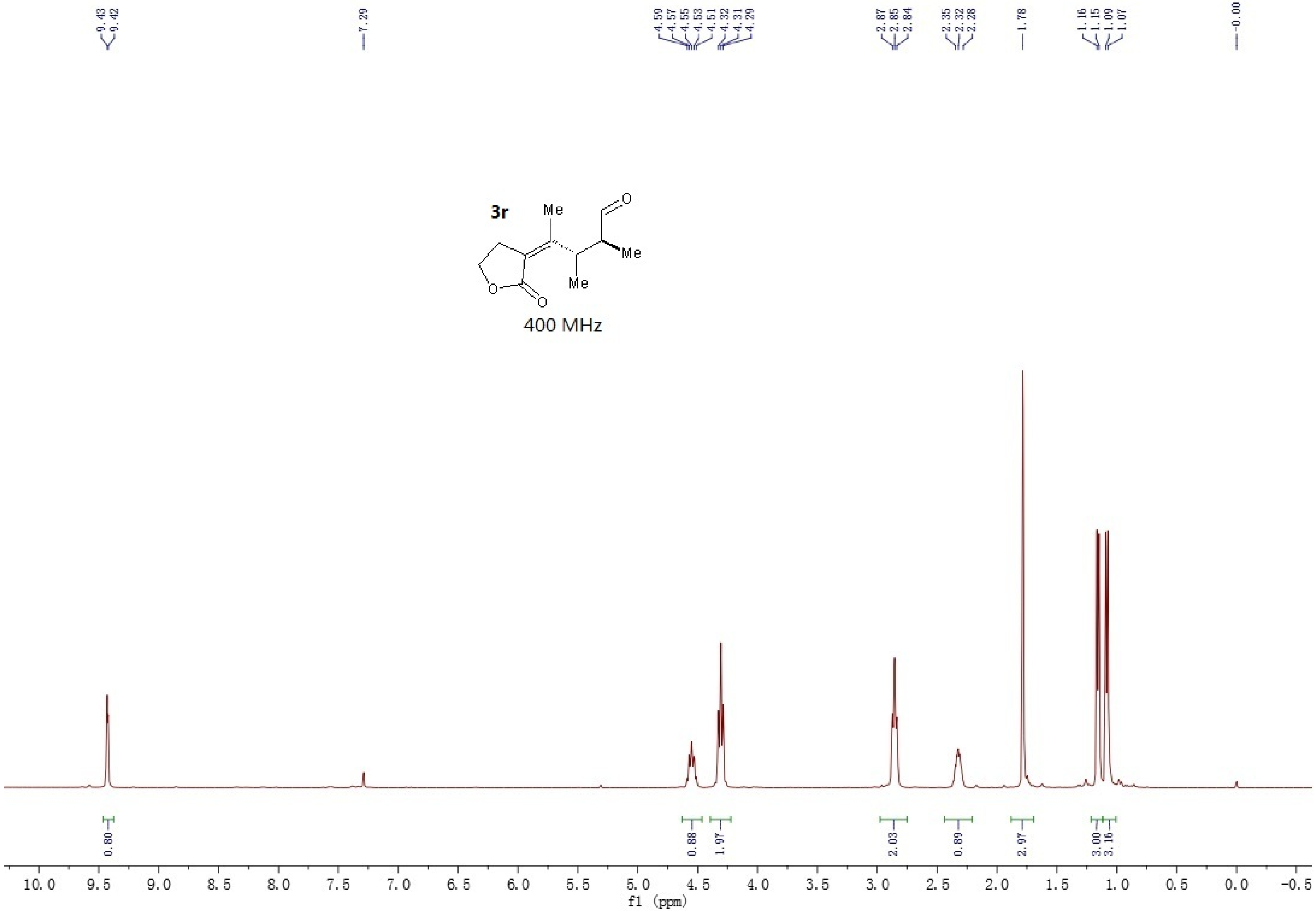
9.43
9.42
7.29
4.59
4.57
4.55
4.53
4.51
4.32
4.31
4.29
2.87
2.85
2.84
2.35
2.32
2.28
1.78
1.16
1.15
1.09
1.07
0.00
3r
Me
O
Me
O
O
Me
400 MHz
0.80
0.88
1.97
2.03
0.89
2.97
3.00
3.16
10.0 9.5 9.0 8.5 8.0 7.5 7.0 6.5 6.0 5.5 5.0 4.5 4.0 3.5 3.0 2.5 2.0 1.5 1.0 0.5 0.0 -0.5
f1 (ppm)

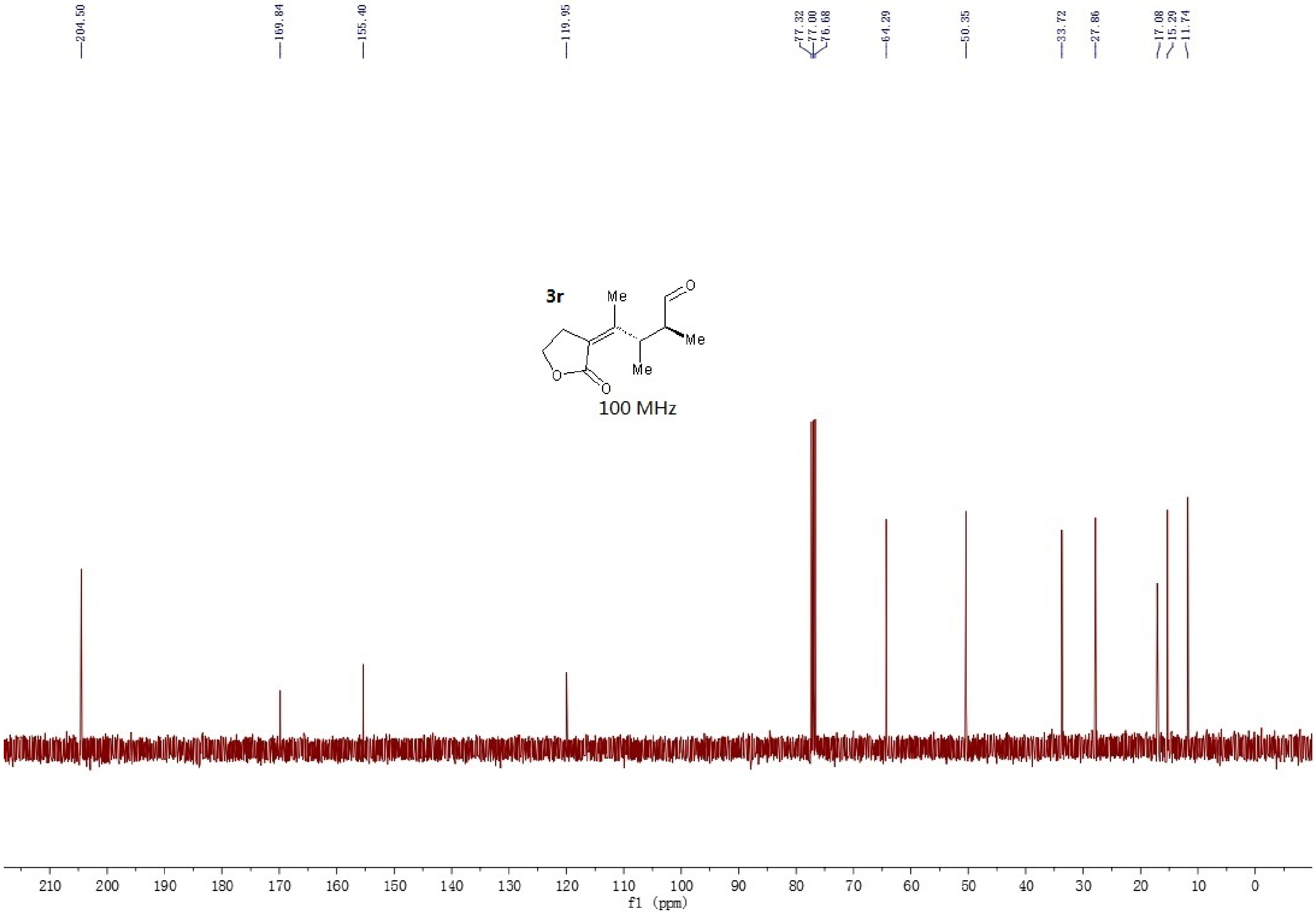
204.50
169.84
155.40
119.95
77.32
77.00
76.68
64.29
50.35
33.72
27.86
17.08
15.29
11.74
3r
Me
O
Me
Me
O
O
100 MHz
210
200
190
180
170
160
150
140
130
120
110
100
90
80
70
60
50
40
30
20
10
0
f1 (ppm)

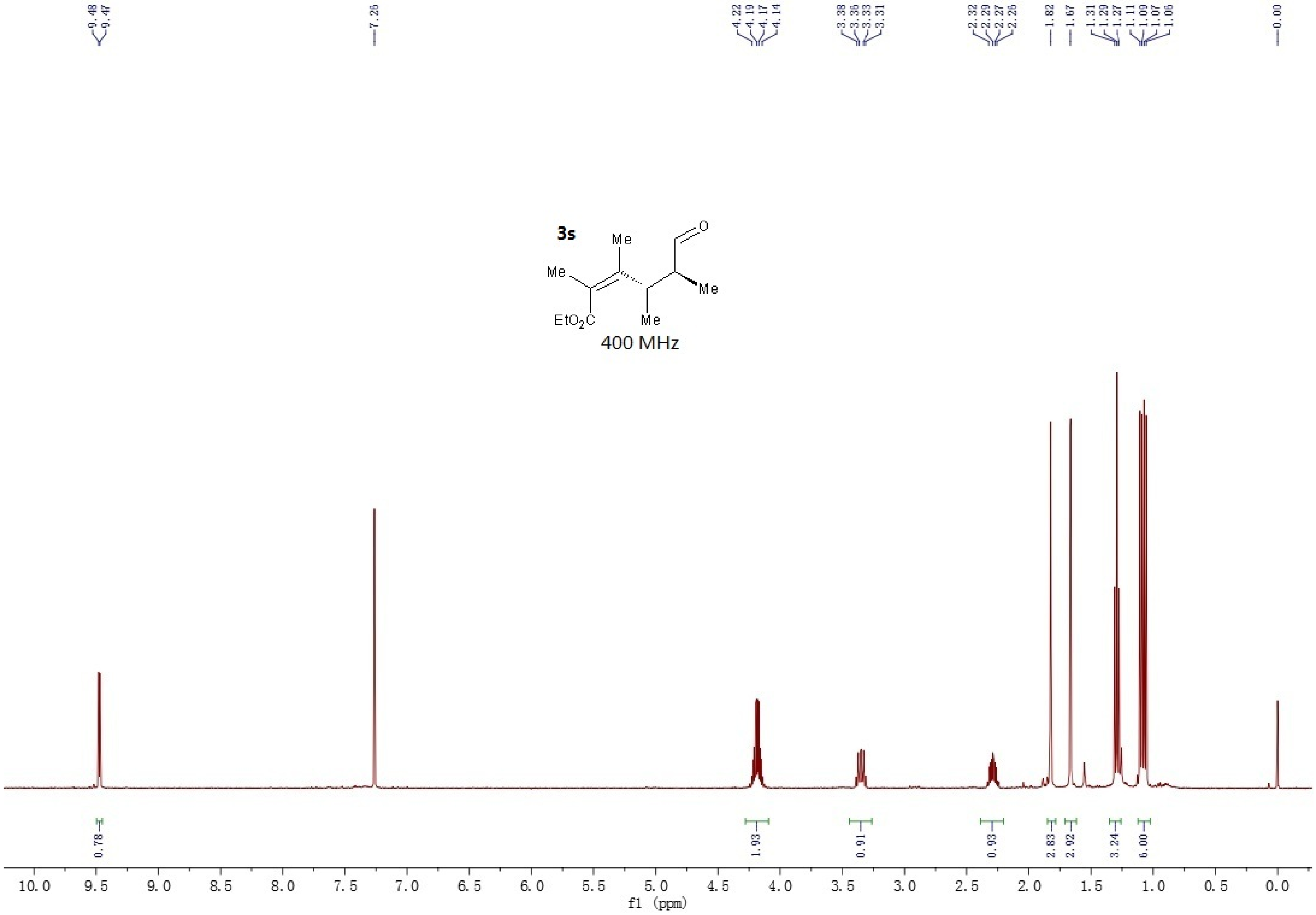
9.48
9.47
7.26
4.22
4.19
4.17
4.14
3.38
3.36
3.33
3.31
2.32
2.29
2.27
2.26
1.82
1.67
1.31
1.29
1.27
1.11
1.09
1.07
1.06
0.00
3s
Me
Me
Me
Me
O
EtO_2C
400 MHz
0.78
1.93
0.91
0.93
2.83
2.92
3.24
6.00
10.0
9.5
9.0
8.5
8.0
7.5
7.0
6.5
6.0
5.5
5.0
4.5
4.0
3.5
3.0
2.5
2.0
1.5
1.0
0.5
0.0
f1 (ppm)

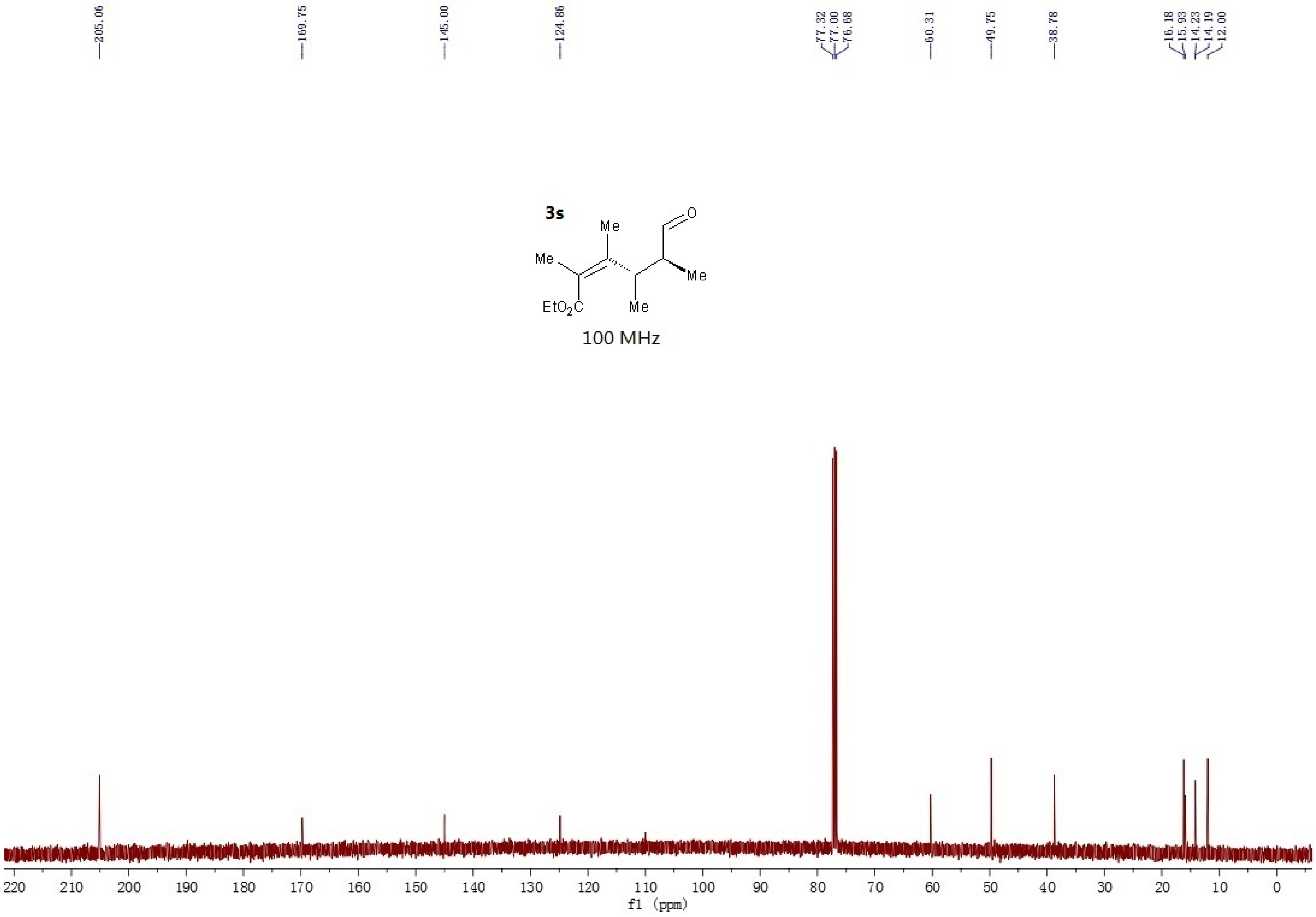

205.06
169.75
145.00
124.86
77.32
77.00
76.68
60.31
49.75
38.78
16.18
15.93
14.23
14.19
12.00
3s
Me
Me
Me
Me
EtO2C
O
100 MHz
220
210
200
190
180
170
160
150
140
130
120
110
100
90
80
70
60
50
40
30
20
10
0
f1 (ppm)

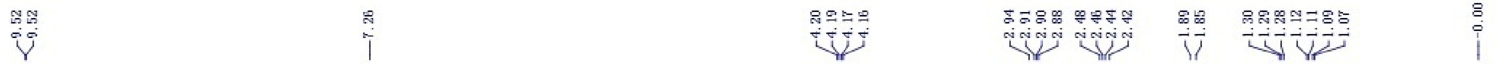

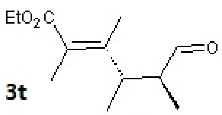

3t

500 MHz

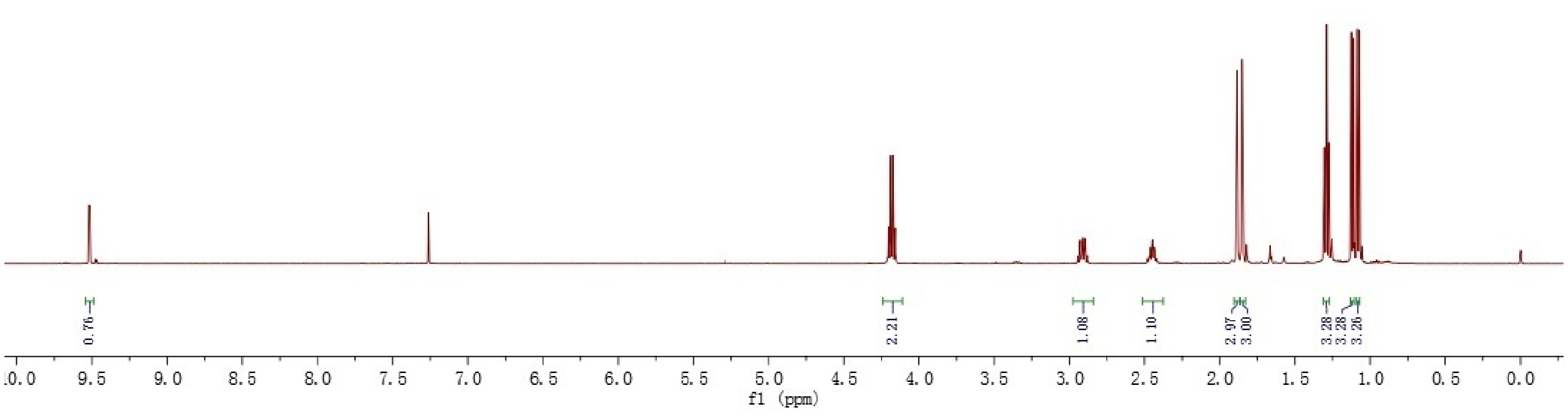

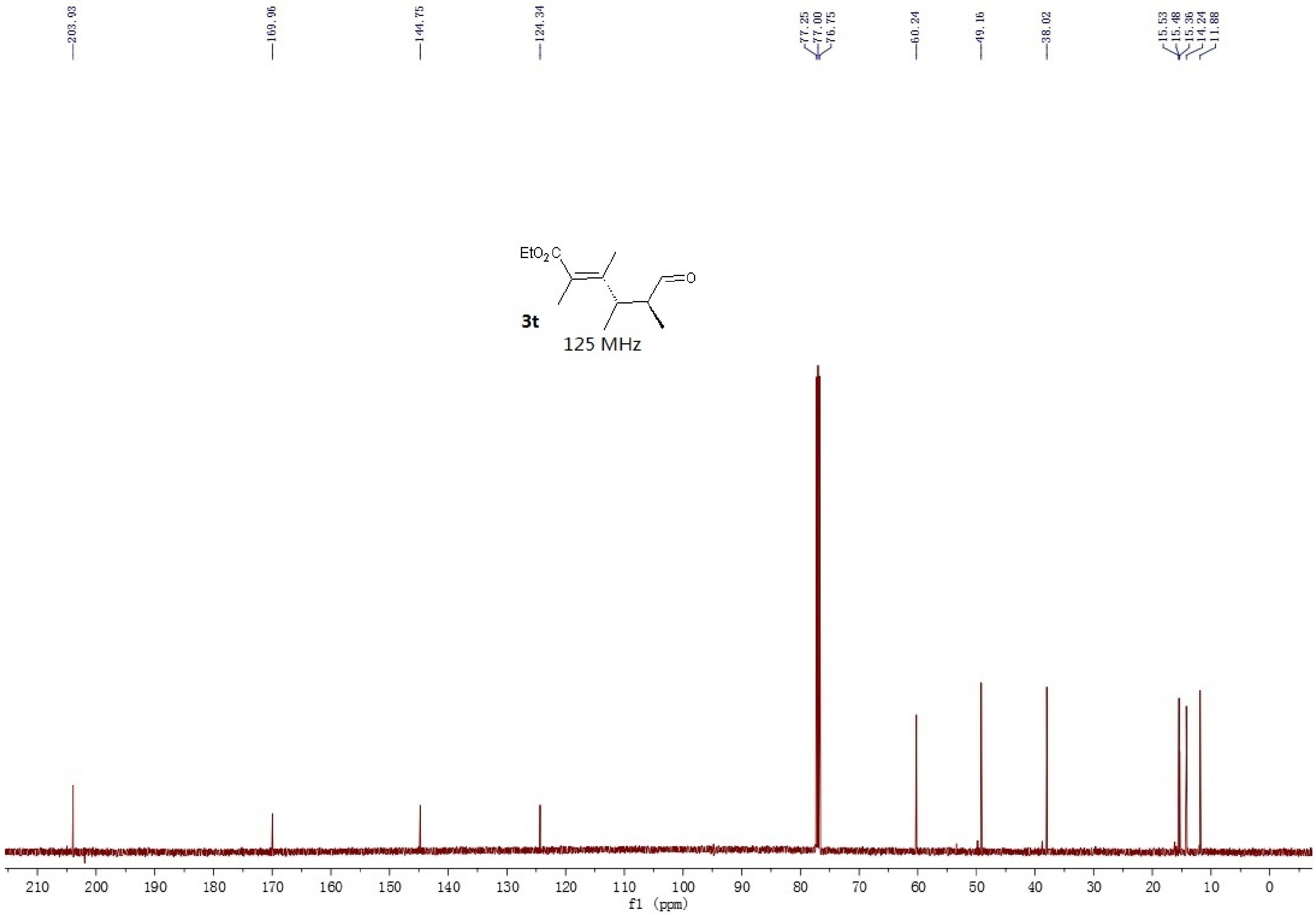

203.93
169.96
144.75
124.34
77.25
77.00
76.75
60.24
49.16
38.02
15.53
15.48
15.36
14.24
11.88
EtO2C
O
3t
125 MHz
210
200
190
180
170
160
150
140
130
120
110
100
90
80
70
60
50
40
30
20
10
0
f1 (ppm)

3t'

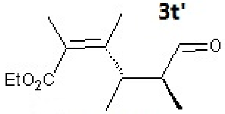
EtO2C
O

500 MHz

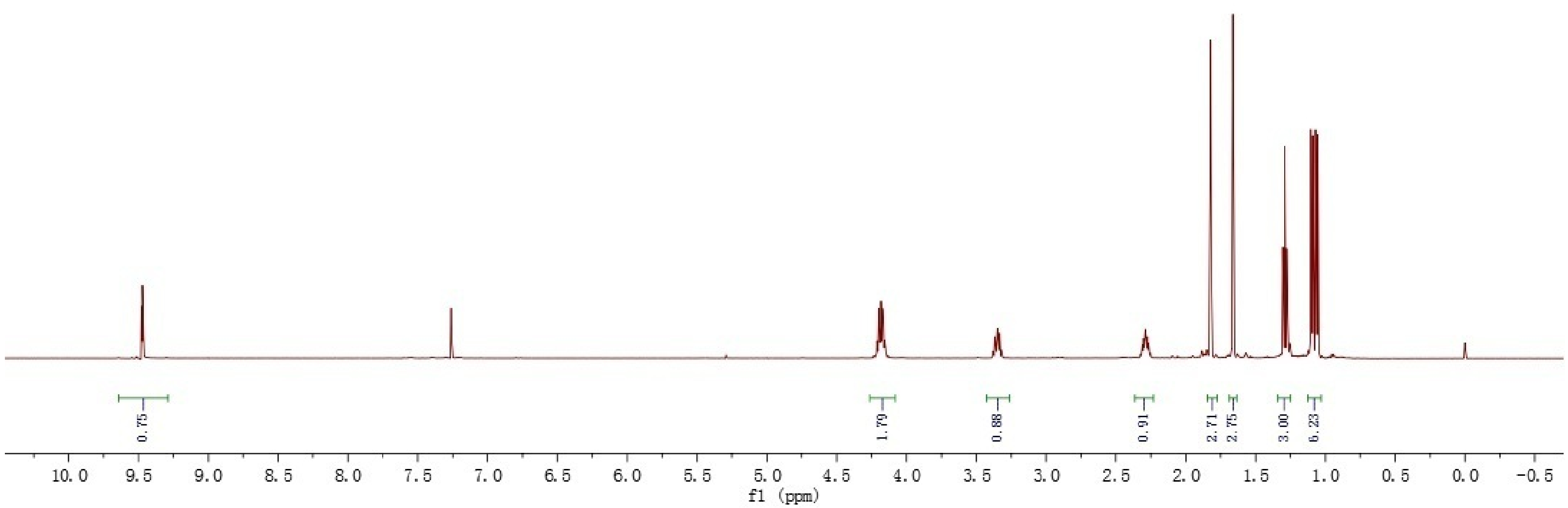
9.48
9.47
7.26
4.22
4.20
4.17
4.13
3.38
3.37
3.35
3.32
2.33
2.29
2.27
2.25
1.83
1.66
1.31
1.29
1.28
1.10
1.09
1.07
1.06
0.00
0.75
1.79
0.88
0.91
2.71
2.75
3.00
6.23
10.0
9.5
9.0
8.5
8.0
7.5
7.0
6.5
6.0
5.5
5.0
4.5
4.0
3.5
3.0
2.5
2.0
1.5
1.0
0.5
0.0
-0.5
f1 (ppm)

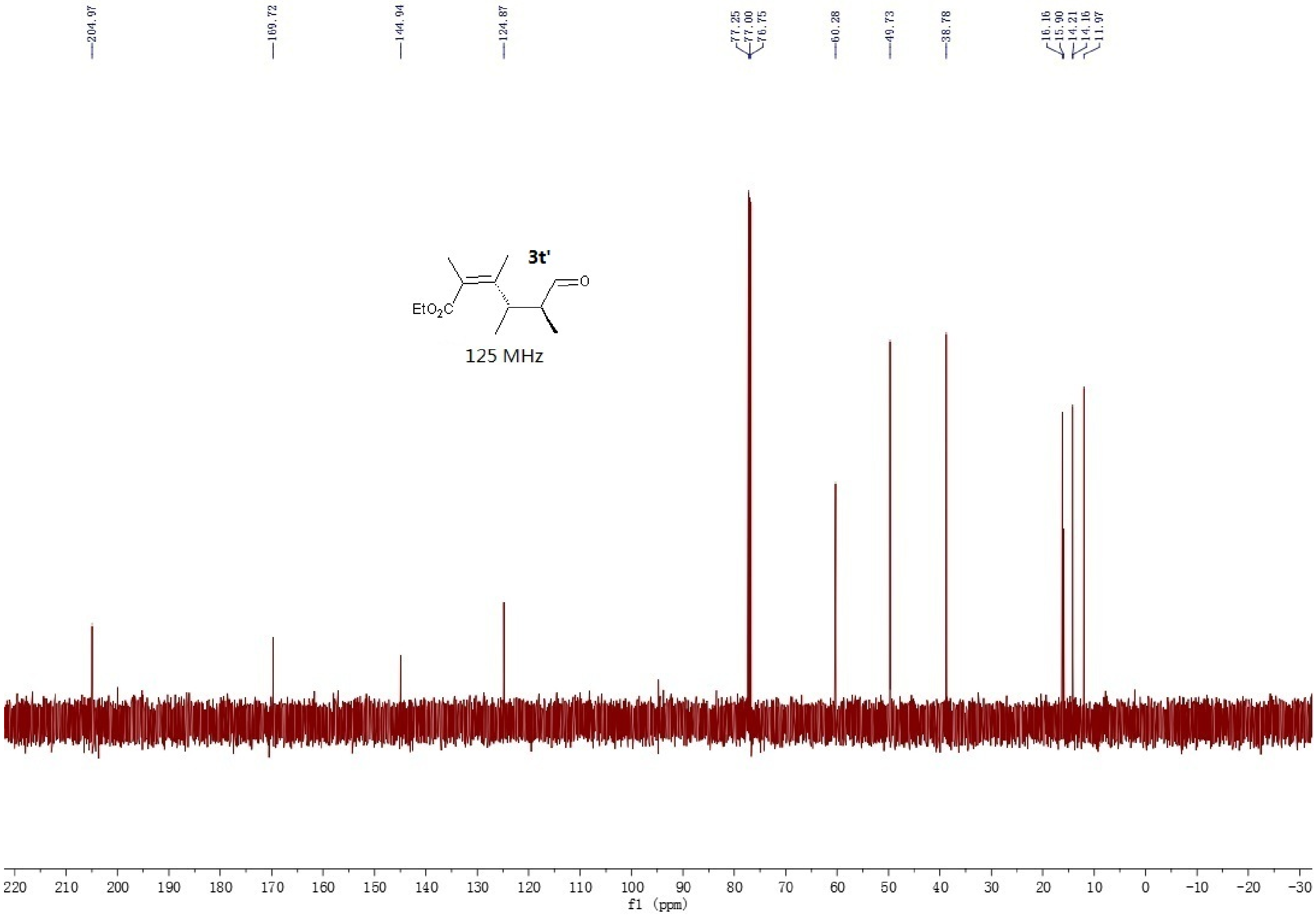

204.97
169.72
144.94
124.87
77.25
77.00
76.75
60.28
49.73
38.78
16.16
15.90
14.21
14.16
11.97
3t'
EtO_2C
O
125 MHz
220 210 200 190 180 170 160 150 140 130 120 110 100 90 80 70 60 50 40 30 20 10 0 −10 −20 −30
f1 (ppm)

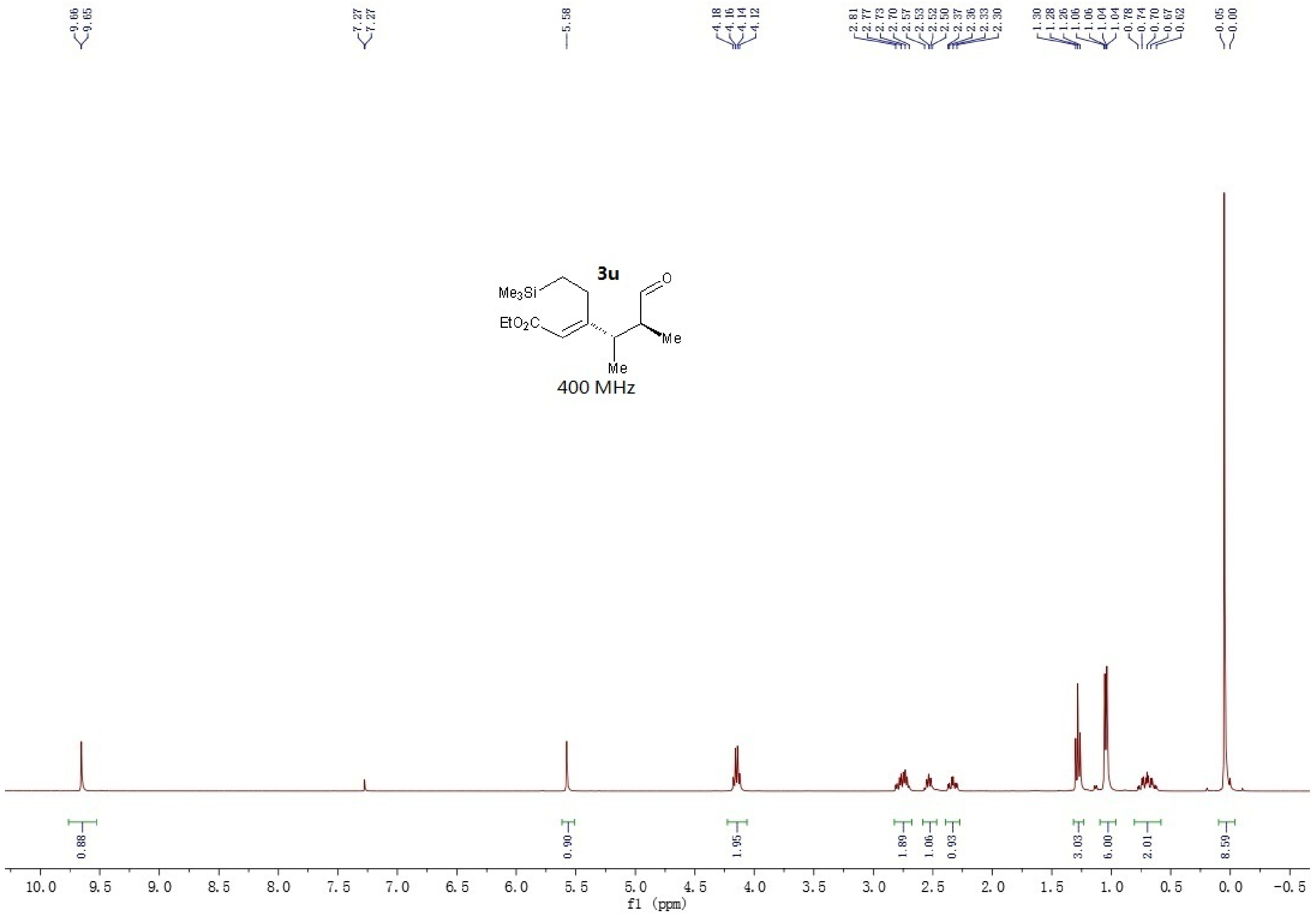
3u
Me3Si
EtO2C
O
Me
Me
400 MHz
f1 (ppm)

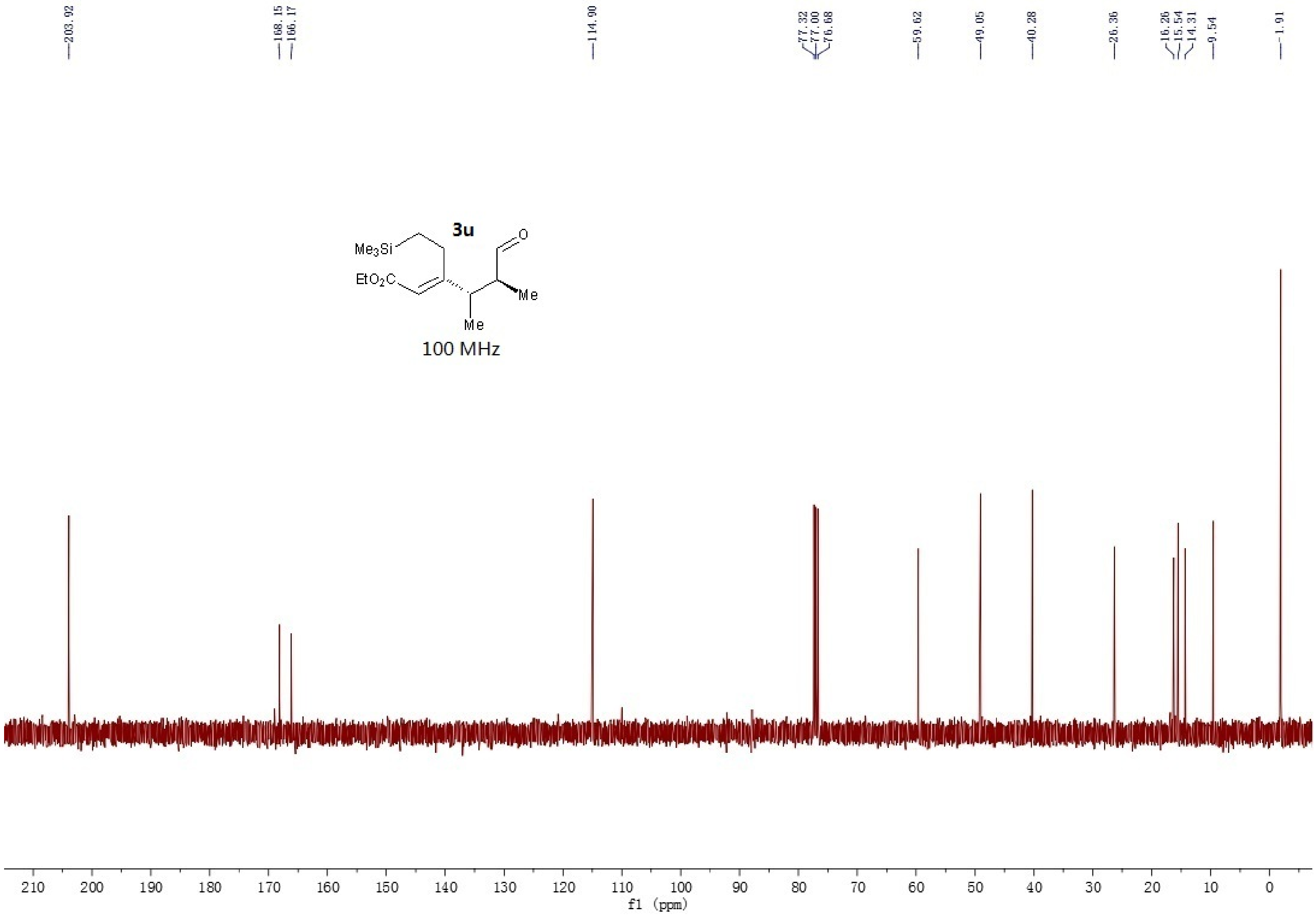

203.92
168.15
166.17
114.90
77.32
77.00
76.68
59.62
49.05
40.28
26.36
16.26
15.54
14.31
9.54
-1.91
3u
Me3Si
EtO2C
Me
Me
O
100 MHz
210 200 190 180 170 160 150 140 130 120 110 100 90 80 70 60 50 40 30 20 10 0
f1 (ppm)

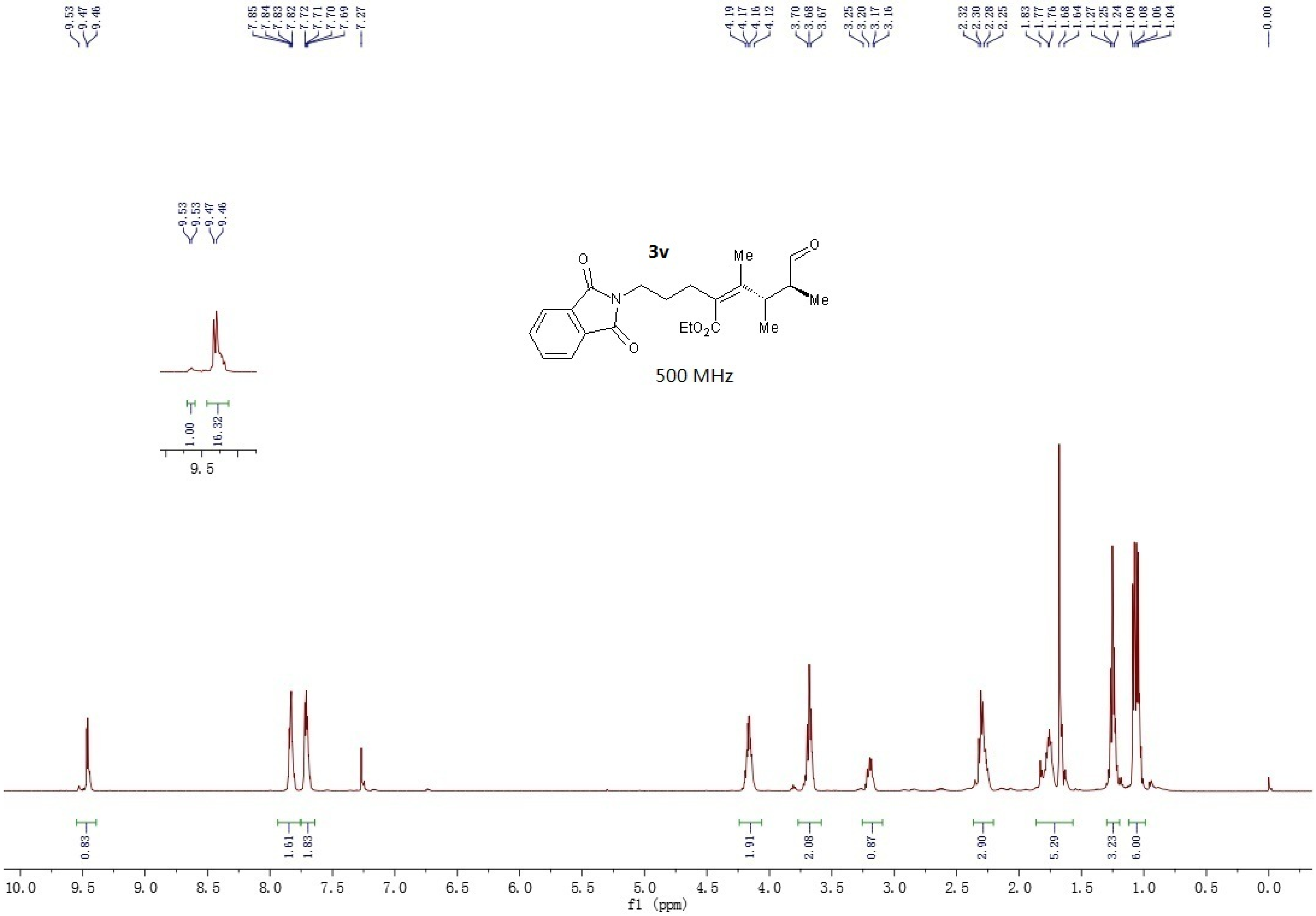
3v
Me
O
O
N
O
Me
Me
EtO2C
500 MHz
f1 (ppm)

3v

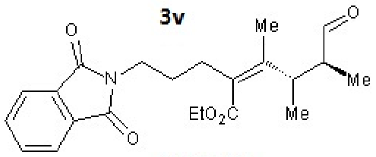
O
N
O
Me
O
Me
Me
EtO$_2$C

125 MHz

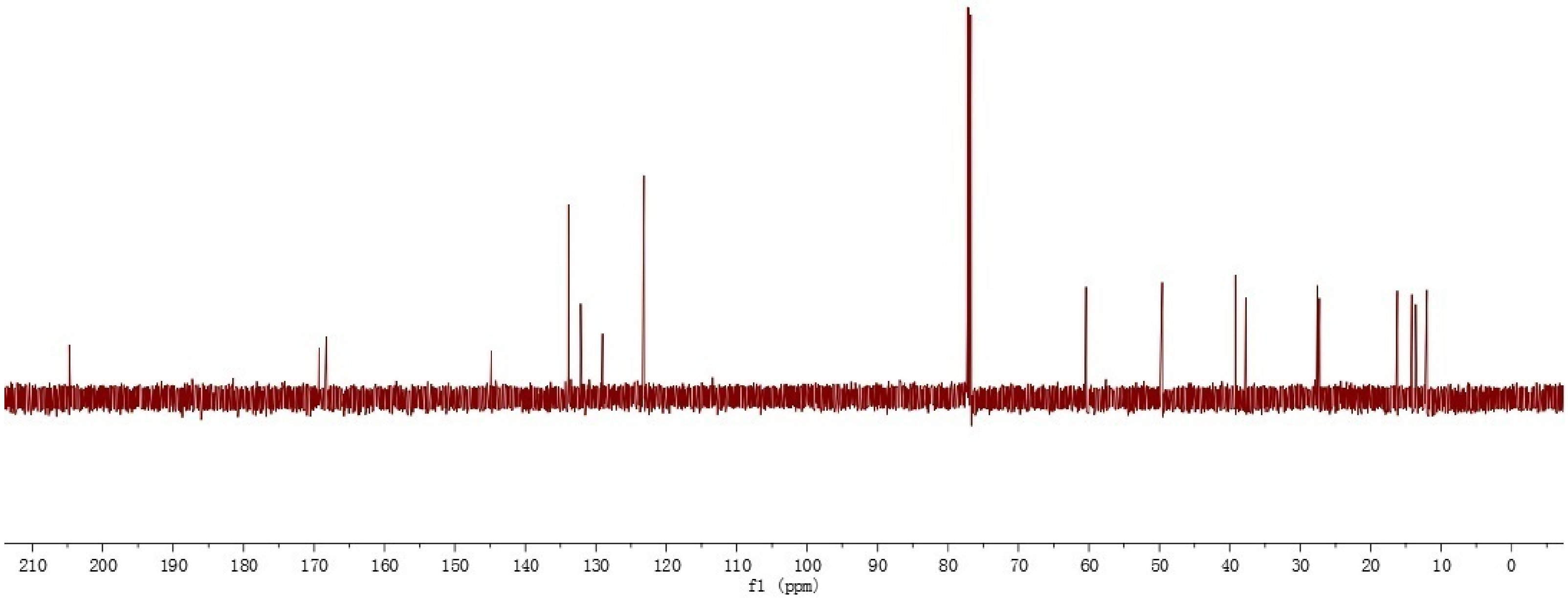
204.74
169.28
168.25
144.82
133.87
132.11
129.02
123.15
77.25
77.00
76.75
60.40
49.62
39.18
37.73
27.51
27.24
16.23
14.15
13.61
11.99
210 200 190 180 170 160 150 140 130 120 110 100 90 80 70 60 50 40 30 20 10 0
f1 (ppm)

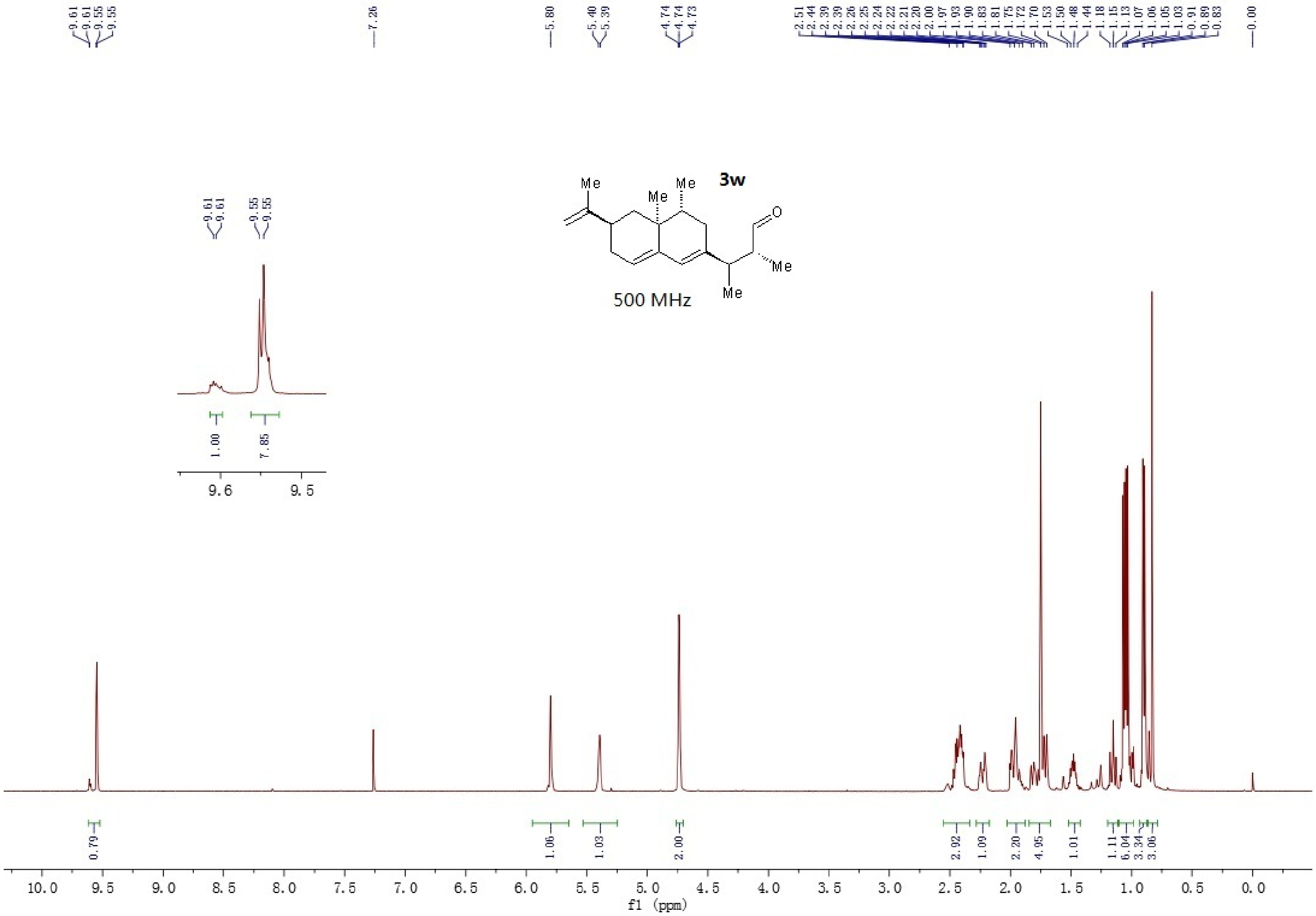
3w
500 MHz
f1 (ppm)

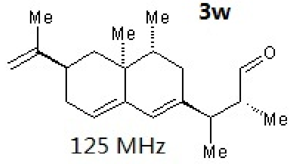
3w
125 MHz

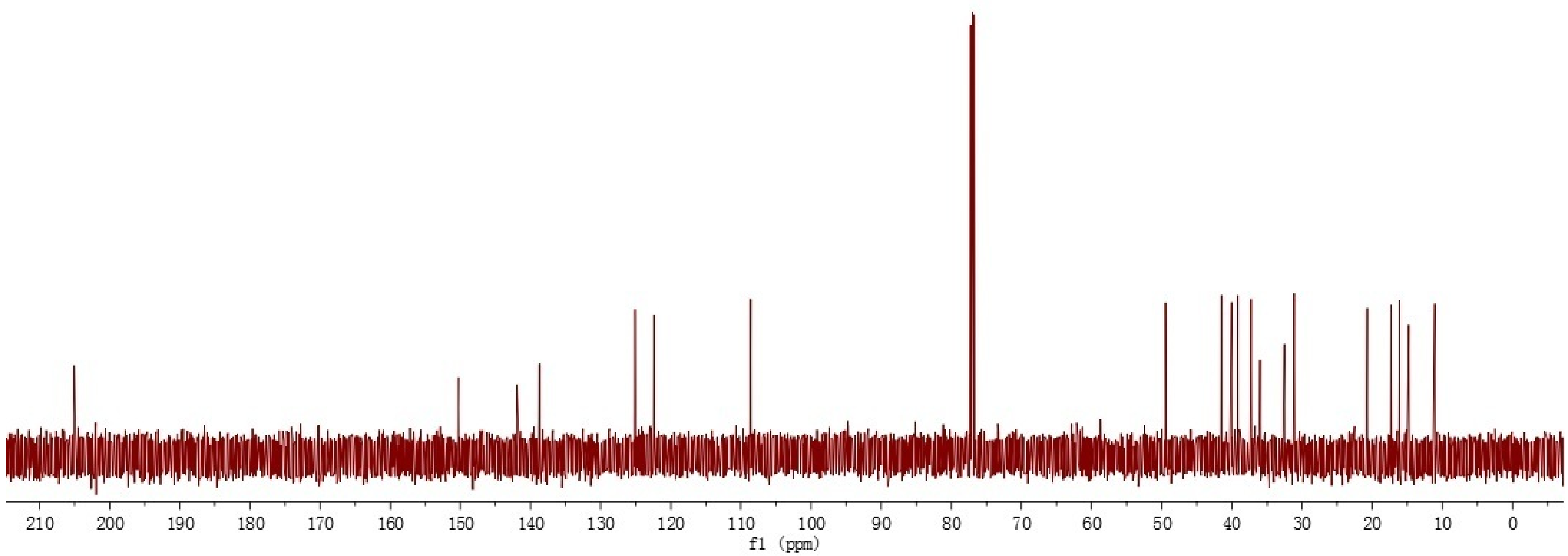
205.06
150.26
141.95
138.74
125.11
122.38
108.60
77.25
77.00
76.75
49.49
41.48
40.07
39.14
37.27
36.01
32.51
31.14
20.69
17.34
16.08
14.79
11.06
f1 (ppm)

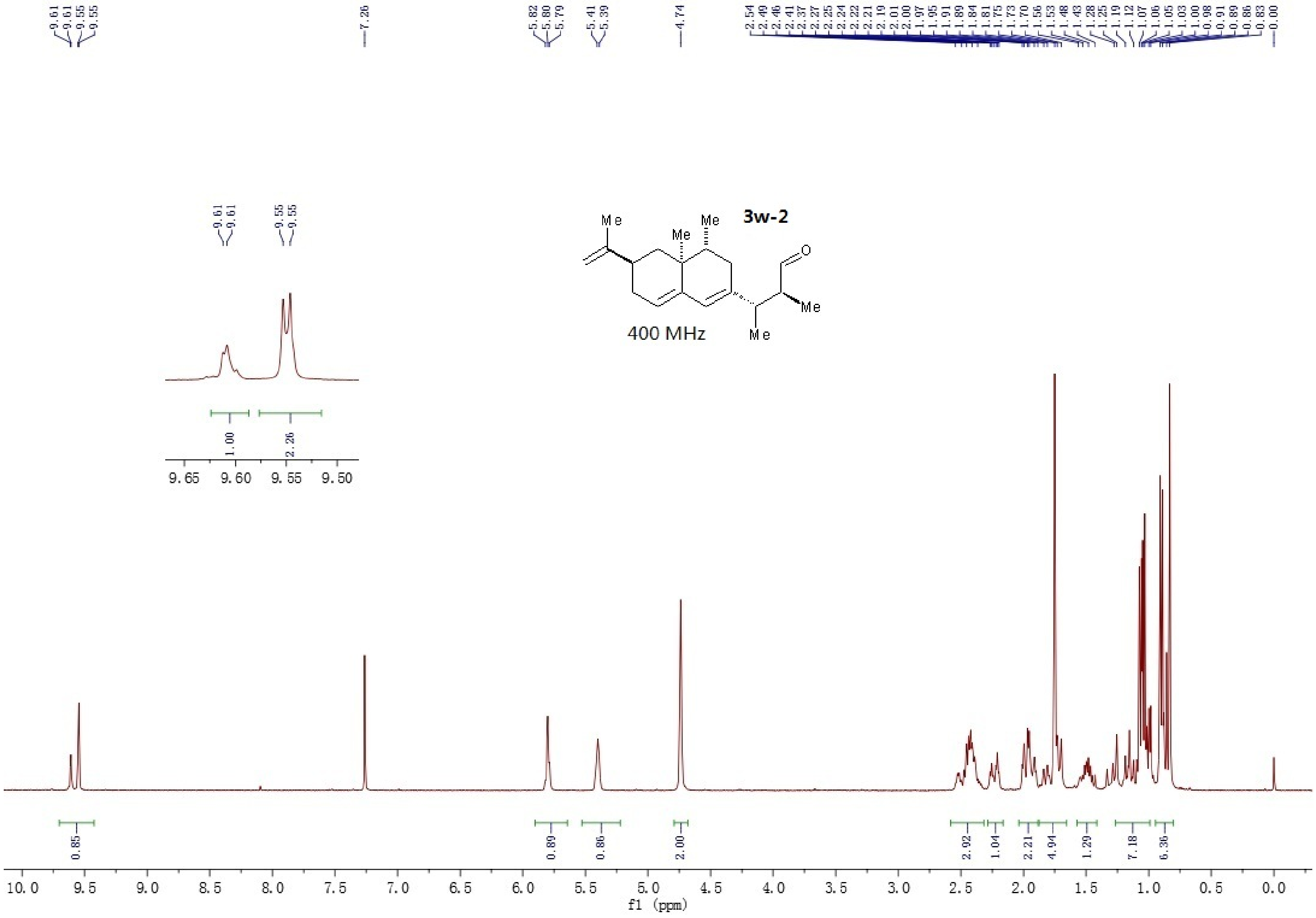
3w-2
400 MHz
Me
Me
Me
Me
Me
O
f1 (ppm)

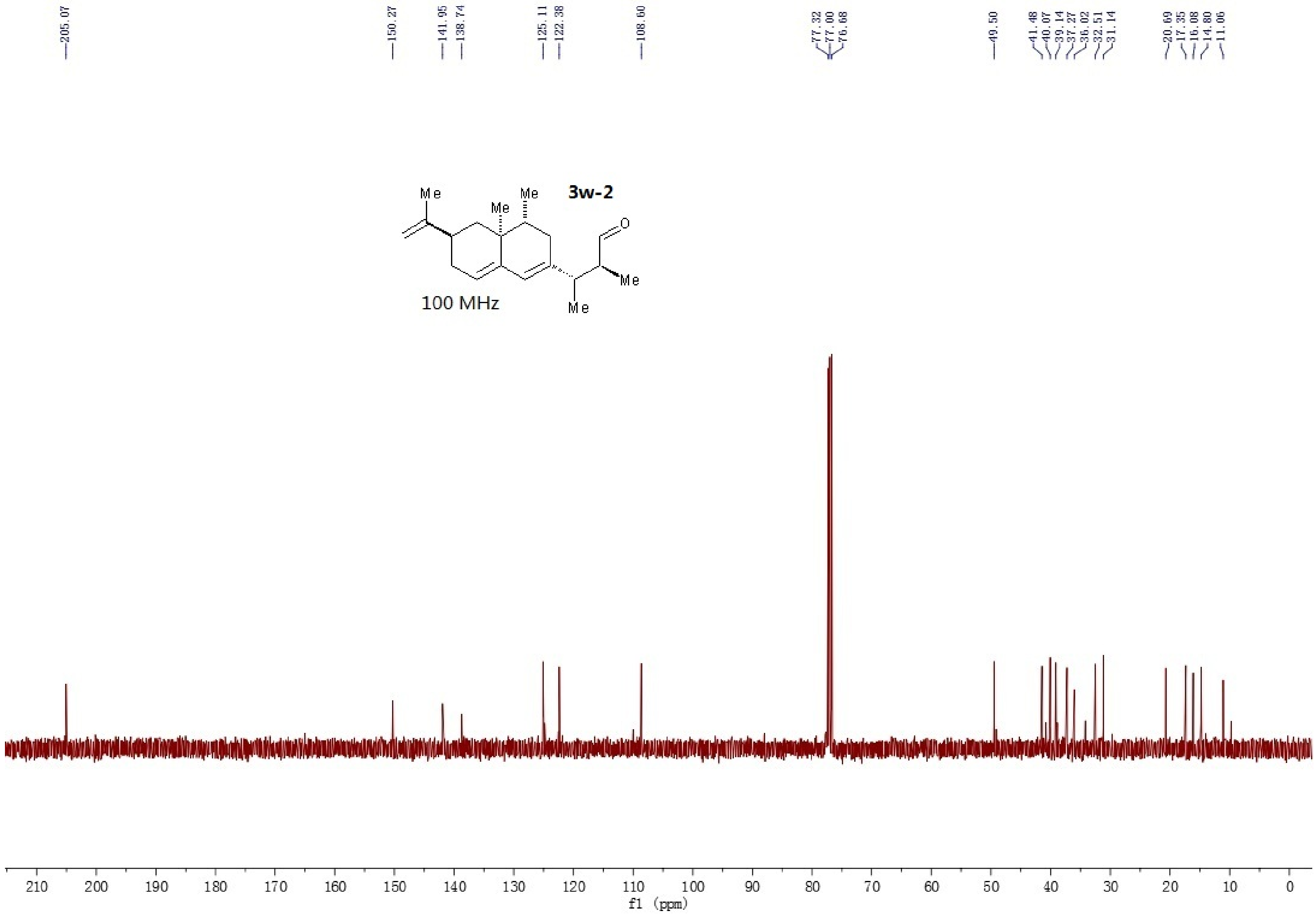
205.07
150.27
141.95
138.74
125.11
122.38
108.60
77.32
77.00
76.68
49.50
41.48
40.07
39.14
37.27
36.02
32.51
31.14
20.69
17.35
16.08
14.80
11.06
3w-2
100 MHz
Me
O
210 200 190 180 170 160 150 140 130 120 110 100 90 80 70 60 50 40 30 20 10 0
f1 (ppm)

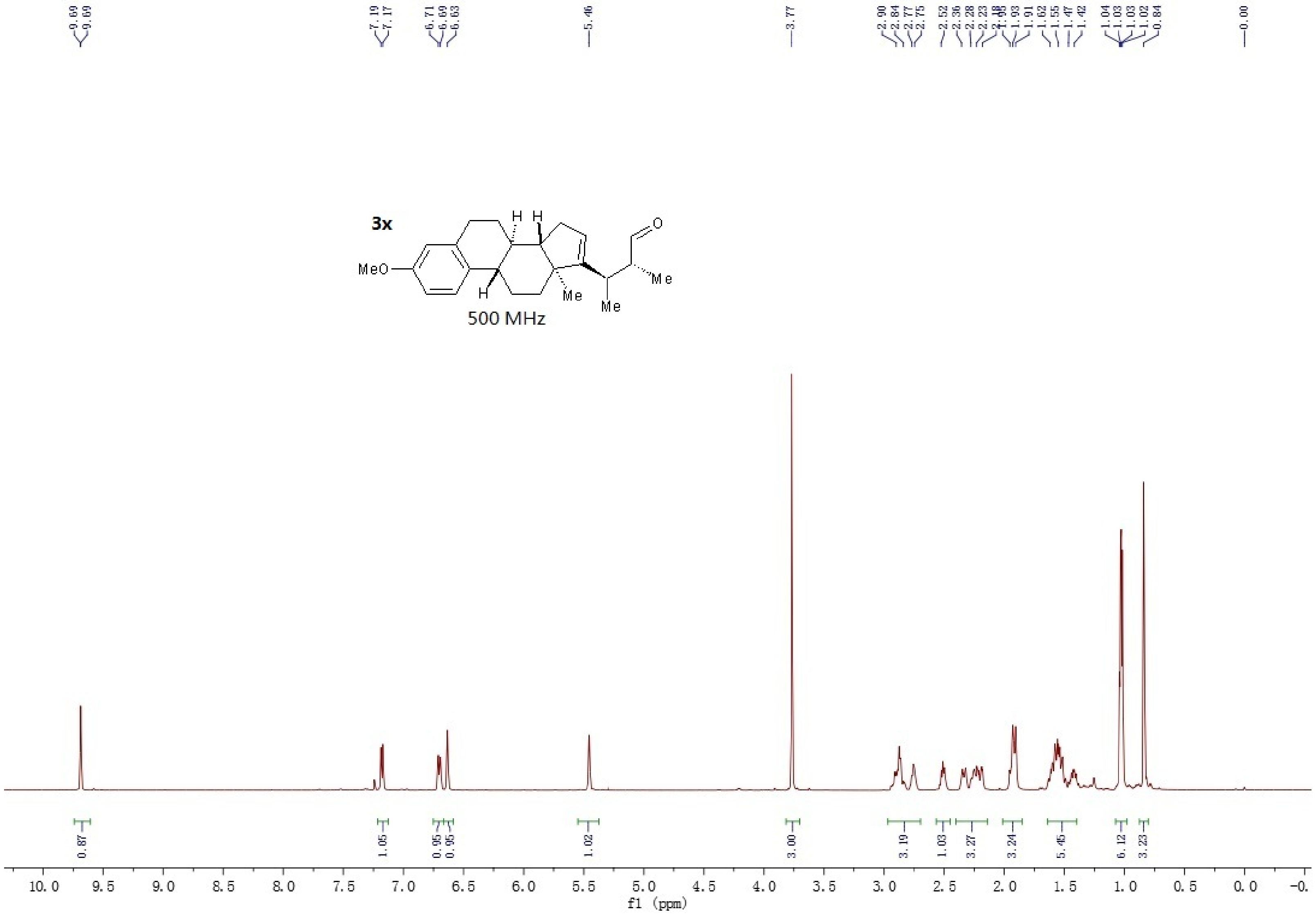
3x
MeO
Me
500 MHz
f1 (ppm)

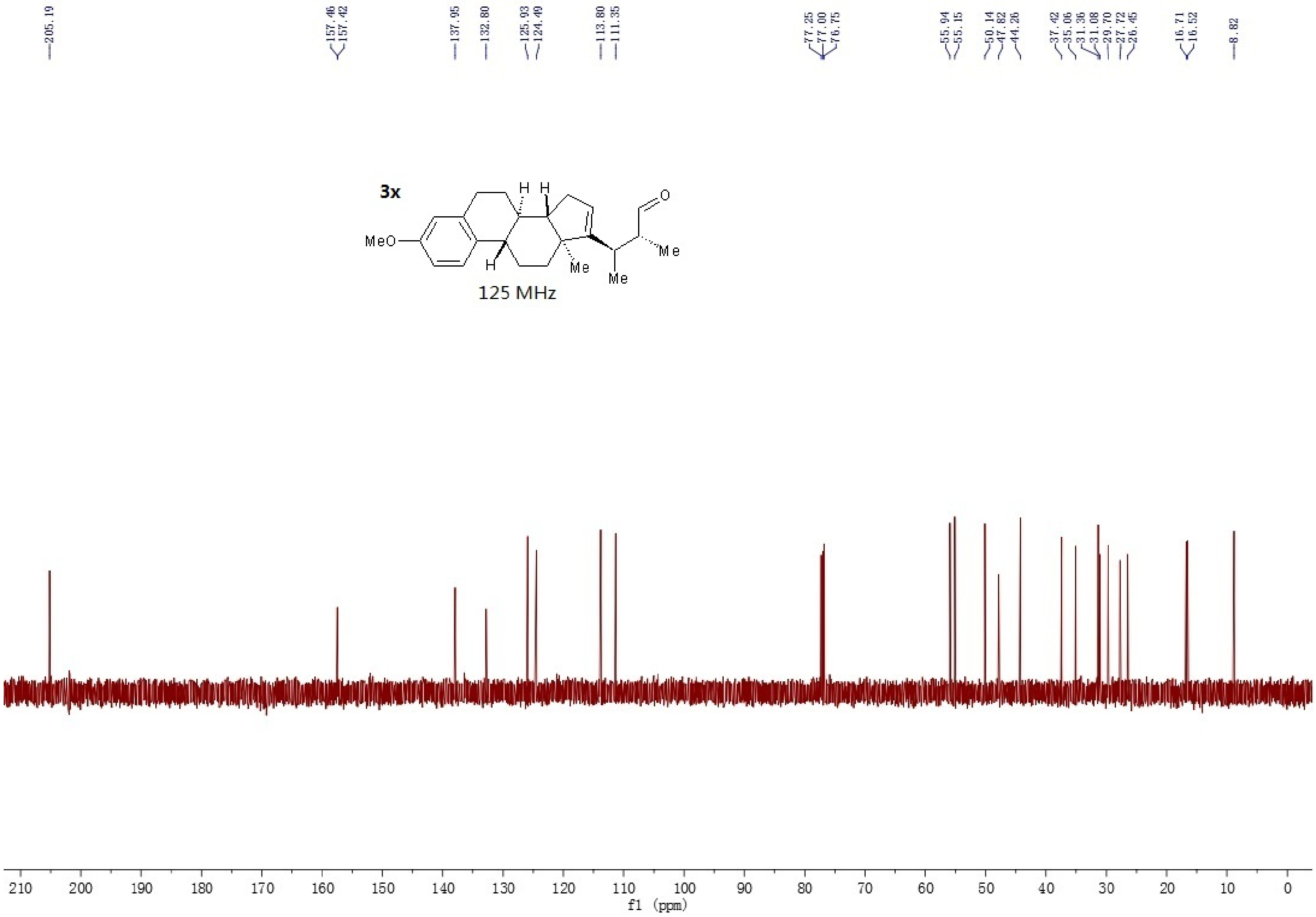
205.19
157.46
157.42
137.95
132.80
125.93
124.49
113.80
111.35
77.25
77.00
76.75
55.94
55.15
50.14
47.82
44.26
37.42
35.06
31.36
31.08
29.70
27.72
26.45
16.71
16.52
8.82
3x
MeO
H
H
H
Me
Me
Me
O
125 MHz
210 200 190 180 170 160 150 140 130 120 110 100 90 80 70 60 50 40 30 20 10 0
f1 (ppm)

3x-2

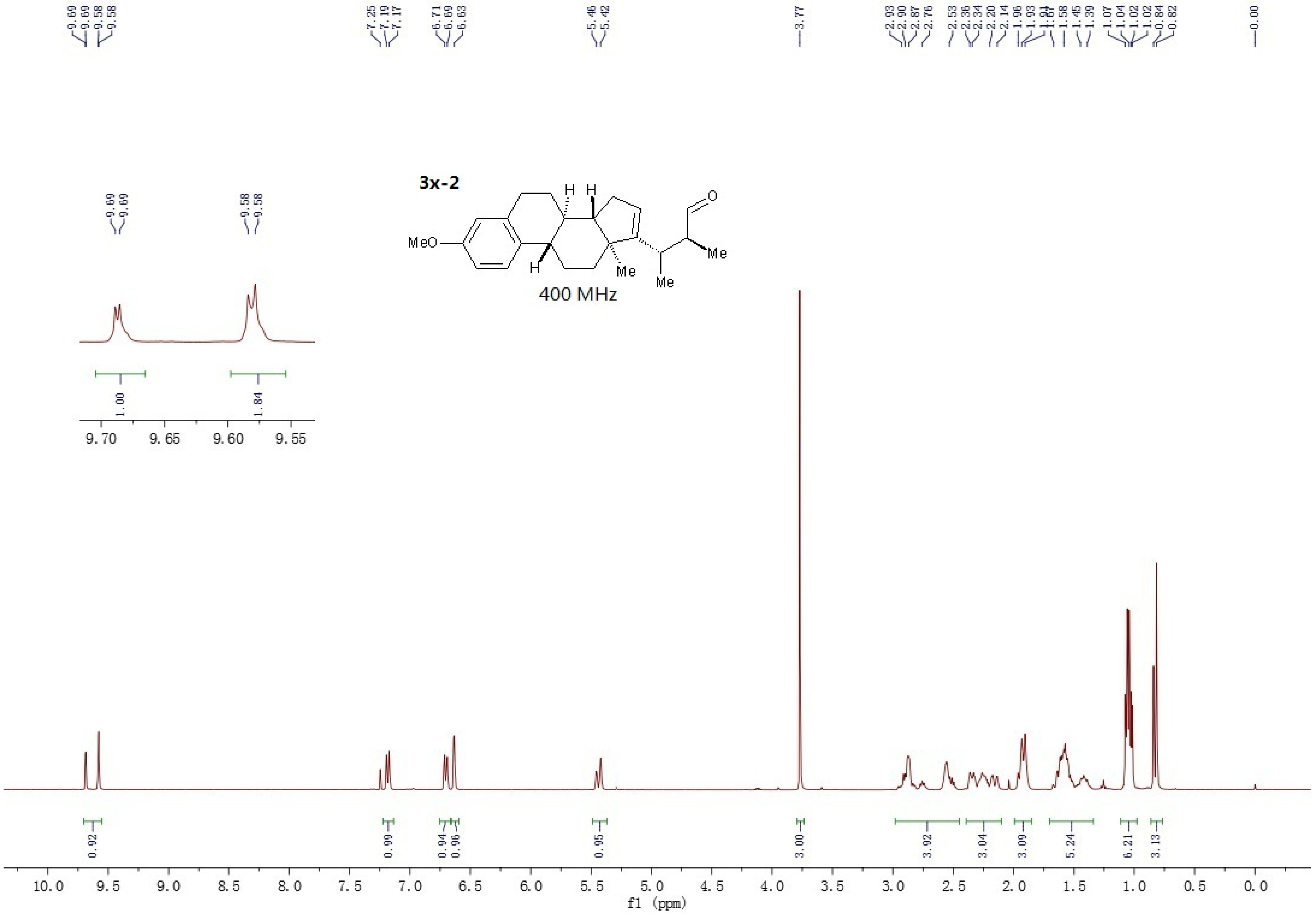
MeO
H
H
H
Me
Me
O
Me
400 MHz
9.69
9.69
9.58
9.58
1.00
1.84
9.70
9.65
9.60
9.55
7.25
7.19
7.17
6.71
6.69
6.63
5.46
5.42
3.77
2.93
2.90
2.87
2.76
2.53
2.36
2.34
2.20
2.14
1.96
1.93
1.58
1.45
1.39
1.07
1.04
1.02
1.02
0.84
0.82
0.00
0.92
0.99
0.94
0.96
0.95
3.00
3.92
3.04
3.09
5.24
6.21
3.13
10.0
9.5
9.0
8.5
8.0
7.5
7.0
6.5
6.0
5.5
5.0
4.5
4.0
3.5
3.0
2.5
2.0
1.5
1.0
0.5
0.0
f1 (ppm)

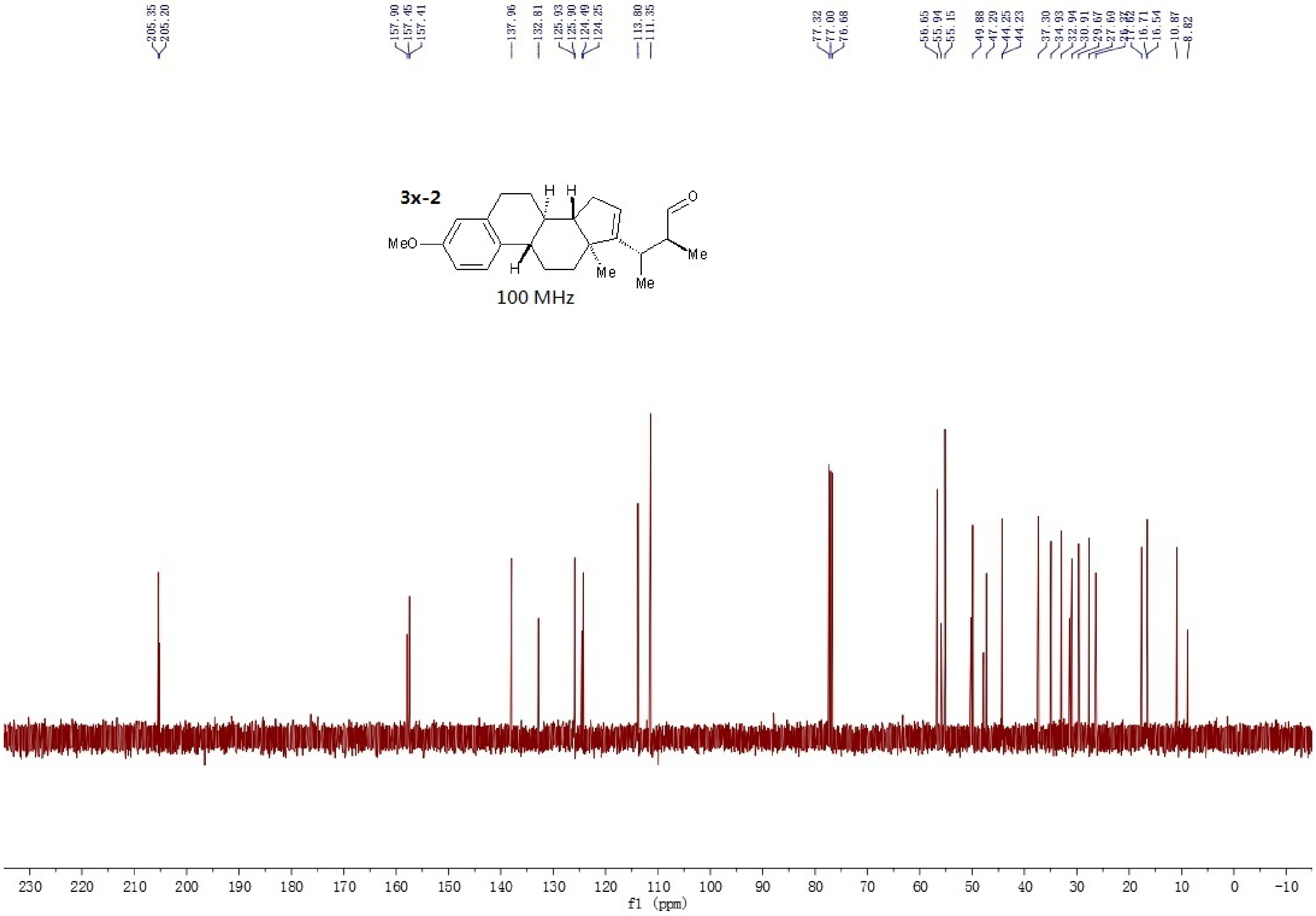

3x-2
MeO
H
H
H
Me
Me
Me
O
100 MHz
f1 (ppm)

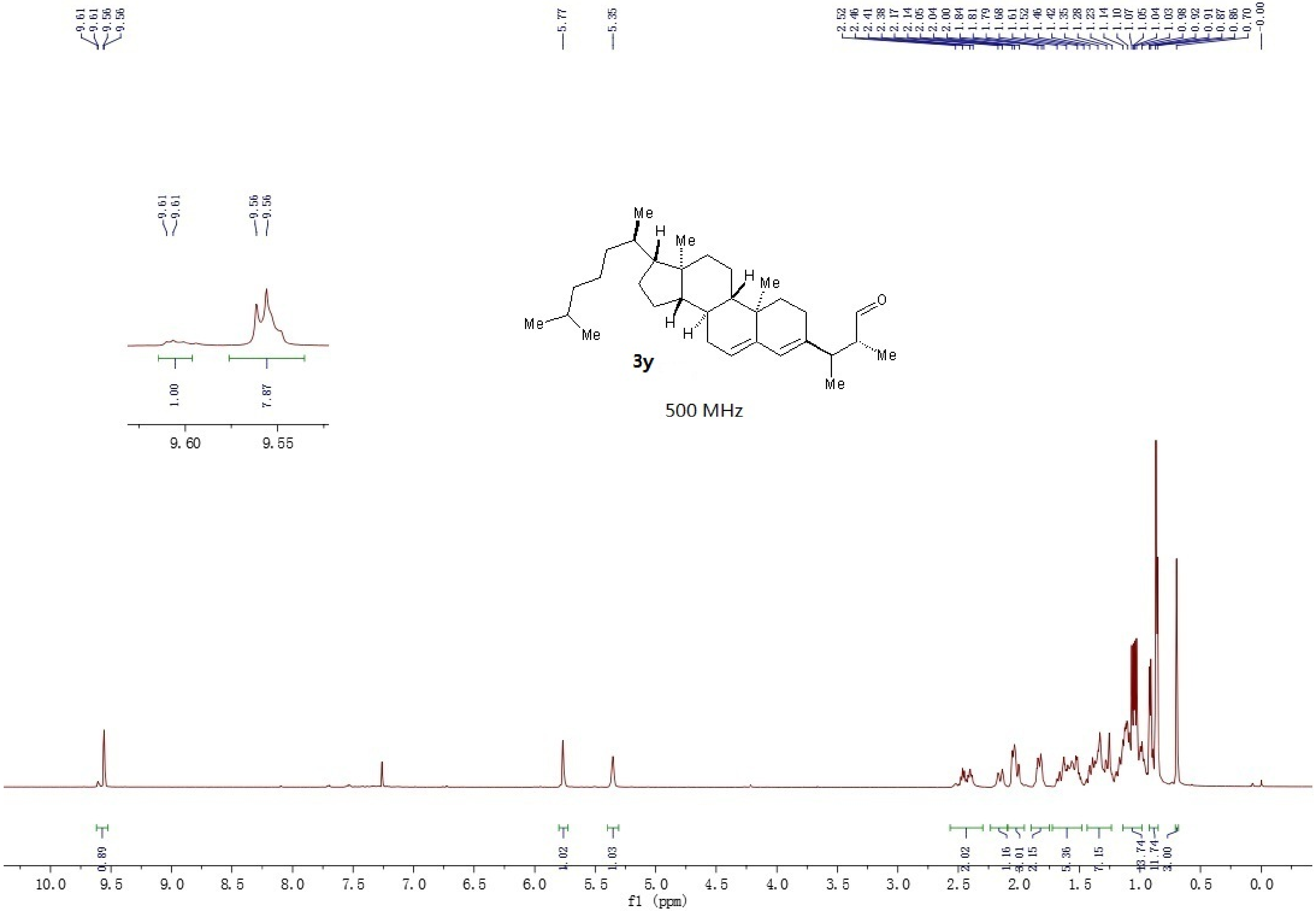
3y
500 MHz
f1 (ppm)

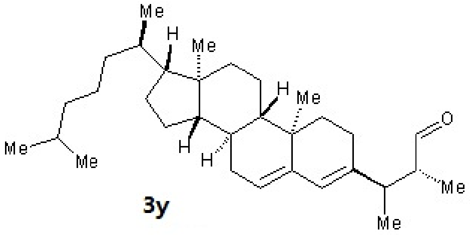
Me
H
Me
H
Me
O
Me
Me
H
H
Me
Me

3y

125 MHz

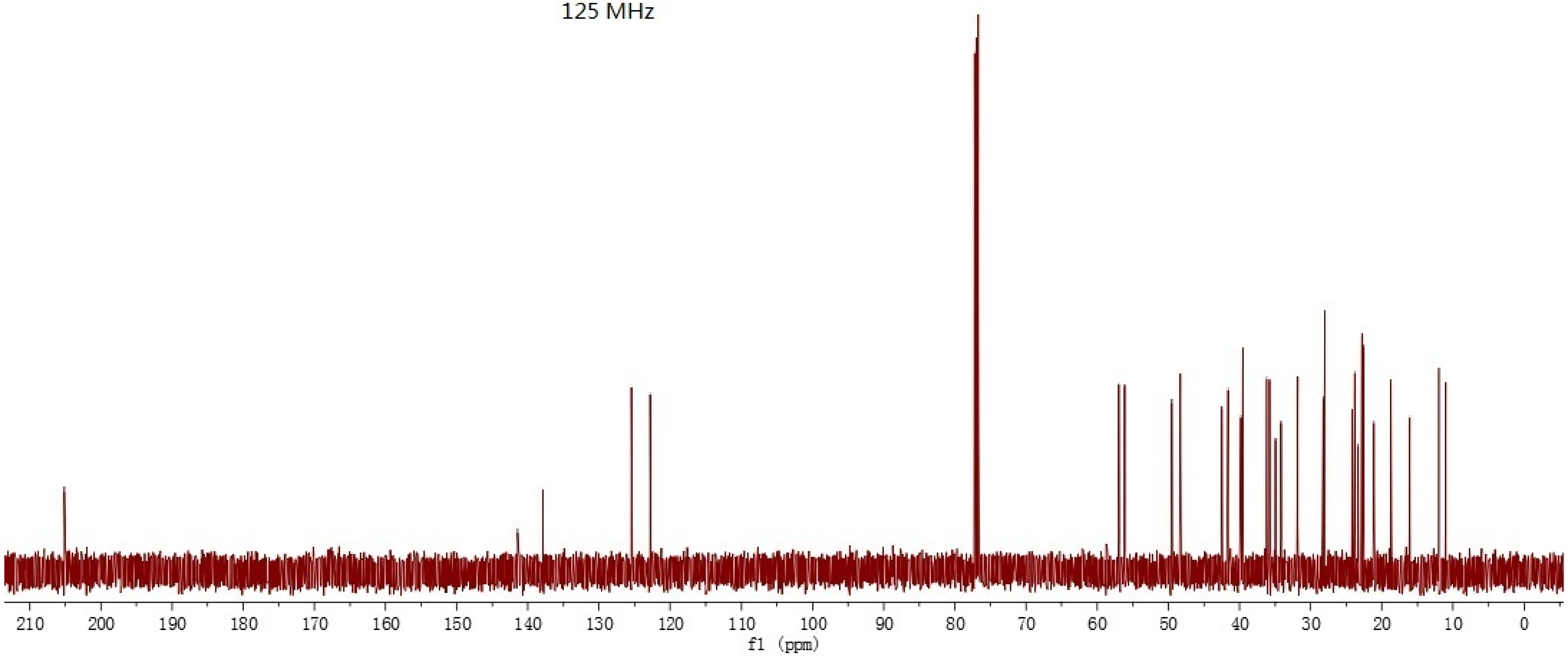
205.12
141.42
137.88
125.45
122.84
77.25
77.00
76.75
56.98
56.16
49.51
48.32
42.47
41.60
39.81
39.52
36.19
35.79
34.96
34.19
31.80
28.24
28.02
24.18
23.83
23.35
22.82
22.56
21.10
18.84
18.72
16.08
12.01
11.04
210 200 190 180 170 160 150 140 130 120 110 100 90 80 70 60 50 40 30 20 10 0
f1 (ppm)

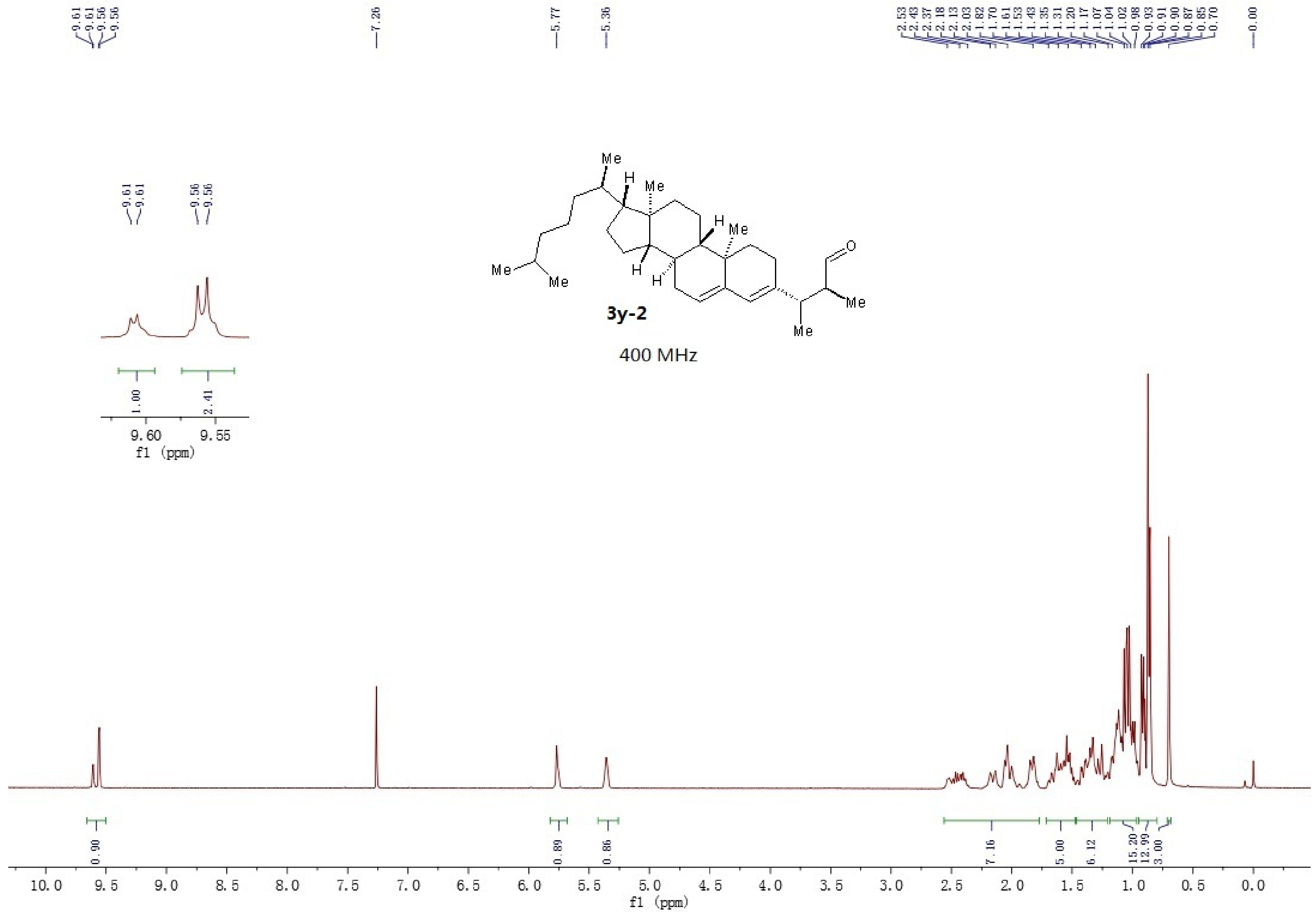

3y-2
400 MHz

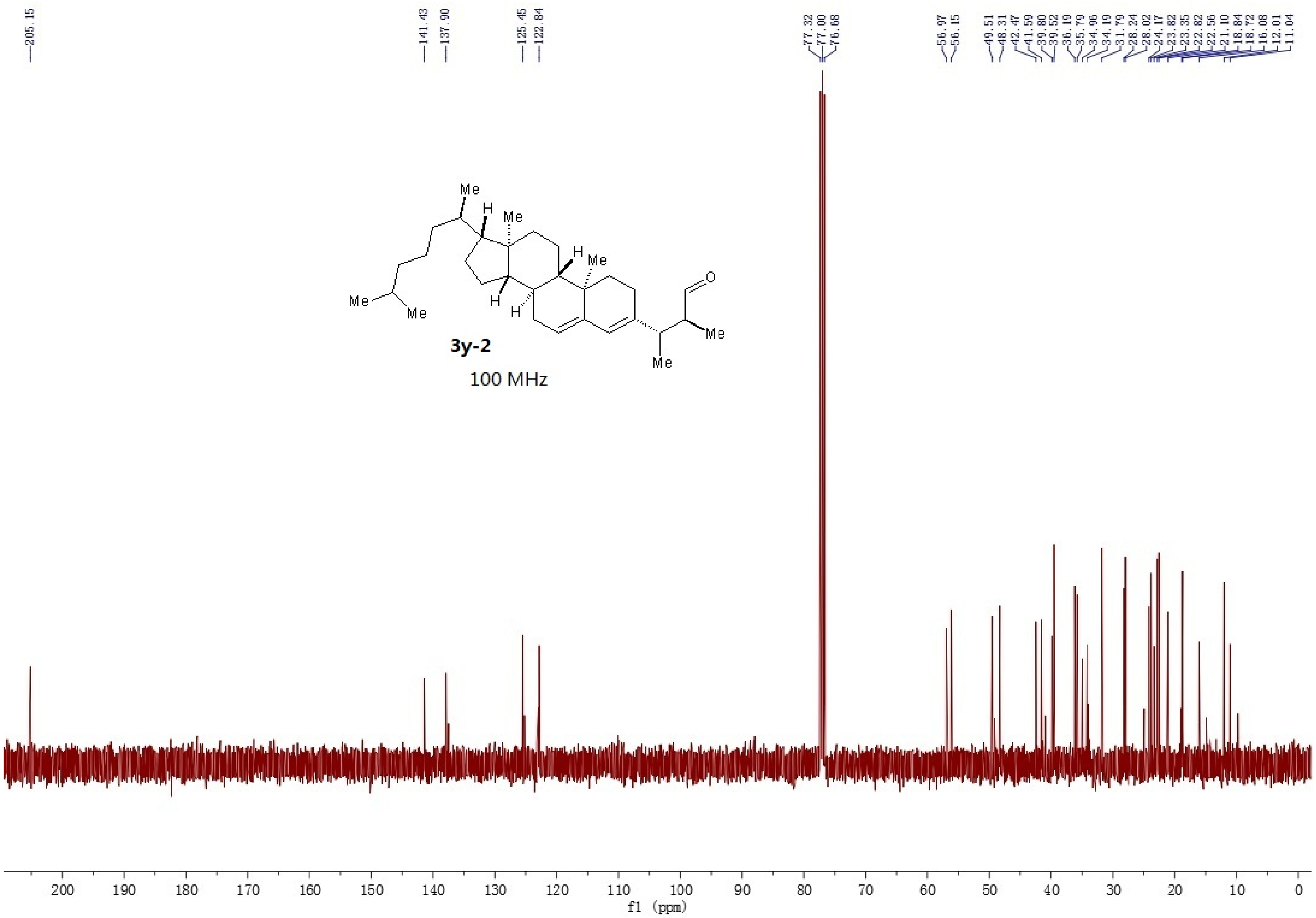
205.15
141.43
137.90
125.45
122.84
77.32
77.00
76.68
56.97
56.15
49.51
48.31
42.47
41.59
39.80
39.52
36.19
35.79
34.96
34.19
31.79
28.24
28.02
24.17
23.82
23.35
22.82
22.56
21.10
18.84
18.72
16.08
12.01
11.04
Me
H
Me
H
Me
O
Me
Me
H
H
Me
Me
3y-2
100 MHz
200 190 180 170 160 150 140 130 120 110 100 90 80 70 60 50 40 30 20 10 0
f1 (ppm)

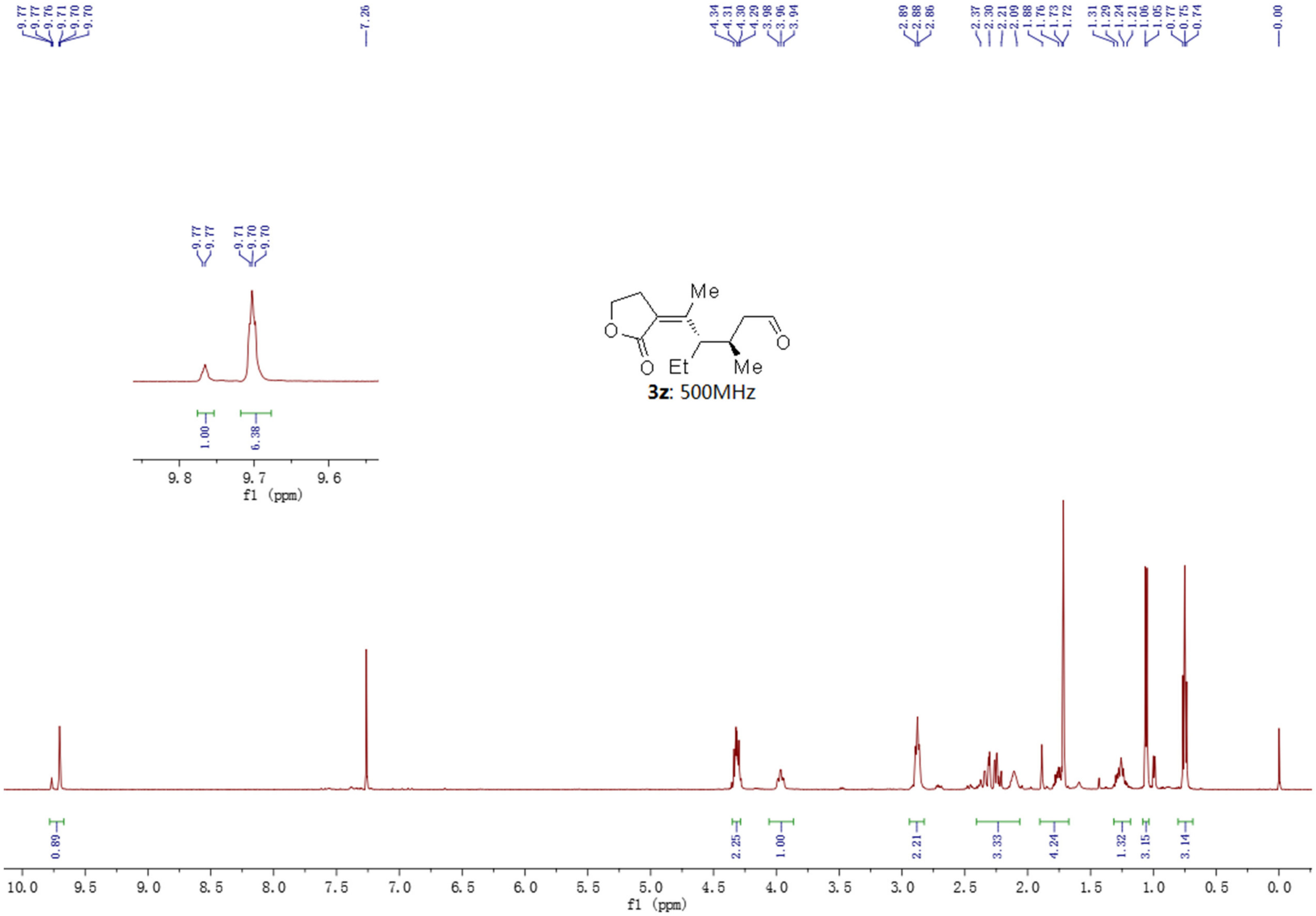

9.77 9.77 9.76 9.71 9.70 9.70
7.26
4.34 4.31 4.30 4.29 3.98 3.96 3.94
2.89 2.88 2.86
2.37 2.30 2.21 2.09 1.88 1.76 1.73 1.72
1.31 1.29 1.24 1.21 1.06 1.05 0.77 0.75 0.74
0.00
Me
O
O
Et
Me
O
3z: 500MHz
1.00
6.38
9.8
9.7
9.6
f1 (ppm)
0.89
2.25
1.00
2.21
3.33
4.24
1.32
3.15
3.14
10.0 9.5 9.0 8.5 8.0 7.5 7.0 6.5 6.0 5.5 5.0 4.5 4.0 3.5 3.0 2.5 2.0 1.5 1.0 0.5 0.0
f1 (ppm)

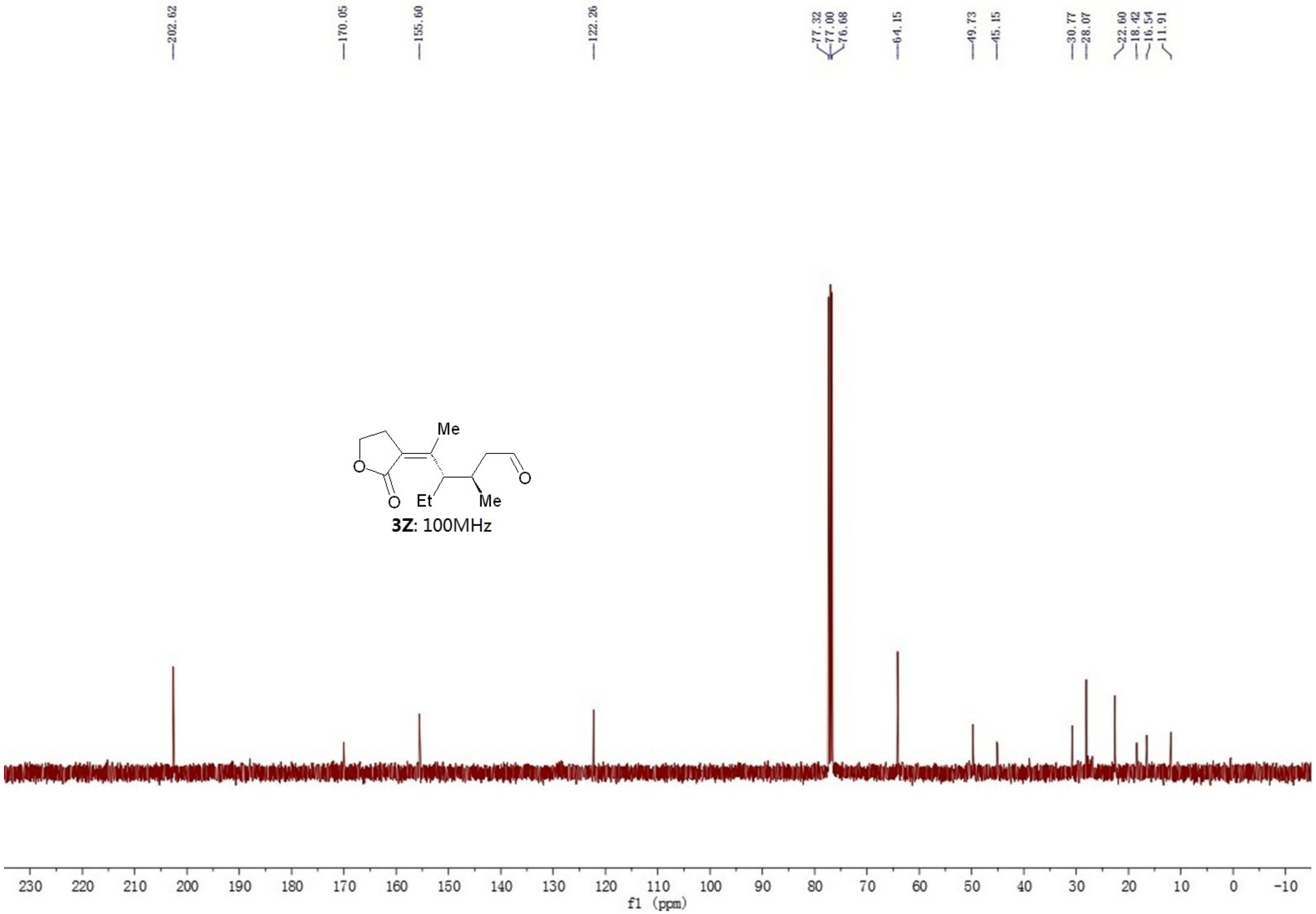

202.62
170.05
155.60
122.26
77.32
77.00
76.68
64.15
49.73
45.15
30.77
28.07
22.60
18.42
16.54
11.91
Me
O
O
Et
Me
O
230 220 210 200 190 180 170 160 150 140 130 120 110 100 90 80 70 60 50 40 30 20 10 0 -10
f1 (ppm)

3Z: 100MHz

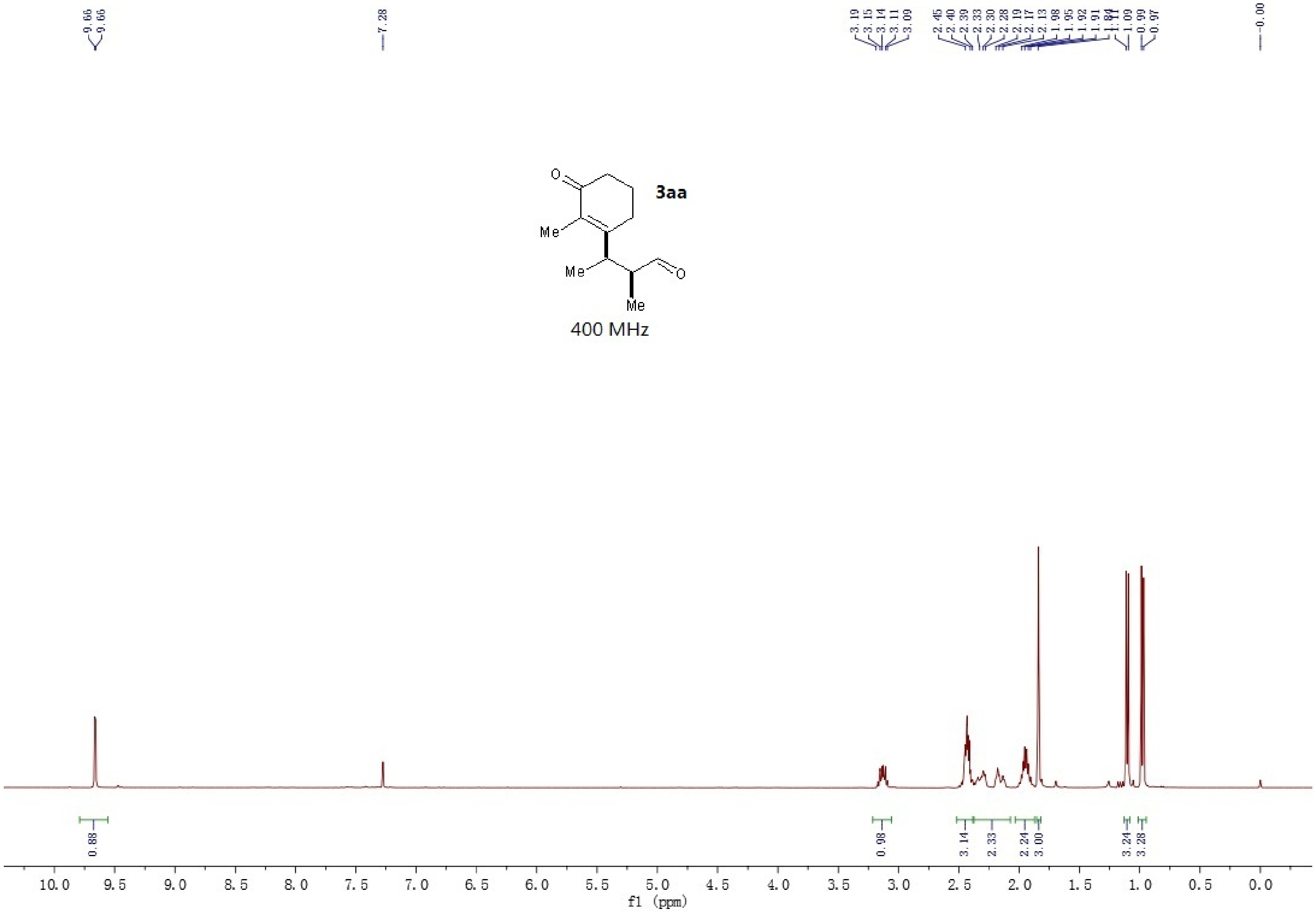
3aa
400 MHz
f1 (ppm)

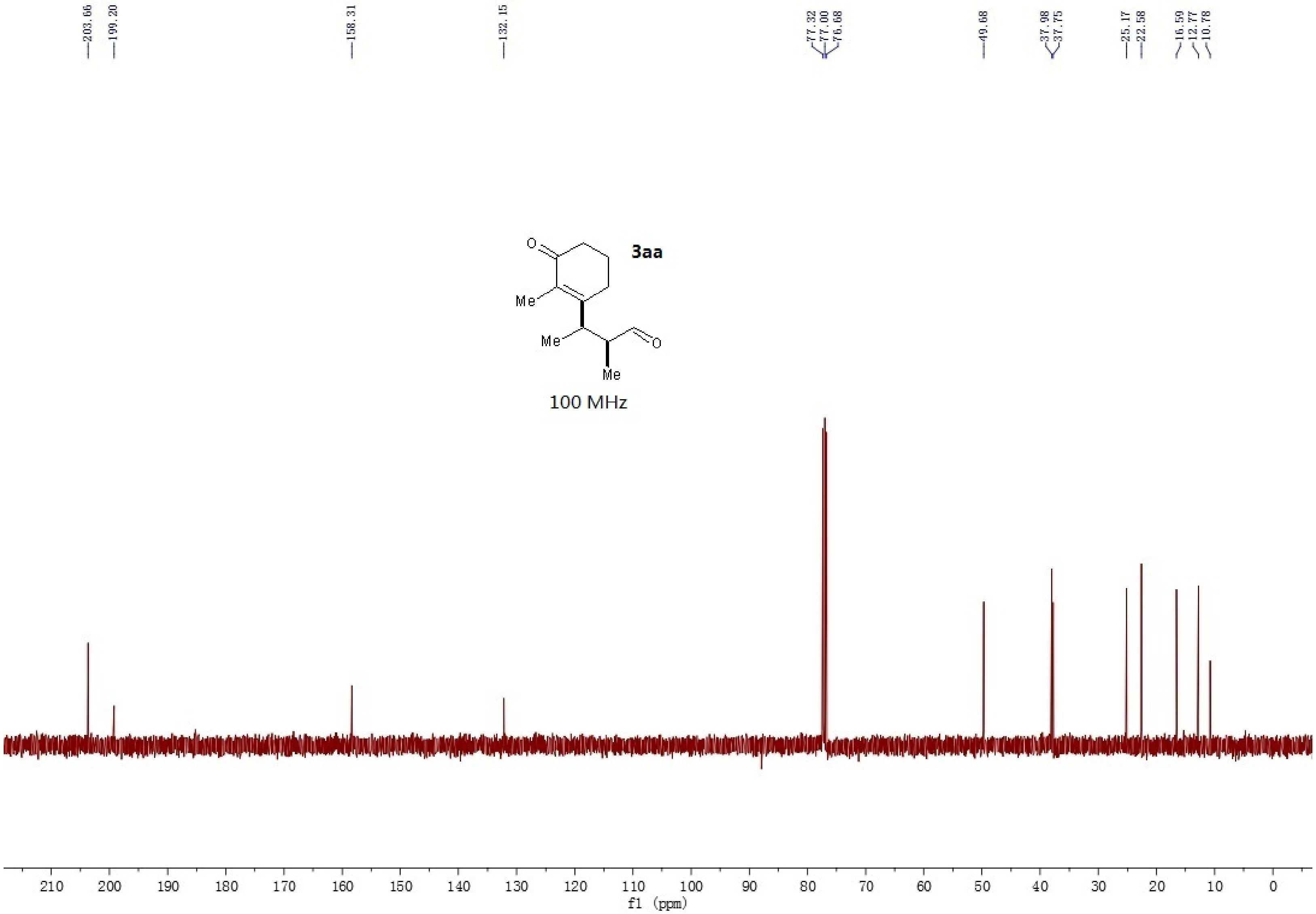
203.66
199.20
158.31
132.15
77.32
77.00
76.68
49.68
37.98
37.75
25.17
22.58
16.59
12.77
10.78
O
3aa
Me
Me
O
Me
100 MHz
210
200
190
180
170
160
150
140
130
120
110
100
90
80
70
60
50
40
30
20
10
0
f1 (ppm)

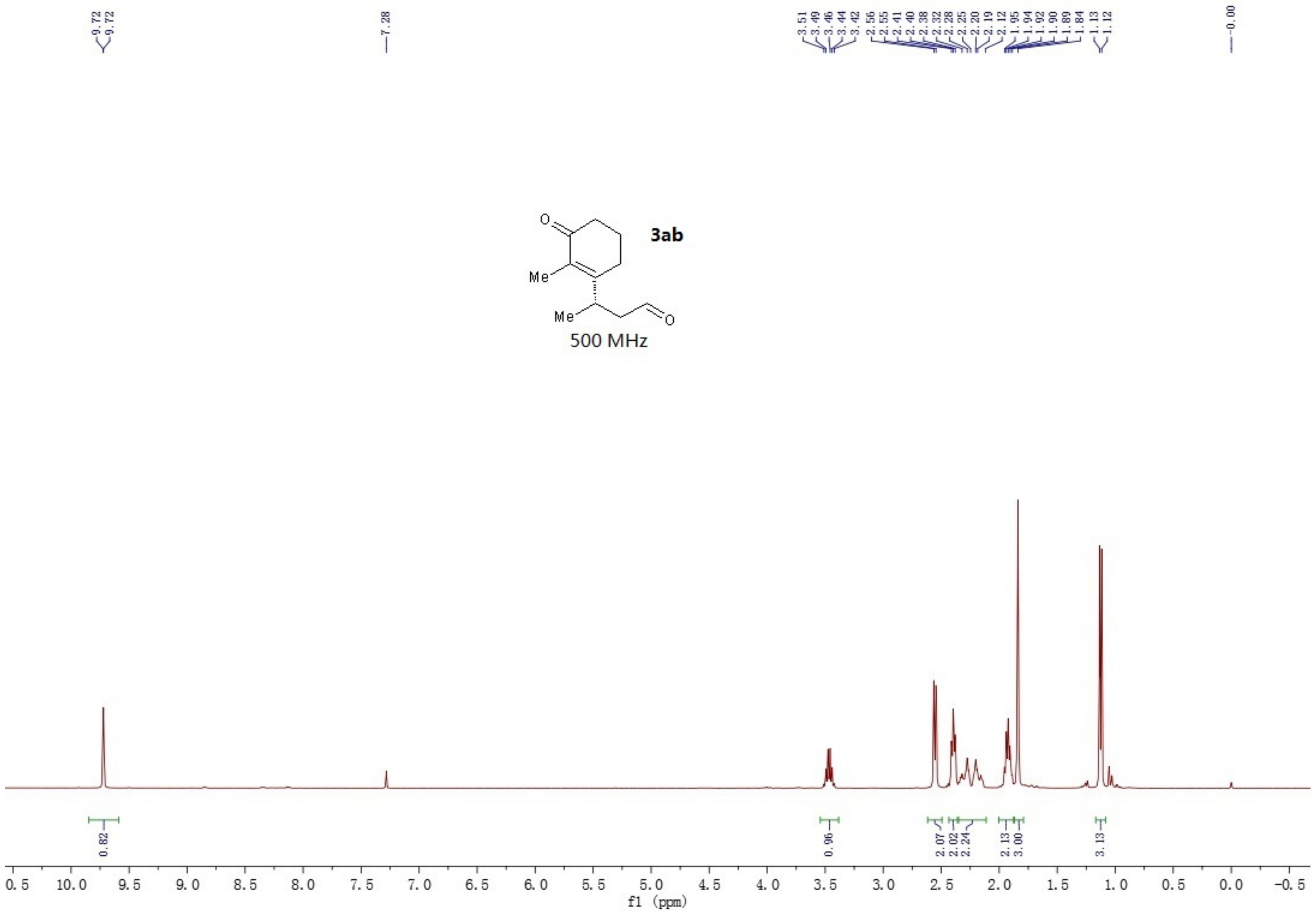
9.72
9.72
7.28
3.51
3.49
3.46
3.44
3.42
2.56
2.55
2.41
2.40
2.38
2.32
2.28
2.25
2.20
2.19
2.12
1.95
1.94
1.92
1.90
1.89
1.84
1.13
1.12
0.00
3ab
Me
Me
O
O
500 MHz
0.82
0.96
2.07
2.02
2.24
2.13
3.00
3.13
f1 (ppm)

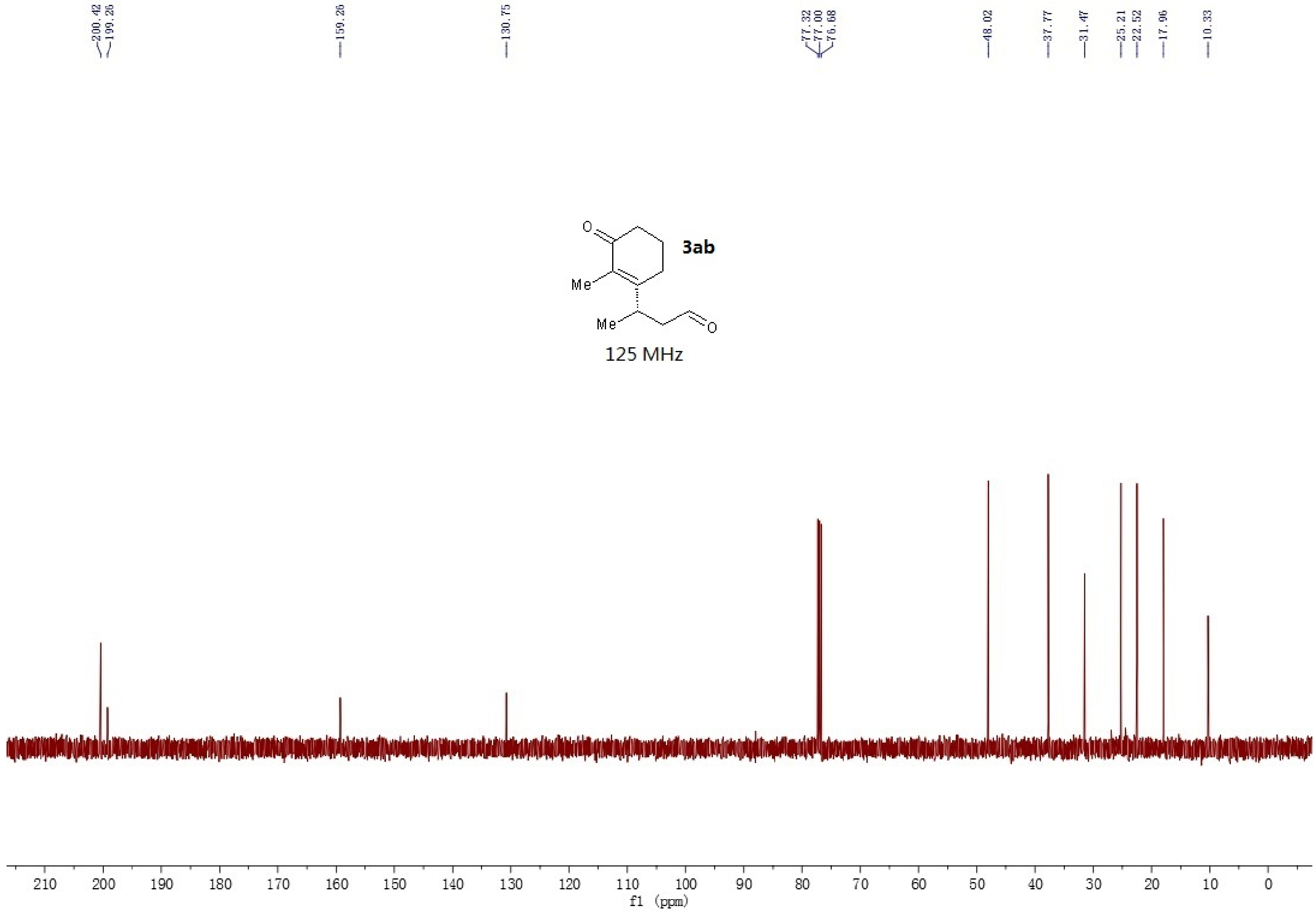
200.42
199.26
159.26
130.75
77.32
77.00
76.68
48.02
37.77
31.47
25.21
22.52
17.96
10.33
O
Me
Me
O
3ab
125 MHz
210 200 190 180 170 160 150 140 130 120 110 100 90 80 70 60 50 40 30 20 10 0
f1 (ppm)

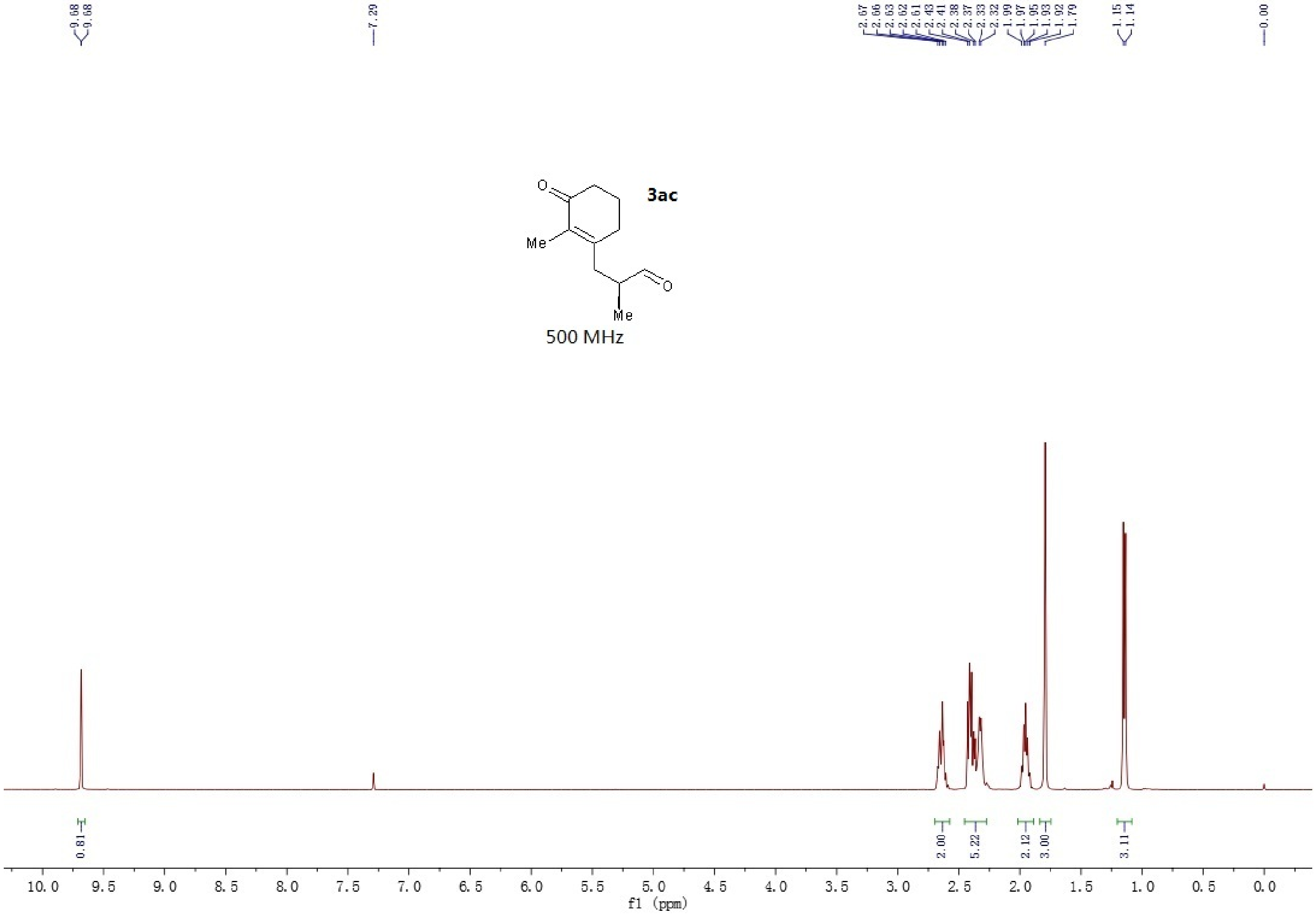

9.68
9.68
7.29
2.67
2.66
2.63
2.62
2.61
2.43
2.41
2.38
2.37
2.33
2.32
1.99
1.97
1.95
1.93
1.92
1.79
1.15
1.14
0.00
3ac
Me
Me
O
O
500 MHz
0.81
2.00
5.22
2.12
3.00
3.11
10.0
9.5
9.0
8.5
8.0
7.5
7.0
6.5
6.0
5.5
5.0
4.5
4.0
3.5
3.0
2.5
2.0
1.5
1.0
0.5
0.0
f1 (ppm)

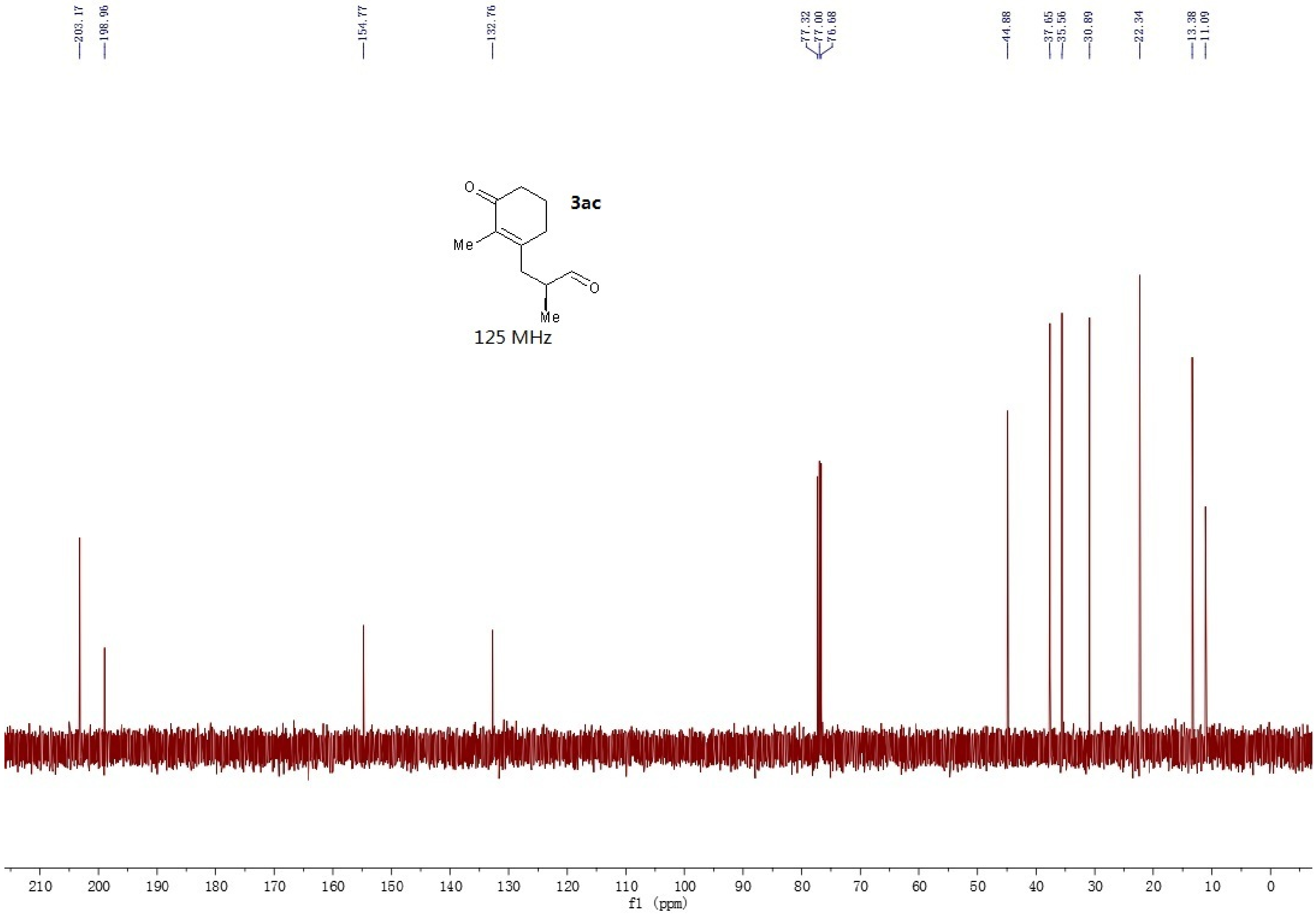
203.17
198.96
154.77
132.76
77.32
77.00
76.68
44.88
37.65
35.56
30.89
22.34
13.38
11.09
3ac
O
Me
O
Me
125 MHz
210 200 190 180 170 160 150 140 130 120 110 100 90 80 70 60 50 40 30 20 10 0
f1 (ppm)

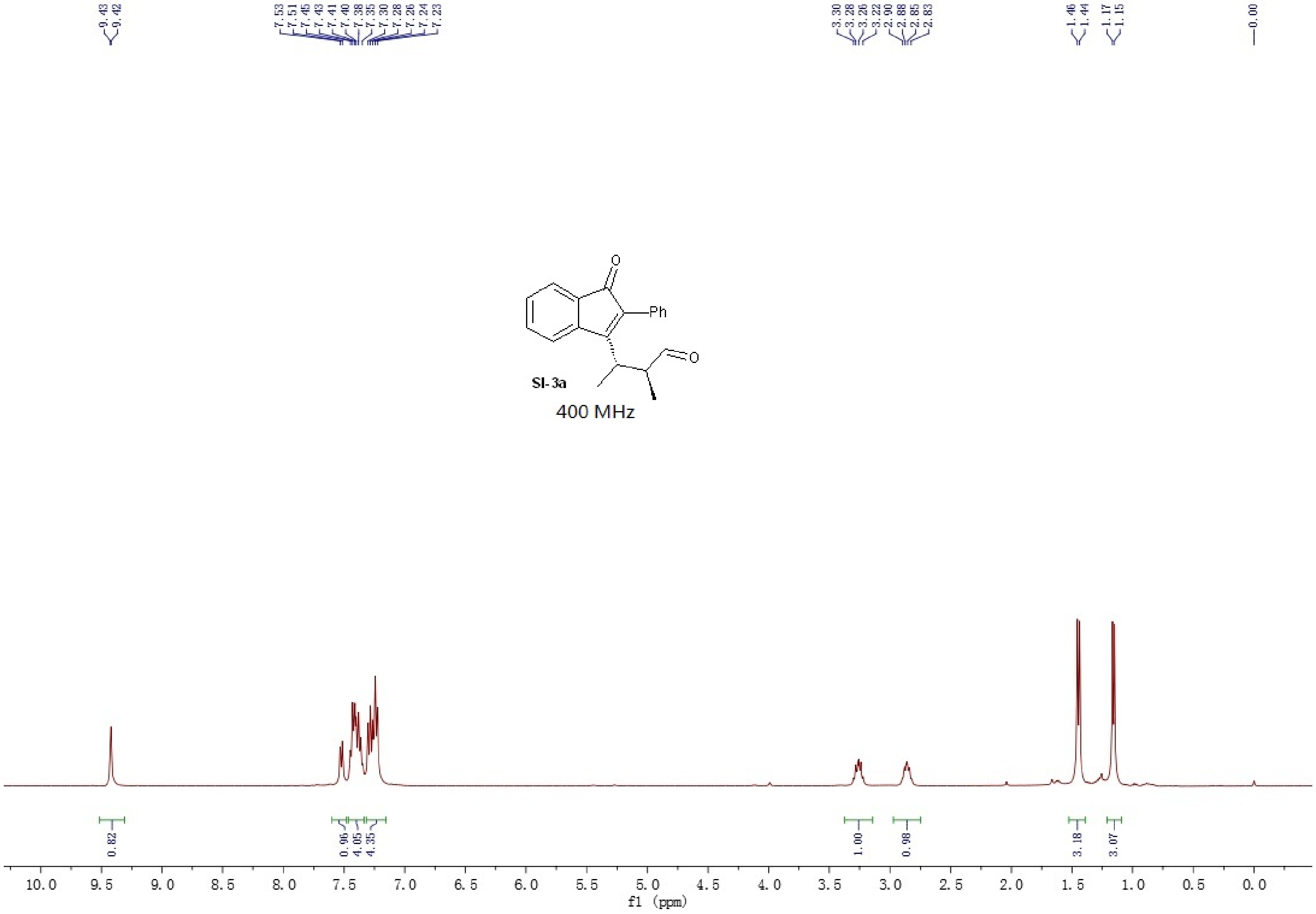
9.43
9.42
7.53
7.51
7.45
7.43
7.41
7.40
7.38
7.35
7.30
7.28
7.26
7.24
7.23
3.30
3.28
3.26
3.22
2.90
2.88
2.85
2.83
1.46
1.44
1.17
1.15
0.00
O
Ph
O
SI-3a
400 MHz
0.82
0.96
4.05
4.35
1.00
0.98
3.18
3.07
10.0 9.5 9.0 8.5 8.0 7.5 7.0 6.5 6.0 5.5 5.0 4.5 4.0 3.5 3.0 2.5 2.0 1.5 1.0 0.5 0.0
f1 (ppm)

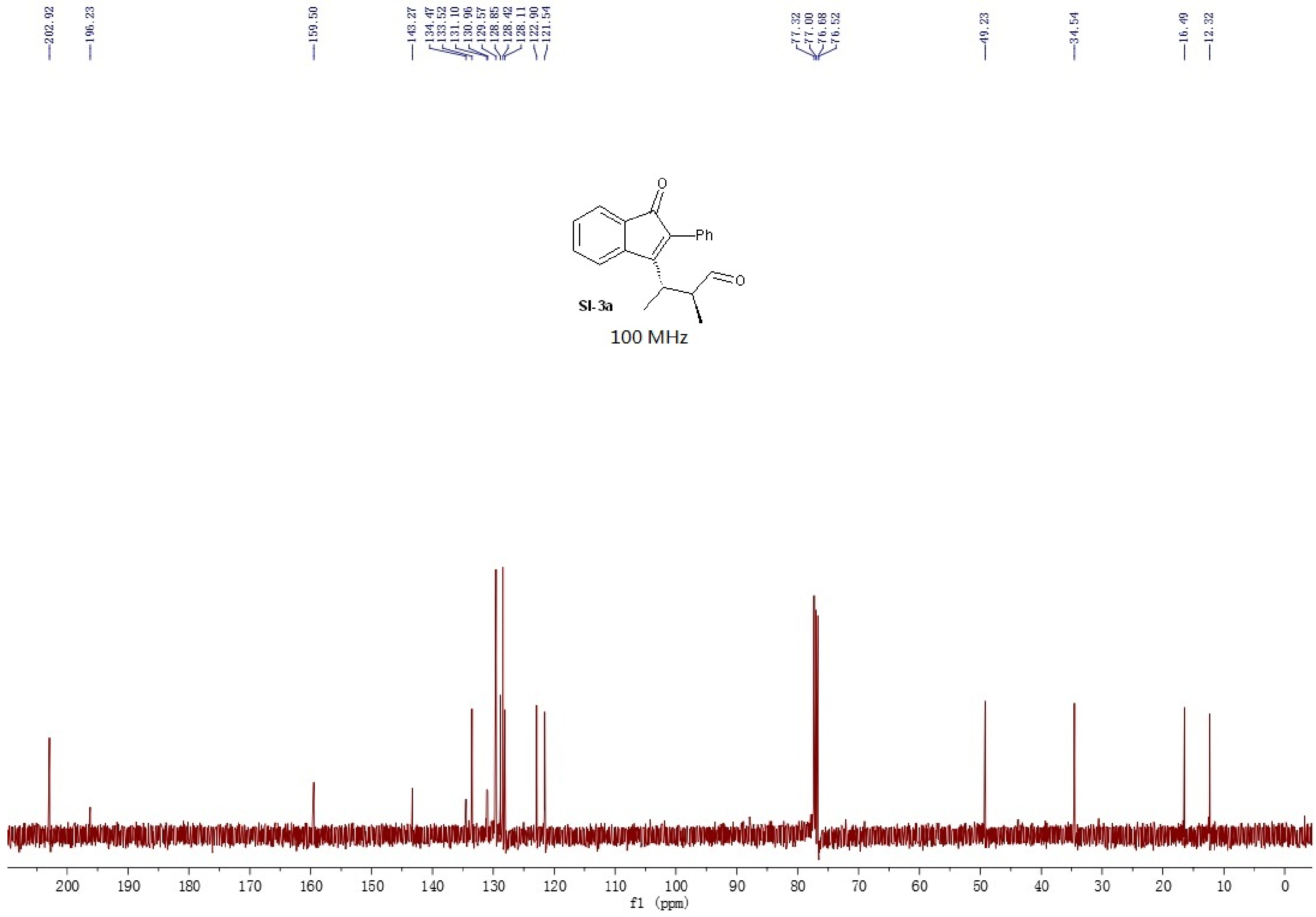
202.92
196.23
159.50
143.27
134.47
133.52
131.10
130.96
129.57
128.85
128.42
128.11
122.90
121.54
77.32
77.00
76.68
76.52
49.23
34.54
16.49
12.32
O
Ph
O
SI-3a
100 MHz
200 190 180 170 160 150 140 130 120 110 100 90 80 70 60 50 40 30 20 10 0
f1 (ppm)

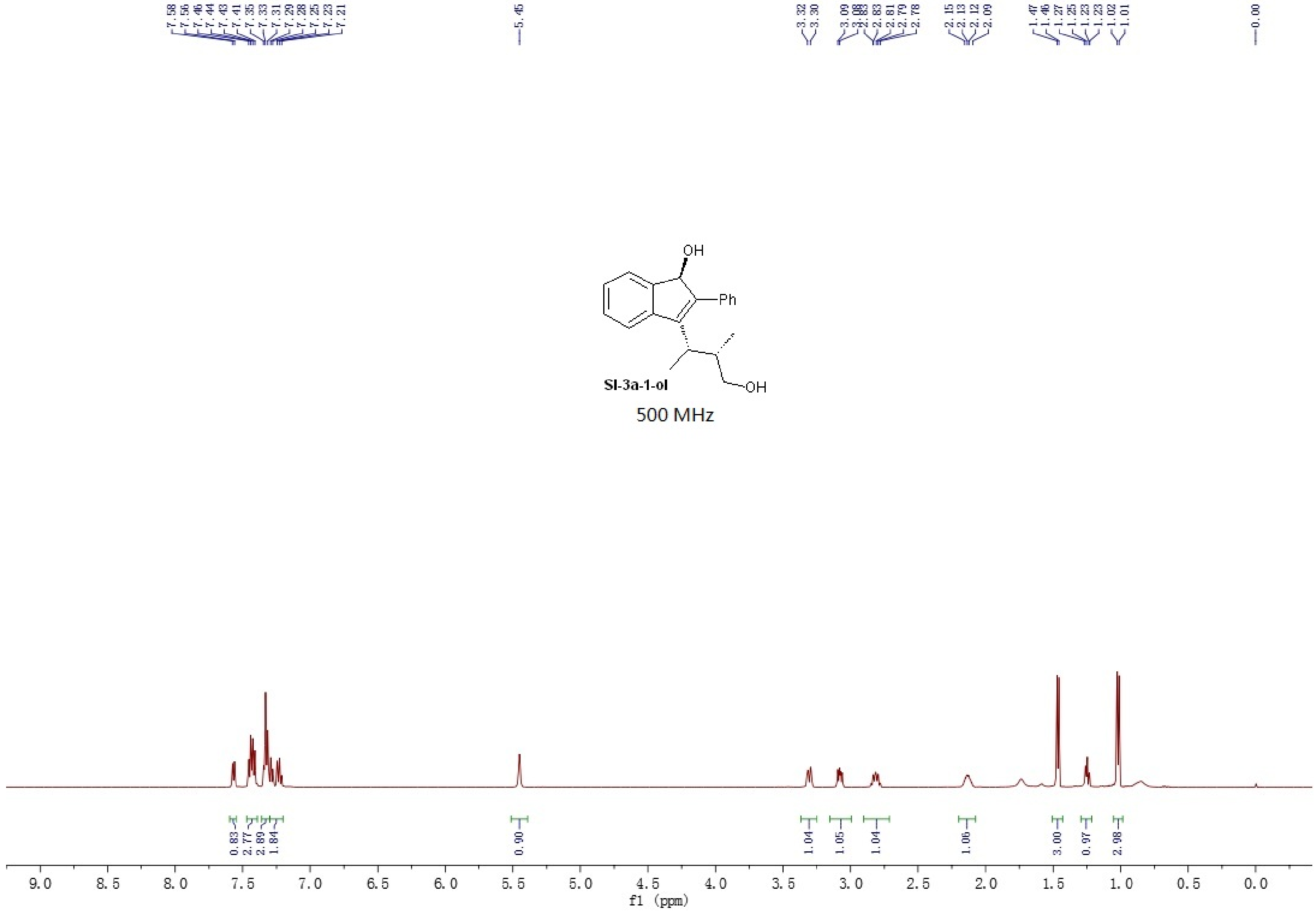
OH
Ph
OH
SI-3a-1-ol
500 MHz
f1 (ppm)

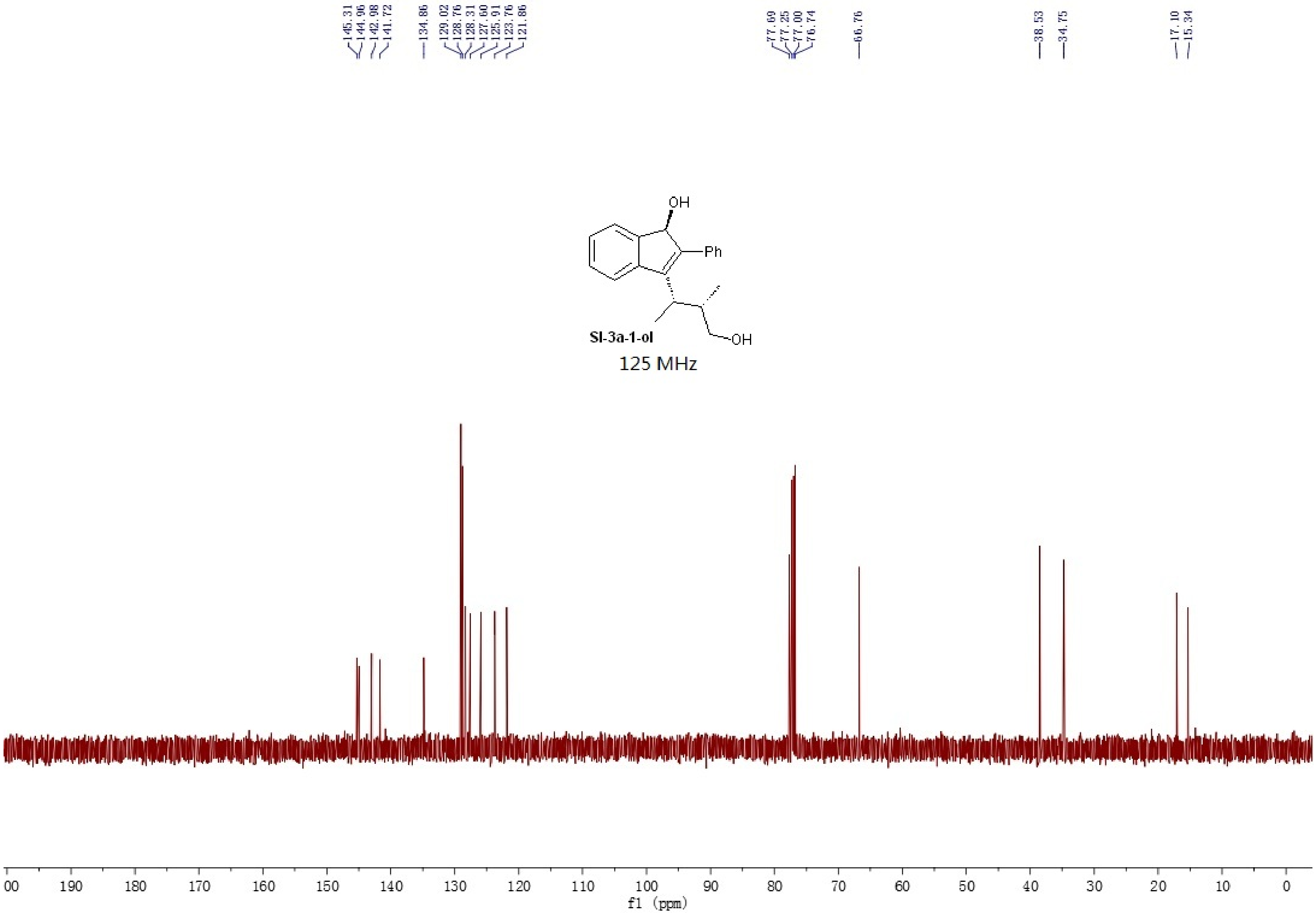
145.31
144.96
142.98
141.72
134.86
129.02
128.76
128.31
127.60
125.91
123.76
121.86
77.09
77.25
77.00
76.74
66.76
38.53
34.75
17.10
15.34
OH
Ph
OH
SI-3a-1-ol
125 MHz
f1 (ppm)

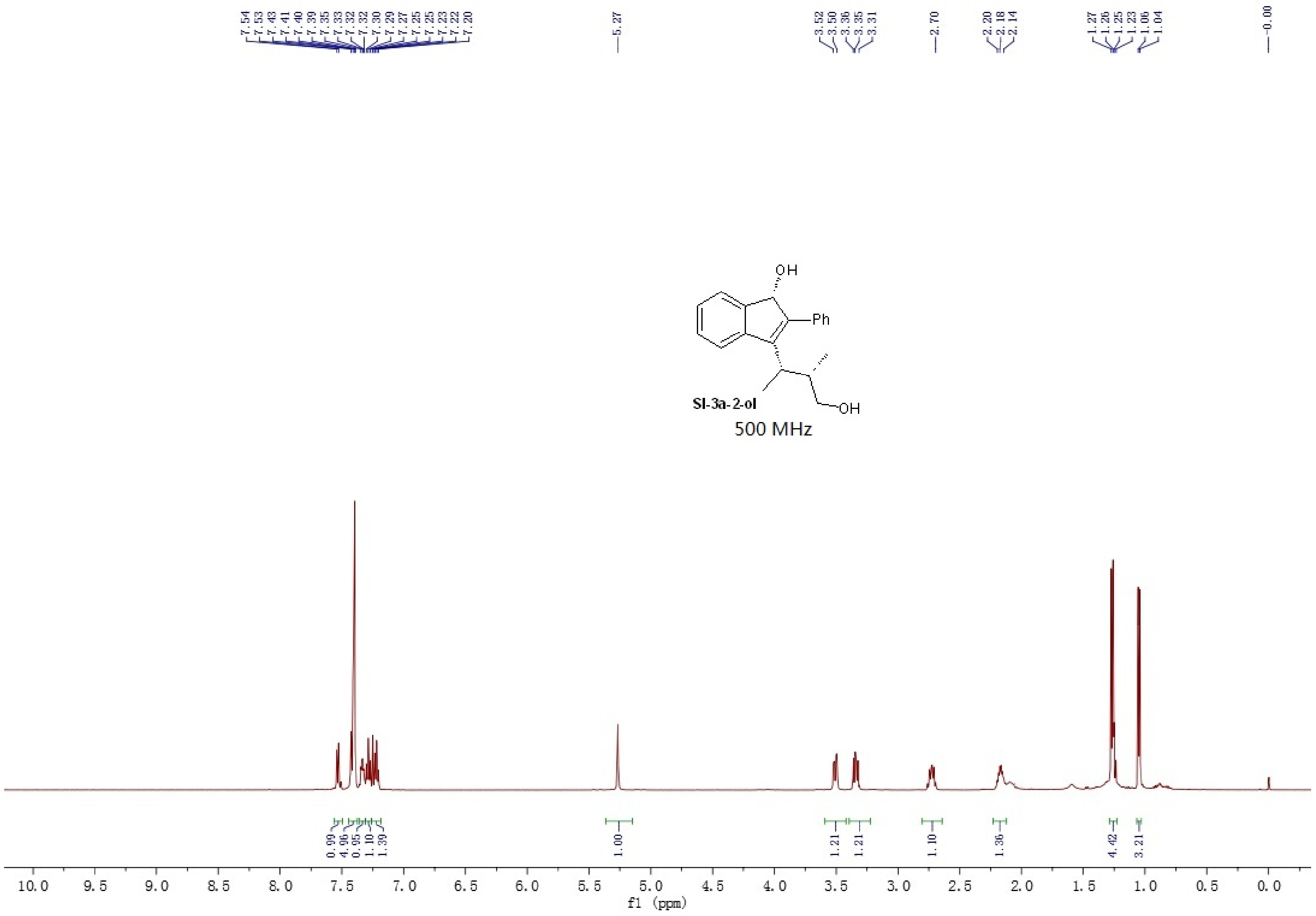
7.54
7.53
7.43
7.41
7.40
7.39
7.35
7.33
7.32
7.32
7.30
7.29
7.27
7.25
7.25
7.23
7.22
7.20
5.27
3.52
3.50
3.36
3.35
3.31
2.70
2.20
2.18
2.14
1.27
1.26
1.25
1.23
1.06
1.04
0.00
OH
Ph
OH
SI-3a-2-ol
500 MHz
0.99
4.96
0.95
1.10
1.39
1.00
1.21
1.21
1.10
1.36
4.42
3.21
10.0
9.5
9.0
8.5
8.0
7.5
7.0
6.5
6.0
5.5
5.0
4.5
4.0
3.5
3.0
2.5
2.0
1.5
1.0
0.5
0.0
f1 (ppm)

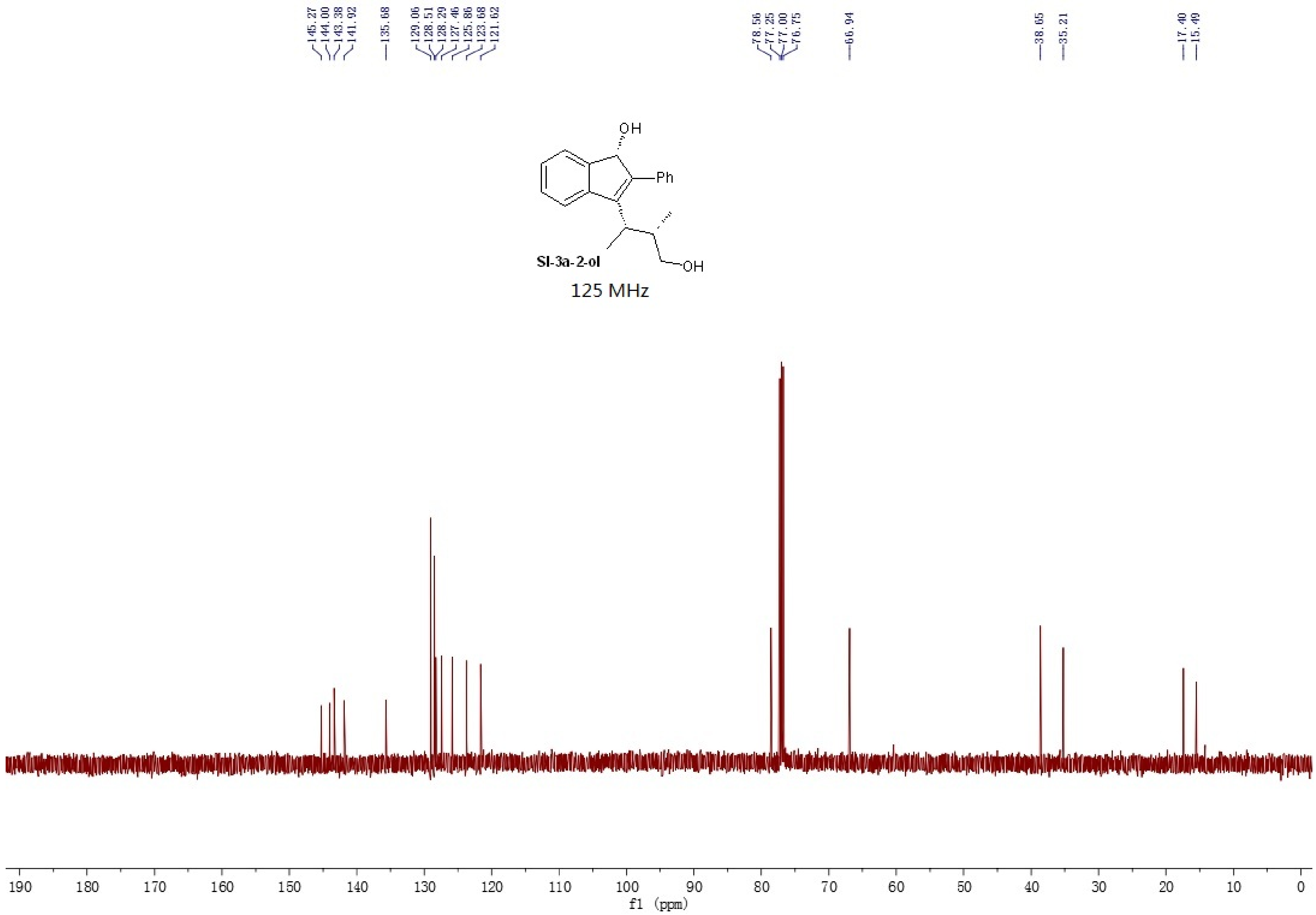
145.27
144.00
143.38
141.92
135.68
129.06
128.51
128.29
127.46
125.86
123.68
121.62
78.56
77.25
77.00
76.75
66.94
38.65
35.21
17.40
15.49
OH
Ph
OH
SI-3a-2-ol
125 MHz
190
180
170
160
150
140
130
120
110
100
90
80
70
60
50
40
30
20
10
0
f1 (ppm)

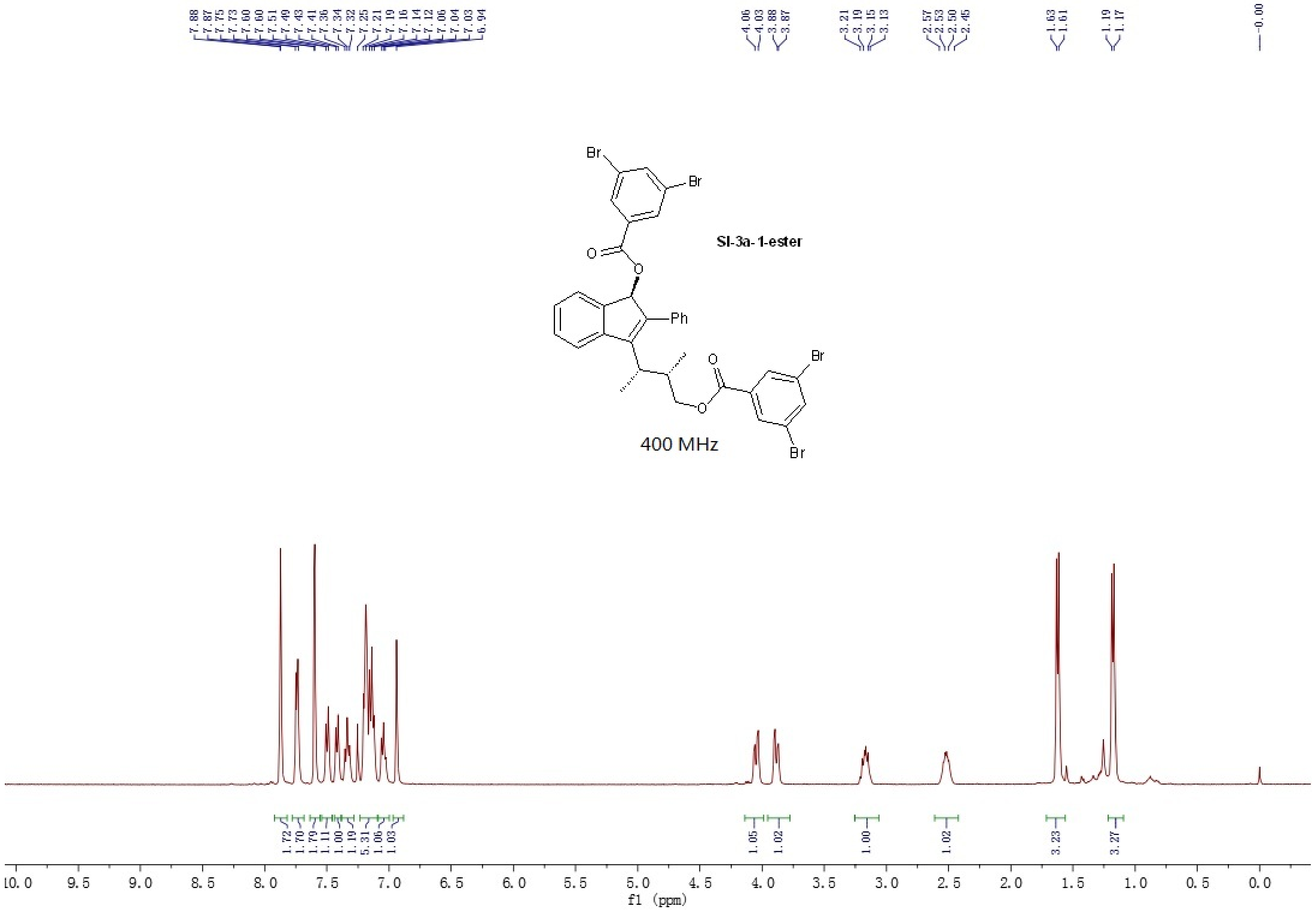

SI-3a-1-ester
400 MHz
f1 (ppm)

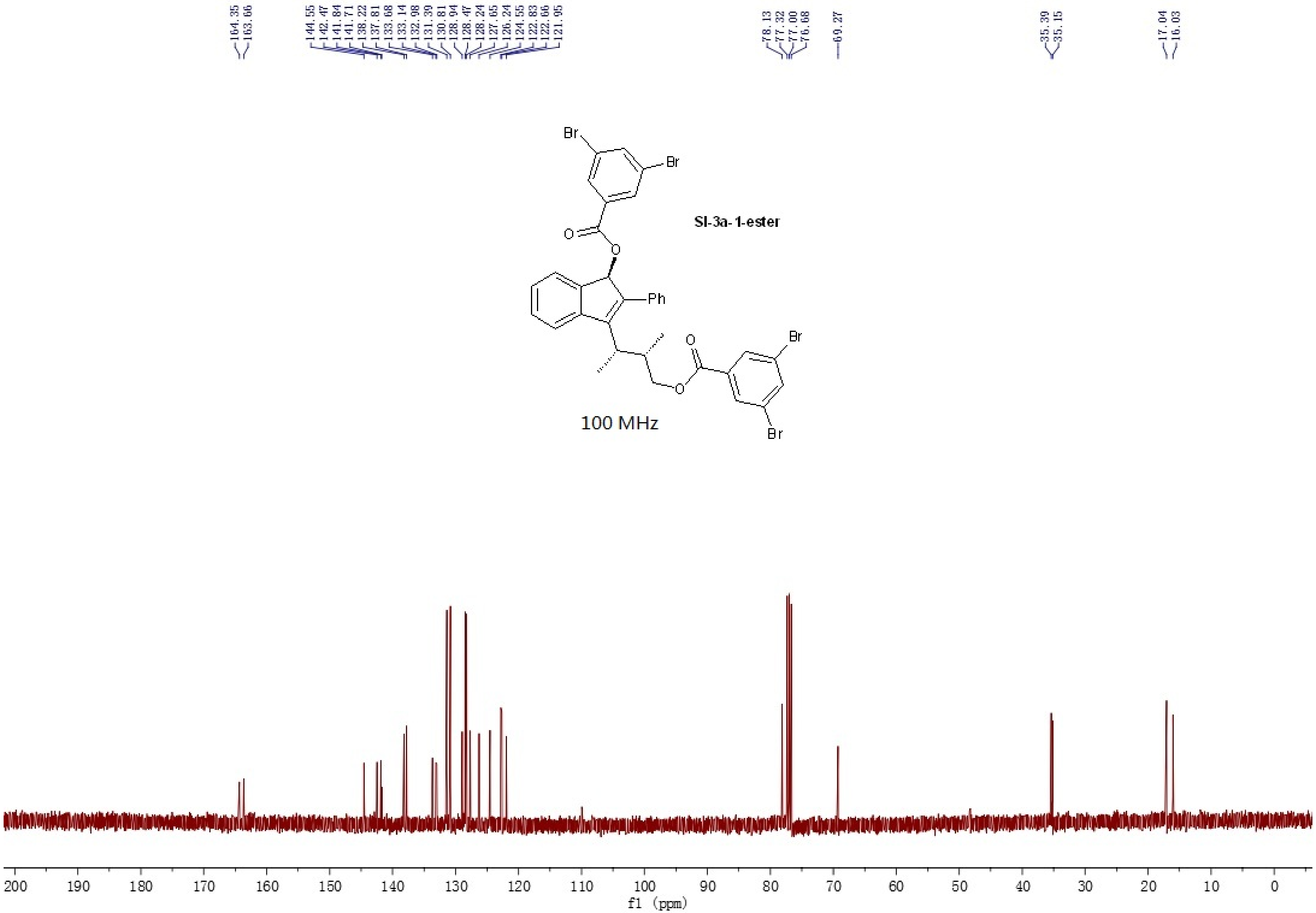
164.35
163.66
144.55
142.47
141.84
141.71
138.22
137.81
133.68
133.14
132.98
131.39
130.81
128.94
128.47
128.24
127.65
126.24
124.55
122.83
122.66
121.95
78.13
77.32
77.00
76.68
69.27
35.39
35.15
17.04
16.03
Br
Br
O
O
Ph
O
O
Br
Br
SI-3a-1-ester
100 MHz
200 190 180 170 160 150 140 130 120 110 100 90 80 70 60 50 40 30 20 10 0
f1 (ppm)

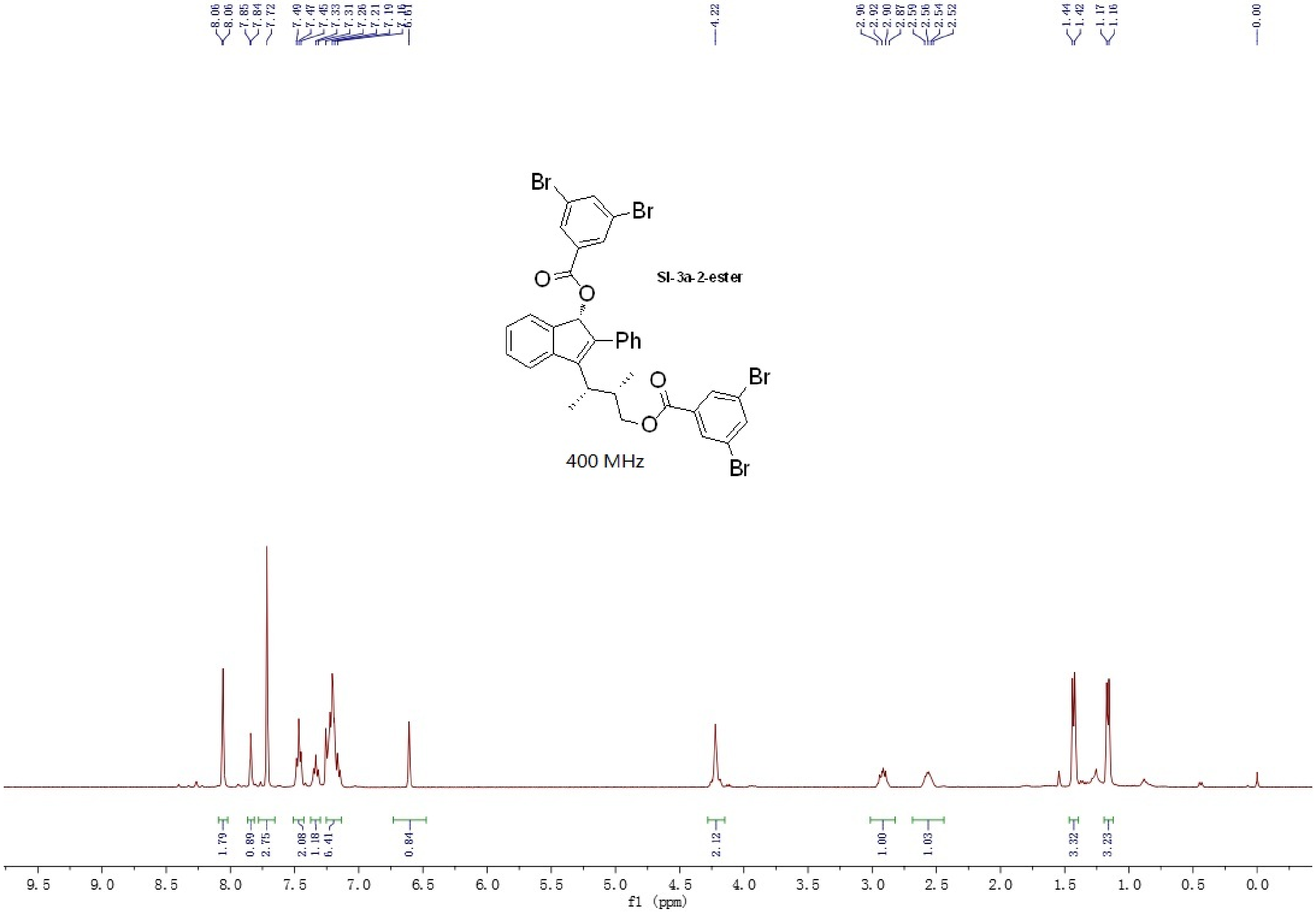

8.06
8.06
7.85
7.84
7.72
7.49
7.47
7.45
7.33
7.31
7.26
7.21
7.19
6.61
4.22
2.96
2.92
2.90
2.87
2.59
2.56
2.54
2.52
1.44
1.42
1.17
1.16
0.00
Br
Br
O
O
Ph
O
O
Br
Br
SI-3a-2-ester
400 MHz
1.79
0.89
2.75
2.08
1.18
6.41
0.84
2.12
1.00
1.03
3.32
3.23
9.5
9.0
8.5
8.0
7.5
7.0
6.5
6.0
5.5
5.0
4.5
4.0
3.5
3.0
2.5
2.0
1.5
1.0
0.5
0.0
f1 (ppm)

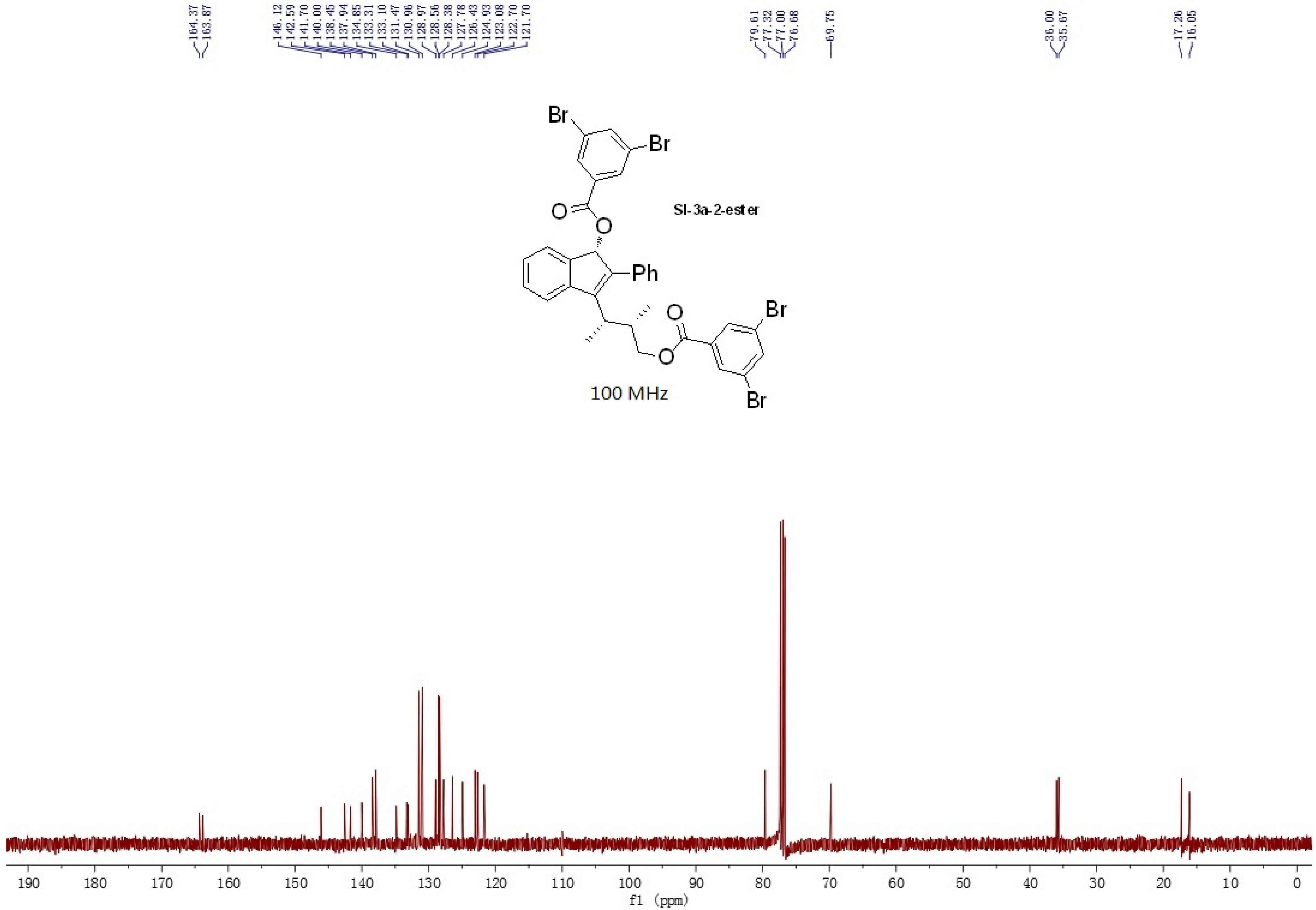
164.37
163.87
146.12
142.59
141.70
140.00
138.45
137.94
134.85
133.31
133.10
131.47
130.96
128.97
128.56
128.38
127.78
126.43
124.93
123.08
122.70
121.70
79.61
77.32
77.00
76.68
69.75
36.00
35.67
17.26
16.05
Br
Br
O
O
Ph
SI-3a-2-ester
O
Br
O
Br
100 MHz
190 180 170 160 150 140 130 120 110 100 90 80 70 60 50 40 30 20 10 0
f1 (ppm)
